# Practical Guide to Measuring Wetland Carbon Pools and Fluxes

**DOI:** 10.1007/s13157-023-01722-2

**Published:** 2023-11-28

**Authors:** Sheel Bansal, Irena F. Creed, Brian A. Tangen, Scott D. Bridgham, Ankur R. Desai, Ken W. Krauss, Scott C. Neubauer, Gregory B. Noe, Donald O. Rosenberry, Carl Trettin, Kimberly P. Wickland, Scott T. Allen, Ariane Arias-Ortiz, Anna R. Armitage, Dennis Baldocchi, Kakoli Banerjee, David Bastviken, Peter Berg, Matthew J. Bogard, Alex T. Chow, William H. Conner, Christopher Craft, Courtney Creamer, Tonya DelSontro, Jamie A. Duberstein, Meagan Eagle, M. Siobhan Fennessy, Sarah A. Finkelstein, Mathias Göckede, Sabine Grunwald, Meghan Halabisky, Ellen Herbert, Mohammad M. R. Jahangir, Olivia F. Johnson, Miriam C. Jones, Jeffrey J. Kelleway, Sara Knox, Kevin D. Kroeger, Kevin A. Kuehn, David Lobb, Amanda L. Loder, Shizhou Ma, Damien T. Maher, Gavin McNicol, Jacob Meier, Beth A. Middleton, Christopher Mills, Purbasha Mistry, Abhijit Mitra, Courtney Mobilian, Amanda M. Nahlik, Sue Newman, Jessica L. O’Connell, Patty Oikawa, Max Post van der Burg, Charles A. Schutte, Changchun Song, Camille L. Stagg, Jessica Turner, Rodrigo Vargas, Mark P. Waldrop, Marcus B. Wallin, Zhaohui Aleck Wang, Eric J. Ward, Debra A. Willard, Stephanie Yarwood, Xiaoyan Zhu

**Affiliations:** 1 U.S. Geological Survey, Northern Prairie Wildlife Research Center, Jamestown, ND, USA; 2 Department of Physical and Environmental Sciences, University of Toronto Scarborough, Toronto, ON, Canada; 3 Institute of Ecology and Evolution, University of Oregon, Eugene, OR, USA; 4 Department of Atmospheric and Oceanic Sciences, University of Wisconsin-Madison, Madison, WI, USA; 5 U.S. Geological Survey, Wetland and Aquatic Research Center, Lafayette, LA, USA; 6 Department of Biology, Virginia Commonwealth University, Richmond, VA, USA; 7 U.S. Geological Survey, Florence Bascom Geoscience Center, Reston, VA, USA; 8 U.S. Geological Survey, Water Mission Area, Lakewood, CO, USA; 9 U.S. Forest Service, Pacific Southwest Research Station, Davis, CA, USA; 10 U.S. Geological Survey, Geosciences and Environmental Change Science Center, Denver, CO, USA; 11 Department of Natural Resources and Environmental Science, University of Nevada, Reno, Reno, NV, USA; 12 Ecosystem Science Division, Department of Environmental Science, Policy and Management, University of California, Berkeley, CA, USA; 13 Department of Marine Biology, Texas A&M University at Galveston, Galveston, TX, USA; 14 Department of Environmental Science, Policy and Management, University of California, Berkeley, CA, USA; 15 Department of Biodiversity and Conservation of Natural Resources, Central University of Odisha, Koraput, Odisha, India; 16 Department of Thematic Studies – Environmental Change, Linköping University, Linköping, Sweden; 17 Department of Environmental Sciences, University of Virginia, Charlottesville, VA, USA; 18 Department of Biological Sciences, University of Lethbridge, Lethbridge, AB, Canada; 19 Earth and Environmental Sciences Programme, The Chinese University of Hong Kong, Shatin, Hong Kong SAR, China; 20 Baruch Institute of Coastal Ecology and Forest Science, Clemson University, Georgetown, SC, USA; 21 O’Neill School of Public and Environmental Affairs, Indiana University, Bloomington, IN, USA; 22 U.S. Geological Survey, Geology, Minerals, Energy and Geophysics Science Center, Menlo Park, CA, USA; 23 Department of Earth and Environmental Sciences, University of Waterloo, Waterloo, ON, Canada; 24 U.S. Geological Survey, Woods Hole Coastal & Marine Science Center, Woods Hole, MA, USA; 25 Biology Department, Kenyon College, Gambier, OH, USA; 26 Department of Earth Sciences, University of Toronto, Toronto, ON, Canada; 27 Department for Biogeochemical Signals, Max Planck Institute for Biogeochemistry, Jena, Germany; 28 Soil, Water and Ecosystem Sciences Department, University of Florida, Gainesville, FL, USA; 29 School of Environmental and Forest Sciences, University of Washington, Seattle, WA, USA; 30 Ducks Unlimited, Memphis, TN, USA; 31 Department of Soil Science, Bangladesh Agricultural University, Mymensingh, Bangladesh; 32 Departments of Biology and Environmental Studies, Kent State University, Kent, OH, USA; 33 School of Earth, Atmospheric and Life Sciences and Environmental Futures Research Centre, University of Wollongong, Wollongong, NSW, Australia; 34 Department of Geography, McGill University, Montreal, Canada; 35 School of Biological, Environmental, and Earth Sciences, University of Southern Mississippi, Hattiesburg, MS, USA; 36 Department of Soil Science, University of Manitoba, Winnipeg, MB, Canada; 37 Department of Geography, University of Toronto, Toronto, ON, Canada; 38 School of Environment and Sustainability, University of Saskatchewan, Saskatoon, SK, Canada; 39 Faculty of Science and Engineering, Southern Cross University, Lismore, NSW, Australia; 40 Department of Earth and Environmental Sciences, University of Illinois Chicago, Chicago, IL, USA; 41 U.S. Geological Survey, Geology, Geophysics, and Geochemistry Science Center, Denver, CO, USA; 42 Department of Marine Science, University of Calcutta, Kolkata, West Bengal, India; 43 Office of Research and Development, Center for Public Health and Environmental Assessments, Pacific Ecological Systems Division, U.S. Environmental Protection Agency, Corvallis, OR, USA; 44 South Florida Water Management District, Everglades Systems Assessment Section, West Palm Beach, FL, USA; 45 Department of Ecosystem Science and Sustainability, Colorado State University, Fort Collins, CO, USA; 46 Department of Earth and Environmental Sciences, California State University, East Bay, Hayward, CA, USA; 47 Department of Environmental Science, Rowan University, Glassboro, NJ, USA; 48 Key Laboratory of Wetland Ecology and Environment, Northeast Institute of Geography and Agroecology, Chinese Academy of Sciences, Changchun, China; 49 Freshwater and Marine Science, University of Wisconsin-Madison, Madison, WI, USA; 50 Department of Plant and Soil Sciences, University of Delaware, Newark, DE, USA; 51 Department of Aquatic Sciences and Assessment, Swedish University of Agricultural Sciences, Uppsala, Sweden; 52 Department of Marine Chemistry and Geochemistry, Woods Hole Oceanographic Institution, Woods Hole, MA, USA; 53 Environmental Science and Technology, University of Maryland, College Park, MD, USA; 54 Key Laboratory of Songliao Aquatic Environment, Ministry of Education, Jilin Jianzhu University, Changchun, China

**Keywords:** Accretion, Accumulation, Biomass, Bulk density, Carbon cycling, Chambers, Core, Decomposition, Dissolved gas, Dissolved organic carbon, Eddy covariance, Greenhouse gas, Groundwater, Hydrology, Incubation, Lateral transport, Litter, Methane, Methods, Microbes, Models, Net primary productivity, Plants, Porewater, Radiometric dating, Remote sensing, Sediment, Soil organic carbon, Water, Vegetation

## Abstract

Wetlands cover a small portion of the world, but have disproportionate influence on global carbon (C) sequestration, carbon dioxide and methane emissions, and aquatic C fluxes. However, the underlying biogeochemical processes that affect wetland C pools and fluxes are complex and dynamic, making measurements of wetland C challenging. Over decades of research, many observational, experimental, and analytical approaches have been developed to understand and quantify pools and fluxes of wetland C. Sampling approaches range in their representation of wetland C from short to long timeframes and local to landscape spatial scales. This review summarizes common and cutting-edge methodological approaches for quantifying wetland C pools and fluxes. We first *define* each of the major C pools and fluxes and provide *rationale* for their importance to wetland C dynamics. For each approach, we clarify *what* component of wetland C is measured and its spatial and temporal representativeness and constraints. We describe practical considerations for each approach, such as *where* and *when* an approach is typically used, *who* can conduct the measurements (expertise, training requirements), and *how* approaches are conducted, including considerations on equipment complexity and costs. Finally, we review *key covariates* and *ancillary measurements* that enhance the interpretation of findings and facilitate model development. The protocols that we describe to measure soil, water, vegetation, and gases are also relevant for related disciplines such as ecology. Improved quality and consistency of data collection and reporting across studies will help reduce global uncertainties and develop management strategies to use wetlands as nature-based climate solutions.

## Introduction

**Fig. 1:**
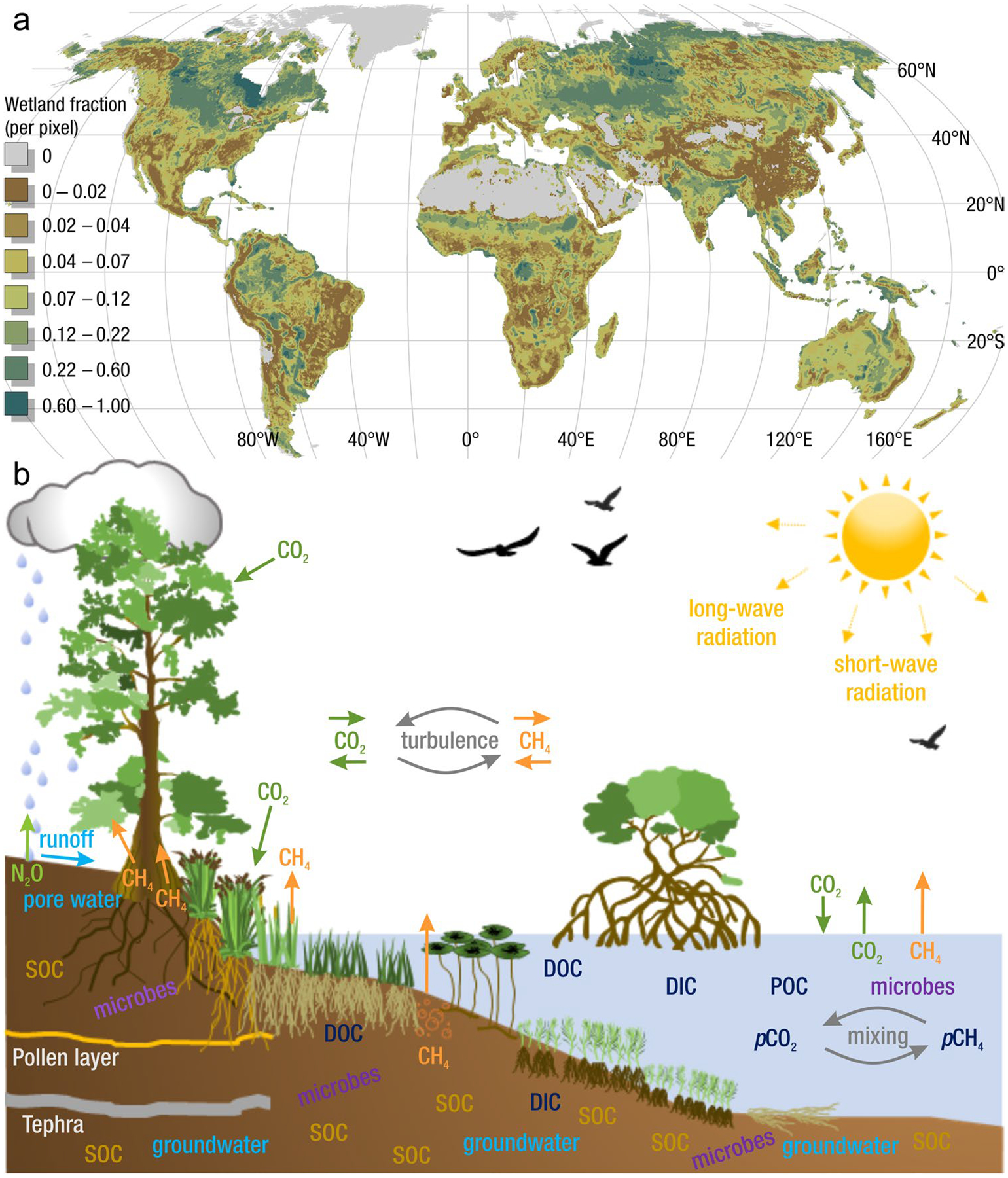
(**a**) Global distribution of wetland extent (fraction per 0.25 degree pixel [~ 25 km^2^ at the equator]) using Wetland Area Dataset for Methane Modeling (WAD2M). Map based on inundation data from Zhang et al. (2021c); Bansal et al. (2023); note the legend colors correspond with quantiles of wetland fraction to help visualize spatial variation across the globe (**b**) conceptual model of wetland carbon pools and fluxes [CH_4_, methane; CO_2_, carbon dioxide; DIC, dissolved inorganic carbon; DOC, dissolved organic carbon; N_2_O, nitrous oxide; *p*CH_4_, partial pressure of CH_4_ in water; *p*CO_2_, partial pressure of CO_2_ in water; POC, particulate organic carbon; SOC, soil organic carbon]

**Fig. 2:**
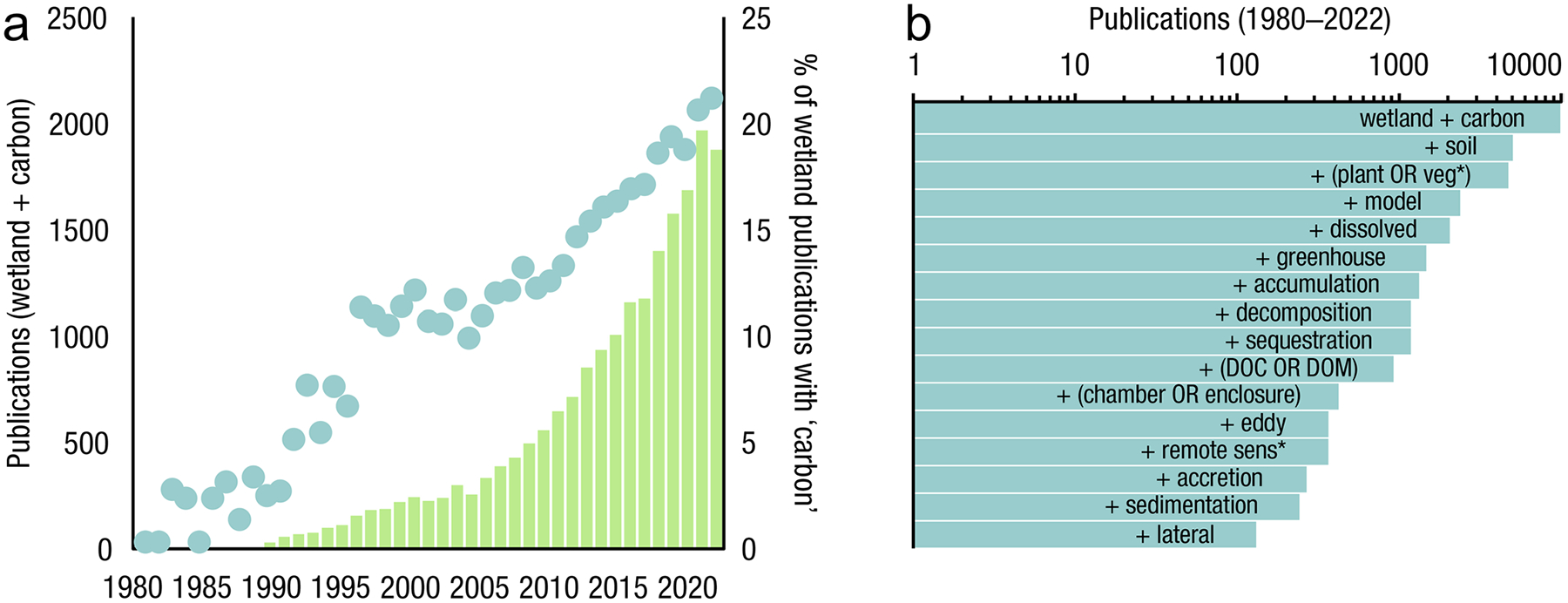
Wetland carbon publications from 1980 to 2022. (**a**) Annual number (bars) and percent (dots) of publications with keywords ‘wetland’ AND ‘carbon’; it should be noted that earlier studies did not focus on ‘carbon’ per se, but did focus on productivity and transfer of organic matter among trophic levels; (**b**) cumulative number of publi cations with keywords ‘wetland’ AND ‘carbon’ (top bar) AND additional keyword(s) (other bars). The ‘*’ symbol indicates any characters can follow. Both panels are based on searches conducted in the Web of Science database (www.webofknowledge.com) in April 2023

The global carbon (C) cycle involves exchange of C between terrestrial, atmospheric, and aquatic reservoirs. Wetlands, which occur at terrestrial-aquatic interfaces, cover only 3 to 8% of the land surface (Fig. 1a) (Lehner and Döll 2004), but they have a disproportionate effect on the global C cycle (Friedlingstein et al. 2020; Temmink et al. 2022). Wetlands, primarily those that are freshwater, account for > 20% of methane (CH_4_) emissions to the atmosphere (Kayranli et al. 2010; Saunois et al. 2020a), store up to half of terrestrial soil organic C (Mitsch and Gosselink 2015; Nichols and Peteet 2019), and supply large amounts of terrestrial C to the oceans (Stern et al. 2007; Köchy et al. 2015). Wetland C reservoirs (referred to as ‘pools’) and exchange rates (referred to as ‘fluxes’; Fig. 1b) are susceptible to rapid change due to human activities and land-use practices such as wetland drainage, restoration, construction, urbanization, and agriculture, as well as human accelerated climate-change feedbacks such as sea-level rise, shifting precipitation, and global warming (Zhang et al. 2017; Moomaw et al. 2018; Bansal et al. 2023). Fluet-Chouinard et al. (2023) estimated that approximately 20% of global wetlands have been lost through anthropogenic conversion since 1700, primarily for agriculture. Changes in wetland C pools and fluxes (Table 1) due to wetland management and global change can shift these ecosystems from atmospheric C sinks to sources, and vice versa. Therefore, understanding and quantifying wetland C pools and fluxes is essential to predict the global effects of wetlands on future climate and to evaluate the extent to which wetland management actions will mitigate or exacerbate climate change (Taillardat et al. 2020; Bansal et al. 2023; Bao et al. 2023; Zhang et al. 2023). As such, the relative number of scientific studies and syntheses of wetland C has dramatically increased in recent decades (Fig. 2; Table 2). However, the inherent complexities of C cycling in wetlands present measurement challenges for this burgeoning discipline. Whether conventional or cutting-edge, methodological approaches are inconsistently applied and are often study-specific. Even the language, terminology, and abbreviations are inconsistent within the discipline (Table 1). Consequently, comparisons and syntheses of data can be challenging, which is, in part, why regional and global estimates of wetland C pools and fluxes are poorly constrained (Melton et al. 2013) compared to other terrestrial ecosystem fluxes.

**Fig. 3:**
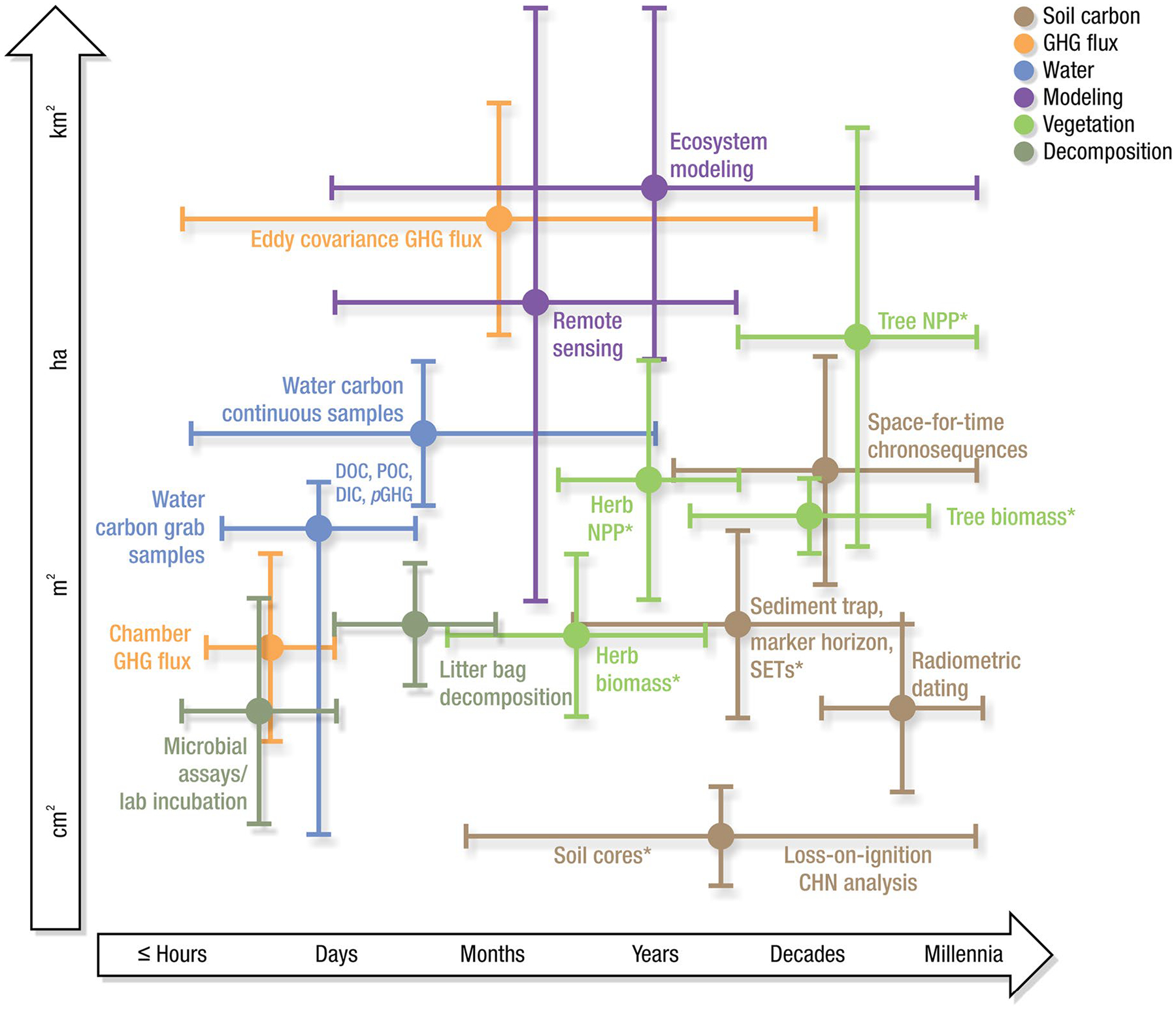
Wetlands have high spatial and temporal heterogeneity in their carbon (C) pools and fluxes. Methodological approaches shown here have different temporal (x-axis) and spatial (y-axis) scopes of inference to assess different carbon pools and fluxes (colors). *Vegetation (green) includes both harvest and allometric methods. *Soil C includes both soil carbon pools and accumulation rates. [CHN, carbon-hydrogen–nitrogen; DIC, dissolved inorganic carbon; DOC, dissolved organic carbon; Herb, herbaceous; NPP, net primary productivity; *p*GHG, partial pressure of dissolved greenhouse gases (GHGs) in water; POC, particulate organic carbon; SETs, surface elevation tables]

Measurements of C pools and fluxes from wetland systems are used to estimate how much C is being absorbed, transformed, stored, and released in soils, water, vegetation, and as gases. These estimates are used to establish baseline pools and fluxes and track changes over time, which are ultimately applied to inform policy decisions and management efforts (Howard et al. 2014; Villa and Bernal 2018). However, assessments of C pools and fluxes are difficult due to complex hydrological, biological, geological, and chemical (collectively referred to as ‘biogeochemical’) mechanisms that control C cycling in wetlands. To be expected, but not always appreciated, is that the underlying processes that affect C cycling in wetlands are highly heterogenous in space, changing across scales from millimeters to kilometers, and in time, changing from seconds to millennia (Fig. 3). Field measurements that are based on sampling approaches of upland systems are often insufficient to capture wetland spatiotemporal heterogeneities, leading to inaccurate estimates of wetland C pool sizes and flux rates. To meet the challenge of quantifying complex processes across diverse wetland environments, decades of interdisciplinary researchers have evolved many methodological approaches to measure C pools and fluxes in wetland settings over broad ranges of spatial and temporal scales (Fig. 3).

**Fig. 4:**
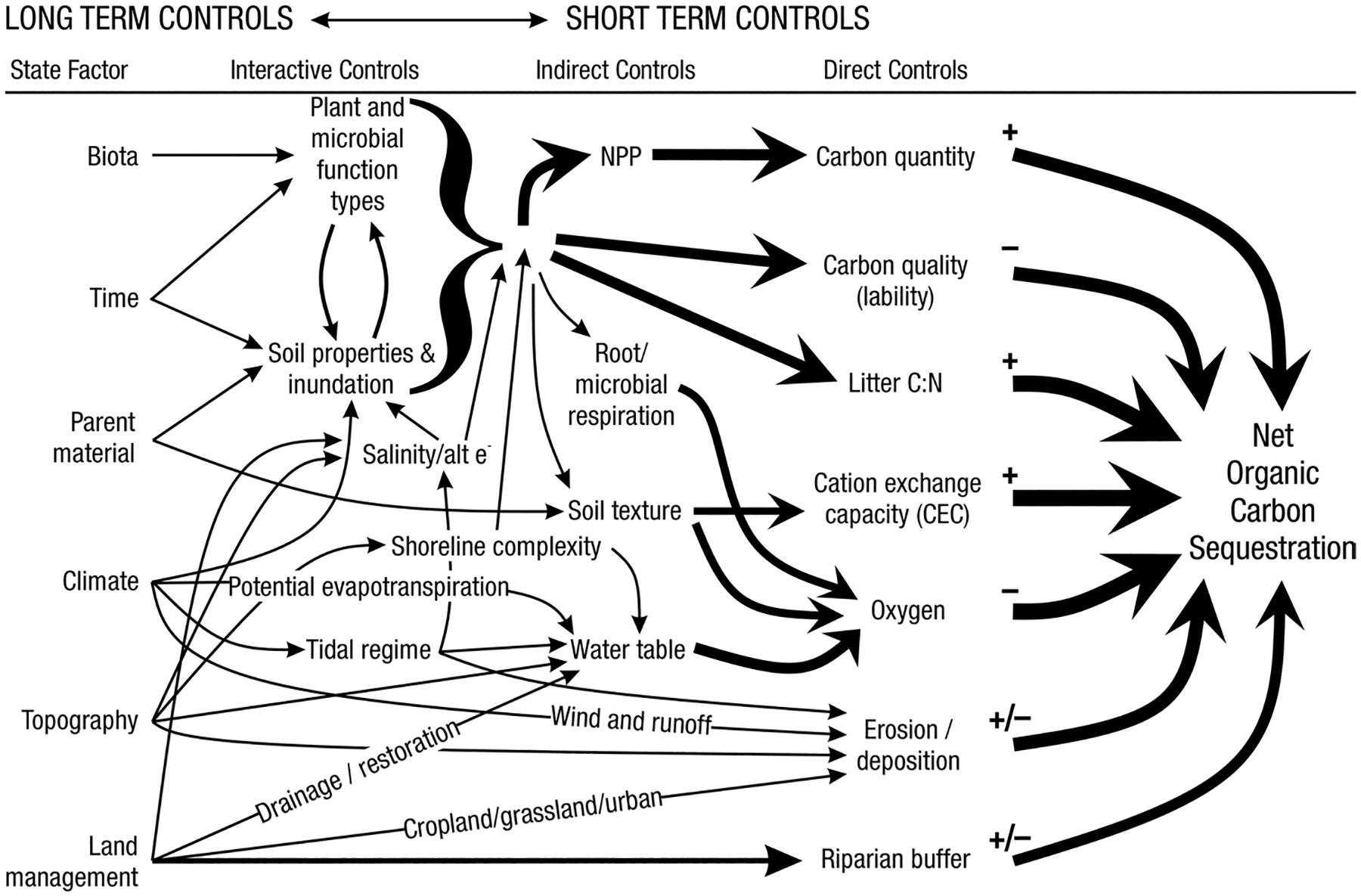
Long-term and short-term controls on net organic carbon sequestration in wetlands. The thickness of arrows indicates their relative strength of influence of controls. The + and – signs indicate the positive and negative relationships, respectively, between controls and net carbon sequestration rate. Image created by Irena Creed and Purbasha Mistry and was based on Chapin et al. (2011). [C:N, carbon to nitrogen ratio; NPP, net primary productivity]

A fundamental part of assessing wetland C pools and fluxes is understanding the abiotic and biotic environmental controls that govern C gains, losses, and transport. Environmental controls include soil and air temperature, precipitation, topography, geology, land cover and land use, hydrology, soil and water chemistry, weather conditions, vegetation, microbes, and many more (Fig. 4) (Bridgham et al. 2013). Quantifying relationships between C pools or fluxes and environmental variables (often referred to as ‘covariates’ or ‘predictors’) leads to valuable scientific understanding and practical applications. For example, microbial oxidation of soil C typically follows wetland drainage and results in carbon dioxide (CO_2_) emissions to the atmosphere. Thus, this effect from drainage is avoidable through management that prioritizes protection of wetland C by keeping soils saturated with water (Neubauer and Megonigal 2022; Zhu et al. 2022). The relationships between C and environmental covariates can also be used to estimate C pools and fluxes in locations not directly measured during wetland C assessments. For example, knowing the relationships between wetland greenhouse gas (GHG) fluxes and temperature can be used to predict changes in GHG emissions in response to global warming (Bridgham et al. 2013; Yvon-Durocher et al. 2014; Zhang et al. 2017; Bansal et al. 2023). Therefore, it is extremely important to measure environmental covariates during any sampling campaign focused on C pools and fluxes.

Each approach for measuring wetland C pools and fluxes has its own unique spatial and temporal scale of inference, applicability to wetland types and conditions, degree of random error and potential for systematic error, equipment costs, personnel training, and sampling timeframes. In this review, we describe conventional and cutting-edge approaches to measure major wetland C pools and fluxes. We provide practical considerations to highlight the strengths, limitations, conventions, and nuances of each approach. This ‘Practical Guide’ is not a replacement for standard method documentation or protocols. By providing information from diverse approaches all in one place, this guide will aid current and future scientists studying wetland C in: 1) making decisions about which method is appropriate or feasible for new research; 2) interpreting past C studies; 3) standardizing future measurements to facilitate comparisons and syntheses among studies; and 4) strengthening and advancing our understanding and models of wetland C cycling.

We hope, in writing this review, that new investigators of wetland C are less hindered by methodological challenges that wetlands supply in abundance; instead, new investigators have this article as a resource to aid their journey from study conception to data collection to communication. For more seasoned investigators, this article can assist with expanding their research breadth into new areas within this discipline, filling data gaps, and providing new perspectives. For new and seasoned investigators of wetland C, we hope this manuscript facilitates communication and collaborations among researchers with different specialties, creating synergies to accelerate the pace of science. Wetlands may play an important role in mitigating climate change. Results from past, present, and future studies will collectively guide changes in policy and land management to maximize climate and other co-benefits from wetlands.

## Overview of Wetland Carbon Pools and Fluxes

*Definitions:* Each component in the phrase ‘wetland C pools and fluxes’ has important implications, and establishing universal wetland C terminology is another challenge that scientists face (Table 1 for acronyms commonly used in the literature and in this review). Wetlands are areas where water covers the soil or is present either at or near the surface, generally with water less than a few meters deep, whether natural or artificial, permanent or temporary, with water that is static or flowing, fresh, brackish, or saline, all year or for varying periods of time during the year (Ramsar 1971; USEPA 2022). The Inter-governmental Panel on Climate Change (IPCC) and other institutions include an additional requirement of changes in chemical and biological conditions due to flooding to meet the definition of ‘wetland’. Regardless of definition, wetlands include a wide variety of organic- and mineral-soil types, including marshes, swamps, bogs, fens, peatlands, and mangroves, and the term ‘wetland’ is increasingly being applied to permanently submerged systems such as reefs, seagrasses, and shallow ponds. ‘Carbon’ is transferred in and out of wetlands and stored in multiple forms. C is most relevant to climate when emitted from wetlands as CO_2_ and CH_4_, or when organic C-based compounds (e.g., plant, algal, microbial remains) are buried in soils and stored for long periods of time. The term ‘pool’ refers to a snapshot quantity of C within a given area and given time that resides in soils, plants, or water. In this review, these three ‘pools’ collectively make up the wetland C ‘stock’ (Windham-Myers et al. 2019). Note, in the literature, the term ‘pool’ and ‘stock’ are used interchangeably, therefore it is important to check source-specific definitions. The various pools have different residence times depending on their biogeochemistry and environmental conditions. The term ‘flux’ is defined as a state of continuous change (or flow) of C within or across a given area per unit time. In studies on wetland C, ‘flux’ is generally used to describe a rate of accumulation, transformation, or transportation of C.

*Sampling design considerations:* When deciding on sampling designs and methodological approaches, it is important to consider the objectives of the study and how the data will be used, which generally fall into one of three categories: 1) to inventory wetland C pools and fluxes; 2) to investigate mechanistic processes; and/or 3) to build wetland C models. If investigators strategically plan representative sampling designs, then data generated from a study can often be used to satisfy multiple objectives (i.e., inventory, mechanisms, and models). In addition, investigators can a priori consider whether their effort would benefit from comparisons or contributions to existing ‘structured’ datasets (i.e., organized in an analysis-ready database) or ‘community-contributed’ datasets (i.e., derived from many individual contributors). Structured datasets are not designed for external contributions (e.g., U.S. Environmental Protection Agency (EPA) National Wetland Condition Assessment). Comparison with structured datasets helps in interpreting results from a given study, but typically requires comparable sampling protocols (e.g., depth of sampling, spatial-resolution, timeframe). Structured datasets, such as national or regional soil surveys, can also provide researchers with a ‘best guess’ of expected C pool sizes and flux rates. Community-contributed datasets (e.g., International Soil Carbon Network, SOils DAta Harmonization, AmeriFlux) are based upon contributions from individual studies, but often have specified sampling designs, metadata, or data-sharing requirements for new data to be eligible for contribution. Despite the extra challenge, community-contributed datasets are extremely important in developing estimates of wetland C pools and fluxes across wetland types and regions. Both structured and community-contributed datasets are used, for example, in national and global C accounting, developing IPCC emissions factors and scenarios, parameterizing process-based models, and more; therefore, it is often ‘worth the effort’ for investigators to contribute data.

Regardless of the objectives, wetland C sampling approaches need to assess and capture the spatial and temporal heterogeneity of C pools and fluxes within and among wetlands (Fig. 3). Determining the appropriate scale and representation of heterogeneity is a major challenge to understanding, measuring, monitoring, and modeling wetland C cycles. The scale of inference is also critical to linking pools and fluxes among other studies. If the aim is to inventory wetland C, the sampling design requires understanding of seasonal or conditional variability such as from shoulder season dynamics (e.g., spring thaw or autumn senescence), floodplain connectivity, and seasonal expansion and deposition of soil surfaces. To upscale wetland C data spatially, relevant spatial representation can be broad (wetland type) or narrow (land-form, species, etc.), depending on a study’s goals. For example, depressional wetlands have concentric rings of vegetation zones that each have unique C pools and fluxes, and thus a representative within-wetland sampling regime would collect data from each zone, with more samples from zones that cover more area. If the goal of the study is to provide information for models, then it becomes more important to capture the full inter- and intra-annual range of conditions. For example, if modeling GHG fluxes, measurements will ideally be conducted along a soil moisture gradient from wet to dry in both warm and cool temperatures to capture the range of conditions that affect production and emissions of GHGs. Ultimately, the sampling design will constrain the spatial and temporal scale of inference, which should always be acknowledged and explicitly defined.

If remotely sensed information or previously collected field data and associated location coordinates are available, then semivariograms can be used to characterize the magnitude and patterns of spatial heterogeneity (Cohen et al. 1990; Doughty et al. 2021), which can then help determine the minimum distance that plots need to be separated to minimize spatial autocorrelation among plots. Semivariograms can also be used to assess temporal autocorrelation among measurements, such as for GHG fluxes (Glukhova et al. 2022). New approaches (i.e., temporal Latin Hypercube) to optimize sampling protocols combine information from semivariograms and the probability distribution of the magnitude of GHG fluxes (Vargas and Le 2023).

*Safety:* There are potential hazards during sampling in wetlands, which can also affect sampling design. In addition to encountering wildlife such as pythons and alligators, many areas can have deep holes or soft sediments in which researchers can be trapped. Researchers should also consider protective gear, including snake proof-boots, waders, personal floatation devices, long-sleeve shirts, anti-mosquito head nets or jackets, and powder-free gloves. Note that this list is not comprehensive and that personal protective equipment should be selected to address the hazards specific to each study site making full use of local knowledge and experience. Also, it is important to be cognizant of other wetland research in the area and generally respectful of the ecosystem. For example, loud noises from hammering soil cores may disturb breeding waterfowl.

*Wetland carbon balance:* Individual components of wetland C pools and fluxes are relatively straightforward to define. However, a holistic definition of the wetland C balance that includes all components is less clear, in part due to different terms used in the scientific literature. Traditionally, net ecosystem production (NEP, Woodwell and Whittaker 1968) is the difference between gross primary productivity (GPP; C uptake via photosynthesis) and ecosystem respiration (ER; C release via the sum of autotrophic [R_A_] and heterotrophic respiration [R_H_]).


(1) $$\text{NEP}=\text{GPP}-\text{ER}=\text{GPP}-\left({\text{R}}_{\text{A}}+{\text{R}}_{\text{H}}\right)$$

Net ecosystem exchange (NEE, Baldocchi 2003) is the difference between ER and GPP, essentially the inverse of NEP.


(2) $${\text{NEE}}^{†}=\text{ER}-\text{GPP}$$

^†^It should be noted that the order of ER and GPP and the subsequent sign of NEE depends on discipline and is often study-specific.

Net primary productivity (NPP) is the plant-component of NEE and NEP, measured as the difference between GPP and R_A_.


(3) $$\text{NPP}=\text{GPP}-{\text{R}}_{\text{A}}$$

When considering a holistic C balance of a wetland ecosystem, there are C fluxes other than CO_2_ exchange through GPP and ER, including: 1) net vertical fluxes of trace gases such as carbon monoxide (F_CO_), methane (F_CH4_), and other volatile organic compounds (F_VOC_); 2) net lateral fluxes of dissolved inorganic carbon (F_DIC_), dissolved organic carbon (F_DOC_), and particulate organic carbon (F_POC_) from surface water and groundwater flow; and 3) other lateral fluxes such as soot emissions during fire (which could arguably be included in F_POC_). It should be noted that these fluxes are net values, indicating that they are a sum of both inputs and losses to the wetland (e.g., F_CH4_ is the net sum of CH_4_ production and oxidation). The term net ecosystem C balance (NECB; Chapin et al. 2006, 2009) is defined as the difference between all C fluxes and NEE.


(4) $$\text{NECB}=\left({\text{F}}_{\text{CO}}+{\text{F}}_{\text{CH}4}+{\text{F}}_{\text{DIC}}+{\text{F}}_{\text{DOC}}+{\text{F}}_{\text{VOC}}+{\text{F}}_{\text{POC}}\right)-\text{NEE}$$

To consider NECB over longer timescales, wetland soils are critically important as they accumulate large C pools under anoxic conditions. As wetland soils build up over time, they preserve chemical and biological information that can be extracted and used to model long-term NECB and wetland contributions to climate change and climate mitigation (Frolking and Roulet 2007; Frolking et al. 2010; Yu 2011).

When reporting NEP, NEE, NPP, or NECB, it is important to define measurement units and direction of flux clearly, and to include specific descriptors of spatial boundaries, timeframes, and individual C flux measurements before aggregating and extrapolating to other scales.

*Wetlands and climate change:* Wetlands, collectively, have a multifaceted effect on climate by affecting the atmospheric concentrations of CO_2_, CH_4_, and, to a lesser degree nitrous oxide (N_2_O). Knowledge of how these three GHGs differ in their atmospheric lifetimes and their ability to trap heat (i.e., absorb and reradiate infrared radiation) is needed to fully assess the climate footprint of wetlands, and thereby understand how wetlands can contribute to nature-based climate solutions (NbCS). We briefly summarize many of the current concepts and metrics to evaluate the effects of wetland GHG fluxes on climate and climate change, but it should be noted that these concepts/metrics are continually evolving, sometimes non-intuitive (i.e., confusing), and can be challenging to communicate to various audiences (e.g., scientists, policy makers, general public).

When considering the effect of wetlands on climate, it is important to distinguish the ‘radiative balance’ from the ‘radiative forcing’ of a wetland (Bridgham et al. 2006; Neubauer and Verhoeven 2019). The radiative balance (or budget) is a measure of how GHG inputs and outputs from a wetland affect Earth’s energy budget *at a point in time*. To calculate the balance, each of the GHGs are put into a common metric to account for their different warming effects, usually CO_2_-equivalent (CO_2-eq_) fluxes (more on this below). The term ‘balance’ does not necessarily imply that the inputs and output are ‘in-balance’ or ‘out-of-balance’, but instead can be thought of as the *balance* on a bill that reflects the difference between charges and payments. Radiative forcing is caused by a change in the radiative balance, or as stated by the IPCC, “an externally imposed perturbation in the radiative energy budget of the Earth’s climate system” (Ramaswamy et al. 2001), which can be positive (warming effect) or negative (cooling effect). For example, a wetland with CH_4_ emissions as CO_2-eq_ greater than CO_2_ uptake is not having a positive radiative forcing effect on the climate if those fluxes are constant over time. However, if CH_4_ or CO_2_ fluxes change due to altered environmental conditions (e.g., increased temperatures, nutrient pollution, saltwater intrusion) or management actions (e.g., drainage or restoration), a positive or negative radiative forcing can occur. N_2_O emissions or uptake from wetlands should also be taken into account due to the strong warming potential of N_2_O and relatively long atmospheric lifetime of 109 years (Forster et al. 2021). Eutrophication from nitrogen (N) in agricultural runoff can transition wetlands from sinks to sources of N_2_O, which can reduce their capacity to function as NbCS (e.g., Roughan et al. 2018).

The time horizon of interest plays a role in determining the positive or negative radiative forcing effects of wetland GHG fluxes. CH_4_ has a greater warming potential than CO_2_, but CH_4_ has an average atmospheric lifetime of only about 10 years compared to centuries to millennia for CO_2_ (Forster et al. 2021). The implication is that wetland CH_4_ emissions may initially cause warming, but eventually there will be a balance between wetland CH_4_ emissions and atmospheric CH_4_ removal, and therefore further wetland CH_4_ emissions do not contribute to warming (Frolking et al. 2006; Neubauer and Megonigal 2015, 2022). In contrast, the removal of CO_2_ and storage as soil organic carbon (SOC) by wetlands has a long-term persistent cooling effect. The switchover point when wetlands shift from positive to negative radiative forcing (i.e., from net warming to net cooling) may occur when the wetland is decades to centuries in age (known as radiative forcing switchover time), with the specific timing dependent on the ratio of CO_2_ sequestration to CH_4_ emissions (Frolking et al. 2006; Neubauer 2014). Many natural wetlands that have sequestered soil C over centuries to millennia are likely having net cumulative cooling effects over their lifetimes (Frolking et al. 2006; Neubauer and Megonigal 2015, 2022). When wetlands are disturbed (e.g., drained), the oxidation of sequestered SOC can cause the wetland to revert to having a lifetime warming effect. Following wetland restoration (e.g., rewetting), SOC is once again being sequestered, even while CH_4_ emission rates increase. The radiative forcing from CO_2_ (and sometimes N_2_O emissions) from unrestored wetlands far exceeds the temporary warming effect from CH_4_ emissions of restored wetlands (Neubauer and Verhoeven 2019; Nyberg et al. 2022).

Practically, to assess the relative radiative forcing of different wetland GHGs on a comparable basis and identify radiative forcing switchover times, measures of wetland GHG fluxes need to be normalized to CO_2-eq_ values. Below, we briefly describe some commonly used CO_2-eq_ metrics/models.

*Global Warming Potential and Sustained Global Warming Potential:* Reporting of relative radiative forcing of wetland GHGs in terms of CO_2-eq_ emissions is most commonly based on the metric Global Warming Potential (GWP), which is typically used for policy and reporting purposes (e.g., IPCC). The alternative Sustained Global Warming Potential (SGWP) metric is used more frequently within the wetland research community (Neubauer 2021). Conversion of non-CO_2_ fluxes to CO_2-eq_ follows the equation:

(5) $${\text{CO}}_{2-eq(i)}={\text{F}}_{\left(i\right)}\times {\text{SGWP}}_{\left(\text{H}\right)}\text{ or }{\text{GWP}}_{\left(\text{H}\right)}$$

where CO_2-eq*(i)*_ is the CO_2_-equivalent per mass of GHG *i* (mass CO_2-eq_ per area per time), F*_(i)_* is the measured gas flux rate (mass of gas per area per time), SGWP*_(H)_* or GWP*_(H)_* is the time specific normalization factor and H is the associated time horizon (e.g., 20, 100, 500 years). When considering multiple GHGs, the CO_2-eq_ of each gas can be calculated separately and then summed, paying close attention to the direction and sign of each individual flux (Neubauer 2021).

The 100-yr GWP (from Forster et al. 2021 [Table 7.15]) and SGWP (from Neubauer and Megonigal 2015 [Table 1]) for CH_4_ are 32 and 45, respectively. As an example, the 100-yr SGWP for CH_4_ (Neubauer and Megonigal 2015, 2019) can be interpreted as, “Over a 100-year period, the annual emission of one kilogram of CH_4_ to the atmosphere will have a radiative effect that is 45 times greater than that of the annual emissions of 1 kg of CO_2_.” The 100-yr GWP and SGWP for N_2_O are 263 and 270, respectively (Myhre et al. 2013; Neubauer and Megonigal 2015). The 20-yr GWP and SGWP for CH_4_ are 87 and 96, respectively, and for N_2_O are 260 and 250, respectively. While the choice of time horizon should be study-specific, the 100-year time horizon is most frequently used by the IPCC and in the scientific literature. It is important to note that SGWP and GWP values differ among sources and have changed over time as models of atmospheric chemical reactions and transport improve.

The key difference between the SGWP and GWP is that SGWP is based on continuous GHG fluxes, as occurs in nature, whereas GWP is based on a one-time ‘pulse’ of a GHG, which is rarely justified in wetland ecosystems (Neubauer and Megonigal 2015). Use of the standard GWP fails to accurately capture the effect of short-lived GHGs such as CH_4_ on climate (Lynch et al. 2020). Therefore, in wetlands, SGWP is more applicable when calculating CO_2-eq_ fluxes.

*GHG perturbation model:* Frolking et al. (2006) introduced a GHG perturbation model that relates the GHG-induced instantaneous radiative forcing to its concentration in the atmosphere at that time. This dynamic model considers the variations in atmospheric behavior of CO_2_, CH_4_, and N_2_O by considering the differences in their radiative efficiencies, atmospheric residence times, atmospheric removal mechanisms, and atmospheric CO_2_ feedbacks. The GHG perturbation model permits the use of a time series of GHG flux input rather than a singular time pulse input. As a result, unlike SGWP or GWP metrics that are time-integrated values, the GHG perturbation model calculates the radiative forcing of a GHG for each year, providing a description of the temporal behavior of wetland GHG fluxes (e.g., Neubauer and Verhoeven 2019) and enabling determination of the radiative forcing switchover time (Günther et al. 2020; Arias-Ortiz et al. 2021).

GWP*: The global change community has long recognized the limitations in the standard GWP approach to describe the climate effects of short-lived GHGs. The 6^th^ IPCC report suggests alternatives that more accurately reflect how changes in concentrations of short-lived GHGs such as CH_4_ result in changes in global temperatures (Forster et al. 2021). GWP* (spoken as ‘GWP star’), for example, is a metric that allows for the conversion of short-lived GHGs into CO_2-eq_ equivalents by accounting for 1) changing emissions of CH_4_; and 2) the time lag in temperature response due to previous CH_4_ emission increases (Cain et al. 2019; Lynch et al. 2020; Smith et al. 2021). Effectively, GWP* results in a larger effect of new CH_4_ emissions on temperature, but the effect decreases after a given amount of time (e.g., 20 years).

*Carbon accounting considerations:* Precise and accurate estimates of wetland C pools and fluxes are important to guide C management and policy, including applications such as national GHG inventories and C offset programs. Wetland C accounting mechanisms may vary in terms of the types of habitats and management activities they encompass, the C pools and fluxes that need to be accounted for, and the methodologies by which they are measured. Double-counting of C pools and fluxes is a risk that should be considered (Thornton et al. 2016). For example, allochthonous C from uplands that is eroded, transported, and stored in wetland sediment may inadvertently be counted in both upland and wetland C budgets (e.g., Valentine et al. 2023). Also, ‘wetland’ versus ‘lake’, ‘inland water’, ‘ponds’, and ‘coastal systems’ are not easily separated using remote sensing, resulting in CH_4_ budgets that overlap in global scale models (Thornton et al. 2016; Saunois et al. 2020a; Richardson et al. 2022).

Using wetlands for C offsets, or C credits, is a growing market, especially in coastal systems, which are often referred to as ‘Blue Carbon’ markets (Villa and Bernal 2018; Windham-Myers et al. 2019; Sapkota and White 2020). The basic concept is that the C removed from the atmosphere by wetland CO_2_ uptake or stored in wetland soils compensates for CO_2_ emissions released elsewhere. Currently (circa 2023), several mandatory and voluntary markets exist, although there is limited consistency among protocols to assess C offsets in wetlands. Challenges to developing a C offset protocol for wetlands include: 1) quantifying and tracking C offsets over time in CO_2-eq_ units, which requires robust and consistent methodological approaches; 2) interannual and regional variability in C offset prices ($/tonnes CO_2-eq_); and 3) emissions of CH_4_ from wetlands, which lessen net GHG reductions from the atmosphere and, therefore, is considered in C offsets. Saline coastal wetlands such as mangroves, salt marshes, and seagrass meadows are favorable for C offsets because of their high rates of organic C accumulation and low CH_4_ emissions (Windham-Myers et al. 2019). However, CO_2_ emissions produced by calcification may exceed C sequestration in systems with high calcium carbonate (CaCO_3_) levels (Howard et al. 2018; Van Dam et al. 2021).

*For this review:* We split wetland C into two general categories: 1) C pools; and 2) C fluxes. We define each pool or flux, discuss its relative importance in the overall understanding of wetland C cycles, explain the *rationale* for its measurement, and identify common and cutting-edge approaches to quantify it. We also convey *what* are the advantages and disadvantages of each sampling approach, its accepted spatial and temporal scales of inference, and current research gaps. We describe *where* and *when* an approach is typically used, and *who* can conduct the measurements (i.e., the expertise and training required). We provide information on *how* the approaches are conducted and list *key covariates* and *ancillary measurements* that are important to quantify pool and flux measurements. These key metadata can make data useful for other scientists who may be building models, upscaling, or conducting comparative analyses, all of which enhance interpretations and understanding of mechanisms driving C pool sizes and flux rates. Additionally, we provide brief overviews of microbial, modeling, and remote sensing techniques used in wetlands. Despite the high level of detail we provide, we strongly recommend that readers consult the source literature that we cite and beyond. We do not expect most readers to read this entire paper from beginning to end, but instead focus on specific C pools or fluxes of interest. However, please note that there may be considerable, relevant information in other sections that may be useful for understanding C pools of fluxes of interest (e.g., water salinity is important for understanding CH_4_ fluxes), which we point out as much as possible while also referencing relevant sections.

## Carbon Pools

### Carbon in Wetland Soils

#### Definitions and Units

*Definitions:* Organic and inorganic C accumulate in wetland soils and form a substantial C pool (Yu et al. 2010; Packalen et al. 2014; Nahlik and Fennessy 2016). Organic C content comes from biotic inputs (e.g., plant and animal debris) and inorganic C content comes from mineral or biogenic precipitates (e.g., CaCO_3_). Peatlands, mangroves, salt marshes, and seagrass meadows have the highest SOC pools of all ecosystems, with values as high as 2,000 Mg C ha^−1^ (Uhran et al. 2021; Temmink et al. 2022). Typically, only the organic fraction of C ‘counts’ towards C sequestration in soils, as inorganic C does not originate from photosynthesized CO_2_. However, there may be conditions in which inorganic C burial qualifies as C sequestration, such as when carbonates enter or precipitate in wetlands waters (Saderne et al. 2019; Wang et al. 2019b; Ouyang and Lee 2020). Inclusion of inorganic C may be especially important in some wetland types where the inorganic C fraction is relatively large (e.g., calcareous wetlands in Florida, USA or the Yucatan, Mexico), which may require differentiation from organic C for accounting purposes (Howard et al. 2014; Saderne et al. 2019; Windham-Myers et al. 2019).

The source of C in wetland soils can be further categorized as ‘autochthonous’ versus ‘allochthonous’ based on whether they are produced in situ or ex situ, respectively (Howard et al. 2014; Van de Broek et al. 2018; Windham-Myers et al. 2019). C pools in both organic- and mineral-soil wetlands are quantified through coordinated measurements of dry bulk densities, C contents, and soil depths (Ciais et al. 2014; Howard et al. 2014; Windham-Myers et al. 2019). *We emphasize the importance of measuring bulk density for interpreting C content in soils*. Organic C pools in surface soils can have varying residence times, depending on environmental controls on microbial activity and on organic matter quality, lability, and recalcitrance (Clymo 1984; Charman 2002). Organic C pools buried in deeper soils are often older with much longer residence times over centuries to millennia (Clymo 1984) compared to shallower soils that may only be years to decades old. Rates of C accumulation can be estimated using various approaches described in Section “Carbon Accumulation in Wetland Soil”.

The terms ‘soil’ and ‘sediment’ (and ‘peat’) are often used interchangeably in the scientific literature, which can lead to some confusion. There are numerous definitions for each term that vary depending on discipline. Overall, most definitions agree that sediment is not formed in place but is layers of “transported and deposited particles or aggregates derived from rocks, soil, or biological material” (SSSA 2021). In contrast, ‘soil’ is defined as vertically weathered mineral and organic material that has gone through biogeochemical transformations in place and over time, and therefore differs in physical, chemical, biological, and morphological properties from which it was derived (van Es 2017; SSSA 2021). Depending on the depositional environment in wetlands, much of the belowground material is a mixture of sediment and soils, and therefore binary definitions are not appropriate and can cause misunderstanding when describing methodological approaches and results (see Kristensen and Rabenhorst 2015 for extensive discussion). In this review, both ‘soil’ and ‘sediment’ are used synonymously.

‘Peat’ generally refers to soils that have a relatively high fraction of organic matter (e.g., > 65%). Peat is the partially decayed remains of the plants that were formerly living at the surface, so it is distinguished from sediment in that the material accumulates in situ in waterlogged conditions, rather than being deposited from above. However, the term ‘peat’ is also often used within coastal systems, such as salt marshes or mangroves, which can include organic C from allochthonous sources (e.g., DeLaune et al. 1981; Kida and Fujitake 2020). Wetland scientists that use hydric soil indicators and soil scientists typically divide organic soil materials into three types based on the amount of decomposition (minimal, intermediate, or advanced): ‘peat’ in the Oi horizon made up primarily of fibric material; ‘mucky peat’ in the Oe horizon with hemic material; and ‘muck’ in the Oa horizon with highly decomposed, unidentifiable sapric materials. The degree of decomposition is typically correlated with SOC content, with peat having the most SOC by weight and muck having the least. The term ‘mucky’ can also be used to describe the fluidity of soil, with mineral clays and silts that flow under pressure/weight, but have relatively little SOC.

*Units:* Wetland soils are typically classified into ‘mineral’ versus ‘organic’ depending on the SOC content (e.g., > 12% cutoff for organic soils, U.S. Soil Taxonomy). Organic, inorganic, or total soil C can be reported in several different metrics (Table 3), including as a proportion (%) of dry mass, mass per unit area, and mass per volume (density), and should have a specified depth and spatial extent. Areal extents (e.g., km^2^) are used for scaling soil C to a given region or system of wetlands, often in teragrams (Tg, 1 × 10^25^ g) or petagrams (Pg, 1 × 10^15^ g) (Yu et al. 2010; Howard et al. 2014; Packalen et al. 2014).

**Rationale:** The largest and most stable pool of C in wetlands is typically located in the soils (e.g., Temmink et al. 2022). Small changes in the soil C pool size may translate into significant changes of C fluxes to or from the atmosphere and adjacent water bodies. Information from studies on SOC pools is collectively used in national and international C accounting reports. The amount of C stored in soils also represents the amount of C that can be lost to the atmosphere as CO_2_ if wetland systems are degraded through drainage or through natural disturbances such as fires in peatlands and tropical cyclones in seagrass meadows. Studies of replenishing lost SOC via uptake from the atmosphere are also important for assessing the role of wetland restoration and construction (e.g., Osland et al. 2012; Bansal et al. 2022) for offsetting increasing atmospheric CO_2_ concentrations.

#### Soil Collection

*What:* Each wetland has its own unique characteristics including soil type, vegetation community, and hydrology; thus, soil collection approaches should be selected accordingly. Extraction of wetland soils is generally conducted using soil corers (Table 4), but blocks of soils as monoliths can also be collected with many of the same basic considerations of protocols. A complete soil profile down through the O and A horizons to the depth of the soil parent material, referred to as the C horizon, establishes information about a complete SOC pool. Note that despite the common term, ‘parent material’, wetlands soils are primarily accretionary – thus not derived from underlying rock layers. All coring methods involve extraction of soil cores while maintaining stratigraphic integrity (i.e., keeping the different layers from moving or mixing) to provide information on C pools along vertical profiles. Volumetric integrity should also be considered to obtain accurate bulk density measurements by accounting for compression during core extraction or using a corer that does not compress the soil (Smeaton et al. 2020, Table 4). The coring approach provides information on the pool of soil C to the depth of the core and by depth increment if desired, but does not provide information on the rate of C accumulation without additional analyses described in Section “Carbon Accumulation in Wetland Soil”.

There are many existing soil databases that incorporate data from a variety of collaborative research networks (e.g., Harden et al. 2018). It is important to understand how data were collected, as some databases are not calibrated/validated for wetlands. Since it may be difficult to know the depth to the C horizon prior to coring, these soil databases can provide a ‘first guess’ as to the soil type and organic layer thickness, the hydrology, and other ancillary information to help guide sample collection protocols. Examples of *global* databases include: the Coastal Carbon Atlas (CCN 2021); the International Soil Carbon Network (ISCN 2021); the International Soil Reference and Information Centre’s World Soil Information (ISRIC 2021); and the Global Map of Black Soils (FAO 2022). Examples of r*egional* and *national* databases include: the European Soil Data Center (ESDAC 2021); the National Wetland Condition Assessment (USEPA 2021); the Soils Data Harmonization (SoDaH 2021; Wieder et al. 2021); the USDA-NRCS National Cooperative Soil Survey (NCSS) Soil Characterization Database (USDA 2023c); the Soil Survey Geographic Database (SSURGO; USDA 2021), which is also available as a geodatabase (gSSURGO; USDA 2023a); the Soil and Landscape Grid of Australia (TERN 2021); Vegetated Coastal Ecosystems (VCE) of Australia (Serrano et al. 2019); and the Canadian Soil Information Service (CanSIS, Agriculture and Agri-Food Canada 2000).

*Where:* The location at which soil cores are collected is dependent on the reporting objectives and the scale of the study. Wetland characteristics (e.g., soils, vegetation, hydrology) are typically heterogeneous with respect to landscape position. If the objective is to determine the soil C pool within a site (i.e., a specific wetland), the boundaries of wetland zones (often based on hydrology or vegetation) can be determined using Global Positioning Systems (GPS) and/or visual assessment methods so representative cores may be collected. Organic C pools can be highly variable across an individual wetland owing to underlying geomorphic context (van Ardenne et al. 2018). For example, some areas within a wetland may be lower in elevation (e.g., hollows) and subject to greater accumulation of organic matter than higher elevations (e.g., tussocks, hummocks) (Webster et al. 2011). Understanding how a wetland developed can help identify spatial heterogeneity and guide sampling (Redfield 1965, 1972; Arndt and Richardson 1988; Schwimmer and Pizzuto 2000). Also, the underlying depositional basins of many wetlands are not flat, and there is often a deepest point – a depocenter – where C pools and depths may be relatively high (van Ardenne et al. 2018). Spatial gradients in water sources within a wetland can lead to variations in water, nutrient, particulate organic C (POC), and mineral sediment loading, influencing soil C densities (Webster et al. 2014). Mobilization and recirculation of sediments due to various forces, most notably wind and aquatic animals, will often cause sediments to focus in these depocenters, but can also move sediments from the open water area to get trapped in vegetated edge (Zarrinabadi et al. 2023). Thus, multiple cores within a wetland (e.g., three or more) are needed to characterize soil C pools. The actual number of cores required will likely increase with wetland size and habitat heterogeneity. A degree of randomization with regard to sample collection helps avoid bias and capture true variation (Howard et al. 2014). Randomization can be applied across an entire site, or within strata (e.g., zones) that represent homogeneous conditions. The latter, referred to as a ‘stratified random design’ is a common design since it ensures sampling in representative strata while maintaining randomization. Semivariograms (Glukhova et al. 2022) used in combination with probability distribution and geostatistics can help optimize sampling designs when a priori information is available (Fennessy et al. 1994a; Vargas and Le 2023).

If the objective is to compare soil C pools across watershed, state, regional, or national scales, soil cores are typically collected to the same target depth at all wetlands. Existing information about C densities and C pool depths may help inform the number of soil cores to collect within a wetland versus across the entire study area being characterized (e.g., watershed, state, region). Young et al. (2018) provide a case study in Australia on optimal sampling design for estimating soil C pools in coastal wetlands.

Wetland coring locations may be selected using a spatially balanced stratified statistical design that considers the diversity and density of wetlands or wetland characteristics across the scale of the study population. A Generalized Random Tessellation Stratified (GRTS) survey design (Stevens and Olsen 1999, 2004) is an example of one method that selects sampling locations that are spatially balanced, meaning that locations with more wetlands have more sample points (‘grts’ function in *spsurvey* R package [Dumelle et al. 2023]). Spatially balanced designs facilitate upscaling of C pools from multiple wetlands in a region. Strata used in the design, which may include specific variables or gradients (e.g., U.S. Natural Resources Conservation Service soil map units, soil wetness, vegetation communities), can be selected based upon the reporting goals. Olsen et al. (2012) provide a summary of sampling designs over large spatial scales, and Olsen et al. (2019) detail a spatially balanced stratified survey design that was used to sample wetlands on a national scale in the United States.

*When:* In the absence of major disturbance events, changes in soil C pools are often slow, hence it may take several years or longer before a significant change can be measured with any degree of confidence. Given the relatively long time frame of C accumulation processes in soils, the time of year for soil C sampling is less sensitive to seasonality, and it is thus usually constrained more by logistics and environmental factors, such as water depth and prevailing weather. For example, it is generally easiest to collect soil cores in non-tidal wetlands at a time of year when water levels are low. Soils are typically more pliable and conducive to coring while they are wetted, but wet soils may be more easily compacted than dry soils. When wetland soils or overlying water are fully or partially frozen, specialized coring techniques are required and conditions can be hazardous. There are, however, instances when sampling frozen soils is preferred; for example, permafrost peatlands are best cored in the winter when they are frozen so that the ‘active layer’ or seasonally unfrozen soil can be recovered (note that surrounding thawed bogs and fens can be cored more easily in summer or autumn). Coring when soils or the overlaying water column are frozen is also useful to avoid compaction of loose, fluid soils. If the study objective includes microbial analyses, sampling frozen soils can help preserve the Deoxyribonucleic Acid (DNA) and Ribonucleic Acid (RNA) sample (Dalcin Martins et al. 2017), albeit microbial communities and activity may change seasonally.

In marine and tidal wetlands, hydrology and water level are important considerations when planning to collect cores. Sampling of soils in shallow marine or tidal wetlands is often done during low tide. If snorkeling or diving are required, sampling is recommended during slack tides to avoid strong tidal currents. Sampling may also be constrained by disturbance regimes (e.g., flooding events, droughts, fires), especially if there are major disturbance events that disrupt the structure and function of the ecosystem.

*Who:* In most wetlands and with many of the coring devices, field technicians can collect soil cores with minimal training, although it can be physically demanding to extract cores and transport soils. More experienced personnel are needed for choosing the location and the timing of the core collection. Furthermore, experience is needed to select the appropriate corer (Table 4) with special consideration of soil characteristics and potential obstructions and impenetrable layers present. In some cases, personnel qualified to operate heavy machinery may be needed, including trailer-mounted or gasoline-powered corers for collecting deep samples in hard soils, including clays. Wetlands that have surface water > 1.5 m deep, such as shallow subtidal wetlands, may require the use of Self-Contained Underwater Breathing Apparatus (SCUBA)-trained personnel to collect cores. If soil cores are collected by soil horizon (as opposed to discrete depth intervals), a soil scientist that specializes in wetland morphology or pedology may be needed to identify horizons and their boundaries, or to train field technicians with some basic guidance to delineate the horizon boundaries and characterize soils within a soil profile (e.g., Schoeneberger et al. 2012; USEPA 2021). The technical ability to discern soil horizons also may contribute to the selection of an approach.

*How:* Soil C pools are measured using intact soil cores, which ensure preservation of the stratigraphic integrity and allow for both C concentration and bulk density to be measured volumetrically on the same sample. In some cases, bulk samples, or monoliths are collected (see below). Some soils are simply not readily cored because of difficulties in maintaining volumetric integrity (e.g., uncompacted peat), determining the soil surface (e.g., thin-mat floating wetlands, unconsolidated, fluid sediment surface layer), and penetrating solid substrates (e.g., tropical soils with thick root surface layers or rocky soils). Specialized corers, such as the Hargis corer tipped with a razor blade, have been developed to overcome compaction in peat soils (e.g., Hargis and Twilley 1994; van Asselen and Roosendaal 2009).

*Soil sampling approaches:* Two primary approaches to sampling soil cores for estimating C pools in wetlands are: 1) sampling by soil horizon; and 2) sampling by one or more depth intervals. The best approach will depend on the soil type, wetland type, environmental conditions (e.g., water depth, presence of ice or woody debris), sampling objectives, type of analyses to be performed, and time and other logistical constraints.

*Sampling by soil horizon:* Soil horizons are physically and chemically distinct soil layers across a depth range that develop as a result of soil forming processes including additions, losses, transformations, and translocations of physical structures, organic compounds, chemical oxidation states, and elemental composition within wetland soils (Simonson 1959; Buol et al. 2011). Each soil horizon will differ in its color, texture, structure, and other soil properties – and thus there may be differences in soil C content and bulk density. A large portion of SOC may be in the O horizon. Classification of soil taxonomy can provide additional information on soil properties, but names differ by country (e.g., Soil Classification Working Group 1998; Soil Survey Staff 1999; Isbell 2016; Land Information System 2021; IUSS Working Group WRB 2022).

Collecting soil cores by soil horizon can include the excavation of a soil pit so that boundaries among horizons – often indicated by changes in soil color, texture, presence of redoximorphic features, structure, and consistency – may be delineated. For these pits, de-watering of soils, such as in seasonally drained bottomland hardwood wetlands, facilitates horizon determination. Depending on the soil conditions and water table level, there are a variety of techniques that may be used to excavate a soil pit. USEPA (2021) provides specific protocol for varying soil conditions and water table levels. Some submerged soils can even be sampled using this approach by building a soil coffer dam and using a hand pump (USEPA 2021). Where de-watering is not an option, cores can be extracted first and then classified by horizon, although the opportunity to collect additional soil information for each horizon may be lost.

Collecting soil cores from horizons within soil pits is not the most common approach in wetlands, nor is it the easiest; but one advantage of this approach is that additional data may be more easily collected about each horizon, such as the soil chemical characteristics and oxidation states. These additional data can give insight into the hydrology, past and present land uses, soil condition, and ecosystem processes associated with C pool quantities and fluctuations with depth (see below *Key Covariates and Ancillary Measurements* for examples of useful information that may be gathered from soil horizons). Identifying horizons can also keep laboratory samples to a minimum to capture variability in soil profiles – otherwise more increments may be needed to identify transitions. A potential disadvantage of this approach is that, because the depth of soil horizons varies from site to site, choosing the correct depth to core may require a series of pilot cores to estimate horizon depths. Horizon samples can also be aggregated to a fixed depth increment (e.g., 0–50, 50–100 cm, etc.) to compare to other studies while still maintaining the additional information on horizons. Collecting bulk density samples from narrow horizons may also be challenging.

*Sampling by incremental or fixed depths:* In wetlands, intact cores are most commonly collected directly from the soil surface (i.e., not using a soil pit) to a specific fixed depth or opportune depth (see below *Soil coring depth*) either as one single core or as a series of incremental cores representing differing depth intervals. Deciding whether to collect a single core or a series of cores for C pool assessments is largely dependent on the length of the core, the soil and site conditions, and the type of soil coring device used. Single soil cores greater than 1 m in length, which are often needed for paleo-reconstruction studies, may require specialized long-barrel coring cylinders and/or powered coring devices, such as a vibrating corer. If the soil is particularly dense or dry at the time of sampling, or the researchers are limited to a non-ideal coring device, it may be easiest to collect several incremental cores from the same hole, representing different depth intervals until the final depth is reached. For example, in a study to quantify wetland SOC concentrations in Northeast China, Ren et al. (2020) collected soils representing depths from 0 to 30 cm, 30 to 60 cm, and 60 to 100 cm from the soil surface. Sampling, and therefore the quantification of soil C pools, is typically specified to a certain depth that is comparable to other published studies and locations. It is not recommended to extrapolate soil C or bulk density to depths below those actually sampled, as those data may not be accurate. However, interpolation within a core using a statistically valid design is reasonable when all depth increments cannot be sampled (e.g., Fourqurean et al. 2012; Kauffman and Donato 2012).

*Soil coring depth:* Both the soil horizon sampling approach and the depth interval sampling approach require a set goal for how deep (from the soil surface) to collect soil based on the study objectives, recognizing that the goal is not always achievable due to site conditions (e.g., deep water), obstructions (e.g., coarse wood, large boulder/rocks), or impenetrable layers (e.g., clay pan, bedrock, cemented layer, ice). Wetland soils often have organic-rich soils that range from a few centimeters to several meters in depth (Donato et al. 2011; Mitsch and Gosselink 2015); therefore, in C accounting studies, it is important to sample depths that include all or a representative fraction of their organic soil thickness. Measuring C to a standard sampling depth, such as 1 m as suggested for coastal C (Howard et al. 2014), allows for comparisons across wetlands, but may miss deeper soil C. Nahlik and Fennessy (2016) found that, when wetlands were sampled to 120 cm in both inland and tidal wetlands across the United States, 65% of the organic C pool was stored in soils from 30 to 120 cm depth. This result emphasizes that sampling deeper soils (e.g., to > 1 m) may be necessary to accurately quantify the soil C pool.

In wetlands with a thick organic layer upwards of 3 to 8 m in depth (i.e., peat), knowing the peat depth is useful in deciding the appropriate coring depth. Prior to coring, the organic peat depth can be assessed with a simple push probe approach using a cone-tipped metal rod attached to a pressure gage or electronic recording device (e.g., Penetrologger, Eijkelkamp Agrisearch Equipment) to measure penetration resistance expressed in pascals per unit cone area (Pa cm^−2^) (Hsu et al. 2009; Parsekian et al. 2012); organic soil depth also can be estimated or verified ‘by feel’ when an experienced technician inserts a probe. Larger cone sizes provide more accurate information but are more difficult to insert into hard soils. While the push probe method is relatively fast, it also has relatively high error compared to other methods (Parry et al. 2014). Information to characterize soil layers can also be detected using non-invasive electromagnetic methods (Comas et al. 2015; Boaga et al. 2020), ground penetrating radar (Zajícová and Chuman 2019), and induced polarization (Slater and Reeve 2002). Ground penetrating radar can be affected by high conductivity environments (e.g., saline estuarine environments; Neal 2004). Small diameter (e.g., 2 cm) Oakfield augers and medium diameter (3.75 cm) JMC Backsaver probes, are often used to preliminarily assess the soil profile, and can be combined with results from core analysis to provide better estimates of total C pools. Studies aimed at chronological reconstructions, such as sea-level reconstructions, may require cores that measure several meters; the total core length will depend on the time scale of interest and the estimated sedimentation rate. For example, sedimentation rates of 1 cm yr^−1^ imply that the upper 100 cm of soils or sediments have the potential to encompass the last 100 years of accumulation, save for any shallow compaction that may have occurred over the 100 years.

**Fig. 5:**
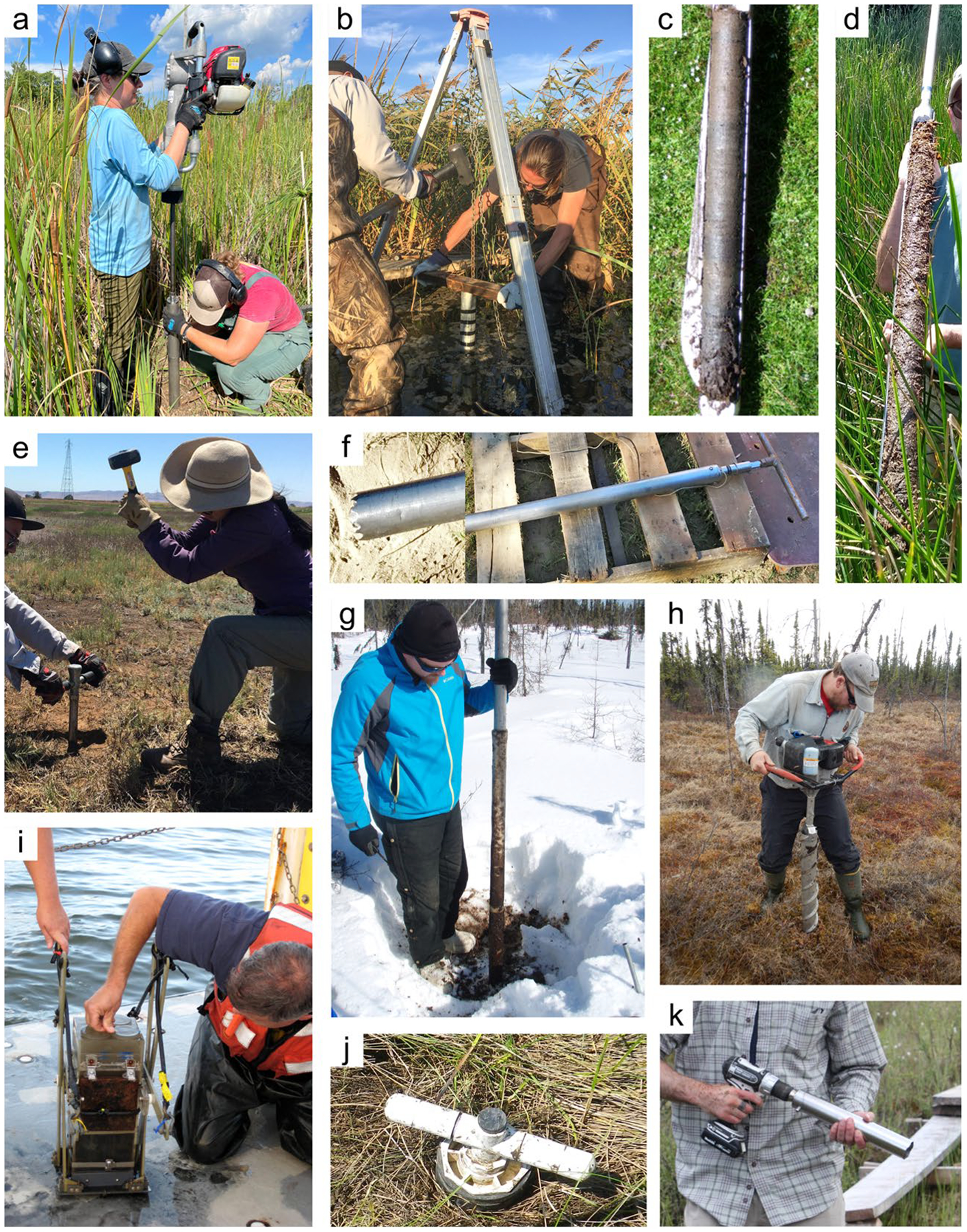
Examples of soil coring and devices, including: (**a**) barrel corer with a gas powered post driver; (**b**) piston corer with tripod (for core extraction); (**c**) Russian (Macauley) peat corer; (**d**, **e**) gouge auger; (**f**) Livingstone piston corer modified with serrated barrel for coring through fibrous sediment; (**g**) core freezer (also referred to as the ‘frozen finger’); (**h**) Snow, Ice, and Permafrost Research corer; (**i**) box-style corer; (**j**) soil coring tube inserted into the soil with core cap and handle above the soil surface; (**k**) hand drill corer. Images with permission from Cathleen Sampselle (a), Ariane Arias-Ortiz (b, e), Carl Trettin (c), Satya Kent (d), Donald Rosenberry (f), Dong Yoon Lee (i), Camille Stagg (j), and Mark Waldrop (g, h, k). See additional images of corers in various figures presented in Osborne and DeLaune (2013)

*Soil coring devices:* Regardless of the sampling approach, one of a spectrum of recommended coring devices (Table 4) can be employed to ensure preservation of stratigraphic and volumetric integrity (and ideally avoidance of soil compaction), allowing for both C concentration and bulk density to be measured on the same core (Smeaton et al. 2020). Corers are highly variable in size and shape but have several similar characteristics. Most coring devices have a cylindrical portion (i.e., the ‘barrel’) used to retrieve the sample, which can be split vertically or used with a liner to facilitate the preservation and removal of the intact core. Many corers have a handle used to aid in inserting, twisting, and extracting the corer from the soil (Fig. 5c). One method to avoid damaging the core is to presplit the coring barrel, tightly clamp it back together using duct clamps, collect the sample, and finally, carefully loosen the clamps and open the coring barrel to reveal the intact core.

Although there are multiple commercially available coring devices, researchers often build their own equipment (e.g., using beveled PVC pipes) to deal with the peculiarities of their research interests and sites. The bulk density of the soil is a key factor in selecting or designing the coring device (Section “Soil Analysis - Bulk Density, Loss-on-Ignition, Elemental Analysis”). Sampling soils with low bulk density without introducing disturbance requires the use of a coring device that is designed to avoid soil compaction and allow the correct determination of dry bulk density. This can be tricky if cores have to cut through woody debris and lignified roots common to forested wetlands. Multiple attempts are often required for a single core; sharpening of the bottom edge of the corer is recommended in such instances. Other types of coring devices are specialized for collecting frozen soils (e.g., the Modified Hoffer Probe) or unconsolidated soils (e.g., the Cryogenic Coring Device). Additionally, various types of shovels can be essential for extracting cores and samples.

*Soil core extraction:* Specific protocols by which intact soil cores are collected are conditional upon the coring device used, although there are some general principles for intact soil coring that can be followed. The primary goal of intact soil coring is to recover a complete, undisturbed sample, typically including the sediment/water interface, that is volumetrically and stratigraphically representative of the soil while in situ. The ideal characteristics of an undisturbed soil sample are: 1) no disturbance of structure; 2) no change in water content or void ratio (i.e., no compaction); and 3) no change in constituent or chemical composition. Specifically, compaction of the soils is ideally, carefully avoided and, should compaction occur, the bulk density measurements will need to be corrected according to calculations (see Morton and White 1997). Care should be taken in the field to collect a complete core, with no voids in or at the bottom of the core, which may require digging the core out to support the bottom of the core sample as it is removed from the surrounding matrix. Finally, generally try to avoid changes in anoxic conditions or temperatures that could cause oxidation-related changes to C content and/or ancillary data used to understand C transformation (e.g., iron [Fe] or sulfur [S] speciation), although maintaining redox is not required for soil C estimates. In some situations where coring is not feasible, an intact soil block can be shoveled out, placed on a tarp, and then cored. Sampling ports can also be predrilled in the core walls to facilitate subsampling of discrete depth intervals; sampling ports are covered with tape prior to sampling and then extracted by inserting a tube (e.g., cut-off syringe) into each port.

Crucial information to collect during the process of sampling includes the total length of the collected core, the depth of surface compaction (i.e., the difference in surface elevation of the inserted core just before extraction versus the true soil surface), the bore depth (i.e., depth to which the coring device was inserted into the soil), and the diameter of the coring cylinder. In some instances, cores can be sectioned into required increments in the field after collection. When sectioning or extruding cores in the field, it is recommended to photograph the intact cores when possible. Pre-labeling sample bags and using waterproof labels facilitates data collection in inclement weather. Steps for collecting soil cores in wetlands for pool assessments have been extensively described previously (Schoeneberger et al. 2012; Osborne and DeLaune 2013; Howard et al. 2014; Soil Science Division Staff 2017; Weintraub 2017; USEPA 2021); ASTM D4823-95 (2019) provides guidance for core sampling in submerged, unconsolidated sediments. Methods of collection of soil cores for analysis of radionuclides and trace elements requires additional care to avoid contamination have been reviewed elsewhere (IAEA 2003; Brenner and Kenney 2013).

*Soil block extraction:* Some soils can be collected as an undisturbed block with standard size and depth dimensions. This soil extraction technique preserves an intact block of soil on which redoximorphic features and other soil properties can be described (Johnson et al. 2003). To extract a soil block, a column is first delineated that is slightly larger than the sample container (such as a three-sided polycarbonate container with sealed joints with a thin, sliding metal floor). A trench the diameter of the sample container (to allow for the insertion of the metal floor) is then carefully excavated around all sides of the intact soil column to the depth of the sample container. While lowering the sample container over the intact soil column, excess soil is gently removed using a knife so that the soil column fits exactly (without compression) into the sample container. Once the sample container is placed, the metal floor can be slid onto the bottom of the sample container from one side of the trench, effectively slicing the block of soil and containing it. The soil block (inside its container) can then be lifted and transported to a laboratory.

*Soil transport and storage:* Transportation of soil samples from the place of collection to a field or permanent laboratory requires care and planning to ensure that the soils are not disturbed. When transporting intact soil cores, it is important to consider the consistency of the soils (i.e., soil type, clay and sand content, water content, organic content), their length, and whether it is best to keep them in a horizontal or vertical position to prevent mixing or down washing of unconsolidated material and to maintain the stratigraphic integrity of the sample. For example, cores consisting of unconsolidated organic or fluid mineral soils, or collected underwater (thus filled with water to the top of the core tube) are often transported in a vertical position after collection (IAEA 2003; Howard et al. 2014). Cushioning the core with foam to absorb vibration can help reduce vertical compaction during transportation. Additionally, it is important to have a water- and air-tight seal on the storage container to avoid evaporative losses, spillage, or addition of water from melted ice within a transport cool-box. For horizontal transport of more solid (less fluid) soils using a corer without a liner tube, cores can be placed in PVC pipes (same core diameter) that are cut longitudinally, and wrapped and sealed accordingly (e.g., plastic wrap, aluminum foil, taped, additional PVC) to prevent disturbance. If the core does not fill the PVC pipe, foam or other material can be used to fill any gaps. It is good practice to label the core with an arrow pointing towards the top of the core. To minimize potential loss of organic compounds through microbial degradation, drying, oxidation, or volatilization, intact cores or soil blocks are generally kept refrigerated or on wet ice while they are returned to the laboratory. In some situations, it may be preferable to maintain ambient temperature conditions. Upon arrival to the laboratory, storage temperature of the cores is usually based on the soil temperature at the time of collection, with unfrozen soils stored in a refrigerator at 4 °C and frozen soils stored in a freezer at − 4 °C or colder until they can be analyzed. Preparation of the soil cores for analysis is based on the study objectives and the suite of analyses to be conducted (Section “Soil Analysis - Bulk Density, Loss-on-Ignition, Elemental Analysis”). For sandy or fluid soils, freezing the cores enables easy sectioning either by depth or splitting along the length.

*Key Covariates and Ancillary Measurements:* During soil collection, there are several ancillary variables that can assist with interpretation of the soil C data, infer processes of C pool formation, and facilitate upscaling. Additionally, ancillary measurements are advantageous to align with existing national and international databases containing wetland soil C data and to provide perspective when data are included in synthesis activities and comparative analyses (Table 2). Some ancillary measurements such as soil pH, conductivity, and redox are ideally measured in the field to avoid artificial changes associated with soil extraction and transport, but they are often measured under laboratory conditions for logistical reasons (Section “Soil Analysis - Bulk Density, Loss-on-Ignition, Elemental Analysis”).

*Site characteristics:* Important site characteristics include latitude and longitude recorded to a minimum of 4 decimal degrees (for merging with 30-m pixel remotely sensed information) and absolute elevation relative to sea level, ideally using differential Real-Time Kinematic (RTK) GPS procedures for sub-centimeter accuracy. It is also important to have a thorough description of the site, including wetland classification or hydrogeomorphic type (e.g., Cowardin et al. 1979; Brinson 1993), soil descriptions and taxonomy, vegetation (species composition and distribution), hydrology (depth to water table at time of sampling or continuous water level record if possible), meteorological conditions and prevailing weather (e.g., air temperature, precipitation), and land use history information. Interviewing land managers can be useful to collect information on wetland-specific management practices.

*Soil core characteristics:* Immediately upon excavating a soil pit or collecting a soil core, it is ideal to assess properties that can change upon exposure to air, such as the presence of hydrogen sulfide odor (i.e., rotten egg smell). Color can change as well: blue-gray colors indicate anaerobic conditions that allow microbial reduction of Fe from ferric (Fe^3+^) to ferrous (Fe^2+^); upon exposure to air Fe^2+^ will oxidize back to Fe^3+^ and form red patches, often seen along roots where radial oxygen loss occurred (Vasilas et al. 2018). Identifying horizon boundaries, horizon names (i.e., taxonomy), and the depths of the upper and lower boundaries of each horizon can provide information about the soil profile. Soil morphologic properties, including soil texture, presence of rock fragments, presence of roots, soil matrix color (hue, value, chroma; e.g., Munsell soil color charts), redoximorphic features (soft masses, nodules/concretions, pore linings/ped faces), presence of masked sand grains, and organic features (organic bodies, stripped matrix, organic infillings), can help identify the presence of hydric soils (Soil Survey Staff 1999; Schoeneberger et al. 2012; USEPA 2021; Soil Survey Staff 2022). Descriptions of the thickness of the organic horizons, bulk density, and soil texture provide necessary information to distinguish wetlands dominated by organic soils from those dominated by mineral soils (e.g., Soil Survey Staff 1999; Nahlik and Fennessy 2016). To facilitate visual descriptions, cores can be collected using solid or open barrel corers lined with transparent tubes or sampler liners, or split barrel corers. In some instances, it is advantageous to split the core lengthwise for examination. Photographs with a measuring tape of the soil’s vertical profile and of each soil core is recommended.

*Organic soil type:* Determination of fiber content and the degree of decomposition (humification) using rapid techniques are useful field metrics for distinguishing different types of organic soils: peat, mucky peat, and muck (Malterer et al. 1992). The von Post humification method is a rapid, albeit subjective, technique that involves squeezing a handful of soils and observing the volume and turbidity of expressed water, the proportion of soils extruded between fingers, and the fiber content and quality of soil (Stanek and Silc 1977).

### Soil Analysis – Bulk Density, Loss-on-Ignition, Elemental Analysis

*What:* After soils have been transported to the laboratory and stored appropriately for C pool assessment (Section “Soil Collection”), they need to be prepared for analysis of organic and inorganic C content, dry bulk density, and other soil properties.

Soil cores can be analyzed as a whole or by depth increments (e.g., slices). Analysis of the whole soil core is sufficient for studies where the average concentration of C (or other attributes) in a defined horizon or over the depth of the core is the metric of interest. Analysis of the whole soil core may reduce sample analysis costs and analysis time. In contrast, a core may be sliced (either in the laboratory or in the field) and analyzed by depth increments of a standard thickness. Intact soil cores sliced into depth increments are important to assess the vertical change in the physical, chemical, and/or biological properties of soils, which serve as proxy records of environmental or ecological change, including changes in C accumulation rates (Section “Carbon Accumulation in Wetland Soil”). Low sedimentation rates (~ 1–2 mm yr^−1^) may require relatively fine sectioning intervals; if high sedimentation rates are expected (several mm yr^−1^), cores can be sliced at thicker intervals. The soil increments can be individually analyzed for C and summed post-processing. Sectioning the soil core into discrete intervals can also be based on soil horizons (e.g., O, A, E, B, C) or special features such as plow, ash, or outwash layers (Stolt and Hardy 2022), whereby a ‘before/after’ approach allows C analysis on two segments that occurred prior to and following a known event. A disadvantage of this approach is that comparing standardized depth increments across sites is difficult without further analysis to match soil horizons with the appropriate depth increment.

There are two common methods to assess the fraction of organic and inorganic C in soils: mass Loss-on-Ignition (LOI) and direct analysis with a Carbon Hydrogen Nitrogen (CHN) elemental analyzer.

*LOI:* LOI is a simple and low-cost metric to determine the percent of organic and inorganic C in a soil (Dean 1974; Heiri et al. 2001; Hoogsteen et al. 2015). Organic matter is burned off in an oven, which leaves behind the inorganic mass as ash (Heiri et al. 2001; Smith 2003; Abella and Zimmer 2007; Wright et al. 2008; Hoogsteen et al. 2015). Both temperature and ignition time will affect LOI results (see below *How*). Therefore, soil type and study goals (e.g., precision and accuracy) should be considered when determining appropriate ignition times and temperatures (e.g., Heiri et al. 2001; Smith 2003; Santisteban et al. 2004; Abella and Zimmer 2007; Hoogsteen et al. 2015). The ash sample can then be burned further at higher temperatures (e.g., 800–950 °C) to determine the inorganic C content, which can also vary by temperature and ignition time. Muffle furnaces are relatively common and easy to maintain compared to CHN analyzers.

The LOI method provides information on the Soil Organic Matter (SOM) content of a sample. A conversion factor is needed to convert SOM to SOC. A widely used general conversion factor of 0.58 (SOM × 0.58 = SOC), which is known as the van Bemmelen factor, has historically been used and assumes that C makes up 58% of SOM. However, the proportion of C in SOM, and thus the conversion factor, will vary across soils and 0.58 is considered too high for many soils (Pribyl 2010). For example, Braun et al. (2020) found a conversion factor of 0.53 for freshwater coastal wetlands on Lake Michigan (USA) and Baustian et al. (2017) found 0.47 across all Louisiana (USA) soils. Ouyang and Lee (2020) found a similar conversion factor (0.52) for salt marsh soils, but a significantly lower one for mangroves (0.21). Craft et al. (1991) found conversion factors ranging from 0.4 to 0.6, depending on SOM content and age of the soils, in North Carolina (USA) salt marshes. Fourqurean et al. (2012) found a conversion factor of 0.43 for global seagrass sediments. Therefore, it is highly recommended to determine local SOM:SOC ratios on a subset of samples that are measured using both LOI and CHN analyzers. The correlation and associated graph of SOM × SOC can be provided in publication as supplementary material. See Bhatti and Bauer (2002), Konen et al. (2002), and Wright et al. (2008) for discussions on unique regressions and conversion factors for LOI versus organic C in specific soil types.

*Elemental analyzers:* Elemental analyzers of CHN are a reliable method to directly measure total soil C (i.e., organic and inorganic), nitrogen (N), and hydrogen (H) through combustion of soils at ~ 925 °C. For a more in-depth description of elemental analysis of C and N see Nelson and Sommers (1996) and Chatterjee et al. (2009). Unlike the LOI method, elemental analyzers directly measure C content, with reliable results and the ability to analyze many samples in a short time following sample preparation. These instruments also simultaneously measure sample N content, as well as other elements. Maintenance time requirements for CHN analyzers involve several hours to a day for packing and installing new reduction and combustion tubes. Elemental analyzers can be relatively expensive, but there are abundant research and contract laboratories that analyze soil samples.

*Where:* LOI analysis requires indoor bench space for a drying oven and vented muffle furnace. In addition to a drying oven for sample preparation, CHN elemental analyzers require hoods to ventilate fumes and indoor bench space. Bench space is also needed for a microbalance for weighing samples in micrograms, although some new elemental analyzers have integrated balances. There will also need to be a low traffic, well-ventilated, dedicated space in the laboratory for the gas tanks (compressed air, oxygen gas [O_2_], helium [He]/argon [Ar]) that are quality assured (e.g., contracted gas specifications with suppliers), that are appropriately stored and inspected (e.g., retaining straps or chains, leak inspection).

*When:* After collection from the field, samples for soil C pool analysis do not need to be processed or analyzed immediately. Field samples can be stored in a refrigerator or freezer until ready for slicing, drying, grinding, and sieving.

*Who:* Some training is required for conducting the LOI procedure and properly weighing samples. Safety concerns include the muffle furnace, which can reach temperatures ~ 1000 °C, and laboratory personnel should be familiar with proper operation of the muffle furnace. Moderate training is required to set up the CHN elemental analyzer and become familiar with procedures and preparation of the samples (e.g., a practiced, steady hand to fill small soil cups). Safety concerns include the acetanilide standards and chemicals for reduction and combustion processes (such as copper oxide, silver vanadate, silver tungstate or magnesium oxide, etc.) and laboratory personnel should be familiar with the chemical safety data sheets.

*How:* Prior to analysis for determination of C content, soil cores can be non-destructively described, segmented, dried, weighed (wet and dry weight), ground, and sieved. Soil mass water content and bulk density also can be determined.

**Fig. 6:**
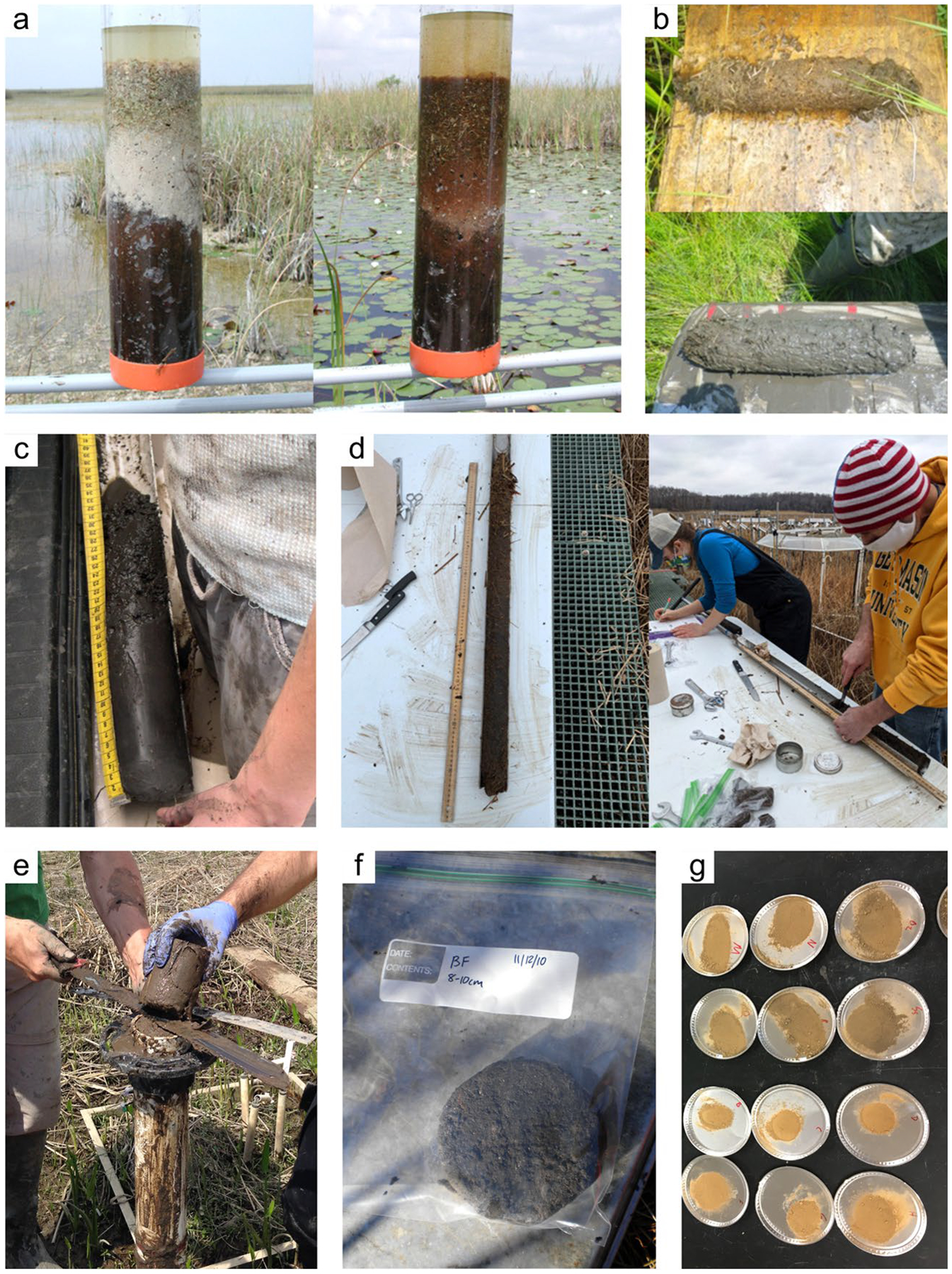
Examples of collection, processing, and analysis of soil cores, including: (**a**) visual comparison of open barrel cores from nutrient unenriched (left) and enriched (right) sites in the Everglades, Florida, USA; (**b**) visual comparison of cores from organic-soil (top) and mineral-soil (bottom) marshes in Louisiana, USA; (**c**) measuring depth of core extracted in Palo Verde, Costa Rica using an open barrel corer that was split vertically post-collection to extract the core; (**d**) measuring (left) and processing (right) a core collected using a gouge auger at the Salt Marsh Accretion Response to Warming eXperiment (SMARTX) in Maryland, USA; (**e**) extruding and slicing core increments from a polyvinyl chloride (PVC) open barrel corer in an intertidal freshwater wetland, Louisiana, USA; (**f**) storing core increment for transport; (**g**) soil samples following determination of carbon content using Loss-on-Ignition (LOI). Images with permission from Sue Newman (a, modified from Reddy and DeLaune 2008), Camille Stagg (b), Amanda Nahlik (c), Satya Kent (d, left), Genevieve Noyce (d, right), Dong Yoon Lee (e), Siobhan Fennessy (f), and Olivia Johnson (g)

*Core description:* Prior to extruding, slicing, or storing, general visual descriptions, which are also conducted when a core is collected in the field, are recorded including length, color, and any gaps, spaces, or incomplete core sections (Fig. 6a–d) (Kroetsch et al. 2011). Descriptions and locations by depth are also recorded for any undecomposed macroscopic plant or animal remains such as wood, leaves, seeds, or shells. If not already completed, core photographs are taken (with a tape measure for reference in the photo) for archiving. If the core is refrigerated or frozen, the length of the core can be remeasured once removed from storage and compared to the length that was recorded in the field to assess whether shrinkage due to water loss has occurred.

*Extruding and Slicing:* There are two general techniques for slicing (i.e., segmenting) sediment cores that can either be conducted in the field or laboratory: 1) slicing whole-core horizontal segments based on depth intervals; and 2) vertically slicing the core in half lengthwise prior to horizontal slicing (Fig. 6d). The first method uses a sediment extruder when necessary (e.g., for solid body corers or cores collected in liner tubes) to push discrete, horizontal intervals of the soil from the corer or core liner (Fig. 6e). The extruded material is sectioned into depth increments of known volume and stored in labelled containers (more details in Glew et al. 2001). The second method requires a lengthwise splitting of the corer cylinder to expose the core, which can then be vertically sliced, photographed, scanned (e.g., for magnetic resonance or texture), described, and sectioned in desired horizontal thickness intervals. Slicing can be accomplished using a variety of tools such as thin wire (e.g., piano or guitar string) and knives. The sectioning thickness (horizontal distance) will depend on the time resolution desired and the amount of material needed for analysis (De Vleeschouwer et al. 2010; Rogers et al. 2015). Also of consideration is the need to store a substantial part of each slice as an archive.

*Drying:* The temperature and time to dry samples can have large effect on sample mass and SOC content. Air or low temperature oven drying (~ 25–40 °C) of soil samples may take several weeks during which microbial mineralization of SOC can occur (Dettmann et al. 2021). Also, low temperature drying may not remove all soil water, which will bias mass determination and underestimate SOC. Warmer temperature can release volatile organic compounds, lowering SOC. Generally, oven drying at 70 to 105 °C for 48 to 72 h is sufficient to reach a constant weight (Gardner 1986), but the exact temperature and heat time to reach a constant weight should be empirically confirmed as some soils may require more time at higher temperatures. If dried samples are stored at room temperature, the samples should be re-dried in an oven prior to analysis.

In addition to air or oven drying, freeze drying has proven effective for soils. Freeze drying has been shown to maintain physical properties such as porosity (Thompson et al. 1985), and it is less destructive to biological indicators (e.g., testate amoebae, diatoms, macrofossils, DNA) or other soil components. Soils can be freeze dried prior to determination of soil properties such as C content and bulk density (e.g., Connor et al. 2001; Hung and Chmura 2006; Fu et al. 2021; Soong et al. 2021). Freeze drying is a preferred option when organic materials are a large part of the sediment, since oven drying can cause considerable hardening, making the task of homogenization more difficult. Freeze drying is also useful for shipping and storing samples (Weißbecker et al. 2017; Clasen et al. 2020). Despite the advantages of freeze drying, oven drying is more common because of its ease and lower cost.

*Grinding and Sieving:* Once soils are air or freeze dried, and prior to C analysis, they are ground and sieved (typically using a 2-mm sieve) to create a homogenous sample. Soil samples can either be crushed and/or ground by hand with a mortar and pestle or with a variety of mechanized soil grinders or pulverizers. Once samples are ground, sieving removes remaining coarse materials such as rocks and large plant material. Volumes of the removed materials are assessed by displacement of water in a graduated cylinder, and then used to correct soil volumes. Mass of the removed materials should also be determined. For some studies, larger objects such as rocks, roots, coarse wood, or shells may be removed prior to grinding and sieving, depending on study objectives. Inclusion or exclusion of these materials will ultimately depend on the question the researcher is interested in. If the goal is to assess the soil C pool, cores should exclude the leaf layer of the soil profile, as well as roots and other dead organic debris from the surface. There is generally no need to remove roots in the soil profile as they are part of the C pool and can remain preserved and identifiable for thousands of years (Drexler et al. 2014). However, large pieces of wood or vegetative debris that are not representative of the soil profile (i.e., random branch from upland) should be removed from deeper soil samples, but may be preserved for other analyses (e.g., radiometric dating). Removals should be done with care to avoid compressing or altering soil samples.

*Dry bulk density:* Dry bulk density is the ratio of the dry mass of a soil sample to the bulk in situ volume of the sample (Blake and Hartge 1986). Determination of dry bulk density (g cm^−3^) is critical for studies of soil C because it is used along with the sample depth increment to convert C content (%, g C g^−1^ soil) to C mass per unit area (g C m^−2^). The relationship between dry bulk density and C content is broadly inverse and consistent across a range of C values, particularly for organic compared to mineral soils (Morris et al. 2016), which can be used as a point of comparison.

Wetlands in the United States have dry soil bulk densities generally ranging from 0.5 to 1.5 g cm^−3^ (Mobilian and Craft 2022), although soil bulk densities can be as low as 0.04 g cm^−3^ for some organic soils (Mitsch and Gosselink 2015). While individual studies have reported bulk densities higher than 1.5 g cm^−3^ for mineral soils (e.g., Hossain et al. 2015a; Nahlik and Fennessy 2016), it is unlikely to encounter or to accurately sample soils with bulk densities greater than 1.85 g cm^−3^ (for reference, sedimentary rocks typically have a bulk density around 2.0 g cm^−3^).

Blake and Hartge (1986) and Al-Shammary et al. (2018) offer reviews of methods for determining soil bulk density, with the most common technique being the core method where the volume of the sample is known. Once core segments have been dried and ground, dry bulk density is calculated as the total dry weight divided by the sample volume. The mass and volume of the sample are adjusted for any coarse materials removed manually or during sieving. As a note, grinding and sieving are not always required prior to bulk density measurements depending on study objectives. When presenting bulk density, it is important to describe how samples were collected and processed (e.g., drying temperature and duration), as well as to document protocols for processing coarse materials.

*Soil mass water content:* If soil water content (mass basis) is desired, it can be readily measured by gravimetric analysis on wet soils before drying (Gardner 1986). A wet soil sample is taken to the laboratory, checked for water loss during transport, and ideally immediately weighed. Water is then removed through oven drying or freeze drying. Soil mass water content is calculated as the difference between the wet and dry weight (air or low temperature drying) divided by the dry weight of the sample (Gardner 1986):

(6) $$\theta_{dw}=\left(\text{weight of wet soil }-\text{ weight of dry soil}\right)/\text{weight of dry soil}$$

where *θdw* is soil mass water content.

*LOI:* For LOI, dried and ground samples are oven dried or freeze dried to a constant weight at ~ 105 °C, which typically takes ~ 12 to 24 h depending on sample size (Fig. 6g). This procedure is warm enough to remove hygroscopic water bound to soil particle surfaces, but not risk removal of mineral associated water (Sun et al. 2009). Once the samples are oven dried and weighed they can be combusted. It is recommended to analyze replicates for each sample (e.g., 3 replicates) to account for potential within-sample heterogeneity. There is a wide variation when it comes to recommended combustion temperatures and ignition times to burn off organic matter, with temperatures ranging between 300 to 850 °C and ignition times ranging anywhere from 0.5 to 28 h (Heiri et al. 2001; Smith 2003; Abella and Zimmer 2007; Wright et al. 2008; Hoogsteen et al. 2015). Generally, higher temperatures for longer periods will burn a larger fraction (up to all) of SOM, but risks combustion of inorganic C and removal of structural water in clay (Sun et al. 2009). Lower temperatures risk incomplete combustion of SOM. The typical combustion time is 1 to 4 h, but may need to be longer depending on soil type and sample size (see Heiri et al. (2001) for a detailed discussion). For example, Heiri et al. (2001) suggest that 1 to 2 h may not be sufficient for samples high in organic matter. Hoogsteen et al. (2015) suggest a combustion temperature of at least 550 °C for 3 h to ensure complete combustion of organic matter. After cooling to room temperature, samples are weighed again and % LOI of organic matter can be calculated:

(7) $${\text{LOI}}_{550}=\left(\left({\text{DW}}_{105}-{\text{DW}}_{550}\right)/{\text{DW}}_{105}\right)\times 100$$

LOI_550_ is the percent SOM, DW_105_ is the dry weight of the sample after drying at 105 °C, and DW_550_ is the weight after combustion at 550 °C (see Dean (1974), Nelson and Sommers (1996), Schulte and Hopkins (1996), Heiri et al. (2001) for more detail on methods).

To calculate inorganic C in soils (e.g., for dolomite-rich soils or those high in CaCO_3_ [limestone]), samples are combusted again at ~ 950 °C, for 1 to 2 h and weighed again after cooling to room temperature (Rabenhorst 1988). % LOI is then calculated as:

(8) $${\text{LOI}}_{950}=\left(\left({\text{DW}}_{550}-{\text{DW}}_{950}\right)/{\text{DW}}_{105}\right)\times 100$$

where DW_950_ is the dry weight of the sample after combustion at 950 °C. LOI_950_ is directly proportional to % inorganic C (i.e., does not need a conversion factor like LOI_550_; Heiri et al. 2001). If results are to be reported as % CaCO_3_, LOI_950_ is multiplied by 1.36 (ratio of CaCO_3_:CO_2_; Bengtsson and Enell 1986).

*CHN elemental analyzer:* If determination of just the organic fraction of C is desired, then soils that contain carbonates need to be pretreated with acids to remove inorganic C (Nelson and Sommers 1996). Carbonates can be removed using acids such as phosphoric (H_3_PO_4_), hydrochloric (HCl), and sulfurous (H_2_SO_3_) (Phillips et al. 2011). Phillips et al. (2011) suggests sequential additions of acid to determine how much will be needed to fully remove all of the carbonates present. Acid fumigation (i.e., exposure of soil samples to HCl fumes contained inside a vessel) using 12 M HCl can also be used to remove carbonates (Harris et al. 2001; Ramnarine et al. 2011; Dhillon et al. 2015) and has been suggested as a more accurate and precise method (Komada et al. 2008; Dhillon et al. 2015). The fumigation times have been found to range anywhere from 6 to 56 h depending on how much carbonate is present as removal rates range from 0.08 to 0.12 mg hr^−1^ (Harris et al. 2001; Ramnarine et al. 2011). Further, when measuring total C in soils that are high in carbonates (> 30% CaCO_3_), sample size is an important factor as larger sample sizes (> 10 mg) may result in incomplete combustion of carbonates. See Phillips et al. (2011) for more detail on selecting sample sizes for carbonate-rich soils.

After inorganic C removal, homogenized samples are packed into tin capsules, generally 2 to < 100 mg depending on amount of C in the soils, and then combusted in the CHN analyzer. The combustion method determines C, N, and H content in soils by converting the C to CO_2_, N to nitrogen gas (N_2_), and H to water by combustion at ~ 925 °C in a purified O_2_ environment. After combustion, the gases are pushed through a reduction tube (~ 640 °C) using a carrier gas, typically He or Ar depending on the instrument, to reduce nitrogen oxides (NO_x_) to N_2_. The final gas products, CO_2_, N_2_, and water, then separate. Thermal conductivity detection or infrared spectroscopy are used to detect N (measured as N_2_), C, and H content (in that order) (Jimenez and Ladha 1993).

*Key Covariates and Ancillary Measurements:* There are several other parameters that are ideally determined when measuring C content via LOI or elemental analyzers. Information on wetland hydrology (Section “Carbon in Wetland Waters”) and vegetation (Section “Carbon in Wetland Vegetation”) are important drivers of soil C pools and fluxes. Other important parameters include soil mass water content, dry bulk density, and mineral fraction or clay content. Dry bulk density is particularly important for quantifying soil C pools.

*Equivalent Soil Mass:* When comparing C pools among study treatments where there are artificial differences in soil bulk density, such as from compaction and tillage (decompaction), the use of an Equivalent Soil Mass procedure (see Ellert and Bettany 1995; Wendt and Hauser 2013) may be appropriate to avoid erroneous under- or over-estimating SOC conclusions. Rather than comparing soil parameters (e.g., SOC) based on fixed depth increments (e.g., 0–15 cm), the Equivalent Soil Mass procedure allows for comparisons among cores based on a common soil mass (reference mass) to avoid artifacts associated with artificially induced changes to bulk density. Equivalent Soil Mass calculations typically require soils from deeper in the soil profile than the target depth increment of interest, and ideally cores are split into multiple fixed increments. Additionally, study sites should encompass study-specific reference conditions (e.g., undisturbed control) to support determination of a reference soil mass, which is required for the Equivalent Soil Mass method. Fowler et al. (2023) demonstrates a simple soil mass correction and provides more details on Equivalent Soil Mass calculations for assessing soil C pools, and see von Haden et al. (2020) for an R script for calculating Equivalent Soil Mass. Ellert and Bettany (1995), Wendt and Hauser (2013), and Fowler et al. (2023) provide visual diagrams representing the technique, which are helpful for understanding the concept. For examples, Badiou et al. (2011) and Tangen and Bansal (2020) accounted for differences in land-use history (e.g., cropland versus grassland) among wetlands using the Equivalent Soil Mass methodology.

*Volumetric water content:* Volumetric water content (soil water content on a volume basis) can be determined once soil mass water content and dry bulk density are known. To calculate volumetric water content, dry bulk density is divided by density of water and then multiplied by the dry weight basis water content. See Blake and Hartge (1986) for detailed methods on volumetric water analysis.

(9) $$\theta_{vb}=\left(p_{b}/p_{w}\right)\theta_{dw}$$

where *θ_vb_* is soil water content (volumetric basis), *p_b_* is dry bulk density, *p_w_* is density of water, and *θ_dw_* refers to soil mass water content from equation 6. Volumetric water content for surface soils (e.g., upper 10 cm) also can be determined in the field using hand-held probes (Section “Chamber Measurements”).

*Texture (sand, silt, and clay) and particle size distribution:* The relative proportions of particles can have important effects on SOC pools and turnover times. Sandy, large-grained soils have lower water holding capacity. Clays can create organomineral complexes that stabilize SOC (Heister et al. 2012).

When using the LOI method for estimating the % C content, information on the clay content may be important because SOM can be overestimated as a result of structural water loss or dehydroxylation (i.e., release of hydroxide [OH^−^]) in clays when combusting at high temperatures. Structural water loss can occur at temperatures as low as 300 °C and losses increase as temperature increases, and dehydroxylation of clays like kaolinite occurs at temperatures > 400 °C (Frost and Vassallo 1996). Therefore, a semi-quantitative correction factor can be applied to soils containing significant fractions of clay (Sun et al. 2009; Hoogsteen et al. 2015). Studies have found correction factors for clay content are relatively low, ranging from 0.0851% to 0.1046% weight loss per % clay (Grewal et al. 1991; De Vos et al. 2005), therefore relatively few studies apply this correction. The type of clay mineral will also partly determine structural water loss. For example, Sun et al. (2009) examined water loss on various soil minerals to obtain water loss values for different mineral types and found that kaolinite and vermicu-late have higher structural water loss compared to other minerals. X-Ray Diffraction (XRD) or other thermal techniques (see below) are effective to quantify clay mineral types.

Common laboratory methods to measure texture of the soil include the hydrometer (Bouyoucos 1962; Beretta et al. 2014) and pipette methods (Miller and Miller 1987), both of which use Stoke’s Law and sedimentation rates of particle fractions (i.e., sand, silt, and clay) to determine the percentages of each. Samples are ideally pre-treated by both chemical and mechanical methods to aid in dispersion and separation of aggregates. The hydrometer method involves measuring density of the particles in suspension at specific times, depending on the particle size of interest. The pipette method involves removing clay particles in suspension with a pipette and separating sand particles with a 53.3 μm screen. The clay and sand particles are then dried and weighed to determine the content of each particle size. See Gee and Bauder (1986) for more detailed methods on particle size analysis.

The ribbon method is a qualitative test to assess texture, which is useful because it can be conducted relatively quickly in the field by an experienced technician. However, results are highly variable among personnel depending on training. Rabenhorst and Stolt (2012) demonstrated that experience, training, and practice using known samples improves consistency and reliability of results. In some cases, performing the ribbon method will not be possible, such as with fluidic wetland soils. Also, high SOM content can act as clay while ribboning. The ribbon test method involves starting out with a moistened ball of soil (~ 25 g) and squeezing it into a ‘ribbon’. The length of the ribbon will give an idea of the soil type. If the ribbon is 2.5 to 5.0 cm in length that indicates the soil is a clay loam while a ribbon length of 5 cm or longer indicates a clay soil. Based on the soil type, the Soil Textural Triangle can then be used to estimate % clay in the soil. See the U.S. Department of Agriculture Natural Resources Conservation Service Soils ‘Guide to Texture by Feel’ for more information and graphics on the ribbon test method and the soil textural triangle (USDA 2023b).

*Oxidation–reduction potential:* A number of C-related biogeochemical processes, from litter decomposition to methanogenesis, are influenced by the oxidation–reduction (redox) potential of wetland soils. Redox meters measure the electrical potential (E_h_) between a measurement platinum electrode and a reference electrode using a high impedance voltmeter. E_h_ is typically converted and reported as oxidation–reduction potential of soils, with values above 300 mV reflecting oxidized (aerobic) conditions and lower values reflecting increasing intensity of reducing (anaerobic) conditions. Commercially available redox meters are typically coupled with pH in multimeters because redox values are pH dependent. Automated and controller options are also available (Megonigal and Rabenhorst 2013; Yu and Rinklebe 2013). Ideally, measurements are conducted in the field or in laboratory mesocosms since redox can change rapidly once a core is extracted. Soil redox is highly variable in space and time but electrodes only measure E_h_ at point scales, so there can be high variability among electrodes, therefore it is important to have high spatial (cm) and temporal replication.

Another option to measure longer-term redox conditions is to use Indicator of Reduction in Soil (IRIS) devices (Sapkota et al. 2022). IRIS devices involve the use of Fe or manganese (Mn) oxide-based paints on materials such as PVC pipes. The pipes are inserted into the ground typically to 0.5 m depth and left for a given period of time. The paint fades in response to reduced conditions. Chemical dyes can also be used to identify Fe^2+^, an indicator of anerobic, reduced soils. Dyes can be added to freshly exposed soils using a sprayer or dropper, or to soils in a vial, and then observe a pink or red color to indicate Fe^2+^ (Berkowitz et al. 2017).

*pH:* Soil pH in wetlands can range from acidic, to neutral, to alkaline (< 4–10). Organic soils tend to be more acidic than mineral soils, although there are many exceptions. Flooding, anoxia, and the build-up of soil CO_2_ and carbonic acid can influence pH as, upon flooding, the pH of both acidic and basic soils approaches neutrality (Shotyk 1988; Gambrell 1994; Mushet et al. 2015b). Depending on the accuracies needed, soil pH can be measured in the field, in intact cores, or in a laboratory using commercially available bench pH meters in a soil solution with a fixed ratio (often 1:1) of soil to deionized water. Again, ideally pH is measured in the field since pH may decrease when soils are dried and oxidation and production of hydrogen ions occurs, especially in soils high in sulfides (e.g., salt marsh and mangrove soils). For detailed methods on measuring soil pH see Thomas (1996).

*Salinity/Electrical conductivity:* Salinity of soils can influence wetland vegetation and microbial processes, and thus has a large influence on wetland C cycling (e.g., Trites and Bayley 2009; Tuxen et al. 2011; Baustian et al. 2017; Luo et al. 2019). Salinity has been shown to be negatively associated with various microbial community metrics (e.g., Zhao et al. 2017; Zhang et al. 2021b), as well as with CH_4_ emissions (Poffenbarger et al. 2011; Qu et al. 2019; Servais et al. 2019). For example, increases in salinity, as occurs in coastal wetlands as sea level rises, may affect plant community composition and sedimentation, which can influence accumulation rates of soil C (Craft 2007; Herbert et al. 2021). Electrical conductivity varies as a function of the amount and types dissolved salts in soils, with higher values indicating higher salinity. Electrical conductivity can be measured using commercially available probes in the field, in intact cores, or in the laboratory in soil slurries with deionized water. For detailed methods on measuring salinity and electrical conductivity see Rhoades (1996). For a discussion on salinity definitions, units, and methods for salinity in water see Section “Carbon in Wetland Waters”.

*Other compounds in soil organic matter:* Knowing the relative fractions of compounds such as lignin and carbohydrates is useful for understanding the sources and fate of soil C. These compounds can be measured using a variety of spectroscopic and biochemical techniques to help discern sources and sizes of soil C (Section “Litter and Organic Matter Decomposition”). Newer techniques include: 1) X-ray imaging and C speciation analysis using scanning transmission X-ray microscopy with near-edge X-ray absorption to elucidate the spatial distribution of organic matter (Seyfferth et al. 2020); 2) solid state carbon-13 (^13^C) Nuclear Magnetic Resonance (NMR) spectroscopy to analyze C molecular structure (Kaal et al. 2019); 3) Fourier-Transform Ion Cyclotron Resonance-Mass Spectrometry (FT-ICR-MS) to assign elemental compositions to individual SOM molecules (Bahureksa et al. 2021); and 4) Nominal Oxidation State of Carbon (NOSC) to estimate the energetic potential of the soil C (LaRowe and Van Cappellen 2011; Dalcin Martins et al. 2017). These techniques are reproducible, but equipment is expensive and requires experienced personnel for operation.

Differential Scanning Calorimetry (DSC), Differential Thermal Analysis (DTA), and Thermogravimetric Analysis (TGA) are rapid and inexpensive techniques to quantify the components and stability of SOM based on relationships between mass loss and/or heat flux with temperature (Fernández et al. 2012). The thermal stability of SOM is a function of its mineral associations and chemical composition, and these methods can provide information on SOM decomposability (Plante et al. 2009).

*Black carbon:* Black C (fine particulate material formed by incomplete combustion of fossil fuels, i.e., soot, charcoal) is an important component of the global C cycle and contributes to SOM stabilization (Kuhlbusch 1998; Leifeld 2007). Black C can be measured using DSC, TGA, or with CHN analyzers (Leifeld 2007; Ding et al. 2014; Guo et al. 2018). Analysis of black C requires several pre-treatment steps to separate black C from other C forms (i.e., carbonates and volatile organic matter) through combustion at 340 to 375 °C for 2 to 24 h. The residual black C can then be analyzed using DSC, TGA, or CHN instruments (see Schmidt and Noack 2000; Gélinas et al. 2001; Caria et al. 2011).

*Quantification and speciation of elements beyond carbon:* Determination of the concentration and speciation of elements in addition to C can provide important information on processes that affect wetland C cycling and storage. It is ideal to plan for these analyses before sample collection to ensure enough material is available and the proper preservation procedures are followed. A complete review of the approaches for element quantification and speciation is beyond the scope of this review, but some examples are provided.

*Nitrogen and Phosphorus:* N and phosphorus (P) are limiting nutrients that typically control rates of plant production and microbial metabolism, and thus influence the organic matter and C contents of wetland soils. The concentration and speciation of forms of organic and inorganic N and P affect their influence on C dynamics, and also N_2_O fluxes. Multiple wetland characteristics (e.g., hydrology) influence how soil C responds to changes in N and P concentrations (e.g., from nutrient enrichment) (Mozdzer et al. 2020); therefore, caution is advisable in interpreting N and P data in relation to soil C in the absence of C process rate measurements (e.g., GHG flux).

Various anthropogenic activities can increase N input into wetlands, which results in changes in the C:N ratio, an important factor for C accumulation and emission. A high C:N ratio, where organic matter in the soil is relatively rich in C and poor in N, can result in reduced microbial activity and greater C content in soils (Reddy and DeLaune 2008). On the other hand, a low C:N ratio is indicative of higher decomposition rates that result in the release of CO_2_ to the atmosphere (Enríquez et al. 1993).

The most common, accurate, and effective method to measure soil total N is by CHN elemental analyzers described in this section (Nelson and Sommers 1996; Chatterjee et al. 2009). Analyzing for total N when measuring total C of a sample is particularly efficient, requiring inclusion of N calibrants and external standard reference materials that are similar to the soil type of the samples being analyzed. The bioavailable fractions of N (e.g., nitrate [NO_3_^−^], nitrite [NO_2_^−^], and ammonium [NH_4_^+^]) are typically estimated using selective chemical extractions (Reddy et al. 2013) and spectrophotometer analysis, similar to P.

P is another limiting nutrient that regulates plant growth and microbial activity in wetland ecosystems. However, P is often non-limiting for wetlands that receive relatively high inputs from agricultural runoff. Similar to N, higher availability of P in systems with low C:P ratios can stimulate biological activity (Reddy et al. 1999). Various forms of both inorganic and organic P are found in wetland soil. Organic P is bound to organic matter such as dead plant or microbial biomass, and is not readily available to plants and microbes until mineralized to simpler, inorganic forms. Inorganic, plant-available forms of P include phosphate (PO_4_^3−^), hydrogen phosphate (HPO_4_^2−^), and dihydrogen phosphate (H_2_PO_4_^−^), collectively known as orthophosphates. However, these inorganic forms can also be bound with elements such as calcium (Ca), Fe, and aluminum (Al) in sediments and become immobilized.

For analysis, soil samples are first prepared (drying, grinding, sieving), then total P is determined through acid digestion using mixture of strong acid such as sulfuric acid (H_2_SO_4_), perchloric acid (HCLO_4_), and nitric acid (HNO_3_). Other examples of extractants include HCl and ammonium fluoride (NH_4_F) (Bray and Kurtz 1945), sodium bicarbonate (NaHCO_3_) (Olsen et al. 1954), Mehlich 3 extractant (Mehlich 1984), H_2_SO_4_, and hydrogen peroxide (H_2_O_2_) (Akinremi et al. 2003). The choice of extractant is dependent on properties of the soil sample (e.g., pH). Total P concentration is estimated by colorimetry based on absorbance values obtained from the spectrophotometer analysis, which involves diluting for bench analysis using water and reagents such as ammonium molybdate [(NH_4_)_6_Mo_7_O_24_] (Murphy and Riley 1962). Colorimetric analysis is the more modern approach compared to the gravimetric and volumetric methods (Sherrell and Saunders 1966).

*Sulfur:* Sulfur (S) is an example of an element that is tightly coupled with C cycling as an essential nutrient critical for multiple biochemical functions including photosynthesis (Balk and Pilon 2011; Walsh 2020). S is also a highly redox-active element with oxidation states from − 2 (e.g., sulfide [S^2−^], many organosulfur compounds) to + 6 (e.g., sulfate [SO_4_^2−^]). Dissimilatory SO_4_^2−^ reduction is an important terminal electron accepting process for the anerobic oxidation of C that inhibits methanogenesis and produces S^2−^ (Martens and Berner 1974; Pester et al. 2012). S^2−^ influences the cycling of Fe and other metals (e.g., Cd, Pb) through the formation and sequestration of insoluble metal sulfides (Zeng et al. 2013; Smieja-Król et al. 2015; Julian et al. 2017). In the case of Fe, this has the potential to affect the stabilization of organic C by reactive Fe^3+^ phases (Lalonde et al. 2012; Johnston et al. 2014). S is abundant in coastal wetlands due to the high concentration of SO_4_^2−^ in seawater (~ 28 mM) but can also be present at substantial concentrations in inland wetlands due to underlying geology (e.g., the Prairie Pothole Region of North America; Goldhaber et al. 2014), evapoconcentration, and pollutants from agricultural runoff (Bates et al. 2002) and acid mine drainage (Aguinaga et al. 2018).

There are a variety of methods for the determination of total S in soils. Wet oxidation methods followed by reduction and colorimetric detection of S^2−^can be performed with limited instrumentation but are labor intensive (Tabatabai and Bremner 1970). More modern total S techniques include sample combustion and detection of the resulting sulfur dioxide (SO_2_) gas with an elemental analyzer (Leitão et al. 2001) and determination of total S by Inductively Coupled Plasma (ICP)-optical emission spectroscopy or mass spectrometry after soil digestion as discussed for *multi-element analyses* (see below; Mahanta et al. 2017). Elemental analyzers are more cost effective and require less technical expertise than ICP instruments but are limited to analysis of C, N, S, and H, while ICP instruments can analyze numerous major and trace elements (but not C, N, or H). X-Ray Fluorescence (XRF) spectroscopy is a potential non-destructive technique for total S determination (e.g., Zhao et al. 2020).

Soil S species can be isolated and quantified through various wet chemistry techniques. For example, SO_4_^2−^ can be extracted from soils using water or other aqueous extractants and quantified by ion chromatography or other methods (Ketterings et al. 2011). Reduced S species including monosulfides (i.e., acid volatile S^2−^), more recalcitrant disulfides (e.g., pyrite), and residual organosulfur, can be isolated and trapped as S^2−^ precipitates and SO_4_^2−^ for quantification by gravimetry or instrumental methods (Tuttle et al. 1986; Bates et al. 1993; Duan et al. 1997). X-ray Absorbance Near-Edge Structure (XANES) spectroscopy is a powerful method that can quantify and spatially map S species directly in the solid phase, but requires access to an appropriate synchrotron beamline (Prietzel et al. 2011; Zeng et al. 2013). Given the sensitivity of many reduced S compound to exposure to O_2_, care needs to be taken during collection, transport, and storage of samples intended for speciation to minimize oxidation.

*Multi-element analyses:* A variety of multi-element analysis packages are available from commercial and academic laboratories. Due to signal interferences between some elements and sample matrix effects, multi-element analyses can be challenging and require expertise and extensive quality control measures. The accuracy and precision of results can be assessed by submitting blind quality control samples such as replicates and certified standards. The numerous methods for determining total concentrations of multiple elements in bulk soil or sediment include those requiring minimal sample pretreatment (non-destructive) and those requiring dissolution or decomposition to get the sample into solution before analysis. Common non-destructive methods are XRF spectroscopy and Instrumental Neutron Activation Analysis (INAA). Both methods are based on measuring radiation emitted from a sample that has been subjected to incident radiation, but XRF is more common since INAA requires irradiation of the sample by neutrons in a nuclear reactor. Wavelength Dispersive XRF (WDXRF) methods are laboratory based and typically report on the order of ten major elements (e.g., Ca, silicon [Si], sodium [Na], Fe). Energy Dispersive XRF (EDXRF) instruments are often portable and can provide quick, non-quantitative estimates of many element concentrations including trace elements (e.g., lead [Pb], zinc [Zn], barium [Ba]) in the field. However, quantitative measurements require user training, instrument calibration, consideration of interferences and matrix effects, and quality control measures. INAA can provide very low detection limits for many trace elements including the rare earth elements (e.g., lanthanum [La], neodymium [Nd]). Sample dissolution methods typically heat a homogenized and ground sample with a mixture of concentrated acids. Once dissolved, the sample is diluted, and the solution analyzed by Inductively Coupled Plasma Mass Spectrometry (ICP-MS) and/or Inductively Coupled Plasma Optical Emission Spectrometry (ICP-OES). ICP-MS and ICP-OES are optimal for different elements and thus the two instrumental techniques are often used in combination and methods can report concentrations for up to 60 elements. Some elements are not amenable to acid dissolution methods (e.g., Si, chromium [Cr]) and require a decomposition treatment referred to as a fusion, followed by ICP-MS and/or ICP-OES analysis. Fusion methods can report many of the same elements as acid digestions, but typically have higher detection limits.

*Multi-element fingerprinting:* Multi-element fingerprinting is an approach that applies multivariate statistical techniques to concentrations of suites of elements, usually 30 or more, including major (e.g., Na, magnesium [Mg], Ca, potassium [K], Al), trace (e.g., barium [Ba], arsenic [As], Cr, Zn, cobalt [Co], cesium [Cs], silver [Ag], cadmium [Cd], copper [Cu], Mn, nickel [Ni], Pb, antimony [Sb], selenium [Se], vanadium [V], hafnium [Hf]), and rare earth elements (the lanthanides, scandium [Sc] and yttrium [Y)]. Variation in elements among wetlands occurs from differences in biogeochemical activity and hydrology. Multi-element analysis has been used to assess the condition of restored wetlands (Wang et al. 2019a, 2020; Zhu et al. 2021), characterize vegetation change (e.g., Jacob and Otte 2004), identify sources of water and sediments (e.g., Rauch et al. 2000; Stutter et al. 2009), and assess hydrochemical connectivity of wetlands (e.g., Yuan et al. 2019; Zhu et al. 2019b). For example, distributions of elements La, praseodymium [Pr], terbium [Tb], bismuth [Bi], thallium [Tl], and thorium [Th] provide evidence of disturbance from agricultural activities at depths greater than 1 m (Yellick et al. 2016; Werkmeister et al. 2018), while Co and Ni provide information about conversion of wetlands to croplands (Zhu et al. 2021).

The elements Fe and Al are also important indicators of land-use effects. Reactive Fe and Al minerals are correlated with C content in upland soils (Rasmussen et al. 2018) and can stabilize wetland C from drainage-induced oxidation (Anthony and Silver 2020). Reactive and mineral phases of Fe and Al can be quantified after sequential extractions (Zimmerman and Weindorf 2010). Under anoxic or fluctuating redox conditions, it is critical to employ methods of sampling and analysis that would preserve Fe^2+^ in the sample (e.g., Viollier et al. 2000).

*Stable Isotopes:* Relative abundances of the naturally occurring stable isotopes of C (carbon-12 [^12^C], ^13^C) and N (nitrogen-14 [^14^N], nitrogen-15 [^15^N]) are used to provide information on the source and fate of organic matter (concepts reviewed in Fogel and Cifuentes (1993) and Rounick and Winterbourn (1986)). They can be measured in bulk pools (i.e., soil, water, and gas) or in specific compounds (e.g., lignin) after separation, combustion, and/or reduction into target analyte gases (CO_2_, N_2_), followed by analysis by isotope ratio mass spectrometry or cavity ring down spectroscopy. Bulk analyses integrate all biologic organic matter sources and isotope fractionation process, while analyses of specific compounds contribute information on their origin sources and fates (Benner et al. 1987; Liu et al. 2020). For example, δ^13^C values of lignin monomers—a biomarker of vascular plants—were used to quantify relative contribution of river, marsh, and marine sources in offshore sediments (Bianchi et al. 1997). Similarly, n-alkanes were used to determine variation in marsh vegetation inputs (Wang et al. 2003).

Stable isotope compositions are typically reported using delta (δ) notation which expresses per mil (‰) differences relative to an international standard. In this notation, relatively higher values are enriched, and lower, more negative values are depleted in the heavier, less abundant isotope relative to the standard. There are a variety of terrestrial and aquatic sources of organic matter into wetland soils that can be identified by isotope analyses. The δ^13^C values are normalized to a carbonate standard Vienna Peedee Belemnite (VPDB) that is used as a reference zero point (Hoffman and Rasmussen 2022). The δ^13^C values of terrestrial plants vary with their photosynthetic pathway: plants with C_3_-type photosynthesis (e.g., trees; approximate δ^13^C range of − 22 to − 35‰) are more depleted in ^13^C than plants with C_4_-type photosynthesis (e.g., warm-season grasses; approximate δ^13^C range of − 9 to − 19‰) (Fry 2006). δ^13^C values of plant communities also differ along salinity gradients, allowing for assessment of past hydrologic and salinity conditions (e.g., sea-level change) using sedimentary C (e.g., Chmura et al. 1987; Chmura and Aharon 1995). Aquatic plants and algae have a wider range of δ^13^C values (approximate δ^13^C range of − 39 to − 11‰) and are ideally characterized for each site (Farquhar et al. 1989). Marine phytoplankton have less variable δ^13^C values (approximate δ^13^C range of − 19 to − 24‰) than freshwater algae, but their intermediate δ^13^C values can hinder interpretation in systems with mixed C_3_ and C_4_ terrestrial inputs (Fogel and Cifuentes 1993). If soils have just two isotopically distinct potential sources of C, simple two-pool mixing models can be used to determine the relative contributions of each source (Balesdent et al. 1987). This approach is ideal for wetland systems that have experienced changes in land cover (sensu Bianchi et al. 2013). However, many wetland systems have multiple sources of organic matter (e.g., terrestrial plants, aquatic vegetation, and phytoplankton) that require multi-isotope approaches (i.e., δ^13^C, δ^15^N, δ^34^S), probabilistic modeling, or more advanced analytical techniques.

Depending on the analyte, analysis of δ^13^C and δ^15^N can require specialized equipment with significant costs for operation. However, commercial stable isotope facilities can provide δ^13^C and δ^15^N data for moderate costs for bulk soils, with some facilities providing analyses of specific compounds for a higher fee. Compound-specific analyses rely on extraction of target biomarkers, separation by gas chromatography, combustion, reduction (if applicable), and analysis by isotope ratio mass spectrometry.

*Sulfur Isotopes:* There are four stable isotopes of S (sulfur-32 [^32^S], sulfur-33 [^33^S], sulfur-34 [^34^S], and sulfur-36 [^36^S]).Most reported values are δ^34^S, which compares the sample ^34^S/^32^S ratio to a meteorite international standard (Canyon Diablo troilite). Much of the wide variation in δ^34^S values results from the strong isotopic fractionation effects of anaerobic SO_4_^2−^ reduction and other microbially-mediated S transformations (Faure 1986; Canfield et al. 1998). The fractionation effect of SO_4_^2−^ reduction is influenced by the rate of SO_4_^2−^ reduction and δ^34^S of the resulting S^2−^ can be up to 70‰ more negative than δ^34^S of the starting SO_4_^2−^ (Jørgensen et al. 2019). Thus, stable S isotope ratio measurements of SO_4_^2−^ and S^2−^ pools can provide insight into the role of SO_4_^2−^ reduction on oxidation of organic C in wetland systems (Wu et al. 2011; Guo et al. 2020). The variation of δ^34^S values of S pools can also be used to trace S inputs into wetland systems (e.g., Bates et al. 2002) and as an additional natural abundance isotopic tracer to source organic matter (Peterson and Howarth 1987; Finlay and Kendall 2007; Maier et al. 2011). S stable isotope measurements have traditionally been made by combustion to SO_2_ on an elemental analyzer followed by isotope ratio mass spectrometry. Compound specific S isotope measurements employing on-line separation by gas chromatography were not possible until developments that couple gas and liquid chromatographic separation to Multicollector-Inductively Coupled Plasma-Mass Spectrometry (MC-ICP-MS) (Amrani et al. 2009; Martinez et al. 2019). Another recent analytical advance is the accurate measurement of ^33^S/^34^S ratios (δ^33^S), which can augment the interpretation of δ^34^S measurements (Jørgensen et al. 2019).

### Carbon in Wetland Waters

#### Definitions and Units

**Fig. 7:**
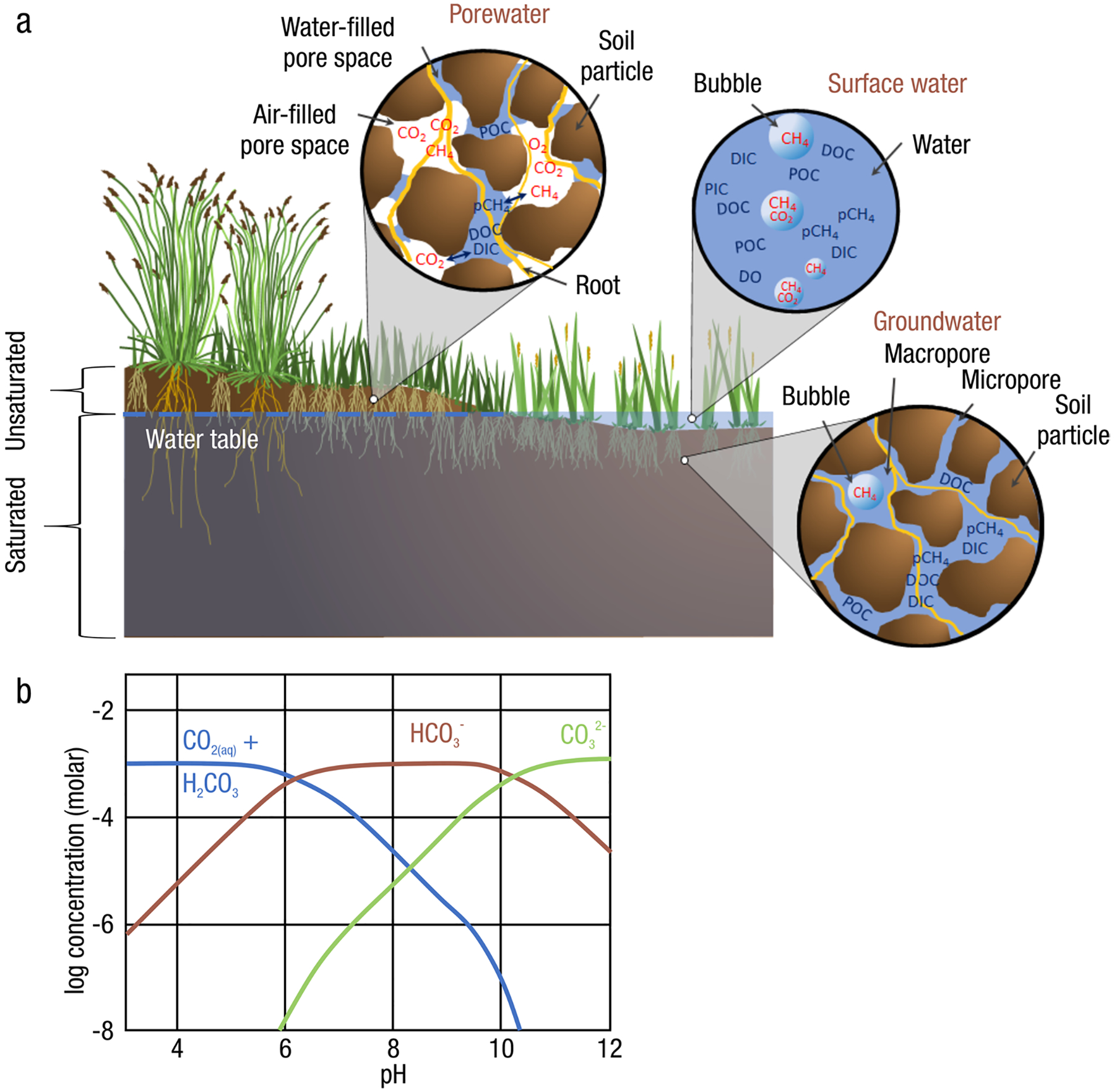
(**a**) Conceptual diagram of wetland porewater, surface water, and groundwater and associated carbon (C) constituents including gaseous (red) and dissolved (dark blue) carbon dioxide (CO_2_, *p*CO_2_), methane (CH_4_, *p*CH_4_), and oxygen (O_2_, DO), Dissolved Organic C (DOC), Dissolved Inorganic C (DIC), Particulate Organic C (POC), and Particulate Inorganic C (PIC). Note that porewater is technically located in the unsaturated zone below the soil surface and above the water table; however, the term ‘porewater’ is typically used in the wetland scientific literature to indicate any water near the sediment surface, such as in the root zone, even if soils are saturated and below the water table; (**b**) DIC speciation for brackish water at 25 °C and 5 g kg^−1^ salinity (5,000 ppm). Images with permission from Kimberly P. Wickland (a) and based on Stumm and Morgan (2012) (b). [CO_2(aq)_, aqueous or dissolved CO_2_; CO_3_^2−^, carbonate ion; HCO_3_.^−^, bicarbonate ion; H_2_CO_3_, carbonic acid]

*Definitions:* Organic and inorganic C compounds are present in dissolved and particulate forms in wetland surface water, porewater, and groundwater (Fig. 7a). Organic C in water originates from algal cells, plant litter, plant root exudates, SOM, and microbial biomass and exudates. The Total Organic C (TOC) pool in wetland waters is divided into Dissolved Organic C (DOC) and Particulate Organic C (POC) pools. In theory, DOC represents all non-colloidal soluble forms of organic matter. In practice, however, there is an operational distinction between POC and DOC based on molecular size. DOC is the fraction of the POC pool that passes through filters with sub-micrometer pore sizes (~ 0.2–0.7 μm) and POC is the portion that is retained on these filters (Aiken 2014). The partitioning of aqueous C into DOC and POC is due to differences in their transport and biogeochemical processing in water, as well as methodological considerations (Battin et al. 2008; Worrall and Moody 2014). DOC and POC are the C fraction of aquatic organic compounds which contain other elements, such as N, S, and P as part of the mass in Dissolved Organic Matter (DOM) and Particulate Organic Matter (POM). Within each of these C pools, there is a diversity of compounds with unique biogeochemical functions.

Inorganic C in wetland waters is dominated by Dissolved Inorganic C (DIC). DIC in wetlands is derived primarily from CO_2_ released by plant and microbial respiration and shell dissolution that dissolves in water and then dissociates into three species: 1) aqueous or dissolved CO_2_ (CO_2(aq)_) defined as dissolved free CO_2_ (most likely > 99%) plus carbonic acid (H_2_CO_3_) (most likely < 1%); 2) bicarbonate ion (HCO_3_^−^); and 3) carbonate ion (CO_3_^2−^):

(10) $$\text{DIC}={\text{CO}}_{2(\text{aq})}+{{\text{HCO}}_{3}}^{-}+{{\text{CO}}_{3}}^{2-}$$

The relative proportions of each DIC constituent and DIC speciation are dictated by acid/base equilibria (Fig. 7b), which are controlled by a range of physical and biogeochemical conditions or processes, such as ionic strength, temperature (e.g., CO_2_ solubility and dissociation constants), biological activities (e.g., photosynthesis and respiration), and the effects of other acid–base species and reactions (e.g., PO_4_^3−^, silicate, and organic acid species). At low pH (< 5), most DIC is present as aqueous or dissolved CO_2_ (Fig. 7b). As pH increases, HCO_3_^−^ increases and becomes equal in proportion to dissolved CO_2_ at pH ~ 6.3. At pH ~ 7.0, HCO_3_^−^ is greater than dissolved CO_2_. At pH > 8.0, HCO_3_^−^ becomes dominant. At pH ~ 10.3, HCO_3_^−^ and CO_3_^2−^ are equal. Above pH 11.0, DIC is mostly as CO_3_^2−^ (Cole and Prairie 2010; Stumm and Morgan 2012). The acid–base reactions that inter-convert one form of DIC into another are rapid and often assumed to be in equilibrium. Established equilibrium equations (Butler 1991; Stumm and Morgan 2012) can be used to estimate the composition of the DIC pool, but these calculations can have large errors because of other acid species and uncertainties in carbonate solubility constant values (Stumm and Morgan 2012; Song et al. 2020b; Kerr et al. 2021). Aqueous geochemical modeling software (e.g., PHREEQC; Parkhurst and Appelo 2013; Orr et al. 2018) can be used to more accurately speciate DIC if the major ion composition of the water is known.

In addition to DIC, another widely measured parameter is *p*CO_2_ which is the partial pressure of CO_2_ in a gas phase that is in equilibrium with dissolved CO_2_. According to Henry’s law, the concentration of a dissolved gas is directly proportional to its partial pressure in a gas phase in equilibrium with the solution (Henry 1803; Sander et al. 2022). For the specific case of CO_2_:

(11) $$p{\text{CO}}_{2}={\text{CO}}_{2(\text{aq})}/{\text{k}}_{\text{H}}$$

where k_H_ is the solubility coefficient (Henry’s constant) for CO_2_. The concentration of dissolved CO_2_ and the *p*CO_2_, are one of the primary interests for wetland C cycling as they are directly linked to respiration and gas flux, and thus can be highly dynamic. It is important to note that, most wetland waters, whether freshwater or saline, will be supersaturated in CO_2_ with respect to the atmosphere (i.e., the *p*CO_2_ of the water is greater than the partial pressure of CO_2_ in the atmosphere) and therefore wetland waters will typically be a source of CO_2_ to the atmosphere (Cole et al. 1994; Cai 2011; Wang et al. 2016).

In tidal systems, diel fluctuations in *p*CO_2_ can be driven by tide direction, tide height, water or air temperature, or other site-specific factors (Turner et al. 2023). Regional climate can be especially important as large differences in water and air temperature will drive air-sea gas transfer. Some tidal systems are net ecosystem calcifying and will dissolve carbonate minerals, whereas others will precipitate CaCO_3_. Carbonate chemistry and its effect on *p*CO_2_ and ecosystem metabolism over annual timescales is an active and developing field of research. Particulate Inorganic C (PIC), defined as inorganic material (namely CaCO_3_) that is larger than a size threshold (e.g., > 0.2 or 0.45 μm), is generally not prevalent in most wetlands, but can be present in some carbonate-rich or high-pH wetland systems in significant amounts (Ye et al. 2015).

Another important form of C in wetland waters is dissolved CH_4_, an organic gas produced from anaerobic C decomposition. CH_4_ is much less soluble in water than CO_2_ and does not ionize into different species. Like dissolved CO_2_, dissolved CH_4_ is highly dynamic in wetland waters due to variable rates of production, consumption, and transport (Segers 1998), as well as factors affecting solubility. CH_4_ solubility decreases with increasing water temperature (as all gases do), and excess CH_4_ (under non-equilibrium conditions) can come out of solution and form bubbles that can be released through ebullition.

*Units:* DOC and POC concentrations are commonly reported in units of mg C L^−1^ and μmol C L^−1^ or mmol C L^−1^ (also written in molar C convention, e.g., μM C). Concentrations of DIC and its constituents (e.g., CO_2_), and dissolved CH_4_ are commonly reported in units of mg C L^−1^, μmol C L^−1^ (also written as micromolar C, μM C), and μmol C kg^−1^ water (this unit is typically used in the ocean sciences community). The *p*CO_2_ or the partial pressure of CH_4_ (*p*CH_4_) are commonly expressed in units of atmosphere (atm), pascals (Pa), or parts per million by volume (ppmv).

**Rationale:** C in wetland waters is often the smallest C pool in wetlands, but the most ‘fluid’ and subject to transport atmospherically and hydrologically, which has important relevance to and implications for wetland C processes and budgets (Tranvik et al. 2009). Porewater dissolved C constituents can provide information on C cycling process rates and pathways, especially when coupled with analyses such as stable C isotopes (Chasar et al. 2000; Hornibrook et al. 2000). Dissolved gases in surface water can be emitted to the atmosphere, and porewater concentration profiles are often used to model fluxes to the atmosphere, especially in tidal wetlands (Windham-Myers et al. 2018). DOC and POC can be mineralized to CO_2_ and/or CH_4_ for eventual emission to the atmosphere or can be transported laterally to hydrologically connected waters including lakes, streams, rivers, and estuaries. Wetland DIC and dissolved CH_4_ can also be transported laterally and, in combination with DOC and POC, can be important components of net ecosystem C budgets (Buffam et al. 2011; Webb et al. 2019; Section “Lateral Flux”).

#### Water Sample Collection – Surface Water, Porewater, Groundwater

*What:* The collection of aquatic samples is the first step to understanding and quantifying C pools in wetland waters. The source of water has important implications for its role in wetland C cycling and biogeochemical processes. C constituents in any wetland water are often subject to rapid changes in concentrations and forms due to a suite of biological processes, geochemical reactions, and physical mechanisms.

There are three main sources of water: surface water, porewater, and groundwater. Surface water is any water, flowing or non-flowing, above the soil surface. Surface-water inputs include precipitation, snowmelt, river or tidal flows, groundwater, or storm runoff from uplands. Surface-water outputs include evapotranspiration (which is loss of pure water, and thus concentrates the remaining aquatic C constituents) and lateral export (which carries aquatic C constituents in hydrologic flows). Porewater and groundwater are both located below the land surface. By some definitions, porewater is the water in unsaturated soil or sediment located between the water table and land surface, also referred to as the vadose zone. In unsaturated soils, the water is held by a tension or suction. However, the term ‘porewater’ is often used in the scientific literature to indicate water that is relatively close to the land surface (e.g., in root zones of plants), even if soils are saturated and above the water table, which is how the term is used in this review. Groundwater is water that is in the saturated zone below the water table, and often flows from areas of higher to lower total head (e.g., lower elevation; Section “Groundwater Inputs and Exports”). However, the distinction between porewater and groundwater is not always clear in the scientific literature. The terms are sometimes used interchangeably or are only loosely defined. It is also common in some disciplines, such as marine science, to define ‘groundwater’ as any water located belowground. The line between porewater and groundwater can be particularly blurry in tidal wetlands where the water table and the direction of the hydraulic gradient can shift rapidly over relatively short tidal timescales of just a few hours (e.g., Wilson et al. 2015b). Groundwater inputs to wetlands include those from adjacent uplands where hydraulic head is higher than the wetland surface, lateral flow from adjacent aquatic systems, or vertical flow from underlying (Cook et al. 2018a) or overlying (Wilson et al. 2015a) saturated sediments. Groundwater export can include lateral flow to adjacent aquatic systems (Section “Groundwater Inputs and Exports”).

**Fig. 8:**
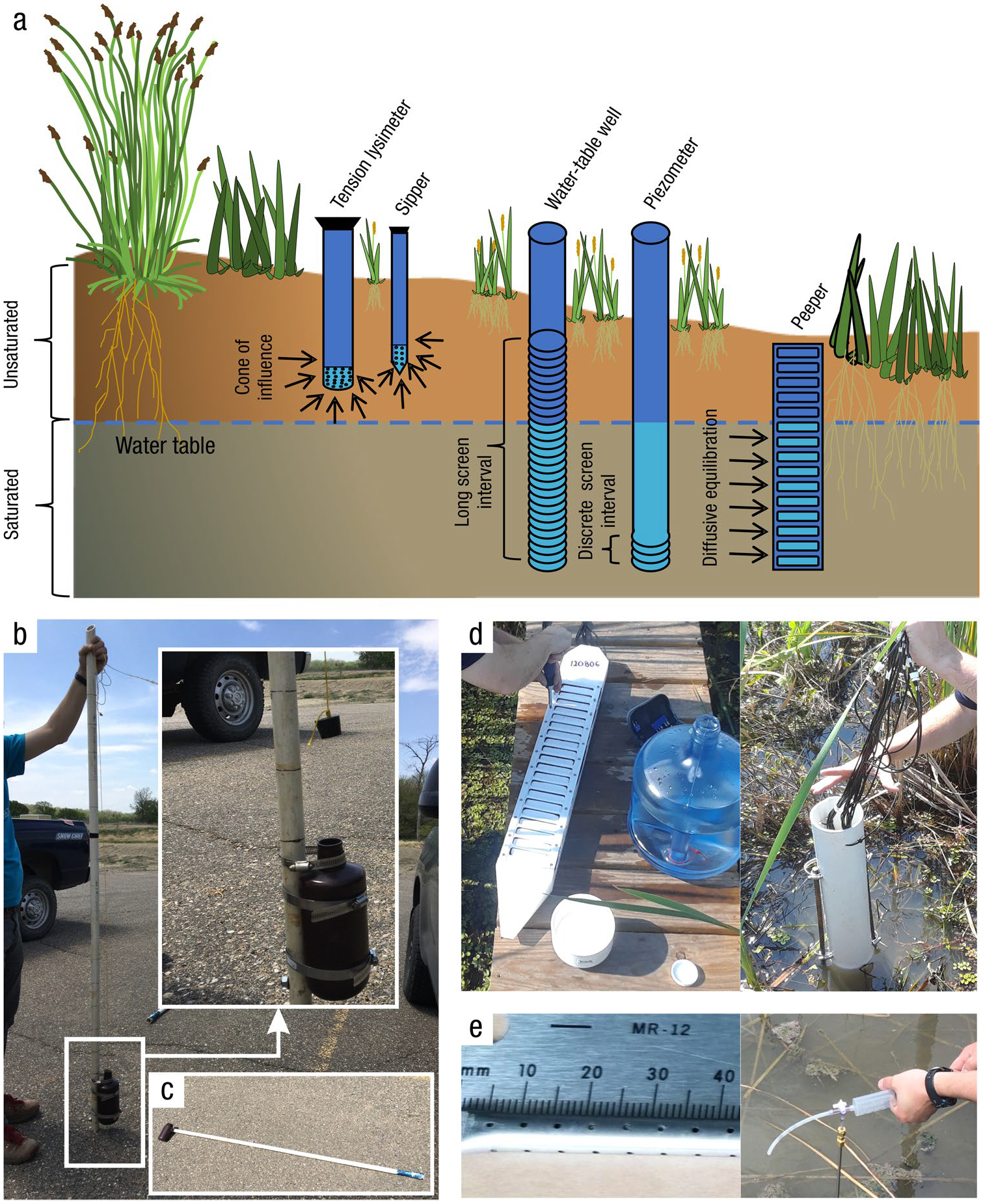
Methods to sample surface water, porewater, and groundwater: (**a**) schematic diagram of tension lysimeter, sipper, water-table well, piezometer, and peeper; (**b**) water sampler devices to collect deeper water: a plastic bottle attached to a 2 m polyvinyl chloride (PVC) pole using plumbing clamps with a string attached to a rubber stopper (inset photo); (**c**) a plastic bottle attached to a 2 m PVC pole using zip ties to sample surface water from the shore or from a boat to avoid disturbance; (**d**) peeper (also called dialysis sampler) with vertically stacked independent wells (left) to measure dissolved constituents as described in MacDonald et al. (2013). Each sample well is connected to Tygon tubing to collect sample water and re-fill with deionized water (right); (**e**) metal tube sipper (left) with holes along the bottom 60 mm for collection of subsurface porewater or shallow groundwater (right); Images with permission from Olivia Johnson (b, c, e) and Jorge Villa (d)

In general, the principal methods for in situ collection of porewater and groundwater involves either equilibration or suction techniques (Fig. 8a; Table 5) (Bufflap and Allen 1995; Teasdale et al. 1995; Fisher and Reddy 2013). Equilibration is a passive sampling method, which relies on either short-term diffusion or movement of aqueous constituents into a sampling chamber or tube (e.g., peeper, porewater equilibrator) or longer-term diffusion and/or advection of groundwater into piezometers or water-table wells. In contrast, suction is an active sampling method, in which water samples are extracted under negative pressure (suction) with lysimeters or sippers, often using a pump or syringe. In instances when suction required is greater than one atmosphere, a pump is needed to push rather than pull the groundwater to the surface.

*Where:* There are many locations within a wetland system where determination of aquatic C and dissolved GHG concentrations could be of interest for C pool assessments, such as in surface-water bodies (e.g., localized pools or flooded areas) or along vertical profiles in the soil or water column (Campeau et al. 2017). If defined flow-through wetlands are present, water samples can be collected from the inlets and outlets. Neglecting the hydrological pathway through draining streams has been shown to result in significant overestimation of the C sink strength of many wetlands (Dinsmore et al. 2010; Leach et al. 2016).

Surface water can be collected just below the surface of water (commonly referred to as the ‘water-atmosphere interface’), as well as deeper into the water column down to the bottom (‘sediment–water interface’). Walking through a wetland can alter vertical profiles, stir up sediment and porewater, and contaminate local and downstream surface-water samples. Sampling from a dock, boardwalk, or other structure is ideal, but other options such as sampling from watercraft or using a pole extender to collect water samples outside the disturbed area can work as well (Fig. 8b, c).

Porewater and groundwater C pools can be collected less-invasively by installing sample-collection hardware at targeted locations and depths, based on the research question (Fig. 8a). For example, C constituents in water from rooting zones of different vegetation communities can help understand the role of wetland plants on C cycling (e.g., Rey-Sanchez et al. 2019; Bansal et al. 2020; Villa et al. 2020). Other locational considerations include local hydrological conditions such as along topographic gradients, hydrological flow paths, and surface and subsurface geochemical conditions. In wetlands (or locations within wetlands) without ponded water, the depth to the groundwater table can be measured to help to locate the screen positions and length of piezometers or wells to be installed. Vertical distribution of soil hydraulic properties can help identify confining layers in the aquifer (e.g., clay) and improve interpretation of the data.

It is important to confirm that water samples are collected from the correct horizon or source of interest. Suction methods, in particular, have a ‘cone of influence’ around the insertion point of samplers (Fig. 8a). If suction pressure or sample volume is too high, then the cone of influence may extend beyond the zone of interest (e.g., porewater samples can inadvertently be pulled from the overlying surface waters and deeper groundwater). Note that ‘cone of influence’ is a general term and the belowground geometry of the volume influenced by suction methods can be more spherical or cylindrical, with this variation based on the geometry of the sampling device, its depth and orientation, and the negative pressure applied. For example, a suction sampler with a single water inlet at a specific depth below the water table and that collects a small-volume sample is likely to have a roughly spherical volume of influence. When planning a sampling design, it can be useful to estimate the volume of influence based on the expected geometry of the sampling device to be used and the expected sample volume required to meet the proposed analytical needs. The radius of the cone of influence could be estimated from the equation for the volume of a sphere:

(12) $$\frac{V_{S}}{\sigma }=\frac{4}{3}\pi r^{3}$$

where V_S_ is the required sample volume and *σ* is the soil or sediment porosity.

*When:* Point-in-time collection of water samples (referred to as ‘grab samples’) for aquatic C pools is ideally conducted at least monthly to capture seasonal variability, although many studies conduct sampling every two to three months due to logistical constraints. If less frequent sampling is necessary, it may be important to time the sample collection to capture the most significant seasonal changes expected at the study site. For sites that experience seasonality, during snowmelt or storms, physical attenuation or dilution can reduce C concentrations in surface water and porewater. Similarly, seasonal changes in temperature or plant growth patterns can shift the rates and pathways of C transformations within and between the various aqueous C pools described above. Rapid release of CO_2_ to the atmosphere from porewater can also occur in boreal peatlands, primarily during shoulder seasons (e.g., autumn), in response to changes in thermal gradients (Campeau et al. 2021). Meltwater and rainwater also enhance dissolved O_2_ (DO) in surface water and porewater, which influences biogeochemistry and C speciation. Water samples are often collected after pulse perturbations such as intensive precipitation events or anthropogenic disturbances.

In planning when to collect samples, it is important to remember that hardware such as piezometers or water-table wells must be installed before sampling (e.g., up to one month) to allow them to equilibrate with surrounding porewater or groundwater. More time for installation and equilibration may be required in particularly challenging settings. For example, in the Prairie Pothole Region of North America, water does not readily flow into boreholes during excavation because of low-permeability sediments (Winter and Carr 1980), making it difficult to determine the depth of the water table, and, therefore, the depth at which water-table wells should be installed.

*Who:* Experience with wetland hydrology and biogeochemistry is required to select sampling locations, install hardware, and estimate the time needed to equilibrate. Hardware installation involves understanding soil type, porewater formation, and hydrological flow paths. The use of sampling tools and protocols can be explained and demonstrated to general technicians relatively quickly depending on the preferred method.

*How:* The various methodological approaches for sampling wetland water listed below vary depending on the location of water (surface water, porewater, or groundwater) and on the C constituent being measured (dissolved, particulate, or gaseous). Regardless of the exact sampling method deployed, there are many opportunities to introduce contamination during the sample collection and handling (including filtration) processes, and some C constituents are more prone to contamination than others. It is always advisable to collect ‘blanks’ (Type I [ultrapure] or Type II water, possibly with matrix-matching calibration) regularly to assess and ensure sample and data quality. Many C constituents in water can also be measured by in situ automated sensors described below (Section “Radiometric and Stratigraphic Dating - Laboratory Techniques”).

*Sample vessel:* Some vessels are better suited than others depending on the aqueous C constituent of interest. If using bottles to collect samples, a pre-combusted amber glass and/or acid-washed bottle with inert septum (e.g., polytetrafluoroethylene) cap is recommended, particularly for DOC measurements. Some researchers do not recommend plastic containers that may release or absorb organic C, but professional grade, acid-washed, high-density polyethylene bottles can be used if water samples are processed only for DOC concentrations and optical characterization. Because DOC concentration in wetland water is usually high (> 10 mg L^−1^), bottle effects due to absorption and desorption from plastic containers can generally be ignored. If water samples will be used for detailed molecular characterizations, glass containers may be preferred, although glass can break when frozen and therefore acid-washed plastic bottles are also used. Dissolved gas samples require gas-tight vials or syringes; see Section “Dissolved Greenhouse Gases, Dissolved Inorganic Carbon” for more details.

*Surface-water collection:* Water samples can be collected in combusted glass bottles or with syringes. The collection vessel is typically rinsed a few times with the wetland water to be sampled. If collecting water at the top of the water surface, bottles can be submerged or syringes can be inserted directly below the surface. For deeper surface-water collection, plastic (often Tygon or Teflon) tubing can be attached to syringes or a pump and lowered to the desired depth. If using bottles to sample deeper surface water, a rubber stopper attached to string can be placed on the cap of the bottle. The bottle can be lowered to the required sampling depth, and then pulling the string will release the stopper allowing the bottle to fill with sample (Fig. 8b). These methods all disturb the sample during collection by inducing water advection and/or mixing with air. An alternative approach to collect deep water samples that minimizes these issues is to use a commercially available Van Dorn sampler. Van Dorn samplers collect single samples with no advective pressure, and thus the whole water sample is representative of dissolved, particulate, and gaseous concentrations at a specific depth.

*Porewater and groundwater collection:* Collection of dissolved C constituents and ancillary water-quality metrics in porewater and groundwater can be conducted in the field using peepers, piezometers, water-table wells, and suction lysimeters (Fig. 8a), as well as laboratory-based squeezing or centrifugation.

*Peepers:* Sediment peepers (also known as ‘diffusion dialysis samplers’ and ‘diffusion equilibration samplers’) were originally designed by Hesslein (1976) and are commonly used to sample dissolved gases and other soluble diffusible constituents (e.g., DOC) in wetlands (Martens and Klump 1980; Chanton et al. 1989; Schipper and Reddy 1994). In general, peepers are a set of vertically arranged, small chambers with solid walls and one porous membrane (e.g., 0.22 μm pore size) or mesh wall containing a solution of the appropriate salinity or hardness made from deionized or distilled water. This fill water is often deoxygenated (e.g., purged with N_2_ gas) prior to insertion of peepers into the soil to ensure they maintain ambient redox conditions (Fig. 8d). Peepers are inserted to a target depth in sediments and deployed until diffusive exchange leads to equilibrated concentrations of dissolved constituents (gases and soluble compounds) between surrounding interstitial porewater and the peeper fill water. Upon extraction of the peeper, samples can be extracted from each peeper chamber with syringes and stored in capped vials for analysis of C constituents (vacutainers for dissolved gases, anoxic glass vials for dissolved C species). Peepers allow for in situ sample collection from multiple depths, with minimal disturbance, preservation of ambient redox conditions, and without pressure-induced artifacts (Azcue et al. 1996), providing highly resolved and accurate vertical profiles. The disadvantage of peepers is the time necessary for equilibration (hours to a few weeks) and potential membrane breakdown (VanOploo et al. 2008; Villa et al. 2020). Detailed peeper construction, sampling, and handling procedures have been reviewed by Teasdale et al. (1995). Peepers are typically removed from the sediment prior to collecting water samples from each peeper chamber, which risks change to redox conditions. Some designs allow peepers to remain in the sediment during sample collection (Fig. 8d) (MacDonald et al. 2013; Seyfferth et al. 2020).

*Piezometers and water-table wells:* Piezometers and water-table wells allow continuous access for groundwater sampling, and provide a measure of hydraulic head (pressure) that is useful for assessing flow paths and sources (Section “Groundwater Inputs and Exports”). Piezometers are essentially the same as water-table wells, but with a short well screen such that they represent a discrete depth interval within a groundwater flow system. Screen depth intervals typically range from a few centimeters to tens of centimeters. Groundwater samples can be collected at multiple depths by installing nested, multi-level piezometers with the screened interval of each piezometer located at different depths below land surface. It is important to remember that C constituents measured from piezometer samples represent an integrated (averaged) value over the length of the screen interval.

A water-table well is designed with a screen length that is sufficiently long such that the water table, which varies in height over time, is always situated within the screened interval (Fig. 8a) (Sprecher 1993). As with piezometers, C constituents measured from water-table well samples represent an integrated value over the (longer) length of the screen interval, and thus a water sample is representative of a cumulative groundwater source. The distribution of the depths of water-table wells and piezometers are best deployed according to local hydrogeological conditions and C constituents of interest.

For installation of piezometers or water-table wells in wetlands soils, a commonly used method is augering a hole to the targeted depth and then inserting a piezometer or water-table well inside the augered hole. Augering can often be accomplished with a hand auger, although a soil exploration drilling rig (e.g., Giddings drill) or a rotary drill rig may be needed. For easier installation, it is possible to use drive-point piezometers that can be installed directly into the wetland soils, although this is a more expensive and less flexible approach. A challenge with hand augering in wetlands is that the hole being excavated can rapidly fill with sediment and begin to collapse. This challenge can sometimes be circumvented by alternatively driving a PVC pipe into the soil and augering out inside the pipe until the desired depth is reached. When driving a pipe or other object into wetland soils, it is often necessary to have a lever, winch, or other form of mechanical devise to recover that object. It can be useful to know the stratigraphy of the wetland soil in which the piezometer or well is being installed. It is sometimes possible to collect a soil core that is wider than the piezometer or well, and then immediately install the piezometer or well in the hole that is created. The recovered core can then be used for parallel soil C analyses (Section “Carbon in Wetland Soils”).

The annular (ring-shaped) space surrounding screens of piezometers or water-tables well are typically filled with permeable material, commonly sand. Above the screen, the annular space is filled with low-permeability material, such as bentonite or cement, to prevent water from flowing vertically along the outside of the well casing (Lapham et al. 1997; USACE 1998). For water-table wells, non-screened intervals are typically filled either with sand or with the material that was removed from the hole when it was excavated. Piezometer and well installation in shallow soils is relatively easy and low-cost, while installation in deeper subsoil interfaces involves more effort and greater cost.

Since wetlands are relatively flat, elevation is extremely important to measure accurately. Position of all hardware with respect to elevation can be determined using RTK GPS, ideally to accuracies of less than 1 cm to track elevations and representative groundwater sources over time. Tightly fitting casings can be installed inside piezometer and water-table wells to minimize water storage within the well itself, prevent surface-water contamination if the well overtops with surface water (particularly useful in tidal settings), and minimize exposure of groundwater to air (Lapham et al. 1997; USACE 1998; Wilson et al. 2011). Even so, wells and piezometers need an air hole drilled either in the cap or the top of the pipe to allow air to enter and leave as the water level falls or rises.

After installation, the effectiveness of piezometer and well integrity can be checked by pumping water out from the piezometer or water-table well several times over a period of days to weeks, both to ensure ease of flow of water through the well screen and to remove any foreign water introduced during well construction. When multi-level piezometers are installed, their integrities can be checked for each borehole by removing water to decrease water height in one piezometer and measuring changes in height in adjacent piezometers using water level loggers (see below *Key Covariates and Ancillary Measurements*).

Where the water table is less than about 8 to 9 m below the wetland surface, well water can be collected using a syringe or a pump connected to tubing lowered beneath the water level inside the well. It is recommended that non-reactive tubing be acid-washed and rinsed thoroughly with deionized water before use and ideally between sample collections. Stiff tubing can make it easier to reach to the bottom of the well, and clear, gas-impermeable tubing is advisable if collecting dissolved gas samples to visually inspect for the formation of gas bubbles during sampling. To ensure that the collected samples are representative of the depth of interest, it is recommended to pump out 2- to 3-times the volume of water from the piezometer before sample collection. Note that it can take a long time (hours) for groundwater to recharge the piezometer or water-table well if it is installed in a low-permeability substrate. It is sometimes necessary to first pump out the stagnant ground-water from all wells at a site and then return to each well to collect samples. The first collected samples can be used to rinse tubing and bottles prior to sample collection. Triplicate samples should ideally be collected. It is recommended to wash and dry tubing after each sampling trip to avoid biofilm development inside the tubing.

If using a pump, the ‘Low Stress (Low Flow) Purging and Sampling Procedures’ using a bladder pump is an effective method to collect water samples (Puls and Barcelona 1996). Using a bladder pump minimizes changes in the hydraulic potential surrounding the well and avoids generating gas bubbles within the tubing that commonly occurs when water is removed under suction. Peristaltic (suction) pumps can also be used, although degassing, pH modification, and loss of volatile compounds can occur, all contributing to increased error (Puls and Barcelona 1996). Peristaltic pumps cannot be used if pumping groundwater deeper than one atm suction (about 8 to 9 m below ground level). The same restrictions apply to suction sampling by hand using a syringe.

The drop in hydrostatic pressure of groundwater during suction-driven sample collection through a small-diameter pipe or tube can cause spontaneous ebullition of gas bubbles (degassing). This can result in the underestimation of dissolved gases that can be supersaturated with respect to atmospheric equilibrium. Alternatively, when atmospheric concentrations of these gases are greater than dissolved gas concentrations, contamination during sample collection is a concern. Even so, effective suction-driven sample collection for dissolved gas analysis can still be achieved by using low flow rates. This can be confirmed by checking for visible ebullition of dissolved gases in (clear or translucent) sample tubing and sample vessels during sample collection.

Water samples can be collected directly into sample bottles from the bottom up to minimize air contact. When the water is slowly overflowing, the bottle is capped immediately. Alternatively, water can be collected in bottles that are pre-filled with an inert gas (e.g., N_2_) in the headspace to avoid oxidation artifacts. Sample containers are typically placed in an ice-bag or cool-box immediately after collection, and analyzed relatively quickly after returning to the laboratory, or else preserved for longer holding times (sample holding time varies with constituents; see below *Sample Storage*).

*Tension lysimeters and sippers:* Porewater samples from unsaturated sediments can be extracted by installing tension (also call ‘suction’) lysimeters (e.g., Pütz et al. 2018), which operate in a different manner than piezometers or water-table wells. Wells and piezometers extend to the saturated part of the porous media, whereas tension lysimeters are designed to extract water from the unsaturated zone (although they can extend below the water table). A tension lysimeter consists of a porous cup attached to a water collection vessel. The porous cups are typically stainless-steel or ceramic but can be made of other materials (see Weihermüller et al. (2007) for review of materials). These devices can be built or purchased in a wide variety of sizes and configurations to meet specific sampling needs. The porous cups of tension lysimeters are installed to a specific depth. Two tubes typically extend from the cup to land surface. Vacuum is applied through one of the tubes at a constant suction for a specified amount of time to induce porewater to flow through the ceramic cup and into a small vessel to which the cup is attached via the second tube. To avoid clogging, silica flour can be added to the hole before inserting the porous cups to ensure a good connection with the sediment without contaminating or clogging the porous cup. When porewater collection occurs, a tube is lowered into the vessel to suck out the collected porewater, or pressure is applied to push the porewater into a sample-collection container. Other types of tension lysimeters have only a single tube extending to the surface that can be attached to a syringe to apply a vacuum. The syringe slowly fills with sample over time such that the vacuum suction pressure decreases over time. Like piezometers, a nest of multiple lysimeters can be installed to multiple depths. High vertical resolution sampling can be done by drilling small holes at regular depth intervals through the wall of a core tube, inserting a small tension lysimeter (e.g., Rhizon sampler) horizontally into the core through each hole, and extracting the interstitial water. Tension lysimeters may not be appropriate for measuring dissolved GHGs because higher tension can cause degassing and bubble formation (e.g., Pütz et al. 2018). Tension lysimeters that allow for low tension, slow sampling rates, and collect samples through clear tubing and into clear vessels can help to minimize degassing.

‘Sipper’ (also referred to as ‘drive-point sampler’) is a common, generic term that is often used to refer to a device for collecting porewater below the water table. Sippers often refer to stainless steel or plastic pipes (~ 0.5 cm diameter) with holes or slits over a given interval (Fig. 8e). These devices are inserted into the sediment to a desired depth and water can be immediately suctioned out either with a syringe or pump (Fisher and Reddy 2013). A minimal volume of porewater is drawn through the sipper prior to collection to rinse and acclimate the sampling hardware. One advantage of sippers is that they can be made in-house relatively easily or commercially purchased. Another advantage of sippers is the relative ease of placement and collection. However, insertion of the sipper can cause a short-time disturbance of porewater conditions, and the small holes or slits can clog easily depending on the soil type.

*Squeezing and centrifugation:* There are several other methods that can be used to collect porewater or groundwater (Fisher and Reddy 2013). One such method involves collecting sediment cores, dividing them into disc-like sections, and squeezing out the interstitial water using pistons inserted into either end of each section. This method often uses custom-built ‘squeezers’. Porewater can also be extracted using centrifugation, which may require maintenance of oxygen-free conditions (Keimowitz et al. 2016; Dalcin Martins et al. 2017). Squeezing and centrifugation methods may result in much higher C values than other methods like sippers because the centrifugal pressure employed to extract water from both macropores and micropores can also lyse microbial cells and/or force C out of plant tissues, potentially inflating dissolved C concentrations (Gribsholt and Kristensen 2002). These methods are useful if determining attributes such as porewater salinity. Macropores and micropores may also have different C content because anoxic conditions can persevere in micropores longer than macropores, thus limiting aerobic mineralization of SOC (Kechavarzi et al. 2010; Arnold et al. 2015).

*Sample storage:* Preservation opportunities and needs vary for different C pools. DOC, DIC, and dissolved GHG analyses often need to be conducted shortly after sample collection and preparation, as various physical, chemical, and biological processes (e.g., degassing, coagulation, oxidation) could affect the measured concentrations. However, there are multiple ways to extend holding times for different constituents, and they are compound specific. Filtration, for example, is a first step for preserving DOC pools, but it can alter DIC speciation. Similarly, adding preservatives such as mercury chloride (HgCl_2_) are necessary for inhibiting biological activity. Samples can be frozen as well, although this can cause physical disturbances of dissolved C (flocculation) and dissolved gas partitioning. More details on specific storage and preservation requirements for different C constituents is provided below in Sections “Dissolved Greenhouse Gases, Dissolved Inorganic Carbon” and “Total Organic Carbon – Dissolved and Particulate Organic Carbon”. For analysis of DNA and RNA, a − 80 °C freezer is recommended to avoid rapid decomposition (see Section “Wetland Microbiome” for details on storage for microbial analyses).

*Key Covariates and Ancillary Measurements:* There are many variables that can assist in the interpretation of the sources, fate, and dynamics of C constituents in wetland waters. Important variables include water and air temperature, depth of sample collection, and prevailing weather conditions. Many of the site environmental variables can be measured relatively easily under field conditions using multiparameter sondes. A sonde is the traditional instrument that is used in the field as it can hold multiple sensors and log data at the time of sampling or continuously. Field measurements of environmental variables over long period of time can help determine the ideals conditions for water sampling (i.e., the better site conditions are understood, the better the context for the grab sample of water).

*Air and water temperature and weather conditions:* Air and water temperature can be measured in situ with simple probes making sure to avoid exposure to direct sunlight. Air temperature and other weather variables such as humidity/vapor pressure, windspeed, atmospheric pressure, and solar radiation can also be obtained locally using handheld devices (e.g., Kestrel Pocket Weather Meter). Meteorological information can also be obtained from nearby weather stations, although the data should be confirmed as representative of site conditions. In some cases, water-column temperature-depth profiles at high temporal resolution are of interest (e.g., to assess hydrological inversions or mixing of the water column) and can be measured using a series of sensors deployed at multiple depths relative to site bathymetry (Holgerson et al. 2022).

*Precipitation:* Precipitation can fall as rainfall, snowfall, or a mix of the two. Because the hydrology and geochemistry of wetlands is a result of the integrated inputs and losses of all hydrological and associated geochemical fluxes, precipitation is an extremely important variable that should ideally be measured continuously. The only exception could be during winter, when precipitation that typically falls as snow can be considered collectively as a single hydrological and geochemical input during ensuing spring snowmelt. Precipitation is often not distributed uniformly across an area of interest. Therefore, depending on the size of a particular wetland or wetland complex of interest, multiple precipitation gauges may be necessary to estimate the actual volume of precipitation over the surface area. If no gauge is adjacent to or within the wetland catchment, data from a network of more distant precipitation gages can serve as a surrogate through spatial interpolation (e.g., Rosenberry and Hayashi 2013; Dingman 2015).

The simplest measurement method is with a precipitation gauge that collects precipitation and is emptied and recorded manually. However, if the gauge is not conveniently located such that frequent manual observations can be made, preferably on a daily frequency, some of the collected precipitation can be lost to evaporation between observation intervals. Prevention of evaporative water loss for collection of precipitation chemistry is equally important. Therefore, automated precipitation gages often are deployed, commonly in the form of a tipping-bucket or weighing-bucket mechanism (Rosenberry and Hayashi 2013). The tipping-bucket gauge is known to under-measure rainfall during high intensity events, and the weighing-bucket gauge requires more maintenance to ensure accuracy and minimize evaporation from the gauge. Both automated devices, as well as a manual gauge, are subject to under-measurement if adjacent tall vegetation intercepts wind-driven precipitation that otherwise would have fallen in the gauge, or when windblown snowfall (and also rainfall with strong winds) blows across the gauge opening rather than falling into the gauge. Maintenance of an appropriate 45-degree inverted cone of air space above the gage that is free of vegetation will minimize decreased measurement efficiency. Installation of a snow shield can minimize under-capture of snowfall.

*Water depth:* Even for a single grab sample, setting the context is important in relation to distance from water surface and/or from sediment surface. In most wetland settings, surface-water depth can simply be measured manually using a measuring stick or tape at the time of sample collection. Simple staff gauges for rapid repeat measurement are accurate unless bathymetric changes alter surface sediment elevations, and thus apparent water depths. For deep waters, wells, or whenever continuous measurements of water-level fluctuations are required, a water-level datalogger (i.e., pressure transducer) can provide high accuracy depth data. Water-level dataloggers measure the pressure of the water column above the pressure sensor. Water level meters can also be used to measure discrete, rather than continuous, water levels in wells and piezometers.

*pH:* pH is the negative log_10_ of the aqueous hydrogen ion (H^+^) concentration. It affects many aquatic biological and chemical processes including weak acid–base equilibria (e.g., relative abundances of CO_2(aq)_ + H_2_CO_3_/HCO_3_^−^/CO_3_^2−^). Thus, accurate pH measurements are important for the calculation of dissolved CO_2_ or total DIC concentrations from measured alkalinity. Two methods have been well established to measure water pH in aquatic systems, including potentiometric and spectrophotometric methods. A wide range of pH electrodes and sensors are available for in situ potentiometric pH measurements. In principle, such measurements involve detection of electric potential of a sample electrode against that of a reference electrode. Calibration is performed by measuring electrode responses in a series of standard buffer solutions with known pH at a given temperature. The response of pH electrodes may drift over time, requiring regular calibrations. Similar in principle, solid-state selective field effect transistors pH sensors (e.g., Martz et al. 2010; Bresnahan et al. 2014) are available for seawater measurements. Spectrophotometric pH measurements involve adding pH sensitive indicators into the sample water and measuring absorbances at the two wavelengths corresponding to the distinct colors of indicator dissociated basic and acid species, respectively (Clayton and Byrne 1993; Douglas and Byrne 2017). The absorbance ratio at the two wavelengths is a function of the sample pH. Both laboratory-based analyzers and in situ sensors are available for spectrophotometric pH measurements (e.g., Cullison Gray et al. 2011). Spectrophotometric pH measurements are considered to be more accurate and stable than potentiometric measurements and have the benefit of requiring less (sometimes no) calibration. Potentiometric measurements have the advantage of easy deployment/operation and being reagent-free. For any pH measurements, it is important to report temperature and salinity as pH is sensitive to these parameters.

*Salinity/Electrical conductivity:* Salinity generally refers to the dissolved salt content of a waterbody (or soils). For oceanographic studies, salinity is defined by the Practical Salinity Scale (PSS), which is based on the conductivity ratio between a seawater sample and a potassium chloride standard solution (Lewis 1980). Because salinity, in this context, is defined by a ratio, it is technically unitless, but Practical Salinity Units (PSU) are used frequently in the scientific literature. Moreover, the PSS is only defined for salinities between 2 and 42, and is therefore not universally applicable in wetland studies that span the range from fully freshwater to hypersaline. For most wetland studies, therefore, salinity is presented as electrical conductivity or total dissolved solids (see below *Total suspended and dissolved solids*), and the following terms are used interchangeably: salinity, electrical conductivity, specific conductance, and Total Dissolved Solids (TDS). Thus, salinity terminology and units must be evaluated on a study-by-study basis.

Electrical conductivity (as indexed by specific conductance when normalized to 25 °C) indicates the capacity of water to pass electric flow, which is directly affected by ions in TDS, such as salts. Thus, electrical conductivity can be measured in situ continuously and is useful to quantify changes in salt concentration, or salinity. Salinity can vary over time due to changing sources (e.g., seawater, groundwater, wastewater), dilution or evapoconcentration (constituents get concentrated as water evaporates) (Wilson et al. 2015a), direct salt application (e.g., roadsalt, Kaushal et al. 2021), or natural geochemical processes (e.g., pyrite oxidation to SO_4_^2−^, Goldhaber et al. 2014). Beyond physical tracing of flowpaths and residence times, salinity itself is an important controller of C cycling because it strongly influences biological activities such as photosynthesis and transpiration (Ball and Passioura 1995; Krauss et al. 2022a). Further, salinity, as an indicator of SO_4_^2−^ supply from seawater is among the best predictors of CH_4_ emissions in tidal wetlands (Poffenbarger et al. 2011) and inland saline wetlands (Pennock et al. 2010). Salinity is also a necessary covariate in any study involving the measurement of GHG concentrations because it influences the solubility of dissolved gases in water.

Both chloride (Cl^−^) concentrations and specific conductance are typically unaffected by biogeochemical processes and can serve as conservative tracers of water masses. Therefore, they can confirm the origin of a water sample to ensure that sampled water is representative of a specific horizon of interest, which is especially important when using suction methods for sample collection. For example, specific conductance and Cl^−^ concentrations of a porewater sample can be compared to shallower or deeper horizons to confirm that sampled water is from the target horizon. When specific conductance or Cl^−^ concentrations differ between paired samples, it usually indicates dilution by an external water source outside the zone of interest.

Electrical conductivity is reported in microsiemens or millisiemens per centimeter (μS cm^−1^ or mS cm^−1^, respectively). Amperometric probes use two electrodes that pass a fixed voltage between the probes in solution. Potentiometric probes use four-ring probes that pass current and measure the potential drop in current. There are several commercially available electrical conductivity probes. Handheld refractometers can also be used to measure salinity, although their precision and resolution are typically markedly lower than the conductivity probes. Even so, refractometers are robust, simple to use, adequate to get quick field measurements, and are particularly useful in settings where large variations in salinity are expected.

*Total suspended and dissolved solids:* Total Suspended Solids (TSS) are particles in the water column that are generally inorganic materials but can also include plankton, decomposing organic materials, bacteria, and algae. More TSS reduces water clarity. Heavier TSS material can settle on the wetland surface and contribute to sediment accretion (Morris et al. 2012), while lighter, smaller TSS material remains in suspension. TSS can be directly measured by filtering a known volume of water sample through a filter (e.g., 0.20 or 0.45 μm), and weighing the dried filter (mg L^−1^) (Section “Total Organic Carbon – Dissolved and Particulate Organic Carbon”). The organic content of TSS can be used as a measurement of POC. TDS are solids in the filtrate (e.g., salts) from the TSS measurement, which can be weighed following evaporation of the filtrate. TDS include inorganic and organic substances and is often correlated with electrical conductivity or specific conductance, salinity, alkalinity, and hardness (Rhoades 1996). Unlike specific conductance, TDS can be used in transport measurements or load calculations (as calculated in ‘SPAtially Referenced Regression On Watershed’ attributes [SPARROW] models, Smith et al. 1997).

*Turbidity:* Turbidity is a measure of water clarity, which is based on the amount of light scattered by TSS, dyes, colored (or chromophoric) DOM (CDOM), and humic acids. TSS, POC, and turbidity are often, but not always, correlated (Bianchi et al. 1997; Villa et al. 2019a). Short-term increases in turbidity often occur following storm or wind events associated with particulate transport, but are also related to porewater discharge and algal growth in stagnant waters. Turbidity is measured in Nephelometric Turbidity Units (NTU) or Formazin Nephelometric Units (FNU), which are relative terms of light scatter and do not directly indicate TSS. NTU or FNU can be measured using in situ sensors on grab samples or measured continuously using automated sensors. As with other sensors and probes, fouling can occur on turbidity-measurement probes; thus, they require regular cleaning. In addition to automated sensors, turbidity commonly is measured using a Secchi disk, which is lowered into the water until no longer visible to provide a metric of water clarity (Preisendorfer 1986). Once calibrated within specific wetlands, turbidity can be used as a metric of POC (e.g., algal cell) or DOC presence.

*Dissolved oxygen:* DO (mg L^−1^ or % saturation) is a (relatively) easily measured indicator of C cycling processes and thus useful for identifying source and fate of aquatic C pools. Measurement of DO has evolved from time-consuming titrimetric methods (Winkler 1888) to electrochemical microsensors (Revsbech 1989) and optical sensors (Klimant et al. 1995). Rapid development of optical sensors and data-logging capabilities have made stable and affordable autonomous DO sensors available from several manufacturers (e.g., PME miniDOT). This has allowed new lines of hydrological and ecological research and enhanced the precision of traditional estimates of whole-system metabolic rates, respiration and photosynthesis, and net ecosystem metabolism. For example, studies have shown that metabolic assessments of lentic ecosystems can be vastly improved by deploying multiple autonomous O_2_ sensors in strategically targeted locations (Staehr et al. 2010; Van de Bogert et al. 2012; Schilder et al. 2013). Also, the development of fast-responding micro-sensors has allowed eddy covariance measurements of O_2_ fluxes underwater, which through stoichiometry can be linked to C fluxes and metabolism (Section “Eddy Covariance”) (Berg et al. 2003, 2022).

*Light levels:* Light, specifically photosynthetically active radiation (PAR), measured in units of photosynthetic photon flux density (PPFD, units of μmol m^−2^ s^−1^), is an important driver of C processes in surface water because of its direct effects on biological, chemical, and physical processes (e.g., photosynthesis, photodegradation, and stratification, respectively). There are several commercially available sensors designed for underwater PAR measurements (e.g., Apogee Full-Spectrum Quantum Sensor; LI-COR LI-192 Underwater Quantum Sensor). Other sensors measure lux (measured in lumens m^−2^) that can be converted to watts m^−2^ (e.g., HOBO Pendant Temperature/Light Data Logger), which can be roughly calibrated to PPFD using a PAR sensor. Decreasing light levels along depth profiles to calculate light extinction coefficients are often used for metabolism studies and can be measured by placing sensors on an extension rod for grab samples or along a chain for continuous data.

*Chlorophyll:* As a key molecule in photosynthetic activity, aquatic pools of chlorophyll (especially Chl-*a*) can be used to quantify algal C pools, and/or identify rates of photosynthetic inputs with grab samples or in situ sensors. High water column concentrations of Chl-*a* are associated with high nutrient concentrations (N and P) and nuisance algal blooms. While C pools may only be temporarily enhanced by an algal bloom, algal dynamics affect a number of C-related biogeochemical processes (e.g., CH_4_ production, León-Palmero et al. 2020), as well as water quality, including toxin production (Binding et al. 2013; Reid et al. 2019). Measuring chlorophyll concentrations with high accuracy involves collection of water samples, immediate dark filtration and light-free preservation at low temperatures (− 80 °C needed to preserve cells and Chl-*a* before it degrades to phaeophytin), rupturing of cells, extraction of chlorophyll, and then analysis with a spectrometer of fluorometer (Reeder and Binion 2001). While this laboratory-based method is the most accurate for measuring chlorophyll species, it is time consuming and requires a high level of expertise and costly equipment. Chl-*a* (the most common form of chlorophyll) is often measured in situ using fluorescence probes (e.g., YSI 6025). Probes emit light at 470 nm that Chl-*a* containing cells absorb and re-radiate at 650 to 700 nm, which is measured by probe photodetectors and converted to Chl-*a* concentrations (ug L^−1^). Blue-green algae probes are also available with a different fluorescence response. Fouling of spectral probes, especially through algal growth, necessitates periodic manual or autonomous cleaning by physically wiping sensory ports or through ultraviolet degradation of surface films. Due to the spectral signature of Chl-*a* (i.e., it has high absorbance in blue and red wavelengths and strong reflection of near-infrared wavelengths), algal C pools in water can also be estimated using passive optical remote sensing in calibrated models (Cannizzaro and Carder 2006; Binding et al. 2013).

*Upscaling to the wetland:* For studies aiming to describe C pools in water at the whole-wetland scale, information on wetland water depth, volume, and/or surface area are needed in addition to concentrations (e.g., mg L^−1^, μg L^−1^) of C constituents measured discretely or with sensors.

A common method for scaling C in wetland water is to use the relationships between depth, volume, and surface area (e.g., Gleason and Tangen 2008; Tangen and Finocchiaro 2017; McKenna et al. 2018). Development of depth–volume–area relationships (referred to as ‘curves’) requires data from detailed topographic (e.g., Gleason and Tangen 2008; Tangen and Finocchiaro 2017) and/or hydrographic/bathymetric surveys (Densmore et al. 2013; Stateczny et al. 2021), which are then used to create digital elevation models (DEM). DEMs also can be developed using remotely sensed data (Section “Remote Sensing”). Once DEMs have been created, water volumes and surface areas can be determined for various depths (depth from sediment surface), and these data are used to develop statistical depth–volume–surface area curves that can either be generalized (e.g., Gleason and Tangen 2008) or site-specific (Tangen et al. 2013; Tangen and Finocchiaro 2017). Water depth measured during water sampling, whether from manual measurements or data loggers (e.g., pressure transducers), can then be used to estimate water volume for the scaling of C constituents in wetland water. The volume of water in the near-surface (e.g., upper 1 m) porewater can also be included in the wetland water volume through determination of soil porosity.

For tidal wetlands, water depth (or height) and volume of water exchanged can be measured, which are then used to determine the wetland surface extent that the water covers (Bergamaschi et al. 2011). Corrections can also be applied to account for soil characteristics (moisture content, porosity). Additionally, wetland drainage can be characterized using water routing analyses applied to a DEM (Wang et al. 2016). A study that used both the height-volume change and the routing analysis techniques at a brackish tidal marsh in California (USA) obtained strong agreement between techniques in estimates of wetland extent (Bogard et al. 2020a).

Estimates of tidal inundation area can be complicated by multiple factors. The volume of tidal exchange, and thus wetland footprints, vary through time with tidal cycles. Moreover, the exchange of water entering and leaving the wetland at the point of measurement in adjacent creek channels is often imbalanced due to overland runoff, evaporation, and other potential factors (Bergamaschi et al. 2011; Wang et al. 2016; Bogard et al. 2020a).

An important consideration is that the concentrations of water constituents are temporally variable owing to biological activity, water and solute inputs and losses, as well as to concentration and dilution associated with factors such as evaporation, precipitation, and surface flow. Therefore, repeated measures over time can provide a more wholistic representation of the C pool in wetland waters. When channelized, flowing water volumes are of interest (e.g., lateral flux), stage-discharge curves can similarly be used to estimate water volumes of lotic systems (e.g., Carey 2003; Cook et al. 2018b).

#### Dissolved Greenhouse Gases, Dissolved Inorganic Carbon

*What:* Accurate determination of DIC and dissolved GHGs (CO_2_, CH_4_, N_2_O) is important for understanding drivers of GHG emissions from wetland waters. Data on the concentration of these constituents can be central to studies ranging from individual processes to overall ecosystem functioning, including total wetland C and GHG balance estimates (Elberling et al. 2013; Trifunovic et al. 2020). Although recent technological advances allow continuous GHG measurements with various in situ sensors (Section “In situ Sensors and Analyzers”), manual water sampling is still needed for sensor calibration, profile assessments, and initial site characterization. Moreover, in situ sensors may not be available to all researchers and many research questions can be adequately answered through a well-designed grab sampling campaign. For sampling of dissolved pools of GHGs, headspace equilibration is commonly used. The gas of interest is equilibrated between known volumes of sample water and headspace gas within an airtight bottle, syringe, or vial, and the concentration of the analyte in the headspace gas is then determined. The original concentration of the dissolved gas in water can be calculated. DIC can be similarly determined by acidifying the water sample.

*Where:* The locations to collect water samples in the field are described in the previous section on water sample collection (3.B.i). If using the headspace equilibration method, equilibration of water samples with headspace gas is ideally performed in the field to avoid alteration from field conditions. Analysis of the headspace gas is typically conducted in a laboratory equipped with a gas chromatograph (see Section “Chamber Measurements” for details on gas chromatographs). Laboratory based gas chromatographs require bench space and often cylinders of multiple compressed gases.

*When:* Isolation of dissolved gases and DIC from the water sample is ideally conducted quickly to prevent changes in concentrations associated with microbial activity that can occur within hours of collection. To preserve samples for longer periods, method-specific additives can be used. For the acidified headspace method, the addition of concentrated acid typically lowers the sample pH to ~ 2. Although the acidic conditions slow down many of the biological processes, Åberg and Wallin (2014) found that respiration processes continue at low pH (although at low rates) and recommended analysis within three days upon sampling. There are also examples of adjusting the pH to ~ 11 to prevent CH_4_ oxidation in the water samples prior to analysis (Bastviken et al. 2002). Another common way of preserving samples is to halt microbial activity by adding a toxic amount of highly concentrated HgCl_2_ or zinc chloride (ZnCl_2_) (Cane and Clark 1999; Campeau et al. 2017).

Leakage of gas in or out of the sample container is an issue of concern when storing gas samples prior to analysis (Magen et al. 2014). Storage testing of a given method (including standards and spike recovery) is always recommended in order to understand the method-specific time constraints. Using grease-sealed, high-density, nylon syringes helps increase the storage time for extracted gas. Another storage method is to collect gas samples in gas-tight evacuated containers such as Vacutainers, Exetainers, or headspace vials sealed with butyl rubber septa and aluminum crimp caps. Adding a small amount of highly concentrated sodium chloride (NaCl) solution, in which GHGs have low solubility, can provide extra protection against gas leakage. Storing the container upside down allows the NaCl solution to provide a barrier between the gas phase and the septum. If liquid or gas samples are collected and stored for later dissolved gas analysis, gas leakage is less if vials are stored upside down with the cap submerged in water.

*Who:* Samples for determination of DIC and dissolved GHG concentrations are often more complex and challenging to collect, store, and transport than samples for many other aqueous constituents (e.g., DOC) as samples can quickly be affected through interaction with the atmosphere. Training and adherence to a specific protocol is needed to ensure consistent sampling by multiple personnel.

*How:* Various types of headspace and CO_2_ extraction methods are commonly used for determination of DIC and GHG concentrations in water. Below are examples of frequently used methods.

**Fig. 9:**
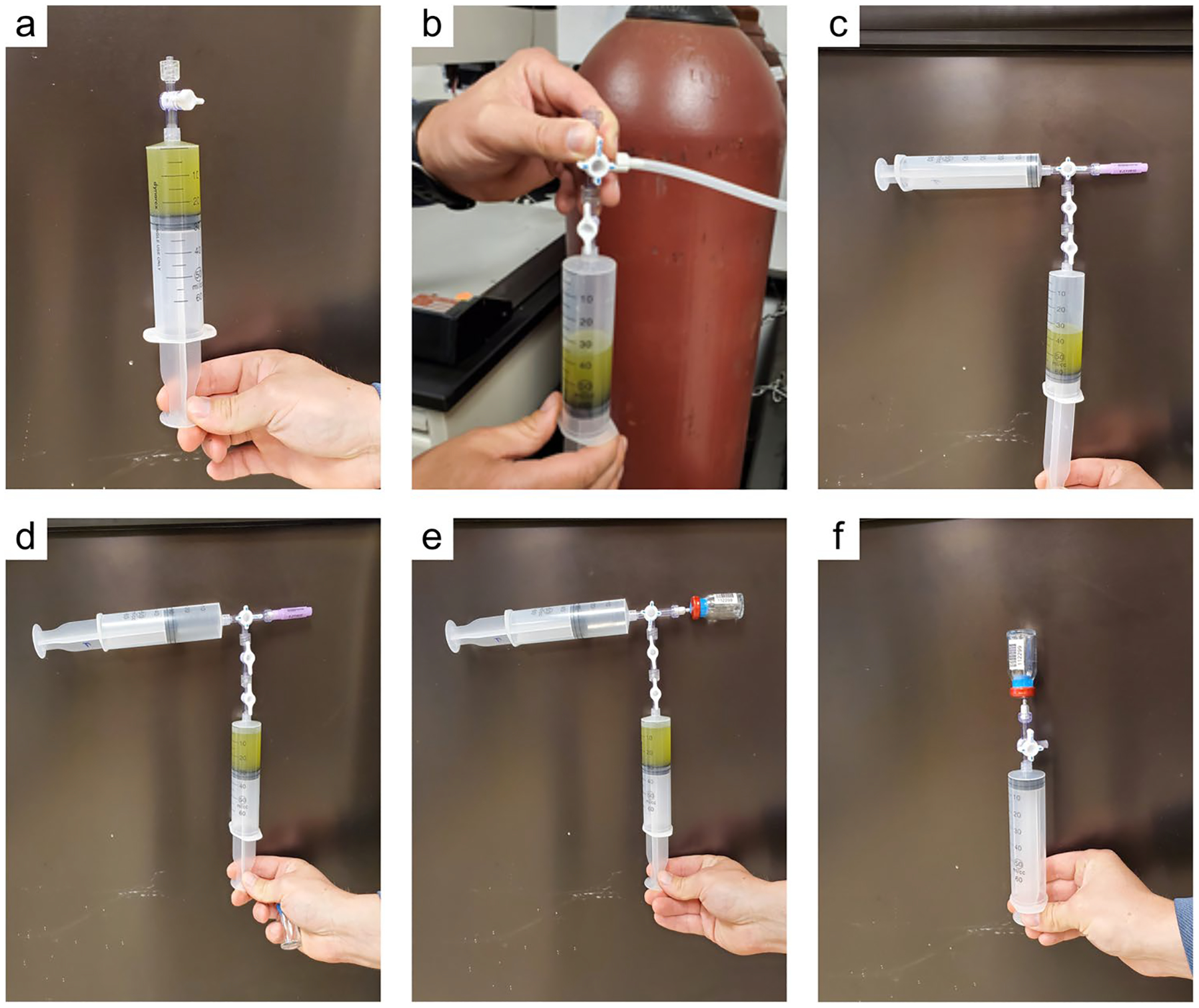
One example sequence of direct headspace determination through equilibration. (**a**) Fill a syringe with 25 mL of sample water; (**b**) add 35 mL of analyte-free inert gas (e.g., N_2_ or He) to syringe and then shake for 3 to 5 min by hand or mechanical shaker (not shown); (**c**) connect sample syringe to an empty syringe with an attached needle (steps c and d and the extra 1-way valves are to limit moisture entering the vial and may not be required depending on gas analyzer); (**d**) transfer headspace gas to the empty syringe; (**e**) insert the needle into a vacuum-evacuated crimptop serum vial with butyl stopper; and (**f**) push the headspace gas into the vial. Both syringes have Luer Lock tips for connecting 1-way or 3-way stopcock valves. Care should be taken to ensure valves are oriented correctly at each step to avoid loss or contamination of sample. Images with permission from Sheel Bansal

*Headspace equilibration method:* A common technique for collecting GHG samples is the headspace equilibration method (also referred to as ‘direct headspace determination’). There are several protocols for carrying out headspace determination, but they all involve equilibrating an aqueous sample with an analyte-free gas such as N_2_ or He (typically at 1:1–1:3 v/v ratios, Jahangir et al. 2012) within a closed system such as a gas-tight bottle, vial, or syringe (Fig. 9). Note that ambient air can be used for the direct headspace determination if analyte-free gas is not available and anticipated dissolved gas concentrations are substantially greater than atmospheric concentrations. Equilibration of analyte gas between the water sample and headspace gas is accomplished by vigorous shaking for at least 3 to 5 min either by hand or a mechanical shaker at approximately 400 rpm (McAuliffe 1971; Kling et al. 1991). Samples are ideally allowed to stand for 30 min before a portion of the headspace gas is extracted into exetainers/vacutainers/syringes/vials for analysis on a gas chromatograph. Alternatively, a water sample may be injected directly into a sealed, N_2_-purged vial such that the equilibration occurs in the vial, and then the headspace gas can be sampled directly for analysis. GHG concentrations in the headspace gas can be determined by gas chromatography or gas analyzers based on absorption spectroscopy technology (Section “Chamber Measurements”).

The concentration of the dissolved gas of interest in the water sample is calculated by dividing the total amount of gas that partitioned between the water sample and headspace gas by the volume of the water sample. The amount of GHG in the headspace is calculated from the concentration of the GHG in the headspace and the headspace pressure, volume, and temperature. The amount of gas remaining in the water sample is determined by Henry’s law (Eq. 11), the partial pressure of the GHG in the headspace, and the volume of the water sample. See Magen et al. (2014) for an example of these detailed calculations and Sander (2015) for gas-specific Henry’s constants. Note, the symbol used for Henry’s constant is inconsistent among studies including: K_Hx_, *K*_WA_, *K*_AW_, and *k*_H_ (Sander et al. 2022).

*Acidified headspace method:* DIC can be determined by the acidified headspace method (Stainton 1973; Wallin et al. 2010), which is similar to the direct headspace determination method except that the water sample is acidified to a pH of approximately 2 with a small volume of a concentrated acid (e.g., 37% HCl, 85% H_2_PO_4_^−^), which converts all carbonate species to CO_2_ (Fig. 7b). The acid can be added prior to sampling or post sampling through the septum/stopper with a needle or via a 3-way stopcock. The concentration of CO_2_ in the headspace is then determined and used to calculate the total amount of DIC that was in the water sample. The concentration of dissolved CO_2_ in the sampled water can be calculated from the total DIC concentration, in situ pH, water temperature, and carbonate system equilibrium equations (see Wallin et al. 2010).

*Acidified CO_2_ extraction method:* CO_2_ extraction after sample acidification using an inert carrier gas such as N_2_ has been used for DIC measurements in both seawater and freshwater (Wang and Cai 2004; Dickson et al. 2007; Wang et al. 2013b). In this method, the water sample is first acidified with relatively low concentration acid of similar ionic strength to the sample (e.g., a 10% phosphoric acid in 0.7 M NaCl solution for measurements of seawater samples). Sample CO_2_ is extracted by purging high purity N_2_ (a carrier gas) through the acidified sample to a CO_2_ detector for measurement of total CO_2_ in the acidified sample (equivalent to total DIC). Commonly used CO_2_ detectors or analyzers include the nondispersive infrared CO_2_ analyzer (e.g., LI-COR infrared CO_2_ analyzer) and the coulometer (e.g., UIC CO_2_ coulometer). A certified reference material is often used to calibrate the gas analyzer regularly (e.g., at least once daily) (Wang and Cai 2004).

*Indirect CO_2_ determination:* Due to the limited amount of existing headspace-based datasets, dissolved CO_2_ concentrations are often estimated from alkalinity concentrations that are determined by titrimetric methods (Neal 1988; Humborg et al. 2010; Butman and Raymond 2011). This approach typically assumes that the sole contributors to measured alkalinity are carbonate species (CO_3_^2−^ and HCO_3_^−^) and uses carbonate system equilibrium equations to calculate dissolved CO_2_ from alkalinity concentration and in situ pH and water temperature. Although this assumption is suitable for some systems, it can generate erroneous estimates of dissolved CO_2_ concentrations in systems with low alkalinity, low pH, and/or relatively high concentrations of non-carbonate/bicarbonate alkalinity (Wang et al. 2013a; Wallin et al. 2014; Abril et al. 2015; Song et al. 2020b). For example, high DOC concentrations can contribute substantially to alkalinity, which frequently occur in wetland systems (Abril et al. 2015).

*Key Covariates and Ancillary Measurements:* Many of the additional measurements that are useful for interpreting GHG and DIC concentrations are described previously in Sections “Water Sample Collection – Surface Water, Porewater, Groundwater” and “Total Organic Carbon – Dissolved and Particulate Organic Carbon”. In particular, in situ, high accuracy pH, water temperature, air pressure, and salinity data are needed to perform calculations of dissolved gas concentrations. Dissolved GHG and DIC concentrations can be paired with continuous, sensor-based observations of environmental parameters (e.g., water temperature, stage, pH) to build regression models that estimate DIC or CO_2_ concentrations at high-resolution time intervals (Wang et al. 2016; Chu et al. 2018; Bogard et al. 2020a).

#### Total Organic Carbon – Dissolved and Particulate Organic Carbon

**Fig. 10:**
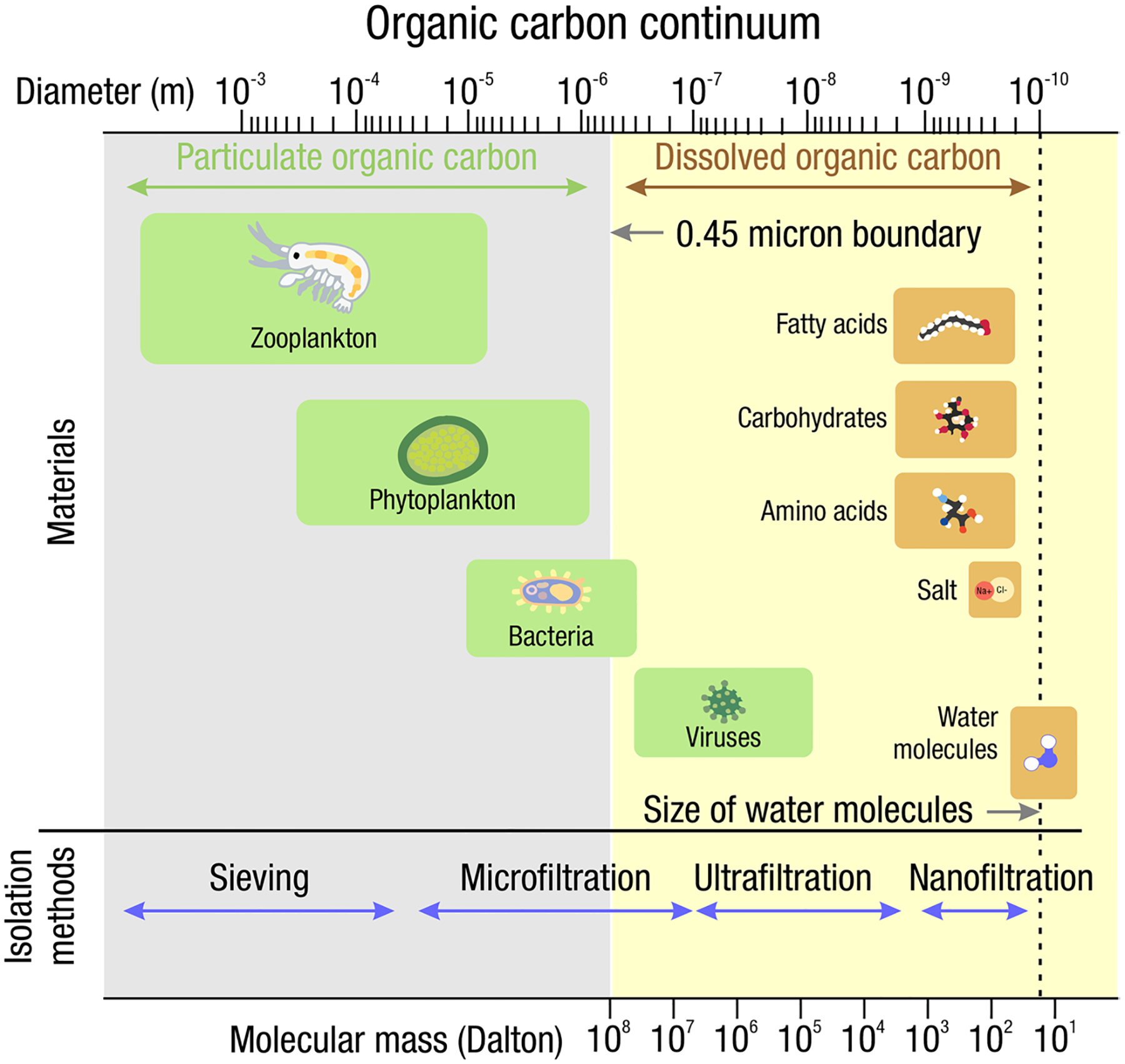
Continuum of organic carbon in water (based on Thurman 1985 and modified from Chow et al. 2022). Particulate (gray shading) and dissolved (yellow shading) organic carbon are defined as greater or less than 0.45 μm (micron) size, respectively. The types of organisms and molecular compounds span a range of sizes. Methods to isolate different materials require different filtration methods. Image with permission from Kelly Wing-Yee Cheah

*What:* Organic C in water, which is usually reported as TOC, represents a variety of natural organic materials in aquatic ecosystems, from nanometer-scale organic molecules such as proteins and carbohydrates to millimeter-scale organic debris degraded from plants and animals, as well as living microorganisms such as zooplankton and phytoplankton (Fig. 10) (Thurman 1985; Chow et al. 2022). Traditionally, TOC is operationally separated into DOC and POC based on the cutoff point at 0.45 μm pore size filter (although 0.70 μm or 0.20 μm are also used frequently). It is important to isolate different fractions in water because they behave differently in terms of hydrological transport and biogeochemical processes (Chow et al. 2006; Battin et al. 2008; Worrall and Moody 2014). In addition, DOC can further be separated into different molecular weight fractions using ultrafiltration (e.g., 1, 5, 10, 30 kilodalton, depending on the nominal molecular weight cutoff membranes) (Tadanier et al. 2000) or hydrophobic/hydrophilic fractions using chromatography resins (Leenheer and Croué 2003; Chow et al. 2005). The most common and accepted method for TOC analysis is to quantify the total CO_2_ emitted after complete oxidation through catalytic combustion or wet chemical oxidation using a TOC analyzer (USEPA 2004; Chow et al. 2022).

There are some shortcomings and sources of potential error that researchers should be aware of when measuring TOC, DOC, and POC. DOC and POC pools can interconvert in the water column through different biogeochemical processes such as coagulation (flocculation), precipitation, and photo- and bio-degradation over time. Their concentrations can be affected by the sampling protocols and storage time (Spencer et al. 2007; Nimptsch et al. 2014; Heinz and Zak 2018). Also, the definitions of DOC and POC can be different among research teams and articles (Chow et al. 2022), so researchers should pay careful attention to these definitions before comparing data among studies.

*Where:* Commercial TOC analyzers are bench-top instruments that usually require a 220 V power supply. Cylinders of compressed air or O_2_ are needed as combustion gas. The analytical procedure also requires use of dilute acid (usually HCl) to eliminate inorganic C in water. Therefore, TOC analysis can only be conducted in a laboratory with corresponding safety features. Also, a laboratory equipped with Type I or II deionized water and a filtration system using either pressure or vacuum is recommended.

*When:* Samples are typically filtered within 48 h following collection and then acidified to pH ~ 2 (usually 2 μL of concentrated HCl for 100-mL sample) to inhibit microbial activity (Potter and Wimsatt 2009). The filtered, acidified samples are typically stored at ≤ 4 °C and analyzed within 28 days. In some cases, precipitation of humic acid has been observed following acidification (Spencer et al. 2007). If any precipitation or cloudy suspended mixture is observed after storage, those affected samples should not be used for DOC quantification. Several studies demonstrated that significant microbial degradation of organic matter can occur in samples stored at 4 °C, especially if samples contained significant biodegradable organic C or nutrient-rich water (Kaplan 1994; Peacock et al. 2015; Nachimuthu et al. 2020). If the acidification approach is not feasible (e.g., if there is need to determine the inorganic C fraction in the samples), Åberg and Wallin (2014) recommended that analysis be performed within three days of sampling. Freezing water samples at − 20 °C or lower has been suggested for long-term storage, although DOC composition can change after freezing–thawing due to lysis of microorganisms, flocculation, or formation of precipitates (Spencer et al. 2007; Fellman et al. 2008; Peacock et al. 2015; Heinz and Zak 2018).

*Who:* To analyze organic C in water, technicians should have some education or experience in aquatic chemistry. The TOC operator also needs to receive training in filtration, dilution, instrumentation, and laboratory safety (e.g., working with pressuring systems, acids and bases, and high temperature devices). Protective equipment such as gloves, laboratory coat, and safety goggles are needed.

*How:* To determine TOC concentration, unfiltered or 1.5-μm filtered samples are analyzed for C using LOI or a CHN analyzer. POC can be determined by the difference between TOC and DOC (POC = TOC − DOC) (Chow et al. 2005). POC can also be quantified by determining the C content remaining on a filter paper following filtration for DOC (Cambardella and Elliott 1992). The filter membrane is dried in an oven at 65 °C or lower (to minimize oxidation) and ground (often with a ball mill grinder) to homogenize the filter. However, the potential error of this approach may be relatively large due to the loss of mass on the filter membrane and apparatus during the drying and transferring processes.

*Choice of filter:* For dissolved constituents, water filtration is a critical first step. Water samples collected from natural environments contain a variety of particles, sediments, and debris (Fig. 10). Filtering water samples before organic C analysis is generally needed in order to curb biologic activity and remove larger particles that can potentially block and damage the tubing or syringes of the TOC analyzer. A glass microfiber filter with 1.5-μm pore size, which is used for total suspended solid analysis (USEPA 1999), can be used for measuring TOC and as a pre-filter prior to the DOC filtration. The 1.5-μm filtrate is used in the TOC analyzer. Although some of the commercial TOC instruments can analyze large particles up to 10 μm (i.e., in unfiltered samples), large particles can settle at the bottom of the vials unless a stirring system is included in the autosampler. Also, incomplete oxidation of large particulates can affect accurate determination of TOC in water (Aiken et al. 2002).

A filter with a pore size of 0.45 μm is conventionally used for differentiating DOC and POC, although smaller and larger pore sizes have been used in many studies (Nimptsch et al. 2014; Chow et al. 2022). The 0.45-μm or smaller pore size filter is made of organic polymers which can release or absorb organic C to or from water (Yoro et al. 1999; Karanfil et al. 2003; Nimptsch et al. 2014). Among commercially available membrane materials, polyethersulfone has the least extractable organic C. Washing with 500 mL or more of deionized water is recommended to minimize C contamination if a 47-mm diameter disc filter is used (Karanfil et al. 2003). The 0.2-μm or smaller pore size filter is preferred if water samples contain high levels of colloids (e.g., from soil porewater) or nutrients (Chow et al. 2005). Microorganisms including bacteria can be filtered from water when a 0.2-μm or smaller pore size filter is used. However, because of its small pore size, filtration time can be longer and require more frequent filter replacement. Rinsing the filter with the first few mL of water sample before filtering the remaining sample volume can help to minimize filter-derived contamination. A robust plan for collecting frequent field blanks is the best way to isolate and quantify any contamination issues and to determine the actual detection limits for the entire sampling and analysis procedure used. A variety of blank types may be needed to assess different procedure steps and pieces of equipment.

*Filtering Protocols:* Samples are typically stored in a cool-box filled with ice (or fresh wetland) water immediately after collection in the field and then transferred to a 4 °C refrigerator once returning to a laboratory. A 47-mm diameter glass filtration set with a pressurized or vacuum system is generally needed for filtering sufficient liquid volume for DOC and other water analyses. Using a plastic or ceramic filtration apparatus is not recommended because these materials can release or absorb organic C from water samples. All non-volumetric glassware can be heated in a muffle furnace at 425 °C for 2 h to eliminate residual organic contaminants before use (USEPA 2004). In addition, membrane discs are washed with at least 500 mL deionized water (Type I or II water) and the first ~ 25 mL of filtered sample is typically discarded (Karanfil et al. 2003). It is highly recommended that water is filtered through a 1.5-μm glass fiber filter as a prefilter prior to 0.45-μm filtration. A new, pre-rinsed filter should be replaced as soon as any visible water accumulates on top of the filters to avoid filter fouling; that is, ‘filtration cake’ can form on the membrane surface altering the pore size of the filters. One hundred milliliters of water should pass through the membrane filter within 1 to 2 min under pressure or vacuum. Syringe filters (usually 33 mm in diameter) can alternatively be used, but each syringe filter can only process 10 to 20 mL of water before clogging (and sometimes much less depending on the quality of the water being filtered). Syringe filters are most appropriate for small volume (a few milliliters to tens of milliliters) porewater sampling applications.

*TOC Analyzer:* TOC concentrations in water samples are determined by measuring the total CO_2_ produced by oxidation of organic C in water. There are two oxidation methods that are commonly used and commercially available: 1) ultraviolet-promoted persulfate wet oxidation; and 2) high temperature catalytic dry combustion (USEPA 2004). Ultraviolet-promoted persulfate wet oxidation generally has a lower detection limit (i.e., down to 2 μg L^−1^ of C) than high temperature catalytic dry combustion, but may not be suitable for marine or estuary water samples with a high salt content (McKenna and Doering 1995). High temperature catalytic dry combustion is more commonly used because the instrument can process different water matrices (including wastewater and seawater) and a wide range of DOC levels, from 0.05 mg L^−1^ to 30,000 mg L^−1^ of C. Prior to injecting water samples to the reactor, samples need to be acidified and bubbled with air to purge inorganic C and volatile organic C from samples. These two steps are often performed within the instrument and the remaining organic C is referred to as non-purgeable organic C. The CO_2_ produced from oxidation of the non-purgeable organic C is typically quantified by non-dispersive infrared absorption. Potassium hydrogen phthalate is commonly used as a standard for DOC because it is highly soluble and contains aromatic ring and carboxylate groups, which are essential functional moieties of natural organic matter. Spike/recovery tests can also be conducted to assess accuracy of the method, where a known amount of standard is ‘spiked’ into a sample and the measurement, or ‘recovery’, of spiked material is assessed. Calibration curves, using a standard solution, can be obtained by automatic serial dilution with Type I or Type II deionized water (e.g., 1:2, 1:4, and 1:8). If spiked or diluted standards are too far below or above expected values, then it is possible that some constituent or reaction in the sample matrix is causing depressed or elevated recovery of the sample. For quality assurance/quality control, standards and blanks are placed between samples (USEPA 2004).

*Key Covariates and Ancillary Measurements:* Concentrations and compositions of TOC, DOC, and POC are highly variable with environmental conditions. General water-quality parameters such as pH, specific conductance, total N, and total P are typically determined for the same samples because these parameters could affect the solubility and degradability of certain DOC fractions (Zsolnay 2003). Also, hydrology is an important factor affecting TOC concentrations. Concentration and composition of TOC can vary significantly along a hydrograph after a storm event (Majidzadeh et al. 2017; Uzun et al. 2020). Researchers should pay attention to the hydrological conditions such as water inputs, water flow rates, groundwater mixing, etc. (Section “Water Sample Collection - Surface Water, Porewater, Groundwater”).

*Absorbance and fluorescence:* Spectral measurements and indices, such as Ultraviolet A (UVA) absorption at 254 nm (A_254_) of filtered water (usually 0.45 μm pore size), are helpful to understand the source and fate of DOC compounds. Specific UVA (SUVA) is equal to the UVA normalized to DOC concentration (SUVA (L mg^−1^ C^−1^ m^−1^) = UVA (cm^−1^) / DOC (mg C L^−1^) × 100). SUVA has been widely used as a surrogate of aromatic C content (aromaticity) of DOC (Weishaar et al. 2003; Chow et al. 2008). Other wavelengths such as 272 nm, the ratio of 265 to 365 nm, and the spectral slopes of 275 to 295 nm and 350 to 400 nm are also measured and serve as surrogates of molecular weight and other characteristics of CDOM (Helms et al. 2008). Fluorescence spectroscopy can characterize different Fluorescent DOM (FDOM) fractions and components including quinone-like moieties as well as humic-acid like and fulvic-acid fractions (Cory and McKnight 2005; Zhou et al. 2013). Wetland DOC absorbance and fluorescence measurements are often complicated by the presence of Fe, Fe^3+^, and Fe^2+^, necessitating correction or extraction (Poulin et al. 2014).

*Radiocarbon dating:*Carbon-14 (^14^C) dating of DOC can provide information as to whether the C is sourced from ‘old’ or ‘young’ organic matter. For methods on radiometric dating, see Section “Radiometric and Stratigraphic Dating - Laboratory Techniques”.

#### In situ Sensors and Analyzers

**Fig. 11:**
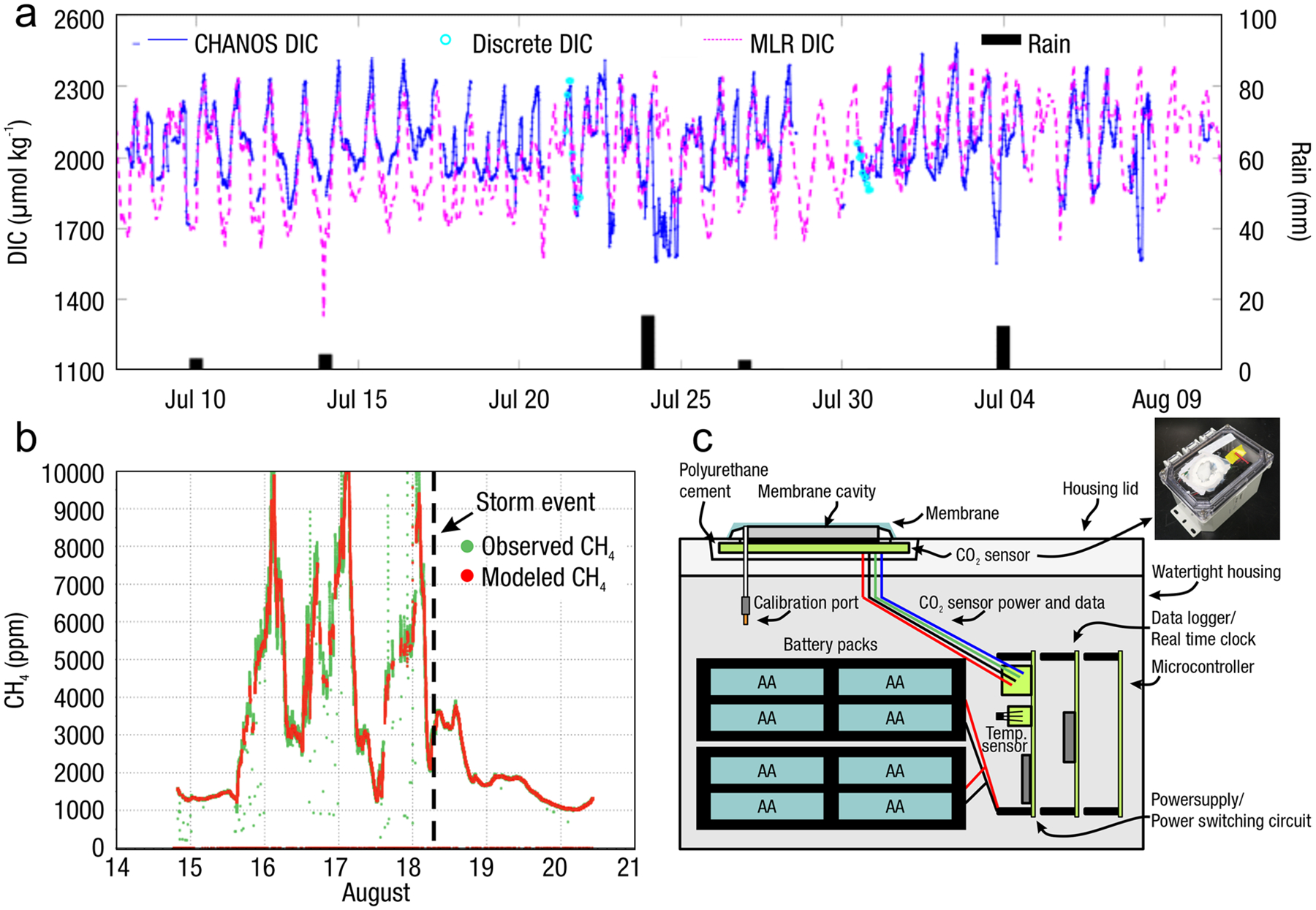
Examples of high-frequency in situ sensor data and equipment: (**a**) time series of dissolved inorganic carbon (DIC, blue dots and line) concentrations measured using a Channelized Optical System (CHANOS) at the tidal creek of the Sage Lot Pond marsh, Waquoit Bay, Massachusetts, USA; discrete samples of laboratory measured DIC concentrations (cyan circles) were used to validate CHANOS DIC (Wang et al. 2015); a multi-linear regression model of DIC (MLR DIC) estimates (pink, dashed line) concentrations (Chu et al. 2018); precipitation amount (mm, black bars); (**b**) time series of measured (green dots) and modeled (red dots and line) dissolved methane (CH_4_) concentrations using a Membrane Inlet Mass Spectrometry (MIMS) showing disruption of daily patterns following a storm event in a wetland in the Prairie Pothole Region of North America; (**c**) schematic diagram and picture of a partial pressure of carbon dioxide (*p*CO_2_) sensor (Haase and Sanford 2018). Images with permission from Zhaohui Aleck Wang (a), Christopher Martins (b), and Karl Haase (c)

*What:* Manual sample collection of wetland waters for measurements of C concentrations may not represent averages and are likely biased by logistics (e.g., daytime sample collection). Therefore, data with high spatial and temporal resolution should provide less biased estimates of averages. In situ sensors not only provide information on pools of C in wetland waters but also changes in pools over time. In situ sensors and analyzers for measurements of *p*CO_2_, *p*CH_4_, DIC, CDOM, and FDOM are either commercially available or available as research prototypes (*circa* 2023). In situ systems substantially increase temporal coverage of measured parameters compared to discrete grab measurements. Their measurement frequencies vary from seconds (near instantaneous) to tens of minutes, hours, or days, which can then be averaged over time (Fig. 11). Depending on the sensors or analyzers, occasional grab samples for bench analysis (as described above for discrete water sampling) may be needed to verify or calibrate in situ measurements. Of further consideration, detection limits are generally higher than laboratory-based protocols and may not be low enough for field measurements. Biological, chemical, and physical fouling is a major issue for in situ deployment in many wetland systems. Some sensors or analyzers may have built-in anti-fouling mechanisms, such as wipers, ultraviolet irradiation, in situ water filters, anti-fouling paint, and copper or other metal alloy meshes.

*Where:* Locations for deployment of in situ sensors and analyzers depend on sampling purposes and research questions, as well as the capability, maintenance, and logistical requirements of the devices. In general, these systems can be deployed at places based on the same considerations for discrete manual samples, with the added consideration of access to a suitable power supply and accessibility to the deployment sites for scheduled maintenance of the sensors or analyzers.

*When:* Depending on deployment purposes, in situ sensors or analyzers can be deployed at most times of a year. Measurement frequency is an important parameter for deployment. The general rule is to maximize measurement frequency while considering the constraints of power requirement, data storage capacity, and other limiting factors. Power requirement of the device is an especially important consideration for establishing deployment and revisit intervals. Many devices can run on battery power. However, if the deployment is longer than batteries can sustain power, alternate power sources such as solar, wind, or line power will be needed. Regular maintenance at suggested time-intervals is often recommended by sensor or analyzer manufacturers and may also depend on the rate of biofouling.

*Who:* Personnel usually require training to operate and deploy in situ sensors or analyzers. Each sensor or analyzer may require different knowledge and skills. A good practice is to have dedicated personnel to conduct deployment and maintenance throughout the deployment period. Deployment personnel should maintain a log of deployment, maintenance, calibration, verification, and other important events.

*How:* A wide variety of in situ sensors and analyzers have been used to study C pools and fluxes in wetland waters. These devices are based on a wide range of methods, often requiring special knowledge for deployment to ensure high quality measurements.

*In situ pCO_2_ sensors:* Commonly available in situ *p*CO_2_ sensors generally rely on CO_2_ equilibration between the sample water and the headspace of gas (e.g., CO_2_-free air) or a reagent (e.g., a pH sensitive dye), followed by detection of the CO_2_-equilibrated medium using internal infrared detection (e.g., CO2-LAMP, SIPCO2, CONTROS HydroC^®^ CO2, Pro-Oceanus CO2; Johnson et al. 2010; Bastviken et al. 2015; Hunt et al. 2017; Blackstock et al. 2019) or colorimetric spectroscopy (e.g., SAMI-*p*CO_2_, Sunburst Sensors). While these sensors provide realistic estimates of *p*CO_2_ under some conditions, their application in physically dynamic environments can be particularly error-prone (e.g., tidal channels, lentic systems undergoing rapid overturn and mixing); these sensors are ideally applied alongside other independent measurement techniques (Loken et al. 2019; Bogard et al. 2020a; Trifunovic et al. 2020).

Water samples run through equilibrator systems can reduce the lag time needed for complete equilibration between sample water and headspace, thus increasing temporal resolution of water C and chemistry (Yoon et al. 2016). Rapid equilibration systems, such as sprayer- or marble-type equilibrators, strip water of dissolved gases. High frequency gas analyzer (Section “Chamber Measurements”) can be attached to watercraft with continuous water collection and equilibration, which allows investigators to relatively quickly describe spatial variation in dissolved C constituents and water chemistry parameters (e.g., Crawford et al. 2015).

Both *p*CO_2_ and *p*CH_4_ can be measured by submersible Membrane Inlet Mass Spectrometry (MIMS), which uses a thin membrane to pass analytes from wetland waters that are then transferred to a mass spectrometer for measurements (see Online Resources for addition details on MIMS). MIMS allows for rapid, continuous monitoring of the partial pressures of multiple gases simultaneously, including *p*CO_2_ and *p*CH_4_.

Solid-state *p*CO_2_ sensors (*p*CO_2_ optode) are also used in wetland waters (Atamanchuk et al. 2014; Clarke et al. 2017; Staudinger et al. 2018). The method is based on CO_2_ equilibration between the water sample and a pH sensitive fluorescent dye, followed by fluorescence detection (Atamanchuk et al. 2014; Clarke et al. 2017). Such sensors do not need pumping mechanisms to transport the gas samples, thus significantly reducing the complexity of deployment and maintenance, albeit with longer response times and potentially lower accuracy.

*In situ pCH_4_ sensors:* A range of commercial sensors are available for *p*CH_4_ measurements. Generally, the sensors rely upon equilibrating the aqueous phase and the gas phase, which can be achieved through various gas equilibration setups (Maher et al. 2013b; Trifunovic et al. 2020). Sensors are based on the absorption characteristics of CH_4_, with a high-sensitivity sensor (parts per billion [ppb] range) employing cavity-enhanced techniques including off-axis integrated cavity output spectroscopy (e.g., Los Gatos Research instruments), cavity ring-down spectroscopy (e.g., Picarro instruments), and tunable infrared laser direct absorption spectroscopy (e.g., Aerodyne Research Instruments). These cavity enhanced techniques have also enabled in situ measurement of stable C isotope ratios of CH_4_ due to differences in the infrared adsorption spectra of ^12^CH_4_ and ^13^CH_4_ (Xu et al. 2022). The air–water equilibration times need to be considered as the low solubility of CH_4_ can result in long equilibration times from minutes to days (Bastviken et al. 2015; Webb et al. 2016). *p*CH_4_ sensors in wetland surface water work well when concentrations are high (e.g., > 6,000 umol/mol in Trifunovic et al. 2020), but *p*CH_4_ is often low and below the detection limits of in situ instruments. It can be challenging to attain time-series measurements of belowground dissolved *p*CH_4_ because water sources may change faster than in situ sensors equilibrate. One approach to avoid this issue is to install buried gas equilibration chambers belowground that allow dissolved gases to equilibrate across a gas-permeable membrane into an N_2_-filled chamber (Schutte et al. 2016), which can then be connected to a gas analyzer.

*In situ DIC sensors:* In situ DIC sensors are less common than *p*CO_2_ sensors (*circa* 2022). However, prototype in situ sensors and flow-through analyzers for DIC measurements have been reported (Wang et al. 2007, 2013b; Sayles and Eck 2009; Liu et al. 2013; Fassbender et al. 2015). In situ DIC detection methods of these systems is generally based on CO_2_ equilibration between an acidified sample and a standard solution through a gas permeable membrane, followed by either spectrophotometric, conductometric, or infrared absorption detection.

Some in situ sensors can simultaneously measure two out of three carbonate system parameters (i.e., DIC, pH, *p*CO_2_) (Wang et al. 2015). These dual sensor systems are desirable because the aquatic carbonate system can then be fully resolved with equilibrium calculations using the two measured parameters. In addition, using measured pairs of DIC-pH, DIC-*p*CO_2_, or pH-*p*CO_2_ in such calculations may result in smaller calculation errors (Rao and Ma 1993; Wang et al. 2015). For example, Channelized Optical System (CHANOS; Wang et al. 2015) has been designed to make simultaneous measurements of the DIC-pH pair or the DIC-*p*CO_2_ pair based on spectrophotometric principles (Fig. 11a). The system has been deployed in a coastal salt marsh to capture high-resolution lateral DIC exports (Chu et al. 2018).

*In situ CDOM/FDOM sensors:* DOM is a large mixed pool of components that have very different absorbance, fluorescence, and reflectance properties. Optical sensors are capable of measuring DOM in these pools and providing an accurate record of spectrally active compounds, whether through absorbance (e.g., CDOM) or fluorescence (e.g., FDOM) (Henderson et al. 2009; Watras et al. 2011; Downing et al. 2012). CDOM and FDOM sensors are often deployed to determine DOC concentration and derive DOC sources and fluxes. In situ FDOM sensors provide almost instantaneous results, are non-invasive, and often contain loggers to collect data. Excess turbidity, high DOM concentrations, and high temperatures can interfere with FDOM measurements and shift the relationship between FDOM and DOC concentrations, which can be accounted for by using correction factors (Henderson et al. 2009; Watras et al. 2011; Downing et al. 2012). As with most sensors, calibration of FDOM sensors is required based on manufacturer specifications. CDOM concentrations in surface water can also be determined using remotely sensed information (Cao and Tzortziou 2021).

*Key Covariates and Ancillary Measurements:* The suite of additional variables to measure are generally the same as those previously described above in Section “Water Sample Collection - Surface Water, Porewater, Groundwater”. Many of the multiparameter systems that can be used for point-in-time measurements are also designed for continuous in situ measurements.

### Carbon in Wetland Vegetation

#### Definitions and Units

*Definitions:* As wetland plants grow, they fix atmospheric CO_2_, dissolved CO_2_, and/or HCO_3_^−^ into complex organic compounds to build biomass aboveground and belowground. Vegetation (i.e., the assemblage of individual plants in a site or region) biomass in wetland systems can be substantial, representing a relatively large living C pool (Kayranli et al. 2010; Zou et al. 2021; Chou et al. 2022). Either from mortality, senescence, or disturbance, much of plant biomass moves to detrital pools, where it can be consumed by detritivores, decomposed back to the atmosphere as CO_2_, removed via lateral transport, or incorporated into SOC pools. Aboveground biomass largely decomposes to atmospheric CO_2_ over years to decades, whereas belowground biomass has greater opportunity for long-term soil incorporation. The relative rates of vegetation dynamics depend on the environmental conditions present in the wetland (Brinson et al. 1981). Growth and mortality rates determine the residence time of C in plant biomass, which varies depending on the life history characteristics of species so that annual vegetation, perennial macrophytes, and woody species vary in the duration of their stored C pool. The C content of plants also varies by species and vegetation type (Brinson et al. 1981), for example, live woody tissues in trees vary in C content from 42 to 56%, with higher amounts of C in conifer versus deciduous trees (51 versus 48%, respectively; Thomas and Martin 2012). The differences in plant form and functional types among wetlands species, and the conditions in which they grow in wetlands (e.g., open air versus submerged underwater), require different methods to assess biomass, with one of the biggest differences being between forested versus herbaceous wetlands.

*Units:* Vegetation biomass is typically reported in g m^−2^ at the sample plot scale, often based on estimates from smaller scale (e.g., 0.25 m^2^) measurements (Table 6). As with soils, C pools in vegetation are often scaled-up over large areas from the sample unit and are reported in Mg C ha^−1^.

**Rationale:** C in vegetation is the primary pathway linking atmospheric C to soil C, serving as a conduit for SOC accumulation and the associated radiative cooling effects of wetlands on global climate (Neubauer and Megonigal 2015; Nayak et al. 2022). At its base level, vegetation production is the dominant C input in most wetlands (i.e., net primary productivity [NPP]). Vegetation also influences other C pools. For example, deep soils are formed volumetrically from historical plant inputs (Temmink et al. 2022). Similarly, water column C is leached or released from living and dead plant and algal tissues, fueling heterotrophic respiration (R_H_) and trophic transfers or lateral exports. Vegetation has biophysical effects on other wetland C pools by acting as gas conduits through aerenchymatous tissues, thereby releasing CH_4_ to the atmosphere (Bansal et al. 2020). Plants also stabilize soils, reducing erosive losses. The C pool in vegetation is also subject to rapid change from environmental conditions such as weather (e.g., storm events, heat waves), and from land-management activities such as herbicide application, burning, or biomass harvesting. Methods to collect vegetation data efficiently, precisely, and accurately are critical tools for understanding C uptake and developing strategic vegetation-management policies to mitigate climate change.

#### Biomass – Herbaceous Vegetation

**Fig. 12:**
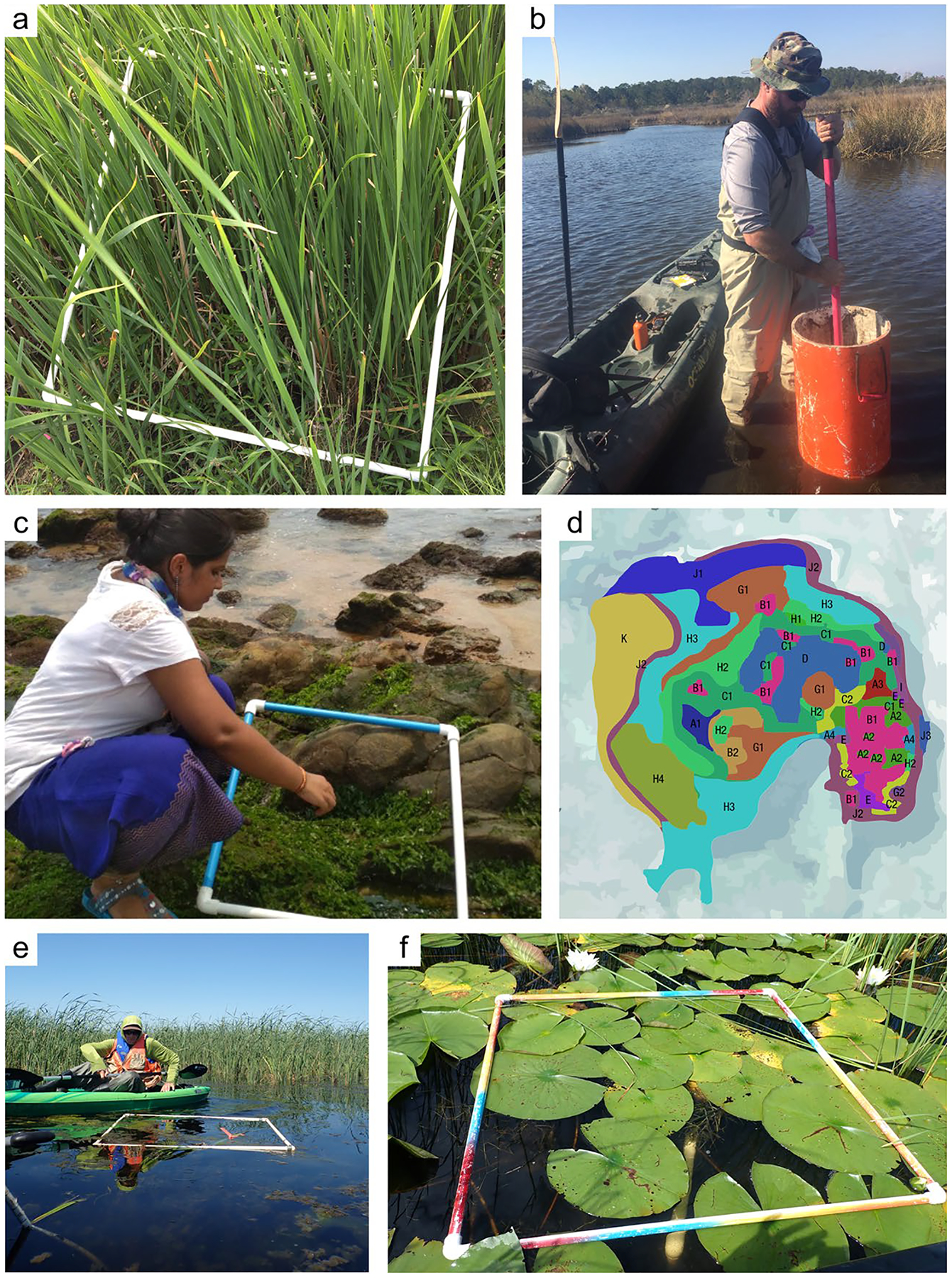
Various methods to assess aboveground biomass. (**a**) 0.25 m^2^ polyvinyl chloride (PVC) quadrat marking a clip plot for *Typha* biomass; (**b**) using a cylindrical unit sampler with a modified rake to collect submerged aquatic vegetation in coastal South Carolina (USA) estuarine wetlands (Bauer et al. 2020); (**c**) collecting macroalgae using a PVC quadrat from Vishakhapatnam coast of Andhra Pradesh in western Bay of Bengal, India; (**d**) map of vegetation zones within a prairie pothole wetland (Williams 2015); (**e**, **f**) sampling floating vegetation using a PVC quadrat from a kayak in Munuscong Marsh along St. Marys River, a Great Lakes connecting channel between Lakes Superior and Huron, North America. Images with permission from Olivia Johnson (a), Beau Bauer (b), Kakoli Banerjee and Prajna Paramita (c), Shelby Williams (d), and Logan St. John (e, f)

*What:* The two main approaches to quantify C pools in the biomass of herbaceous vegetation are *harvest* and *allometry* (Howard et al. 2014). Harvesting plants provides direct information on the quantity of biomass at a given location and time. Harvesting works well for emergent vegetation because their aboveground stems can be relatively easily encompassed by a standard size quadrat and clipped at the soil surface (Fig. 12). The inherent mobility of floating and submerged vegetation can pose challenges to the harvesting method. Harvest may yield more accurate measurements of biomass than allometry, though harvesting still has a set of potential problems, for example, destructive sampling may create experimental artifacts that can interfere with other components of a study. Harvest methods do not account for losses due to leaf senescence, grazing, or root exudates, but can, if conducted repetitively over a year, account for aboveground losses. Measurements of belowground biomass pose unique challenges. For example, when collecting soils to estimate root and rhizome biomass, the size and location of the soil volume sampled can lead to bias due to high belowground heterogeneity.

Indirect methods to assess plant biomass include measuring a proxy variable (e.g., cover, density, or height) (Fig. 12) and using allometric regression equations to estimate biomass (e.g., Fennessy et al. 1994b). The non-destructive and relatively quick sampling of allometric methods may allow for higher replication than harvest methods. Allometry may also be preferred over harvest for establishing permanent plots with repeated biomass assessments, or if there are other concerns with removing biomass, for example, if the study site is relatively small, if accumulation of inter-seasonal biomass is an important component of study, or if there are regulatory considerations (e.g., endangered species/protection). However, there can be relatively large errors associated with using allometry compared to harvest methods, especially if there is variation in hydrology, salinity, bulk density, and soil nutrients that affect aboveground and belowground plant growth (e.g., Stagg et al. 2018; Chou et al. 2022). For an overview of equations and values for biomass, see Craft (2013), Conner and Cherry (2013), Rivera-Monroy et al. (2013), or Banerjee et al. (2022a).

*Where:* For both harvest and allometric methods, first consider the zonation of vegetation in a wetland made up of distinct plant communities (Fig. 12d). Zones can be delineated using aerial photography or by traversing along borders of zones using GPS and mapping the GPS points using Geographic Information System (GIS) software. In coastal marine systems, macroalgae typically are found attached to substrates in sandy and rocky areas along coastlines in intertidal mudflats and subtidal zones. One or multiple plots are typically established in each vegetation zone, which can either be randomly or systematically distributed. The number of plots required depends on the total area of wetland and the degree of heterogeneity of vegetation; an optimal number of plots will capture within-wetland variation (sites with more heterogeneity need more plots). If upscaling in space is a research goal, estimates of the areal coverage of each zone or community type are needed. It is important to space plots in a way to avoid experimental artifacts/disruptions from walking/trampling across a wetland.

Establishing plots is relatively straightforward in zones with emergent macrophytes because vegetation is rooted in place and plot markers, such as flagging or PVC pipes, can typically be left in place during the growing season (or longer) (Fig. 12a). Plots in open water with floating and submerged vegetation and algae are more difficult to establish because vegetation may be mobile and plot markers often need to be anchored in the sediment or geolocated using GPS (Fig. 12e, f).

*When:* Vegetation biomass can either be measured multiple times during the year or as a one-time measurement, depending on the purpose of the survey, regional climate, and environmental conditions. A one-time harvest approach is typically conducted during peak biomass in regions with distinct growing and non-growing seasons. Peak biomass occurs when plants have reached their maximum biomass prior to senescence. The timing of peak biomass is species-specific, so multiple sampling events during the growing season may be required to capture each peak. Peak biomass harvests are convenient because they are often closely aligned with annual NPP (Section “Net Primary Productivity”). A single, belowground harvest generally occurs near the end of the growing season after roots and rhizomes have fully developed and belowground carbohydrate allocation is complete (Asaeda et al. 2008; Tursun et al. 2011).

Seasonal patterns in water levels may affect sampling efforts (e.g., pre-monsoon, monsoon, and post-monsoon in tropical systems). For example, it is more difficult to extract a standard volume of belowground biomass if there is standing water at the collection location, therefore harvest of belowground biomass samples at the time of lowest water depth is preferred. For non-tidal wetlands, sampling can occur any time of the day, although diurnal fluctuations in environmental conditions and insect or animal activity may affect comfort and safety. In tidal wetlands, vegetation is overtopped with water during much of the diel cycle. Therefore, sampling ideally occurs during low tide, which may be limited to once or twice a day for 3 to 4 h at a time.

*Who:* Biomass sampling is relatively straightforward for field technicians after plot locations and protocols are established. More training is required if species identification is desired, especially if belowground separation by species, or by live versus dead biomass, is required. Additional specializations and equipment are required to measure many of the key covariates and ancillary measurements.

*How:* The primary methods to assess vegetation biomass are different for aboveground versus belowground biomass.

*Aboveground harvest:* For emergent vegetation, plant biomass from the current growing season is cut to ground level (i.e., clip plots, Fig. 12a) and removed from plots with dimensions usually ranging from 0.25 to 1.0 m^2^. Larger plots are more representative of vegetation biomass and community, but require more time and effort. Submerged aquatic vegetation and floating vegetation biomass can be harvested from the water column using a rake and a cylindrical core (Fig. 12b) or quadrat sampling device (Merino et al. 2005; Howard et al. 2014; Bauer et al. 2020; Masto et al. 2020; King et al. 2023). Cylinders can be made of PVC and the edges can be sharpened or lined with metal cutting teeth to facilitate substrate penetration and separation of roots (Bauer et al. 2020). Different species or functional groups and plant litter are ideally separated during harvest and stored separately. If species or functional groups (e.g., emergent, floating, submerged) are not separated for biomass analysis, it is advisable to at least record a percent cover for each species or functional group within the quadrat before harvesting. Care should be taken, especially in emergent biomass harvest, to distinguish between dead plant biomass (from previous years) and senescing biomass that is still attached to the present year’s growth if the goal is to assess annual biomass production. For seagrasses, epiphyte loads on the leaves can be removed and analyzed separately (Howard et al. 2014).

Once the aboveground plant tissue has been appropriately sorted, it is then dried (~ 60–70 °C) and weighed. Drying at higher temperatures can volatilize N, which will have minimal effect on weight but affects ancillary measurements of tissue quality. It is important to dry plants relatively quickly after harvest to avoid mold growth. If drying is not possible in a timely manner, samples can be stored in a cool-box or freezer. The sampling supplies needed for the harvest method are relatively low-cost and conventional (e.g., clippers, bags, dryer, scale). For more details on macrophyte harvest methods, see Craft (2013) and Howard et al. (2014). After aboveground biomass is dried, it is weighed and the total C content for each species or functional group is estimated by multiplying the dry weight of the organic matter per area by the C conversion factor, which is either directly analyzed or drawn from literature value (see below *Carbon conversion factor*). The total C content of aboveground biomass can then be calculated and scaled by vegetation cover to the total representative area (e.g., Pg C km^−2^).

Macroalgae (e.g., seaweeds, kelp) do not have true leaves, stems, or roots, and are typically found attached to substrates (Fig. 12c) in sandy and rocky areas along coastlines and in subtidal zones; thus, biomass is collected at the whole-thallus (plant body) level for species with undifferentiated thalli, or subdivided into stolon/stipe and blade sections (Trevathan-Tackett et al. 2015; Kumar et al. 2023, see Online Resources for additional information on macroalgae). C content of macroalgal thalli is measured following similar methods described for vascular plants above. Tissue can be washed in situ with seawater to discard foreign extraneous particles. In the laboratory, the fresh macroalgae are thoroughly washed with tap water to remove the remaining salt and sand from the surface of the sample. The fresh algae are spread over blotting paper to eliminate excess water prior to drying. It is important to note that the contribution of macroalgae to coastal wetland C budgets is debated. Most macroalgae occur on hard substrates, potentially limiting their C sequestration potential in soils (Hill et al. 2015). The challenges associated with integrating macroalgal C pools into wetland C budgets are reviewed by Krause-Jensen et al. (2018).

*Aboveground allometry:* In emergent, macrophyte-dominated wetlands, measurements of aboveground cover, height, or stem density in plots can be used as a proxy of aboveground biomass. The proxy can then be used to estimate aboveground biomass using allometric regression equations. Allometric regression equations can be determined either empirically by destructively harvesting nearby plots, or by using published equations derived from the same plant species growing in similar conditions. For example, Lu et al. (2016) provide allometric data and relationships for several coastal marsh species including *Schoenoplectus americanus* and *Phragmites australis* under a range of environmental conditions. Also, Fennessy et al. (1994b) used allometry to estimate aboveground biomass production of *Typha* spp. in restored freshwater systems. Regressions to predict biomass can also be developed using water and soil chemical parameters. For example, Banerjee et al. (2022a) demonstrated that the peak aboveground biomass of salt marsh grass (*Porteresia coarctata*) in a coastal wetland of India could be predicted well using the covariates of water temperature, water and soil pH, water and soil salinity, soil bulk density, soil organic C, and texture.

*Belowground harvest:* For belowground harvesting, soil substrate is collected as a block or core (Neill 1992) with standard size and depth dimensions using shovels or soil cores (Section “Soil Collection”). Soils are then washed on a sieve, usually 2 mm, to separate soils from plant tissue, and all roots and rhizomes are removed (Section “NPP – Herbaceous Vegetation”). Live plant biomass can be separated from dead plant biomass by placing soils in water as living tissues tend to float in water, or by using staining methods (Bernard and Fiala 1986; Nieman et al. 2018). Separating species requires experience and is often not possible if tissues are similar or decomposed. After sifting and sorting roots, belowground biomass is then dried (~ 60–70 °C) and weighed (Whigham et al. 1989). Similar to the aboveground harvest method, the sampling supplies for belowground harvest are relatively low-cost and conventional (e.g., shovels; although corers can be expensive). Belowground harvest may provide more accurate information, but can be extremely time consuming in both sample collection and processing. Depth of root biomass is another important metric for C inventory and modeling.

*Belowground allometry:* The ratio of belowground to aboveground biomass (also referred to as root to shoot ratio) has been established for many plant species, and is therefore often used to estimate belowground biomass from measures of aboveground biomass (Howard et al. 2014; Pan et al. 2020). Belowground allometry is much faster than harvesting, but is subject to greater error (e.g., Chou et al. 2022).

*Key Covariates and Ancillary Measurements:* Many variables can aid interpretation of herbaceous biomass data, such as plant tissue chemical composition and C content, the production of plant litter, and plant community composition and structure. Many of these ancillary measures are also useful for tree-dominated wetlands and for measurements of NPP.

*Carbon conversion factor:* The percentage of plant biomass that is C can be determined either using values from the scientific literature, or empirically using a CHN analyzer or LOI methods (similar to methods used for soil C; Section “Carbon in Wetland Soils”). Briefly, plants are dried (~ 60 °C), ground, and weighed before elemental analysis in a CHN elemental analyzer. If using LOI, the percent organic matter can be obtained by measuring the difference between dried plant weight and the remaining mass after combustion at 550 °C, known as ash-free dry weight, which is then converted to % C by applying a conversion factor (e.g., 42–56%).

*Plant litter biomass:* The amount of plant litter in a wetland is, in itself, an important and often large wetland C pool. In addition, like live biomass, litter can affect a number of other ecosystem functions (Stoler and Relyea 2020). Aboveground, non-woody litter tends to be ephemeral and a relatively high fraction of aboveground litter decomposes to CO_2_ or is translocated out of wetlands via lateral transport. Belowground litter often breaks down over time and becomes part of the SOC pool. Emergent wetlands can build up significant amounts of dead grasses and grass-like vegetation such as sedges and rushes, sometimes referred to as ‘thatch’. The height and density of thatch can alter biophysical conditions that affect wetland C dynamics (e.g., blocking sunlight, inhibit NPP, Bansal et al. 2019).

Plant litter biomass can be measured using similar techniques as for aboveground and belowground biomass, usually in conjunction with live plant biomass measurements. One common challenge is determining where the litter layer ends and transitions to the surficial sediment, which can be composed of mucky or fluid material. In the field, materials that are in some stage of intermediate decomposition and cannot be identified are typically considered flocculated organic C (sometimes referred to as ‘floc’). A more standardized and quantitative approach to distinguish between litter and floc is to pass the sample material through a sieve (1–10 mm); any material that passes through the sieve is no longer considered to be litter (Bärlocher 2005). However, sieving is not practical in many situations, for example, root mats do not pass-through sieves.

*Environmental conditions:* For a given species, the total amount and relative allocation to aboveground versus belowground biomass in a wetland is governed by several soil properties including soil temperature, moisture, chemistry (e.g., salinity and nutrients), and dry bulk density (Section “Carbon in Wetland Soils”). Hydrological status is also important (e.g., water table depth, soil moisture). Since water table and soil moisture are dynamic at daily to longer time scales, periodic measurements of these variables throughout the year are ideal to characterize hydrology. Where possible, measurements of redox state and concentrations of porewater electrical conductivity in saline-prone areas, as well as N, P, and S in soils and porewater can be useful to understand variation in C pools and fluxes (Section “Soil Analysis - Bulk Density, Loss-on-Ignition, Elemental Analysis”).

*Vegetation community composition and structure:* Species abundance and composition are primary drivers of plant and litter biomass. Changes in plant community composition, such as through management actions or the spread of invasive species, can have large effects on biomass production. For example, large stature invasive emergent species such as *Typha* × *glauca* or *Phragmites australis* tend to have much larger biomass compared to their native counterparts (Bansal et al. 2019). Species composition can be measured in biomass plots, or more generally across a wetland in each vegetation zone (DeKeyser et al. 2003). Plant community structure such as percent cover, and plant traits such as stem or leaf height, stem density, leaf area index (LAI), and reproductive status can help interpret the values of aboveground biomass in a given plot (e.g., are there many small plants or few large plants). Similarly, changes in species composition can alter ecosystem processes such as biomass production through changes in plant functional traits that control, for example, rates of C uptake and photosynthetic pathways (Chapin et al. 1997; Cavender-Bares et al. 2016).

There is growing evidence that ecosystem functions, such as primary productivity, are more dependent on the diversity of plant functional groups rather than the diversity of species (Hooper and Vitousek 1998; Hooper et al. 2005). Plant functional groups, organized based on similarities in physiological, morphological, and/or taxonomic traits, have been studied relatively less in wetlands than in terrestrial systems (e.g., Wardle et al. 2012; Bansal and Sheley 2016). Wetland mesocosm experiments have been used to quantify the effects of functional group diversity on biomass (Bouchard et al. 2007; Potvin et al. 2015). For instance, Bouchard et al. (2007) found that increasing functional group diversity led to greater belowground biomass, no change in aboveground biomass, and a decrease in CH_4_ emissions. Thus, understanding how the loss or gain of key functional groups alters C fluxes is important to understand how human activities and/or restoration efforts affect wetlands.

*Tissue quality:* C content can vary by tissue type and species, requiring specific conversion factors. Byrd et al. (2018) calculated a mean value of 44.1% for coastal marsh aboveground vegetation. While the majority of plant C is stored in structural tissues such as cellulose, other compounds such as lignin, lipids, and starch, can also contribute to biomass depending on tissue type and time of year. For example, translocation of carbohydrates during autumn into rhizomes of emergent vegetation can be substantial (e.g., 50% of biomass, Asaeda et al. 2008), and critical for spring re-growth or clonal regeneration following disturbance (Tursun et al. 2011; Bansal et al. 2019). Methods to measure carbohydrates typically involve enzymatic or acid digestion of finely ground plant materials followed by spectrophotometric analysis (Asaeda et al. 2008; Bansal and Germino 2010; Chen et al. 2013) with blanks and standards. Of the various chemical constituents of vegetation, lignin content plays a crucial role in determining rates of plant litter decomposition, and thus the residences time and fate of plant C (for lignin analysis see Section “Litter and Organic Matter Decomposition”).

Other chemical constituents in wetland vegetation, such as protein or lipid content, can be useful to understand rates of growth and how changing environmental conditions (e.g., sea-level rise) may affect future growth rates. In addition, wetland plants, such as macroalgae, are being used as food sources to serve the growing needs of human nutrition (see Online Resources for information on nutrient values of macroalgae and methods to measure tissue protein, carbohydrate, lipid, and astaxanthin content).

*Root exudates:* Rooted plants can lose C to soils as root exudates, which can contribute to the SOC pool, prime microbial respiration, and fuel methanogenesis (Basiliko et al. 2012; Waldo et al. 2019). Measurements of root exudates directly is challenging, so indirect methods using ^13^C labeled CO_2_ or root exudate analogues (e.g., glucose, citrate, amino acids) can be used to estimate the relative contribution of exudates to the SOC pool, respiration, and methanogenesis (Basiliko et al. 2012; Girkin et al. 2018; Waldo et al. 2019).

#### Biomass – Trees

**Fig. 13:**
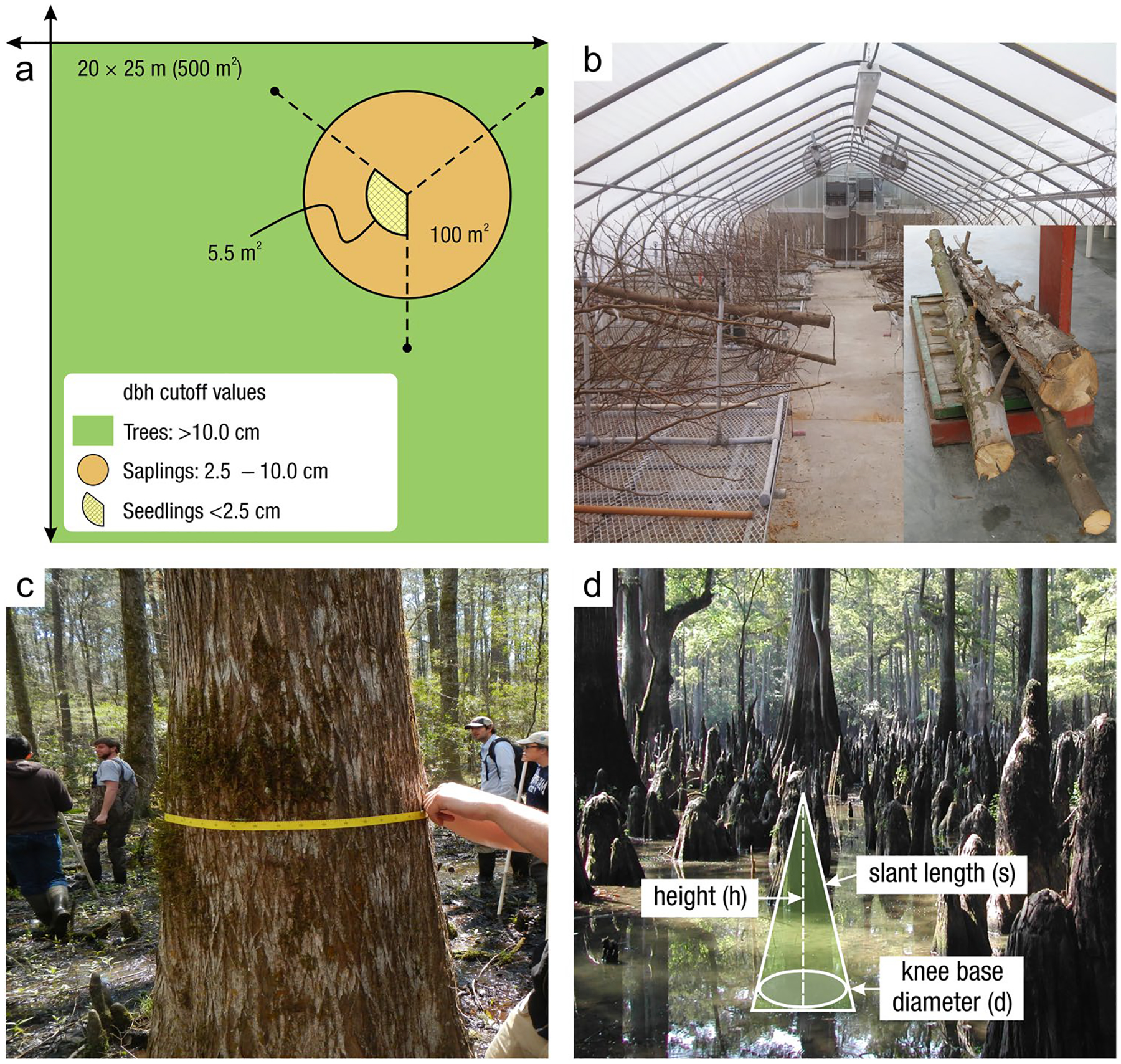
(**a**) Wetland tree biomass survey plot (20 × 25 m size); All trees > 10 cm diameter at breast height (dbh, 1.3 m) are measured in the green area, all saplings > 2.5 and < 10 cm dbh are measured in the orange circle, and seedlings < 2.5 cm dbh and shrubs are measured in the yellow hatched section; the dotted lines represent 10 m transect locations for dead and downed wood surveys; (**b**) drying whole trees and weighing (inset) specimens to determine biomass of the main trunk; (**c**) measuring dbh using a diameter tape measure; (**d**) standard knee data required for biomass calculation (Middleton 2020b). Images with permission from Jamie Duberstein (a), Herman W Hudson III (b), William Conner (c), and Beth Middleton (d, with permission of Elsevier)

*What:* The two main approaches to tree biomass assessment are harvest and allometry (Fig. 13). Harvest methods can provide valuable information about species’ biomass in specific regions, but suffer from being energy intensive, costly, usually limited in spatial scope, and destructive to the study site (Fig. 13b). Allometry uses new or previously published relationships between measurable tree metrics such as diameter at breast height (dbh: 1.3 m) or root collar diameter, tree height, and either whole-tree biomass or biomass of specific tree components if the methodology allows (Fig. 13c, d). Biomass estimates of non-vine woody plants (e.g., shrubs) use different equations and parameters depending upon the size class of the main trunk (also referred to as ‘bole’). Repeat allometric measurements of tree biomass provide information on the incremental change in biomass, which is used to estimate NPP (i.e., as conducted by the U.S. Forest Service, Forest Inventory and Analysis [FIA]; Section “Net Primary Productivity”).

The dbh size cutoffs for seedlings, saplings, and adults can differ among studies, growth forms, and wetland ecosystems. Tree surveys are often limited to individuals ≥ 10-cm dbh, which typically account for ≥ 90% of aboveground biomass in large stature forests in temperate and tropical environments, such as pine pocosins, baldcypress/tupelo swamps, maple/gum swamps, and bottomland hardwoods (Clark et al. 2001; Hawbaker and Duberstein 2019). In the United States, the FIA national surveys consider that *adult trees* have trunks with a dbh greater than 10.0 cm; *saplings* are 2.5 to 9.9 cm dbh; *seedlings* and *shrubs* are less than 2.5 cm dbh. For smaller stature forested wetlands such as mangroves, trees can be considered adults at 5 cm dbh, saplings from 1 to 5 cm dbh, and < 1 cm for seedlings (Murphy and Lugo 1986; Krauss et al. 2018a, 2020). A common forest survey of standing biomass will include all live trees, while a more thorough survey will include saplings, seedlings, and other herbaceous vegetation (Fig. 13a). Surveys often include standing dead trees in which case the decay class needs to be evaluated (Domke et al. 2011).

The methods to estimate tree biomass described here are for the portion of tree above 30 cm, below which is considered the stump. Stump volumetric estimates are available for some species in the eastern United States (Raile 1982), with some adjustments published later (Woodall et al. 2011). Coarse root biomass and knee biomass of baldcypress (*Taxodium distichum*) can also be estimated (Fig. 13c) (Jenkins et al. 2003; Middleton 2020a). Estimates of dead and downed woody material biomass may also be included, and are particularly important in areas that have undergone rapid community shifts due to major storms events or other disturbances (Sturtevant et al. 1997; Allen et al. 2000; Krauss et al. 2005). To convert tree biomass to C biomass, oven dried biomass estimates are typically multiplied by a scalar of 50% (Birdsey 1992; Woodall et al. 2011) unless species- and site-specific information is available.

*Where:* Assessments of forest biomass may use one or two plots in each forest community type or treatment group, depending upon goals and study design. Plot sizes can vary depending upon stem density, but tree surveys using 20 × 25 m (0.05 ha) plots are commonly sufficient to capture within-site variability in species diversity and size classes of most community types (Fig. 13a). Semivariograms can also be used to determine minimum plot distances (Section “Overview of Wetland Carbon Pools and Fluxes”; Cohen et al. 1990; Doughty et al. 2021).

If the communities of interest have a very dense understory component (e.g., > 15 stems m^−2^), it is often best to sub-sample the larger plot using one to three smaller inset plots. Saplings are often surveyed in 100 m^2^ plots while dense shrub surveys use plots as small as 5.5 m^2^ (Fig. 13a). Plots that are intended for repeated measures can be marked by placing PVC poles at the corners and recording their GPS positions. Similar to herbaceous plants, an estimate of the areal coverage of each community type within the region of interest is needed for scaling purposes.

*When:* Identification of species is necessary for most tree biomass estimation methods. Therefore, data are most often collected during the growing season, after the leaves of deciduous trees emerge. If it is anticipated that a study site will be measured repeatedly over multiple years, then the initial forest survey catalogs the species and locations (e.g., distance and azimuth from a central point) of trees in the plot.

*Who:* Forest inventory surveys are often completed by small teams of technicians who can identify the species common in the area of interest and have familiarity using a diameter-tape (d-tape) or calipers. Consistency in the data collected by all members of the team is extremely important, particularly for measures of dbh. If it is necessary to conduct surveys in winter months, skill in identification of species using characteristics other than leaves (e.g., leaf bud size and position, bark, bud scar, tree shape, etc.) is required.

*How:* Tree biomass is generally measured using allometry, although harvesting is also employed in some instances.

*Harvest:* Measuring whole-tree biomass empirically can be an energy-intensive endeavor for large saplings and trees. Tree spades can be used to mechanically extracts entire small trees including the immediate root systems, although this method is generally not feasible under flooded or saturated soil conditions. Many of the first harvest and allometry studies were conducted using seedlings and saplings (e.g., Telfer 1969; Roussopoulos and Loomis 1979; Williams and McClenahen 1984). Whole-tree harvest studies can be very informative due to relatively high accuracy compared to allometry, albeit biomass values tend to be influenced most by site-specific variables. Therefore, studies using empirical harvest data in wetland systems should consider hydrological conditions during the study (e.g., Hudson 2016).

*Allometry:* Most scientific studies of tree C pools and fluxes use allometric relationships between tree size and whole tree biomass, rather than destructive sampling. Biomass of individual tree components has been calculated through various studies, differing slightly by which components are separated (e.g., stem or trunk, branches, leaves). Tree size and biomass relationships have been summarized for many tree species by Megonigal et al. (1997), Jenkins et al. (2003), and Woodall et al. (2011). These allometric relationships represent the most widely used methods for calculating live tree biomass in the United States. Mangrove allometric equations can be found in Komiyama et al. (2002), Komiyama et al. (2005), Smith and Whelan (2006), Mitra et al. (2012), and Banerjee et al. (2022b). The methods differ in their input data needs, and algorithms are more complex as additional data are incorporated. However, most methods require a measurement of dbh and identification of species for each tree in the plot. Tree dbh is typically measured using a commercially available d-tapes or large calipers and is measured above any noticeable buttress (Fig. 13c). For measurements using calipers, recorded dbh is typically the average of two measurements taken at 90 degrees from one another. Tree height can be measured using handheld clinometers or laser-based height measuring instruments.

The simplest allometric relationships are summarized in Megonigal et al. (1997). This method uses species-specific equations for 15 of the most common wetland tree species in the southeastern United States, plus two non-specific species (‘other *Quercus’* and ‘other [all else]’). The greatest strength of this technique is the ability to use a single empirical measure of dbh to estimate wood biomass, often allowing investigators to apply the algorithms to datasets that have been collected previously. However, the applicability of the algorithms may be limited outside of the southeastern United States unless assumptions are made regarding the similarity between specific species. The biomass estimate obtained using this method is ‘wood production,’ which includes wood in the trunk and branches but does not include the foliage biomass. Because there is no inherent measure of foliage biomass, this method is best accompanied by a separate measure of leaf production such as provided by leaf litter traps (described below).

Allometric relationships provided in Jenkins et al. (2003) are inclusive of most species in the United States, having been developed by the U.S. Forest Service for application to mensuration data at the national scale. This method combines species into 12 groups, including six hardwood groups, five softwood groups, and one juniper/oak/mesquite group. The estimates involved with this method require at least 30 calculations, which not only provide an estimate of total aboveground biomass, but also allow for the separation of tree components into merchantable stem (i.e., the portion of the trunk extending from 30 cm from ground level to a 10 cm top; bark and wood separated), total foliage, coarse roots, and branches. This method includes an allometric equation for standing dead trees that requires an evaluation of the decay class (Domke et al. 2011).

Allometric relationships provided in Woodall et al. (2011) are the most comprehensive of the three methods described herein. While the previous two methods assume a minimum dbh of 10 cm, the Woodall et al. (2011) method has a minimum dbh requirement of 12.7 cm; smaller stemmed individuals are calculated as saplings (see below). The method involves species-specific allometric relationships for 464 species (wetland and upland) found in the United States, which may further vary by geographic region. The method was developed by many of the same authors as the Jenkins et al. (2003) method and is the current standard for the U.S. Forest Service. There are roughly 55 calculations required to estimate the biomass of each tree, including most calculations included in the Jenkins et al. (2003) method. Data requirements for this method include dbh, as with Megonigal et al. (1997) and Jenkins et al. (2003), in addition to tree height, adjusted tree height if the tree is broken at the top, and percentage of the merchantable trunk that has been lost by storm damage (i.e., percent cull). This method provides separate biomass estimates the main trunk, bark on the trunk, top of the trunk and branches (includes foliage), and bark on the branches.

Coarse root growth and stump growth of trees can be estimated in the same manner as aboveground biomass, provided all required data are collected (e.g., dbh, height, cull for methods of Woodall et al. 2011). An adjustment factor can be applied to stump volume estimates attained from Raile (1982) to estimate stump biomass and biomass of the stump bark. Standing dead trees are inherently included so long as height and decay class are measured. The greatest scientific value of the Woodall et al. (2011) method is its high accuracy of biomass estimates. However, the calculations require tree height and percent cull data, which are often not available within many existing datasets, limiting the widespread application of this method.

Mangrove allometric equations based on dbh can predict dry weight with reasonable accuracy, such as in southeast Asia, India, and the Florida (USA) Everglades (Smith and Whelan 2006). More complex allometric equations use diameter at 30 cm above highest roots, tree height, stem shape, wood density, branch diameters, and forking height (Komiyama et al. 2002; Komiyama et al. 2005; Smith and Whelan 2006; Komiyama et al. 2008). Allometric relationships can change regionally and with environmental conditions (Smith and Whelan 2006; Banerjee et al. 2022b). Aboveground root biomass can also be predicted using dbh, and it is often correlated with aboveground dry weight (Komiyama et al. 2005). Location of dbh measurement can be confusing in some mangrove forests that have a high incidence of *Rhizophora* spp., which has a prop root growth form sometimes obscuring an obvious stem intersection with the soil surface. In scrubby environments, best judgment of dbh location needs to be made relative to the allometric equations applied.

Trees and tree-like shrubs (e.g., *Alnus* spp.) sometimes have many stems per individual sprouting from the base as a growth form that can be natural (e.g., *Salix* spp.) or induced by damage or die-back events (i.e., coppice growth; Connolly and Grigal 1983; Semeniuk 1994; Osland et al. 2014; He et al. 2018). For studies with multi-stemmed species, it is generally more important to account for the number and size of individual stems compared to the number of individual trees, and the terms ‘stem density’ or ‘coppice density’ are used instead of ‘tree density’ (Mosseler et al. 2016).

Finer resolution estimates of plot-scale biomass are attainable by including saplings and shrubs. Sapling biomass can be estimated using Jenkins et al. (2003). In fact, the component ratios used in the allometric relationships (e.g., biomass of foliage) are based on data that include saplings, but the sapling biomass method per Jenkins et al. (2003) was designed for application to the U.S. FIA data, which are largely composed of adult trees. Heath et al. (2009) improved the Jenkins et al. (2003) method by developing sapling adjustment factors, which have been incorporated into Woodall et al. (2011).

Shrub biomass is generally estimated from measures of diameter at the root collar. Day and Monk (1974) and Dabel and Day (1977) developed allometric relationships for baldcypress, Atlantic white cedar (*Chamaecyparis thyoides*), and hardwoods in general using diameter at the root collar and two to three standard parameters, dependent upon species or general class (e.g., ‘mixed hardwood’), with leaves, branches, and stems as separate biomass components.

For baldcypress, knee biomass can be estimated by calculating the volume of knees using allometric techniques (Fig. 13d) (Middleton 2020a). Knee volume is calculated as a cone using field measurements of the slant length and diameter at the base and related to knee biomass regressed against the water displacement volume of the knee cone (see Middleton 2020a). Total biomass of knees in a study area can be determined by estimating the density and biomass of the knees using standard tree mensuration techniques (Middleton 2020a).

*Key Covariates and Ancillary Measurements:* Litter and downed biomass are important pools of C that are often measured along with standing biomass. Many of the ancillary measurements are similar between tree and herbaceous species, such as plant community composition and tissue quality.

*Litter fall:* Litter traps capture falling biomass to estimate the C pool of fresh (current year) litter of leaves, reproductive parts, and woody material from trees and shrubs. Ideally, if the area floods, the traps can be held above the water, or the traps themselves can float (similar to Middleton 1995). Monthly, seasonal, or annual collections of litter fall may be made, with higher frequency collections allowing for more information on turnover rates, and also to maintain equipment.

*Downed biomass:* Biomass of dead and downed wood, sometimes referred to as coarse and fine woody debris, can be estimated using a method that was designed to accommodate data from the U.S. National Fire Danger Rating System (Burgan et al. 1998). A series of 10-m transects can be placed randomly within a study area, or radiating from a single point (e.g., 60° 180°, and 320° azimuth directions; Fig. 13a). Fine woody debris is tallied when wood pieces intersect the transects. Woody debris is often suspended off the ground (e.g., fallen branch leaning against a tree); therefore, woody debris from the ground level to a height of 2 m above the ground are typically tallied. Woody debris is categorized into three fuel categories based on their size and how fast they burn. Fine woody debris surveys count the number of 1-h fuels (0–6 mm diameter) along the transect from 0 to 2.5 m, 10-h fuels (6.3–25 mm diameter) from 0 to 5 m, and 100-h fuels (2.5–7.5 cm diameter) from 0 to 10 m. The diameter of each piece of coarse woody debris (> 7.6 cm) is measured using a large caliper and assessed for its decomposition class at the point where it intersects the transect. The choice of decomposition class is subjective and therefore calibration within the assessment and across teams is ideal. The amount of time that different decomposition classes take to fully decay has an important effect on wetland C pools over time and is currently a gap in the literature. All tallies of downed biomass can be converted to biomass and C pool per unit area (e.g., Mg C ha^−1^) using algorithms described in Woodall and Monleon (2008) and Woodall et al. (2011).

## Carbon Fluxes

### Net Primary Productivity

#### Definitions and Units

*Definitions:* The rate of C fixation during gross photosynthesis (minus small losses from photorespiration) is referred to as ‘Gross Primary Productivity’ (GPP), while the use or dissipation of this fixed C as CO_2_ for metabolism and maintenance is autotrophic respiration (R_A_) (Brinson et al. 1981). Most emergent macrophytes and trees use atmospheric CO_2_ as their C source for photosynthesis, while many floating and submerged aquatic plants may also use water-borne HCO_3_^−^ as it may be several-fold higher in concentration than dissolved CO_2_ (Prins and Elzenga 1989; Colmer et al. 2011; Pedersen et al. 2013). The net balance of GPP and R_A_ is termed Net Primary Productivity (NPP), which represents the net rate of C gained by plants. The largest fraction of NPP is allocated to biosynthesis of plant tissues and structures, while smaller portions of C are allocated to non-structural compounds such as carbohydrates, root exudates, and volatile organic C compounds (Brinson et al. 1981; Chapin et al. 1990; Bansal and Germino 2008; Dundek et al. 2011; Grasset et al. 2015). With regard to wetland C, measures of NPP are convenient because they incorporate photosynthesis, multiple respiratory pathways, and biosynthetic processes into a single aggregate measurement associated with net C flux into plant biomass.

NPP is typically reported for aboveground (aNPP) and belowground (bNPP) plant production. Both aNPP and bNPP can be quite high in wetlands compared to other ecosystem types; for example, temperate zone swamps and marshes can produce plant biomass up to 3,500 g m^−2^ yr^−1^ (as dry weight), while mangrove forests, with mean global values of 171 g C m^−2^ yr^−1^ (Alongi 2018), can produce up to 5,400 g m^−2^ yr^−1^ (Klopatek and Stearns 1978; Cronk and Fennessy 2001). As with measures of biomass, species of different functional types require different methods to assess NPP (Cronk and Fennessy 2001). Rates of primary productivity vary by orders of magnitude among species, among life stages, within and between years, and also in response to myriad environmental factors including hydrology, climate, soil type, and nutrient availability (Brinson et al. 1981); additional landscape-scale research is still needed to address regional data gaps in primary production. bNPP is typically associated with that fraction of plant NPP that contributes to soil C accumulation, but is more difficult to measure, and therefore less frequently reported in the scientific literature than aNPP (Rivera-Monroy et al. 2019).

*Units:* The terms and methods used to report NPP vary and are not used consistently in the scientific literature, making it sometimes difficult to compare the results across studies (Table 6). Depending on the plant community type, C in vegetation is typically reported as the total organic matter produced annually, as g m^−2^ yr^−1^ (or some other unit of time), which can be converted to g C m^−2^ yr^−1^ by using conversion factors that relate plant biomass to C content, which can then be upscaled in space (e.g., Mg C ha^−1^ yr^−1^).

**Rationale:** Primary productivity, the rate of conversion of solar energy and CO_2_ (or HCO_3_^−^) into organic matter, is the foundation of energy flow though ecosystems, and thus a singularly important C flux for nearly all questions of C pools and fluxes. All heterotrophic organisms depend on the energy supplied by primary producers, and an understanding of primary production is important to quantify and model ecosystem functions, food web dynamics, and ecosystem-level C budgets (Brinson et al. 1981; Batzer and Sharitz 2014; Batson et al. 2015; Sleeter et al. 2017).

#### NPP – Herbaceous Vegetation

**Fig. 14:**
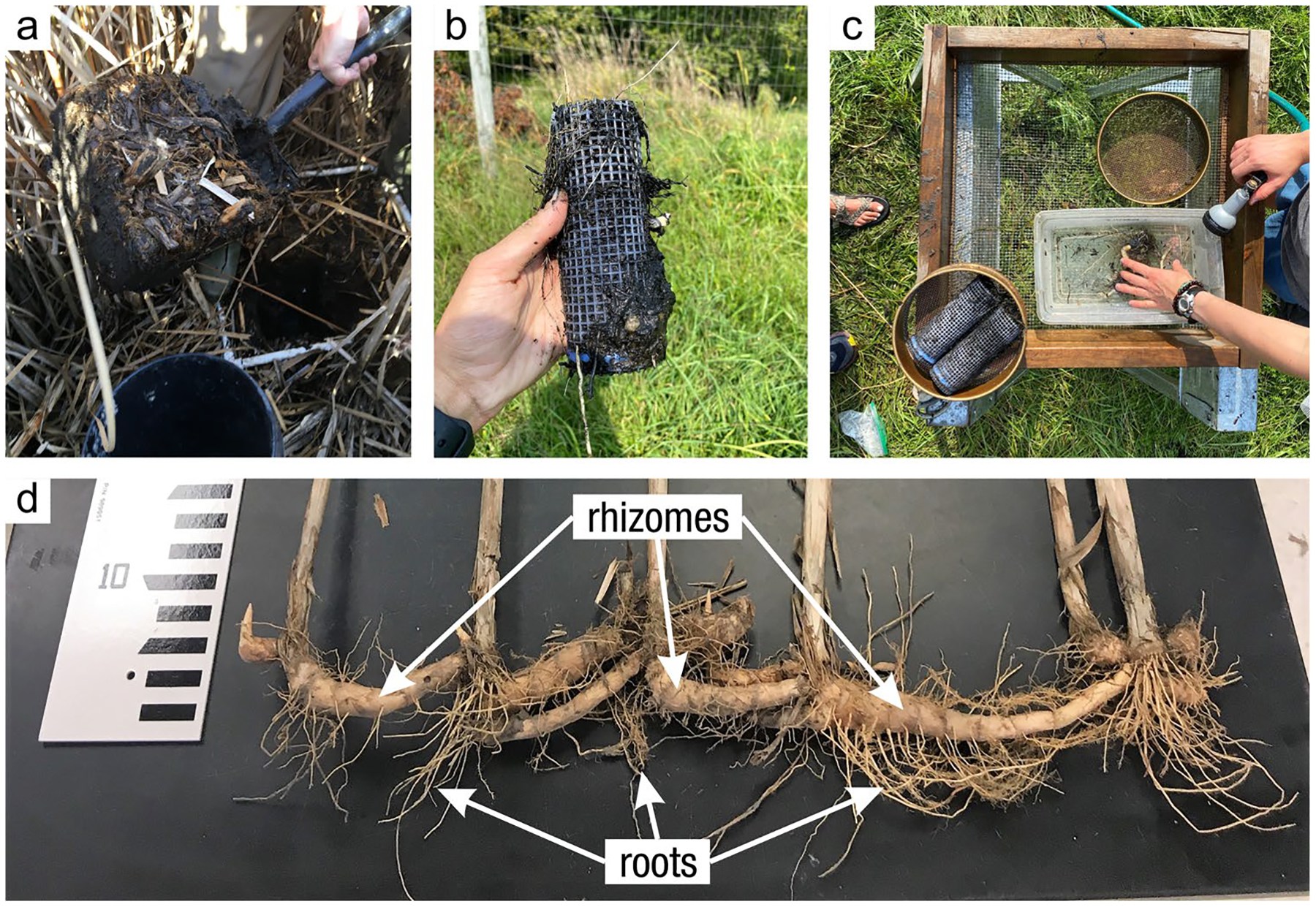
(**a**) Collection of belowground biomass and soils to a fixed size and depth into the rooting zone using a shovel; (**b**) root ingrowth core made of canvas mesh using the Kellog LTER protocol (KBS LTER 2008); (**c**) root washing station of *Spartina alterniflora* and *Phragmites australis* cores from a salt marsh in Connecticut, USA; (**d**) example of *Typha* rhizomes and roots. Images with permission from Olivia Johnson (a, d) and Madeleine Meadows-Mcdonnell (b, c)

*What:* Many of the same basic protocols described for measuring plant biomass apply for measuring rates of aNPP. aNPP can rely on direct harvest methods (one-time harvests during peak biomass or sequential harvests multiple times during the year) or on indirect methods that use physical allometric relationships. bNPP is more difficult to assess directly. Common techniques for bNPP involve serial collections using root ingrowth bags that assess the growth of root biomass into voids over time (e.g., marsh organs, rhizotrons) (Fig. 14). However, there is little understanding as to how the different bNPP techniques compare in wetlands (Krauss et al. 2022b), indicating that using multiple approaches may be needed for accurate estimates. Vegetation type can influence which approaches to use and what they capture (Fig. 14d); regardless of choice, all approaches are labor intensive. For this reason, bNPP is often modeled based on root to shoot ratios developed in previous labor-intensive studies.

*Where:* Plots for NPP measurements are generally distributed around wetlands in each distinct vegetation zone. The same criteria used to determine location for biomass, described in Section “Biomass - Herbaceous Vegetation”, can be used to determine representative locations for NPP assessment.

*When:* For aNPP, if conducting a one-time method, then measurements typically occur when plants are at their peak biomass. Phenology varies among species and by environmental conditions, so multiple measurements may be needed to capture peak biomass for each species × condition combination. For regions with year-round growth, repeated harvests typically occur at least twice: when biomass is at its minimum and at its maximum. Another consideration in the timing of sampling is the patterns of grazing intensity, as many wetland plants are subject to temporal variations in grazing that may alter estimates of the vegetation C pool. For example, grazing from sea urchin outbreaks can lead to the loss of aboveground and belowground biomass (Carnell et al. 2020).

*Who:* NPP harvest and allometric measurements are relatively straightforward for general technicians with basic training after plot locations and protocols have been established. bNPP is notably more challenging, and thus requires more training, especially for species identification and interpretation.

How:

*aNPP:* Whether using direct harvest methods or indirect allometric methods, many of the protocols are the same as for assessing aboveground biomass (Section “Biomass - Herbaceous Vegetation”), especially for emergent macrophytes and floating and submerged vegetation. One additional consideration for NPP is to separate current versus previous years’ growth to determine the amount of growth on an annual basis. It should also be noted that biomass harvesting does not account for volatile C compounds or root exudates, although losses from these sources generally are small. Herbivory can also cause an underestimation of NPP (Shaffer et al. 2015).

A more accurate assessment of NPP involves repeated measurements of biomass through the year or longer to provide an estimate of change in biomass per unit time. If conducting multiple assessments of biomass through the year, multiple plots can be established representing similar vegetative composition that can be harvested at different times throughout the growing season. There are many alternative repeat harvest methods (e.g., Smalley 1958; Wiegert and Evans 1964; Lomnicki et al. 1968; Kirby and Gosselink 1976; Morris 2007; Craft 2013), designed to increase accuracy by accounting for plant mortality that may occur before peak biomass. For example, Wiegert and Evans (1964) proposed using paired plots to estimate NPP of aboveground biomass and plant mortality by assessing the disappearance of plant material over time. In this paired-plot (A and B) design, live and dead biomass is harvested from plot A at the beginning of the study (t_0_), while only dead material is harvested from paired plot B. During a subsequent visit weeks or months later (t_1_), the new dead plant biomass in plot B is used to calculate the instantaneous rate of disappearance of biomass over the time interval between visits, normalized by the number of days (g day^−1^). This, combined with the biomass of plot A, is used to estimate of NPP by accounting for growth (increase in live biomass), mortality (difference in dead biomass), and the disappearance of dead material over that time period. This method assumes that the rates of mortality in the two plots are equal. Lomnicki et al. (1968) simplified this method by adding additional measures of live biomass. In this approach, both live and dead biomass are harvested from plot A at time t_0_, and only dead biomass is collected from plot B. At time t_1_, any new dead material is collected from plot B (as a measure of mortality over that time period, *di*) and the live and dead material is harvested from a newly selected Plot A (i.e., a plot not sampled at time t_0_). The change in live biomass is calculated as the difference of live biomass found in the two A plots over that time interval (Δ*bi*), then the dead biomass is added to estimate NPP. Thus, NPP = *di* + Δ*bi*. Craft (2013) recommends the Lomnicki et al. (1968) method due to its simplicity compared to the more onerous calculations required using the Wiegert and Evans (1964) approach.

*Macroalgae and seagrasses:* Due to challenges of repeated harvesting of macroalgae and seagrasses, direct biomass approaches typically use a photorespirometry method that measures changes in gas concentrations while chambers (e.g., bottle filled with water samples) are exposed to light and dark cycles (Tait and Schiel 2010). This approach can be applied in the laboratory or in some field scenarios, especially in calm intertidal or subtidal habitats with low profile vegetation (Giurgevich and Dunn 1982; Morris 2007; Tait and Schiel 2010; Howard et al. 2014). DO or dissolved CO_2_ are measured at varying light levels (Section “Carbon in Wetland Waters”) to estimate GPP, R_A_, and NPP, respectively, similar to the diel O_2_ method to assess metabolism in the water column (Section “Eddy Covariance”). Submerged plants can also be harvested, their apical tips incubated in light and dark bottles to assess GPP, NPP, and R_A_ (Yoshida et al. 2022). Care must be taken to remove respiring invertebrates. Detailed descriptions of chamber constructions and flux calculations are reviewed in Howard et al. (2014).

*bNPP:* The approaches described below involve collecting samples over relatively small areas (tens of cm^2^), thus relatively high replication may be needed to adequately capture belowground heterogeneity.

*Serial coring:* Among the most common empirical techniques used to measure bNPP is serial coring (Vogt et al. 1991; Brunner et al. 2013). For this method, cores are extracted repeatedly from specific locations on a site over multiple months spanning at least one full growing season or year, and often longer. Cores can be of any volume or depth, as long as the targeted rooting zone is included and there is consistency among serial cores. The assumption is that the change in root biomass in cores collected serially describe bNPP at the sample location. Sampling at multiple locations and core series is ideal, but can increase total samples required (e.g., 10 locations × 6 time periods = 60 cores). Once individual cores are extracted, they are transported to the laboratory on ice in a cool-box, and stored under refrigeration until processing. Soil core processing is described in Section “Soil Analysis - Bulk Density, Loss-on-Ignition, Elemental Analysis”. During processing, roots are separated into live and dead tissue, and often by diameter size class (e.g., fine roots: ≤ 2 mm, coarse roots: > 2 mm; note that definitions of diameter size class vary among studies) (Fig. 14c). Live roots sometimes float in water compared to dead roots, although there are many exceptions. A subsample of roots can be stained (e.g., 1% tetrazolium red) to assess vitality (Windham-Myers et al. 2009). The cores are often divided into depth intervals (e.g., 0–10, 10–20 and 20–30-cm increments). Once live, dead, and size fractions are separated, dried, and weighed, then root productivity calculations can be undertaken using two primary approaches: the Decision-Matrix approach (of which the Smalley approach is commonly used for wetlands, Stagg et al. 2017b) and the Maximum-Minimum approach (Nadelhoffer and Raich 1992). These calculations have been detailed previously (Brunner et al. 2013), but it is important to note here that rates of bNPP using the different calculations (applied to the same data) provide different bNPP rates in g C m^−2^ yr^−1^ (From et al. 2021). It is important to pay close attention to study specific assumptions of each calculation relative to the wetland of interest.

*Root ingrowth bags:* A second empirical technique that is commonly applied in wetlands is the root ingrowth method (Lund et al. 1970; McKee et al. 2007; Castañeda-Moya et al. 2011; Cormier et al. 2015; Middleton et al. 2015; From et al. 2021). For this method, a nylon mesh bag is filled with soil devoid of roots (Fig. 14b). The bag can be constructed from any non-degradable polymer mesh (e.g., crawfish bag netting, vegetable mesh netting, canvas mesh; Fig. 14b). Infill substrate within the ingrowth bag can be sand, potting soil, peat moss, or native soil; ingrowth material ideally matches parent soil material as much as possible. The mesh size of the bag is usually large enough to allow for growth of rhizome tips and roots into the bag. Bias and error can occur from using soils with differing properties (e.g., bulk density) than native soils.

First, cores are extracted from soils (Section “Soil Collection”). Then the filled ingrowth bags are placed into the voids left from the extracted cores. A thin stick (e.g., skewer) inserted through the side of the bag and into the soil can be used to prevent them from floating. The ingrowth bags are left for a period of time, typically 6 to 12 months, and then collected (often during a relatively dry period for ease) and returned to the laboratory for analyses. The initially extracted cores (that made space for ingrowth bags) are processed for live and dead root biomass identically to procedures for serial coring described above. Values of bNPP are calculated using the incremental increase of biomass in the bags over the period of time. Data from root productivity using this technique can be compared to standing biomass of live roots determined from initially extracted cores to determine root turnover (yr^−1^) and longevity (yr) (McClaugherty et al. 1982, Table 6). Limitations of this approach include: 1) the large number of replicates necessary to identify changes over time; 2) root-growth stimulation associated with tissue damage; 3) access to a root-free fill material for the bag versus a root-dense medium, as would naturally occur; and 4) the potential underestimation of production due to the amount of time required for roots and rhizomes to extend into the bag.

*Mini-rhizotrons:* Another empirical technique for bNPP estimation uses in situ mini-rhizotrons. Mini-rhizotrons (Baker et al. 2001a, b) use transparent tubes inserted at an angle into soils and then an internal camera photographs change in root growth over time. Mini-rhizotrons can be very accurate in upland settings (Hendricks et al. 2006), but are more challenging to install and use in flooded soils (but see Iversen et al. 2012). Measurement of mini-rhizotrons requires specialized camera equipment and processing software, and track root demographic change over time to determine turnover and productivity.

*Carbon conversion factor:* Since data from calculations of bNPP are reported as g m^−2^ yr^−1^, conversion from plant biomass to C is still required to produce units of g C m^−2^ yr^−1^. While many scientists use a biomass to C conversion factor of 50%, the true site-specific conversion for roots is often lower (e.g., 0.38–0.47 in marsh and forested wetlands in South Carolina and Georgia, USA; Stagg et al. 2017a). Actual % C in organic matter biomass is ideally determined for each site using standard CHN elemental analyzer or LOI approaches on a sub-sample of collected roots to reduce uncertainty (Section “Soil Analysis - Bulk Density, Loss-on-Ignition, Elemental Analysis”).

*Indirect techniques:* Indirect techniques for assessing bNPP across larger areas include compartment flow or budget approaches (Publicover and Vogt 1993). These approaches are valuable in that they include most of the C processes to determine NEP that sum to or subtract from bNPP to produce C budgets that can be very site-specific (e.g., Krauss et al. 2018b). These indirect approaches, however, rely on calculations of missing components (usually based on aNPP data) in lieu of direct measurements for which variability in estimation is not well constrained. Thus, the area of bNPP inference with budget approaches would match the area of aNPP inference (e.g., 20 × 25 m plots). Among the approaches with high potential for application to wetlands is Total Belowground Carbon Allocation (TBCA) (Giardina and Ryan 2002), although this technique is typically applied in upland ecosystems. To apply TBCA, flux rate estimates (i.e., g C per unit time and space) are required for the following: soil CO_2_ flux partitioned into R_A_ and R_H_, lateral C export, CH_4_ efflux, C inputs from aboveground leaf, fruit, and twig litter associated with aboveground litter-fall, change in C content of root biomass (coarse + fine), and change in C content of the litter layer. The relative contribution of R_A_ (root respiration) and R_H_ to total soil CO_2_ flux can be determined using soil incubations with and without roots (Section “Laboratory Incubations”) or isotopic methods (Hanson et al. 2000), or from literature values. It is also necessary to determine the change in C content of mineral soil, but this component may be assumed to be zero in some wetlands given the difficulties of assessing this change accurately enough over the period of TBCA application, as well as its likelihood to be small. The theory, application, and detailed calculations for TBCA are available in Giardina and Ryan (2002). Comparisons of TBCA versus ingrowth and serial coring techniques in coastal wetlands indicate considerable differences in bNPP estimates among methods (From et al. 2021).

*Key Covariates and Ancillary Measurements:* Many of the covariates for biomass accounting (Section “Carbon in Wetland Vegetation”) also play an important role in assessing and interpreting rates of NPP across varied species composition, including abiotic environmental conditions such as soil properties and hydrological conditions (e.g., water table depth, soil moisture content, and salinity), and biotic influences such as grazing.

*Grazing:* Values of plant biomass and associate NPP calculations can be strongly influenced by grazing from birds, rodents, ungulates, or, in some cases, insects (e.g., Bertness et al. 2008; Davidson et al. 2017; Leffler et al. 2019). Protective enclosures can be placed around a subset of NPP plots or individual plants and compared to non-protected plots or plants to estimate the percent of plant biomass consumed by grazers (e.g., Finocchiaro et al. 2014; Shaffer et al. 2015).

#### NPP – Trees

**Fig. 15:**
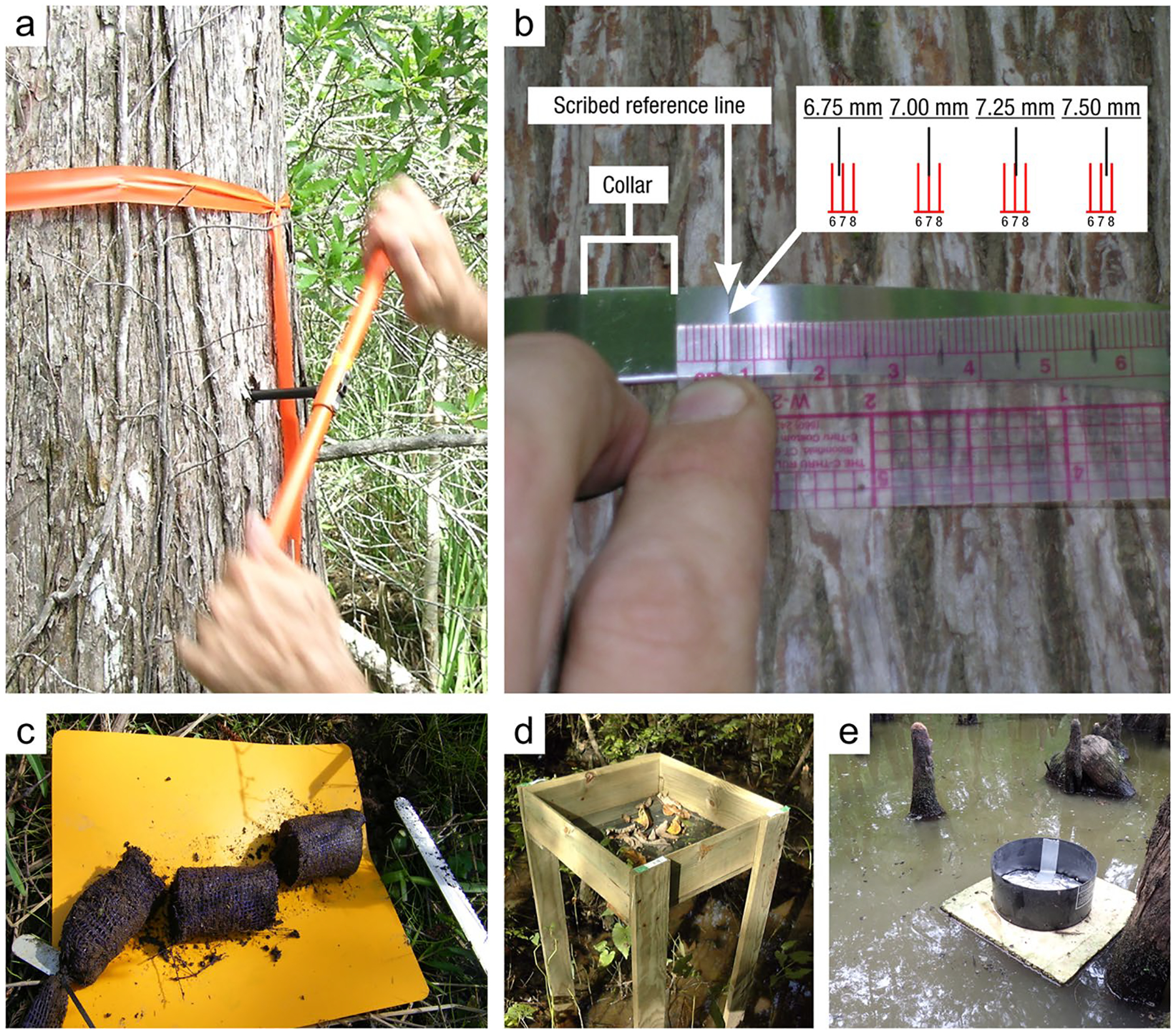
Methods to measure tree growth rates and net primary productivity: (**a**) tree coring using an increment borer; (**b**) dendrometer band measurements to the nearest 0.25 mm to determine radial growth rates; (**c**) root ingrowth bag divided into distinct depth intervals; (**d**, **e**) litter fall traps to estimate overstory aboveground litter biomass production. Images with permission from Jamie A. Duberstein (a, b), William H. Conner (d), Nicole Cormier and Andrew S. From (c), and Beth A. Middleton (e)

*What:* While harvest techniques may be practical or necessary for empirically measuring standing biomass of trees, NPP of woody plants is typically estimated using indirect approaches. Clark et al. (2001) did a thorough review of NPP studies and found most estimates of tree NPP were based on relatively few variables, primarily aboveground biomass annual increment and fine litter production (leaves and twigs < 1-cm diameter). However, more contemporary studies include a larger breadth of variables, including belowground information. Measurable components of NPP from trees include aboveground biomass of wood and foliage, the stump, as well as coarse and fine root growth (Fig. 15). Detailed NPP accounts will also include C losses incurred during the interval between sampling events, including fine litter (leaves and twigs), herbivory or other consumption, fine root turnover, as well as root exudates and C export to symbionts.

Estimates of tree aboveground and coarse root biomass generally involves allometric relationships (Section “Biomass - Trees”) applied to data collected before and after some time interval. Clark et al. (2001) summarizes two general approaches used to calculate tree NPP. The first approach involves tracking aboveground biomass change of marked individuals that met a minimum dbh requirement (e.g., 10 or 12.7 cm), and then summing biomass change across all surviving trees between time intervals, plus adding the biomass increment of new trees meeting the minimum dbh requirements since the previous survey; dead trees are not included in this first approach. The second approach uses total aboveground biomass before and after an interval without tracking individuals, though ingrowth and known mortalities over the time interval are necessary inputs that are incorporated into the estimate of NPP. Clark et al. (2001) emphasizes that failure to include mortalities in the second approach can vastly underestimate NPP for the interval in which mortality occurred; measures of mortality are logistically difficult for studies that re-measure over long time intervals (e.g., > 10 years).

Plot-based NPP studies can be complimented by installing dendrometer bands around the trunk at dbh (1.3 m) on a sub-sample of the tree population (Fig. 15b; a detailed description of dendrometer bands is provided below). After a brief settling period following installation, dendrometer bands expand and contract with the tree, allowing researchers to measure shrinkage and swelling with higher accuracy than attainable using standard forest survey tools such as d-tapes and calipers (Keeland and Sharitz 1993; Ford et al. 2016). The precision of the dendrometer bands allows for more accurate estimates of NPP phenology, making them a useful tool for a variety of studies involving tree growth rates in forested wetland systems (see Conner and Cherry 2013; Ford et al. 2016).

*Where:* The same criteria used to determine location and plot design for tree biomass, described in Section “Biomass - Trees”, can be used for NPP assessment. NPP can be calculated for any forested wetland, provided there are known relationships between the species’ size (e.g., dbh, height) and biomass.

*When:* Tree measurements for NPP are often first conducted during the growing season to facilitate identification of species, while subsequent annual measurements are made during the winter (if logistically feasible) when deciduous trees are dormant and evergreen trees are less active. Individual trees typically vary little in size during dormancy, remaining stable or shrinking a small amount, thus less biased by seasonal variation and allowing greater flexibility in scheduling the measurements. Tree tops are also easier to visually see during dormant seasons with less obstructions from deciduous leaves, increasing accuracy of tree height measurements. If NPP is sampled at shorter intervals (e.g., weekly or monthly) using dendrometer bands, it is important to assure the bands are read in the same order and time of day for each visit to minimize effects from diurnal shrinkage and swelling of tree diameter (Conner et al. 1981).

Growth of mangroves and tropical forests may not always follow seasonal patterns like trees in higher latitude climates with distinct growing seasons (Chowdhury et al. 2008). When annual variation in temperature and precipitation is low, mangroves display fewer intra-annual growth differences (e.g., no growth rings) (Krauss et al. 2007). Generally, mangrove and other tropical tree growth increments follow patterns in hydrological regimes (e.g., monsoons) or other external or endogenous factors (e.g., salinity Borchert 1992; Sánchez-Núñez and Mancera-Pineda 2011).

*Who:* Experience level required for tree surveys is outlined in Section “Biomass - Trees”. Dendrometer band installation requires some practice but can be readily accomplished by most field technicians. Experience working from boats is often needed for coastal sampling of mangroves.

How:

*aNPP:* The methods to assess tree NPP largely rely on repeated estimates of biomass of trees (Section “Biomass - Trees”) using allometric methods (e.g., Megonigal et al. 1997; Jenkins et al. 2003; Woodall et al. 2011). The approaches to assess NPP differ mainly by whether individuals are marked or not, and whether trees that died in the interval between repeat visits are measured as well. The primary data collected from trees is a measure of dbh, though some allometric relationships require height, percent cull, and other metrics as well. Tree growth of known individuals can also be measured using increment borers (Fig. 15a). The overall method of calculating NPP of trees ideally includes an empirical measurement of litter at the beginning of the interval (e.g., for year 1) and a measure of litter at the end of the interval, to account for any changes in productivity that would deviate from the expected foliar biomass provided by allometric calculations (Clark et al. 2001). Therefore, if allometric relationships used in estimating aboveground biomass include foliage, the best practice is to remove that component from the estimates of aboveground biomass increment and instead use empirical leaf litter data (Clark et al. 2001).

*Dendrometer bands:* Dendrometer bands are simple tools that custom fit around tree trunks (or baldcypress knees, see Anemaet and Middleton 2013) for purposes of measuring circumference change, which is transformed to diameter change, and then to either change in NPP or basal area (i.e., the amount of an area occupied by tree stems in a plot). Dendrometer bands are made from commercially available stainless-steel banding resembling wire-ties (some use aluminum bands, but those are more prone to thermal expansion, rust, and breakage), measuring tape, collar (also called ‘buckle’), and springs. Dendrometer bands can usually be installed in about 30 min (Fig. 15b) (Anemaet and Middleton 2013). Methods of dendrometer band construction often date to Hall (1944) and Liming (1957) with a helpful diagram in Cattelino et al. (1986). The traditional method involved etching Vernier lines onto the dendrometer band, but modern applications instead etch a single line next to the collar with a knife and measure to 0.25 mm accuracy with a small ruler (Keeland and Sharitz 1993). Prior to installation, the main trunk of the tree is typically lightly smoothed with a fine rasp or shaver (e.g., Model 21–115, Stanley Black and Decker, Inc.) to remove coarse bark, lichens, and moss to reduce the time necessary for dendrometer bands to become properly secured and responsive to changes in trunk diameter; it is critical not to injure the inner bark of the tree while smoothing the bark.

*bNPP:* Methods to measure belowground productivity are the same as those described in Section “Biomass - Herbaceous Vegetation”, such as serial soil collection and using root ingrowth bags.

*Litterfall:* Litter traps (Section “Biomass - Trees”) can be placed around NPP plots to catch fallen leaves or stems from overstory shrubs and trees (Fig. 15d, e). Litterfall data can be used to estimate levels of aboveground production over a period of time (e.g., annual, season). Such data are helpful to determine the response of species or relative health of trees to various environmental conditions if litter traps are placed across environmental gradients (e.g., water availability or salinity) (Middleton et al. 2015).

*Key Covariates and Ancillary Measurements:* The same key covariates and ancillary measurements for herbaceous vegetation apply to trees, including abiotic environmental conditions such as soil properties, hydrological regimes, and water chemistry. Due to the long-lived nature of trees, longer-term climatic information is also useful for understanding and modeling NPP, as well as historical disturbances (e.g., fire, insect outbreaks) or anthropogenic effects (e.g., tree planting, harvest).

### Carbon Accumulation in Wetland Soil

#### Definitions and Units

*Definitions:* The net exchange of C into and out of wetlands typically leads to accumulation of organic C in their soils (Bridgham et al. 2006). Sources of organic matter include autochthonous (internally produced) or allochthonous (externally produced and deposited) inputs (e.g., Hupp et al. 2019). Autochthonous C inputs into soils are generally dominated by belowground inputs from roots and rhizomes, but can also include some aboveground inputs from plant litter that gets incorporated into soils. In wetlands, belowground production tends to be concentrated in the surficial, top ~ 20 cm of soil (i.e., the ‘rooting zone’), although contemporary root growth has been found at depths of up to a meter or more below the surface (e.g., Kelleway et al. 2017). Allochthonous C inputs include deposition of organic matter attached to mineral sediment from the surrounding landscape outside of the wetland, including from hydrological, aeolian, and animal inputs. Not all C inputs accumulate in wetland soils, with much C lost to the atmosphere as CO_2_ or CH_4_ through myriad decomposition processes (Section “Greenhouse Gas Fluxes”; Bridgham 2014) or exported to downstream ecosystems through lateral flux of particulate, dissolved, and gaseous C (Section “Lateral Flux”; Webb et al. 2019; Bogard et al. 2020a).

While accumulation of soil C can include both organic and inorganic compounds, most studies focus on the organic fraction to estimate changes in SOC. The role of inorganic C on soil C accumulation is less clear compared with organic C. Some studies have shown that the contribution of inorganic C is minimal (e.g., Drexler et al. 2009; Bernal and Mitsch 2013), while others have shown that inorganic C is an important component of total C accumulation (e.g., carbonate precipitation, see Section “Carbon in Wetland Soils” (soil C pool) and 3.B (water C pool); Saderne et al. 2019; Wang et al. 2019b; Ouyang and Lee 2020). Thus, many studies focus on total C to include both organic and inorganic factions.

There are a variety of methods that are used to estimate accumulation of soil C in surficial and belowground C pools (Table 7). Broadly, these methods can be divided into two groups: 1) approaches that directly assess the C accumulation rate (CAR) based upon measurement of soil C mass that has accumulated over a specified area and timeframe; and 2) approaches that measure the sediment accretion rate (SAR) based on the vertical change (cm) of sediment over a known time, also commonly referred to as ‘sedimentation rate’. Measures of sedimentation are inclusive of organic matter plus mineral components, to which a C content conversion factor is often applied to determine CAR. SAR is often measured to answer broader ecological questions about sediment dynamics. Note: sediment build-up can also be measured in units of mass (g) and referred to as the ‘sediment (or soil) accumulation rate’, which is also abbreviated as SAR (see below *Units* for more details on CAR and SAR terminology).

Methods also vary in their temporal and vertical scale of inference (i.e., depth of substrate that they integrate), with estimates of CAR and SAR likely to be sensitive to the method and timeframe used (Breithaupt et al. 2018). For example, methods that evaluate dated sediment cores usually cover longer time periods and greater depths (decades to millennia, > 0.5 m depth) than those which assess contemporary wetland surface processes (days to years, cm depth). Methods also vary as to whether they capture only surface C fluxes (e.g., surface deposition) or sub-surface C fluxes (e.g., deeper root growth, subsidence, long-term decomposition) (Lamont et al. 2020). The choice of approach to be used should therefore be based upon the type and timeframe of information that best suits the objectives of the study. The sampling approaches described in this section, whether shallow or deeper, are generally conducted over a very small spatial scale (from cm^2^ to a few m^2^), with implications for the consideration of sampling design and spatial representativeness.

*Units:* There are many metrics to assess build-up of C in wetlands soils over space and time. The build-up is typically referred to as ‘accumulation’ or ‘accretion’. In general, ‘accumulation’ refers to increase in mass per unit area and time (e.g., g m^−2^ yr^−1^), while ‘accretion’ refers in increase in vertical distance (i.e., elevation change) per unit time (e.g., cm yr^−1^). However, ‘accumulation’ and ‘accretion’ are used synonymously in the literature, as are ‘soil’ and ‘sediment’, therefore it is important to check how terms are specifically defined for any given dataset, report, publication, etc. The terms ‘mass accumulation rate’ and ‘vertical accretion rate’ are often used for greater clarity.

When defined on a mass basis, SAR and CAR are typically measured as g m^−2^ yr^−1^ or g C m^−2^ yr^−1^, respectively. In some cases, fluxes are measured and reported over shorter, sub-annual time scales (Table 7). CAR is sometimes called the ‘apparent rate of C accumulation’ (aCAR), because it measures the *net* sum of inputs and losses, therefore it does not provide a full picture of C dynamics with time. It is also useful to differentiate *surface* CAR from *total* CAR, the latter of which will also incorporate sub-surface C inputs via root in-growth and organic matter exudation.

The term ‘C sequestration’ has been associated with a broader range of definitions including C uptake in plants (month, years), C burial in recent soil deposits (decades, centuries), and/or as longer-term C burial and preservation in deeper soils (millennia; Mitsch et al. 2013; Howard et al. 2014; DeLaune et al. 2018; Van de Broek et al. 2018; Windham-Myers et al. 2019).

When defined based on vertical/elevation change, SAR and CAR are often measured in cm yr^−1^. Note: CAR is not often reported based on vertical change in the literature. However, if there is a known volume per unit mass of organic or mineral matter, one could calculate how much of the overall vertical accretion is due to organic matter versus mineral matter (see, for example, Neubauer 2008; Morris et al. 2016).

**Rationale:** Globally, wetlands are estimated to sequester 0.7 Pg C year^−1^ as organic matter (~ 6% of anthropogenic emissions; Temmink et al. 2022). Peatlands, salt marshes, and mangroves can sequester C at some of the highest rates of any ecosystem, with mean rates up to 200 g C m^−2^ yr^−1^ (Temmink et al. 2022). The measurement of CAR in wetland soils has been widely used as a method of assessing the potential long-term C sequestration capacity of various wetland ecosystems. Soil CAR is a key parameter in C accounting/credits methodology (Needelman et al. 2018). Further, only autochthonous, organic material typically meets additionality constraints in most C accounting protocols. For management and scientific inquiries, measurement of CAR may also be useful to investigations of wetland and catchment geomorphology and paleo-environmental reconstructions.

#### Surficial Deposition

*What:* Deposition of C to the soil surface is an input flux from a variety of materials, compounds, and sources. Deposited material can include wood, intact leaves, phytoplankton, animal detritus, partially decomposed organic matter, carbonate, and C attached to mineral sediment, which includes a range of sizes from coarse woody debris to fine particulate organic matter and colloidal material. Surficial C inputs can be eroded and exported into and out of the wetland by lateral flux. Surficial C inputs tend to be relatively labile organic matter that experience fluctuating redox conditions from repeated wetting and drying, leading to a large portion of the material being lost through decomposition processes. Therefore, the duration of measurement influences the estimate of the surficial C input flux because some materials decompose faster than others. Once the rate of material deposition is assessed, it needs to be analyzed for C content to calculate CAR. The source of deposited material can be determined through geochemical fingerprinting (e.g., stable isotope ratios of δ^13^C and δ^15^N) to ascertain the proportion that is derived from allochthonous versus autochthonous sources (Craft et al. 1988; Van de Broek et al. 2018; Hupp et al. 2019), which is useful for interpreting C budgets and accumulation rates.

*Where:* The criteria to choose a location for assessment of surficial C inputs is similar to those for measuring soil C pools (Sect. 2.A). As always, choice of sampling locations depends on study objectives. Sampling to estimate site-level average flux rates may rely on stratified random approaches that account for the relative size of different zones with differing C inputs and flux rates, such as differences in elevation, vegetation, or soil types. Alternatively, if multiple measurement locations per zone are not feasible, then a measurement location that is either randomly chosen or identified as representative of the entire site is often chosen. Sampling to identify the drivers of variation in surficial C input flux may instead focus on spatial gradients along hypothesized controls of deposition such as water, sediment, nutrient, or ion loading gradients. For example, surficial C input flux could be measured at regular sampling distances along a transect perpendicular to a stream channel to test for the effects of differences in hydrological connectivity on CAR.

*When:* The timing and duration of measurement often depends on logistical constraints, and commonly range from an individual tidal cycle, to daily, weekly, monthly, annual, or several years. Longer durations of measurement are subject to more post-depositional decomposition of the deposited C and therefore tend to show lower average SAR and CAR (Sadler 1981) than shorter term measurements.

*Who:* These methods require a moderate level of experience to deploy, measure, and sample deposited material without causing disturbance. More expertise is needed to conduct laboratory analyses of deposited material.

**Fig. 16:**
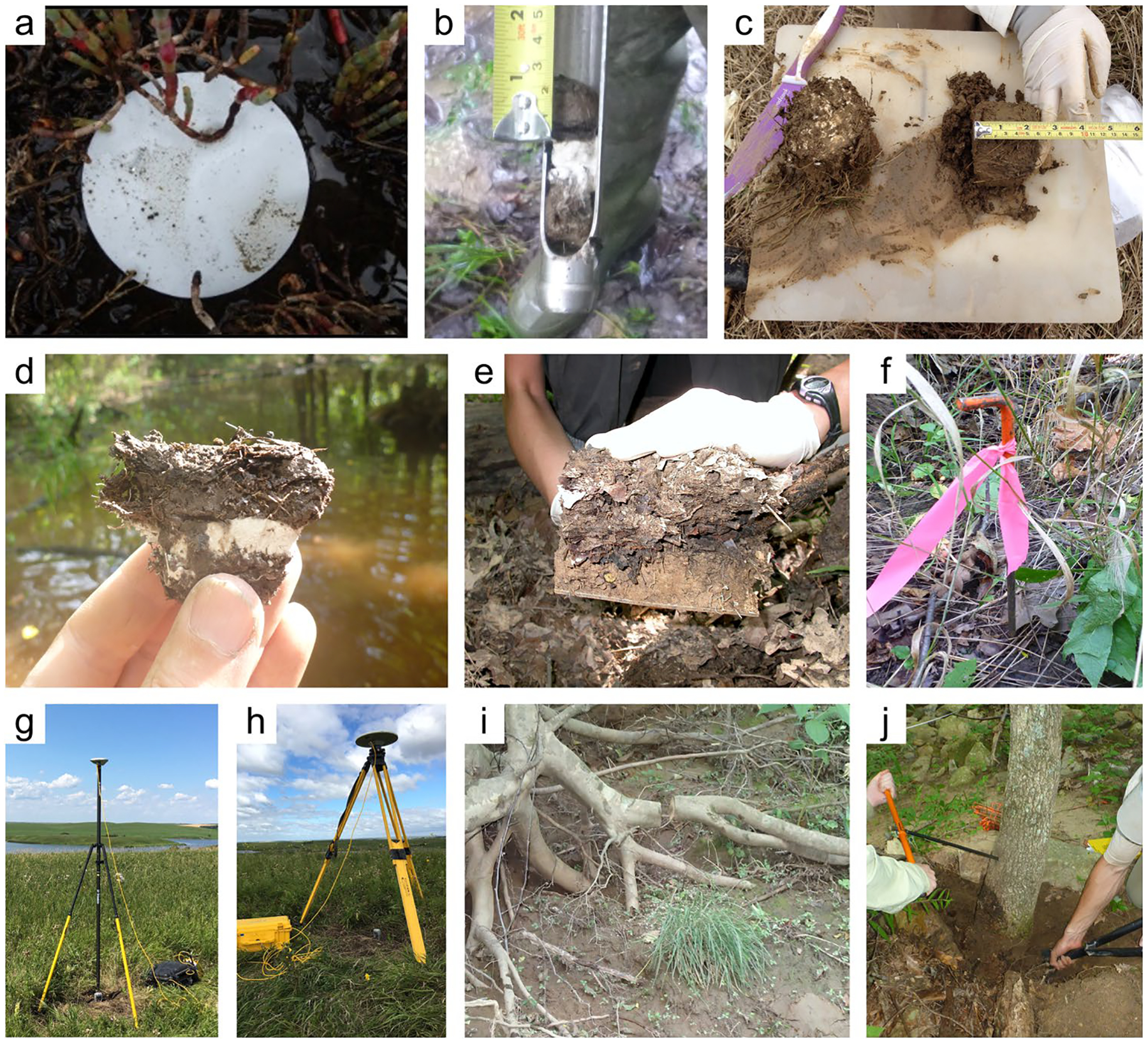
Common methods for assessing surficial soil deposition or erosion: (**a**) glass fiber filter paper sediment trap collecting tidal deposits of sediment and plant detritus in a saltmarsh; (**b**) measuring deposition above a feldspar marker using a soil probe; (**c**) processing a core above a feldspar marker horizon; (**d**) slice of soil showing feldspar marker overlain by accumulated sediment; (**e**) sedimentation tile overlain by sediment; (**f**) vertical pin; (**g**, **h**) surveying an elevation benchmark using a real-time kinematic global positioning system (RTK-GPS); (**i**) dendrogeomorphic bank erosion; and (**j**) dendrogeomorphic vertical change to assess erosion and deposition around tree roots and stem, respectively. Images with permission from Jeffrey Kelleway (a), Greg Noe (b, c, d, e, f, i, j), and Brian Tangen (g, h)

*How:* There are several common methods used to assess surficial deposition or erosion (Fig. 16). Some methods specifically measure vertical deposition, while others integrate vertical and lateral deposition. All methods described below are considered SAR methods, which require application of C content conversion factors, ideally obtained from each SAR measurement location. If site-scale or generic estimates of % C for conversion cannot be avoided, then it is crucial that appropriate existing estimates of % C from the literature are used. For example, the use of a % C conversion factor from predominantly organogenic settings or depths would lead to an overestimation of CAR if applied to a minerogenic setting.

For all SAR methods, great care must be taken to avoid (or limit as much as possible) disturbance of sediments or traps and surrounding areas during monitoring and collection. Depending on the objectives of the study, it may be decided to remove certain types of material (e.g., roots, coarse litter or sediment, crab-excavate, artificial debris) or to remove traps that show obvious signs of disturbance, prior to analysis.

*Sediment traps, filters, plates:* Sediment traps are used to measure deposition of sediment within the water column (Fig. 16a). These collection vessels have an open orifice for collecting falling sediment. The mass of sediment collected, the area of open orifice, and the duration of deployment are used to calculate an aquatic sedimentation rate. The efficacy of different designs of traps depends on the hydraulics of the field setting (Butman 1986). Many examples of designs and materials are available in the literature that are optimized for specific field settings and study goals (Table 7). Most often they are constructed from readily available laboratory or hardware supplies, but manufactured versions are available for open-water samplers.

*Marker horizons:* A marker horizon is an artificial layer that is placed on the wetland sediment surface to assess deposited sediment above the layer (Fig. 16b, c, d). Naturally or anthropogenically created horizons (e.g., deposits from extreme storm events or disturbed soils in plow layers, respectively) can also be used to estimate SAR and CAR (Lowe 2011; Fenstermacher et al. 2016; Drexler et al. 2019; Stolt and Hardy 2022; Section “Radiometric and Stratigraphic Dating - Laboratory Techniques”). Depending on the study objectives and the durability of the marker horizon, the depth of sediment deposited above the marker horizons may be measured repeatedly over time and/or completely harvested at the end of the measurement period. Typically, samples are collected by removing a portion of the marker horizon with accumulated sediment by coring (e.g., cylindrical corer or soil probe), such as with feldspar clay marker horizons, or cutting with knives, and then measuring the depth of sediment above the marker horizons (Fig. 16b, c, d). Sampling or sediment removal is ideally conducted during a period without surface water present above the marker horizon. After careful removal of sediment above the marker horizon, the sediment is dried and weighed to calculate a SAR based on the area of collected sediment (e.g., the inside diameter of the corer) and duration of deployment. The % C and dry bulk density of the deposited sediment (or nearby material at similar depths) are measured to calculate CAR (Section “Soil Analysis - Bulk Density, Loss-on-Ignition, Elemental Analysis”).

Commonly used marker horizon materials include white feldspar clay, brick dust, tiles, rubber pads (Lacy et al. 2020), and AstroTurf (Thomas and Ridd 2004). The materials have widely differing roughness, which influences deposition processes, and should ideally be matched to the density of vegetation above the soil surface. For example, locations with high stem or leaf density near the soil surface could use AstroTurf, whereas feldspar or tiles may be better suited for forested understories. Feldspar clay does not congeal to form an effective marker horizon in very dry conditions or where water is flowing (in these situations, a temporary baffle can be used such as a large open cylinder to restrict water flow). Aboveground vegetation is typically removed from the footprint of AstroTurf or tile installation, whereas feldspar clay can be added around vegetation stems and leaves and also allows new stem growth through the marker horizon. However, marker horizons can only be used to measure net deposition and cannot be used in a location that is experiencing net erosion (e.g., net erosion causes the loss of the feldspar marker horizon or a zero measurement on a tile), generating an overestimate of SAR or CAR in areas of wetlands that are not uniformly depositional. ‘Hard’ marker horizons such as tiles may reduce or prevent localized erosion that would otherwise occur, biasing net deposition rates. If the sampling location is always inundated, then sediment traps, filters, or plates are recommended. If the wetland has many erosional locations, then benchmarks, pins, or dendrogeomorphology (described below) is recommended.

*Reference benchmarks:* The reference benchmark method measures change in the elevation of the wetland sediment surface relative to a fixed benchmark. This can be implemented in a variety of approaches, most commonly including shallow pins (e.g., metal rods such as rebar, or plastic poles) placed into the wetland soil (Fig. 16f), or through repeated elevational surveying of the soil surface over time (Krauss et al. 2003; Kumara et al. 2010; Potouroglou et al. 2017). Vertical pins are placed into the soil to anchor them in place depending on substrate stability. The vertical difference from the top of the pin to the sediment surface is measured over time. In locations with high deposition rates, magnetometers are useful for locating buried metal pins. Repeat surveying can include using a total station (i.e., surveying instrument) to compare the elevation difference of locations in a wetland to a fixed surveying benchmark, or by using RTK GPS with very high vertical accuracy (Fig. 16g, h). Dendrogeomorphological techniques are used to measure the vertical change in the wetland surface elevation by comparing the current surface elevation around a tree to that of the primary basal root collar of the tree that is assumed to represent a fixed vertical position from which the tree germinated (Fig. 16i, j) (Hupp et al. 2016; Noe et al. 2022). These reference benchmark techniques are able to estimate both net erosion and deposition rates. Vertical rates of change can be converted into a flux rate by applying estimates of the C density of the accumulating or eroding material (such as surficial soil cores taken to the depth of change). These approaches also can be used to estimate lateral geomorphic flux of C; for example, lateral erosion of salt marsh or floodplain wetland sediment can be estimated by placing horizontal pins located along eroding edges. The net balance of vertical and lateral deposition and erosion is a fundamental characteristic of the sediment and C balance of wetlands (Noe et al. 2022).

**Fig. 17:**
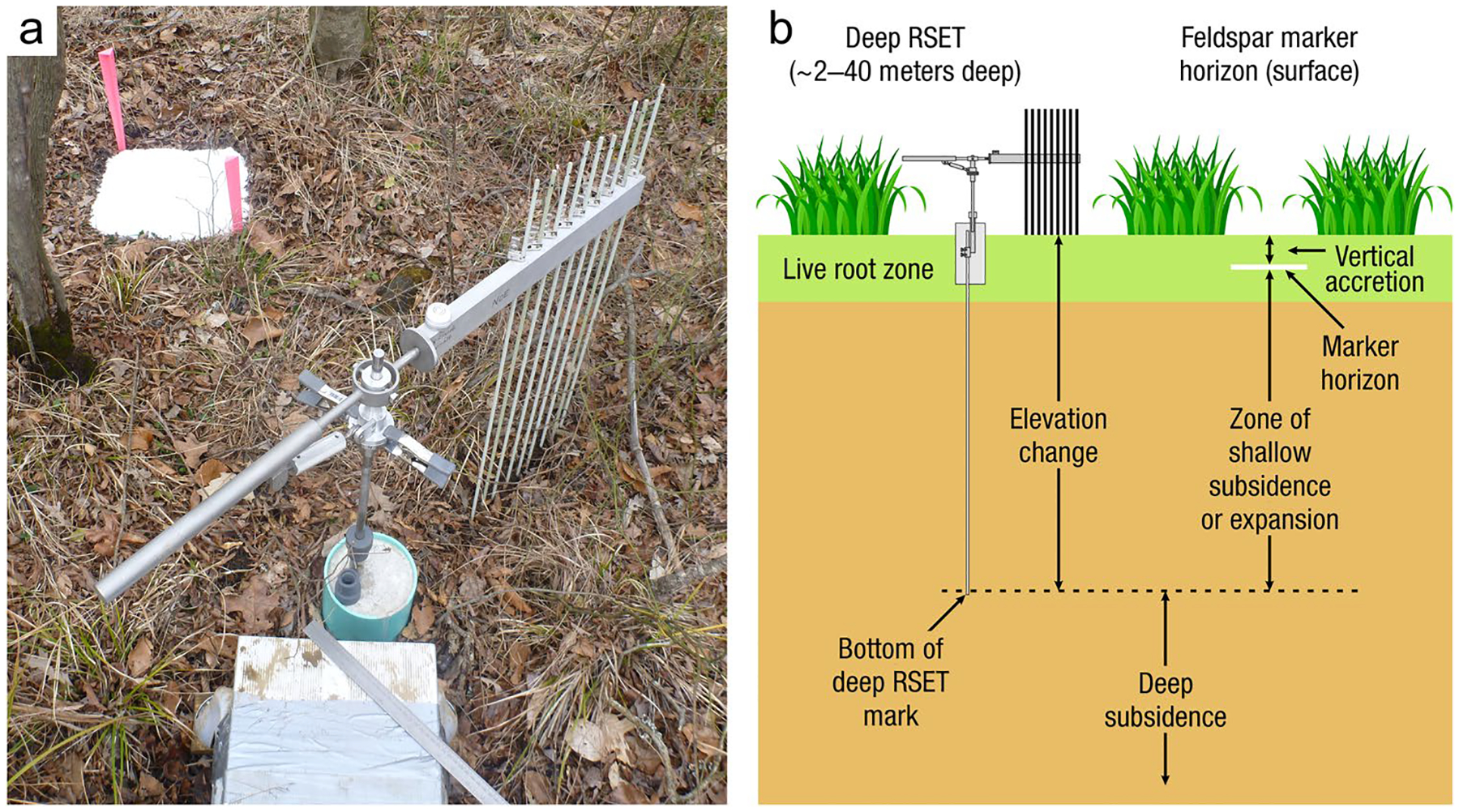
(**a**) An example of a surface elevation table (SET) with a feldspar marker horizon (top left of photo); (**b**) a conceptual diagram of rod SET system (RSET) with a marker horizon (Lynch et al. 2015). Image with permission from Gregory B. Noe (a) and James C. Lynch (b)

*Surface elevation table (SET):* A SET is a mechanical leveling device to non-destructively measure the vertical movement of wetland sediment surfaces (i.e., elevation change) over a period of deployment (Fig. 17), which can be converted into a CAR by applying estimates of the C density of the soil (Lovelock et al. 2014; Cormier et al. 2022). Like the other SAR approaches, using SETs to estimate CAR requires careful measurement of the C density of soil that matches the soil depth of SAR measured over the study. Surface elevation tables have been used around the world to assess rates of vertical change among different wetland types and contrasting environmental settings, as well as to understand how different management treatments affect wetland surface elevation change (Saintilan et al. 2022). When records of surface elevation change are sufficiently long compared to rates of sea-level rise for a region, submergence vulnerability of the wetland can also be determined (Cahoon et al. 1995; Webb et al. 2013; Breithaupt et al. 2018) using various analytical techniques (Russell et al. 2022).

One important advantage of SETs is that both negative values (equating to soil C loss) and positive values (equating to soil C gain) are possible to measure using this technique. Pairing SETs with marker horizons allows researchers to separate vertical accretion from surface elevation change rates, thus accounting for the effects of shallow subsidence, compaction, erosion, and root zone expansion. However, the specific depths that are subsiding, compacting, eroding, or expanding in the subsurface can be difficult to determine, therefore assumptions about the source of accretion (surficial depth or root expansion) and any compaction losses (or not) can bias these results.

The SET includes a constant reference plane attached to a shallow or deep benchmark from which the distance to the sediment surface can be measured using pins lowered to the sediment surfaces (Fig. 17) (Geoghegan et al. 2008). SETs paired with marker horizons are typically placed singly or in replication to represent a wetland site. However, since each SET typically has a limited number of associated marker horizons, the estimates have limited spatial scope of inference compared to similar techniques using only marker horizons with greater spatial replication (Noe and Hupp 2005). Repetitive RTK GPS surveys of SET rod receivers allow for estimation of deep subsidence from the same locations (Cahoon 2015). The difficulty and expense in installing and measuring SETs limits replication of SETs per wetland site. Because SET estimates vary between technicians, it is useful to have the same individual make the measurements at each SET site. The SET-marker horizon technique has transitioned from using pipes to rods (Rod SET; Cahoon et al. 2002) using universal installation standards, which enables comparisons to tidal gauge measurements for vulnerability assessment of sea-level rise (Cahoon et al. 2002; Cahoon 2015 for additional measurement details; Russell et al. 2022 for analytical techniques).

*Key Covariates and Ancillary Measurements:* When measuring surface deposition, many of the key covariates and ancillary measurements are similar to those for soil sample collection and analysis (Section “Carbon in Wetland Soils”). These generally include a good description of each sampling location such as vegetation and hydrology, as well as information on the location of sampling using RTK GPS or a detailed map. While the methods described above allow a researcher to determine the rate of surface deposition, it is also important to know the source of the deposited material. Geochemical and biological analyses of stable isotope ratios (e.g., δ^13^C, δ^15^N, Section “Soil Analysis - Bulk Density, Loss-on-Ignition, Elemental Analysis”), compound specific analyses (e.g., lignin ratios, for lignin analysis; Section “Litter and Organic Matter Decomposition”) or environmental DNA might be used to determine the extent to which sediment C pools represent allochthonous versus autochthonous sources. Particle size analysis (i.e., quantification of the proportion of different mineral size classes) allows better understanding of the depositional environment and changes in SAR or CAR over time (Section “Soil Analysis - Bulk Density, Loss-on-Ignition, Elemental Analysis”).

#### Repeated Measurements of Soil Carbon

*What:* Repeated measurements of the soil C pool in a given location is a robust method to estimate a CAR over time. This approach involves re-visiting and re-measuring soil C at previously sampled locations. One of the major benefits of the repeated measurements approach is that it integrates C gains and losses throughout a soil profile. For example, in some wetlands, production of new C via root growth is a significant source of SOC accumulation that is unaccounted for by surface accumulation methods using marker horizons, but can be captured by a repeated measurement approach (Lamont et al. 2020). The slow rate of soil C accumulation makes this approach most effective if there are many years between sampling events; however, sampling over years to decades is often beyond the scope of most studies. There is also the potential to incorporate the repeated measurements approach in experimental designs of new studies, particularly if establishing long-term sampling plots.

*Where:* The repeated measurements approach can be applied at wetland sites with previous soil C measurements (Section “Carbon in Wetland Soils”; Lamont et al. 2020). The most significant constraint is the presence and availability of reliable historical data on locations that previous samples were collected, which may or may not be publicly available. The high level of spatial variability in soil C pools within wetlands may present a particular challenge to this method. That is, sufficient spatial replication during each sampling event is needed to ensure measured differences in soil C can be ascribed to temporal change rather than spatial variability.

*When:* Historical data on soil C density are needed over a relatively long timeframe (e.g., years or decades earlier) to detect C accumulation that exceeds natural variation. Temporal variability in CAR can be assessed with multiple sampling events in a given location.

*Who:* Soil collection methods can be readily trained to technicians (Section “Soil Collection”). Repeated measurement studies may benefit from the involvement or advice of personnel who collected the historical data to ensure consistency of sampling approaches and locations.

*How:* Typically, coring methods are used to collect soil C and bulk density samples (Section “Soil Collection”). Depending on the objectives, other methods such as pits or monoliths may also be suitable for soil C and bulk density analysis (Lamont et al. 2020). Consistency in sampling methods for % C and dry bulk density among events is important to remove the influence of any biases introduced by using different methods.

Comparisons of soil C density over a specified depth range (e.g., top 30 cm) are typically used to infer CAR within that range. Alternatively, identification of a basal horizon (e.g., lithological, geochemical, or radiometrically dated) that is found within the soil profile on each sampling event can also be used to quantify changes in soil C over time, with C above the horizon assumed to be newly accumulated (Section “Radiometric and Stratigraphic Dating – Laboratory Techniques”).

*Key Covariates and Ancillary Measurements:* It is crucial to have accurate locational data for historical and contemporary sampling events for the repeated measurements approach. High resolution coordinates using RTK GPS is a preferred method (down to a minimum of 4 significant digits), though durable site markers may also be used to find previous sampling locations. Accurate information on the amount of time between sampling events is also very important for this approach. Site-scale environmental change data (e.g., vegetation surveys, hydrological data, land use) can help with understanding the mechanisms of change in soil C between sampling events.

#### Space-for-Time Chronosequences

*What:* Use of space-for-time chronosequences is an approach to examine short- and long-term ecological processes that progress relatively slowly. Short-term chronosequence studies typically represent annual to decadal time scales, while long-term studies often examine processes over centuries to millennia. The application of chronosequences relies on the assumption that spatial heterogeneity approximates temporal trends, and that soil samples of various ages (e.g., time since disturbance or deposition) represent different developmental or successional stages. The chronosequence approach typically involves estimating C density of wetland soils or soil core segments, and then developing statistical relationships (e.g., regressions) between age and C density. Short-term studies often define wetland age as the number of years since a wetland was affected (e.g., drained, restored, created) or formed through rapid sediment deposition in aquatic ecosystems (e.g., new delta progression). Long-term studies typically use age of wetland soil core segments that are dated using radiometric techniques (Section “Radiometric and Stratigraphic Dating – Laboratory Techniques”).

Chronosequences can be used to compare SOC content of restored wetlands of different ages, thereby providing annual estimates of SOC accumulation attributed to wetland restoration (Euliss et al. 2006; Badiou et al. 2011; Tangen and Bansal 2020). Chronosequences have also been applied to created mangrove wetlands of known age and soil C content in Florida, USA, to determine the rate of soil C burial as peats form following wetland creation (Osland et al. 2012, 2020). A similar approach can be used to estimate rates of SOC loss following wetland drainage, or after climate change-induced permafrost thaw. In boreal permafrost peatlands, quantifying SOC of intact permafrost plateaus and comparing them to SOC of a series of bogs that thawed at different points in time (multiple decades to centuries or millennia apart) has provided a useful metric for quantifying loss of permafrost SOC upon thaw (O’Donnell et al. 2012; Jones et al. 2017; Heffernan et al. 2020). This method also allows for the quantification of new SOC accumulation with time since thaw.

*Where:* For short-term chronosequence studies, selection of wetland sites with similar characteristics (e.g., hydrologic regimes) can help limit natural variability in soil C density among sites. For example, a restored wetland in a drier climate may have naturally slower accumulation rates than one in a wetter climate; therefore, differences in soil C accumulation between sites may be less attributable to time since restoration compared to climate. Similarly, for long-term studies, soil cores are more readily comparable with similar physical features such as landscape position, microtopography, and soil depth.

*When:* To the extent possible, wetlands or soil cores with known ages are uniformly distributed across the observed chronosequence to facilitate development of regression models. It is ideal to have an appropriate proportion of wetlands representing older sites due to the high variability and the relatively slow nature of C accumulation. Similarly, soil cores need to be deep enough to encompass the desired time scale.

*Who:* Technicians can be relatively easily trained to collect wetland soils (Section “Soil Collection”). Regression analyses and other statistical comparisons of wetlands or soil core segments distributed across a chronosequence require proficiency in the use of statistical programs and models. For complex study designs, or when the data display high variability, advanced statistical expertise may be required. For long-term studies, requisite expertise is required to estimate ages of soil core segments using radiometric dating techniques.

*How:* Soil collection procedures are the same as described in Section “Carbon in Wetland Soils”. Surficial soil samples (e.g., upper 0–30 or 0–60 cm) are often used for short-term studies, while soil cores for long-term studies typically are collected meters deep. Once ages and soil C have been determined, statistical models (e.g., regressions) are generated to determine rates of C accumulation or loss over time. Additional components of the C budget can be collected as well (Krauss et al. 2018b), depending on the objectives. The chronosequence method can be applied to vegetation biomass and composition as well.

*Key Covariates and Ancillary Measurements:* The key covariates and ancillary measurements for chronosequences are the same as for other soil collection and analysis methods (Sections “Soil Collection”, “Soil Analysis – Bulk Density, Loss-on-Ignition, Elemental Analysis” and “Carbon Accumulation in Wetland Soil”). Background information, including wetland classification, age (e.g., time since disturbance/management), current and historical climate, topographic characteristics (e.g., microtopography, slope grade), soil mapping unit or classification, soil properties (e.g., dry bulk density, texture, particle density, soil moisture, and ice content), peat composition and plant macrofossil determination, vegetation characteristics (e.g., composition, biomass), hydrological characteristics (e.g., hydroperiod, water chemistry), and land-use and management history are important covariates to understand causes of change in soil C.

#### Radiometric and Stratigraphic Dating – Laboratory Techniques

*What:* Core chronologies provide information on when layers of sediment were deposited and therefore are a necessary element to understanding rates of C accumulation in wetland sediments. Because sediments and peat accumulate stratigraphically (with the oldest coinciding with the deepest sediments and the youngest at the surface), researchers can calculate rates of C accumulation through time, and variability in rates can be tied to changes in environmental conditions, often driven by climate or land-use change. There are a suite of dating techniques that range from radiometric (i.e., radioactive isotopes with known half-lives) to other known stratigraphic markers, such as volcanic tephra, pollen, pollution markers, and human artifacts (e.g., Ricker et al. 2012). There are tradeoffs to every analytical method, so an investigator’s choice of which dating technique to perform depends on a number of considerations, including the temporal scale of inference (i.e., years to millennia), the ability to detect fine resolution information (e.g., changes in CAR due to individual events versus averages over time), the conditions that are appropriate for each method (e.g., permanently versus seasonally flooded wetlands), as well as logistical considerations (e.g., pre- and post-processing effort) and costs (e.g., radiocarbon dating is relatively expensive).

**Fig. 18:**
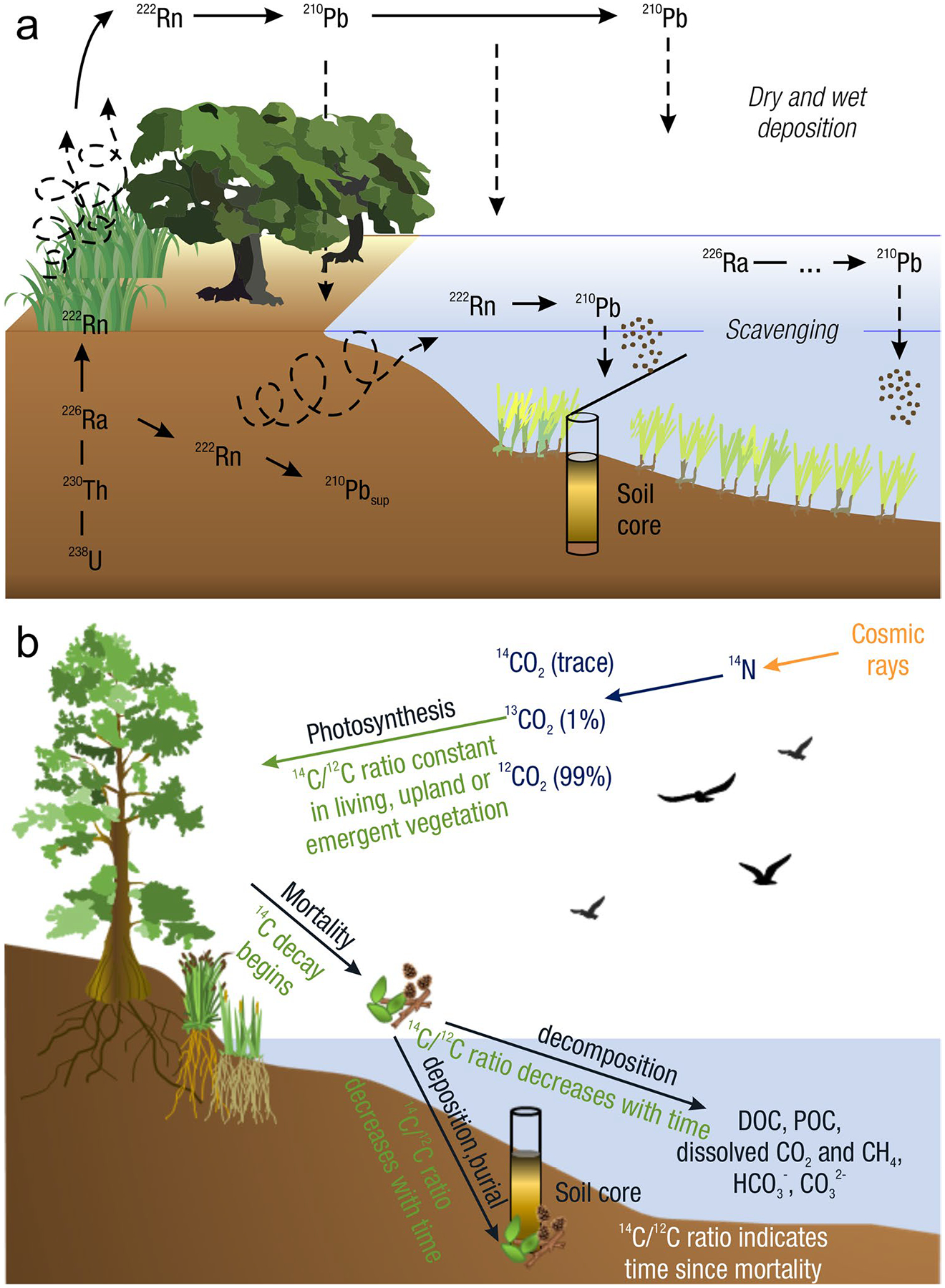
Conceptual diagram (**a**) of the lead (Pb) cycle as it decays from uranium-238 (^238^U), thorium-230 (^230Th^), radium-226 (^226^Ra), radon-222 (^222^Rn), to lead-210 (^210^Pb) from soils to the atmosphere and back down to wetland soils through dry and wet deposition (Arias-Ortiz et al. 2018); (**b**) conceptual diagram of the carbon-14 (^14^C) cycle where atmospheric carbon is incorporated into plants, which subsequently die, decompose, and are incorporated into soils or dissolved carbon species [^12^CO_2_, carbon-12 CO_2_; ^13^CO_2_, carbon-13 CO_2_; ^14^CO_2_, carbon-14 CO_2_; ^14^C/^12^C, ratio of carbon-12 to carbon-14; ^14^N, nitrogen-14; ^210^Pb_sup_, supported ^210^Pb; CH_4_, methane; CO_2_, carbon dioxide; CO_3_^2−^, carbonate ion; DOC, dissolved organic carbon; HCO_3_.^−^, bicarbonate ion; POC, particulate organic carbon]

*Radiometric dating techniques:* The underlying assumption of radiometric dating is that the parent nuclide or daughter product does not enter or leave the substrate after formation, allowing for a calculation of age based on the concentration and known half-life of the isotope in question (Fig. 18). Different radioactive isotopes are used for different timescales depending on the half-life of the isotope. Lead-210 (^210^Pb) and Cesium-137 (^137^Cs) are two of the most commonly used short-lived radionuclides to determine chronologies and sediment accretion rates for the past century (Appleby 2013). Carbon-14 (^14^C), also referred to as ‘radiocarbon’, is a longer-lived radioisotope used to date organic matter as old as 55,000 years (Reimer et al. 2020). Radiocarbon can also be used to date C on other C constituents in the water column (e.g., DOC) and GHGs (e.g., CH_4_), which can provide information on whether the source of C is from older versus younger, more recently fixed photosynthates.

^210^Pb occurs naturally and has a 22.23-year half-life (DDEP 2017), with the limit of application approximately 5 half-lives, or a little over a century (Fig. 18a). The source of ^210^Pb to sediments is ultimately the decay of parent isotopes within the uranium-238 (^238^U) decay series, which consists of several steps. The steps relevant to ^210^Pb start when radium-226 (^226^Ra), a ubiquitous element in soils, decays to radon-222 (^222^Rn), which escapes into the atmosphere, and then decays to ^210^Pb, which falls out of the atmosphere through wet and dry deposition. Once ^210^Pb enters sedimentary environments, radioactive decay is the only removal process, so the distribution of ^210^Pb within a core provides a chronometer (Baskaran 2011). Because most sediments contain ^226^Ra and produce ^210^Pb that is retained in situ, this background activity (referred to as ‘supported ^210^Pb’ in Fig. 18a) is also measured and subtracted to calculate the so-called ‘excess ^210^Pb’. It is also important to consider that ^210^Pb can mobilize vertically in wetland sediments (Urban et al. 1990), particularly peatlands (Vile et al. 1999), when interpreting results. ^210^Pb dating may not be appropriate if mobilization has occurred or sediment activity is too low for reliable measurement, as may occur in regions of low atmospheric deposition (Zhang et al. 2021a), in which case ^137^Cs may be preferred.

^137^Cs has a 30.17-year half-life and is a product of nuclear fission (Be et al. 2016). It is formed from uranium decay and the final product of the decay series is ^137^Ba. Unlike ^210^Pb which is continuously produced because of radioactive decay, ^137^Cs atmospheric deposition is declining. Its presence is primarily a byproduct of atmospheric nuclear testing that began in 1952 and peaked in 1963. The most common applications of ^137^Cs are identification of sediments deposited after the onset of thermonuclear testing in the 1950s, with the assignment of the year 1954 to the sediment depth of initial detection of ^137^Cs, and 1963 assigned to the sediment depth with a ^137^Cs peak. Accidents at nuclear power plants also release ^137^Cs, and the Chernobyl accident in 1986 produced a second ^137^Cs peak in European sediments. The actual peak, or peaks, vary by region as the fallout was affected by location relative to the event and by the weather during the fallout events. Vertical mixing of radioisotopes within sediments by physical (e.g., trampling) and chemical processes is also possible, which can cause over- or underestimation of CAR. Accuracy of the ^137^Cs dating method can be increased by incorporating the 1954 or 1963 dates with other dating methods like ^210^Pb (Drexler et al. 2018; Thorne et al. 2018; Creed et al. 2022), and vice versa for ^137^Cs to corroborate ^210^Pb.

^14^C has a relatively long half-life of 5,730 ± 40 years. ^14^C is produced as cosmic rays interact with atmospheric nitrogen to produce ^14^C (Fig. 18b). The basis for ^14^C dating assumes that after a plant dies and is no longer incorporating CO_2_ into plant tissue through photosynthesis, the ^14^C decay process begins, and the amount of ^14^C decreases relative to ^12^C. The decay rate of ^14^C is constant, so the ratio of ^14^C to ^12^C is used to estimate how much time has passed since the organism (typically plant) died and stopped taking up C. Because the ^14^C is not produced in the atmosphere at the same rate through time, ^14^C ages need to be calibrated to calendar ages (Reimer et al. 2020). Anthropogenic effects on the radiocarbon content in the atmosphere can complicate and/or be used as a tool for radiocarbon dating. CO_2_ produced through fossil fuel combustion is depleted of ^14^C because it is so old (‘radiocarbon-dead’); thus, the change in ^14^CO_2_ in the atmosphere has been used to distinguish natural versus anthropogenic sources of CO_2_ (e.g., Turnbull et al. 2009; Basu et al. 2016, 2020). Open-air nuclear testing in the mid-twentieth century also released radiocarbon in the atmosphere, resulting in a ‘bomb spike’ in atmospheric concentration of radiocarbon. While there is a calibration scheme for post-bomb ^14^C (e.g., CALIBomb, Reimer et al. 2004), the lack of constant production of ^14^C in the atmosphere renders the dating method harder to interpret in recently deposited sediments without additional chronological constraints, as dates will have multiple age possibilities.

Analysis of smaller samples, such as from terrestrial macrofossils and isolated pollen, uses Accelerator Mass Spectrometry (AMS) to measure individual C atoms by mass (e.g., ^14^C, ^13^C, and ^12^C) and provides precise dating of relatively small samples (10–100 mg). Macrofossils from aquatic plants are avoided for radiocarbon dating because atmospheric CO_2_ is not their primary source of C for photosynthesis. Bulk sediment (i.e., including pulverized roots and organic matter) can also be dated using the AMS method, but bulk sediment can contain both older and younger components of C, such as from roots (younger) or recycled C from the water (older), which can compromise the accuracy of dates from bulk sediment. The primary disadvantage of AMS dating is the greater cost, which may limit the number of dates obtained. Another challenge is finding suitable plant macrofossils in sediments or peats. Also, there are often logistical difficulties (i.e., requires specialist, time consuming) in cleanly separating samples such as pollen from other organic material of the same size to accrue sufficient sample mass for AMS measurements (Brown et al. 1989; Zimmerman et al. 2019; Tunno et al. 2021).

**Fig. 19:**
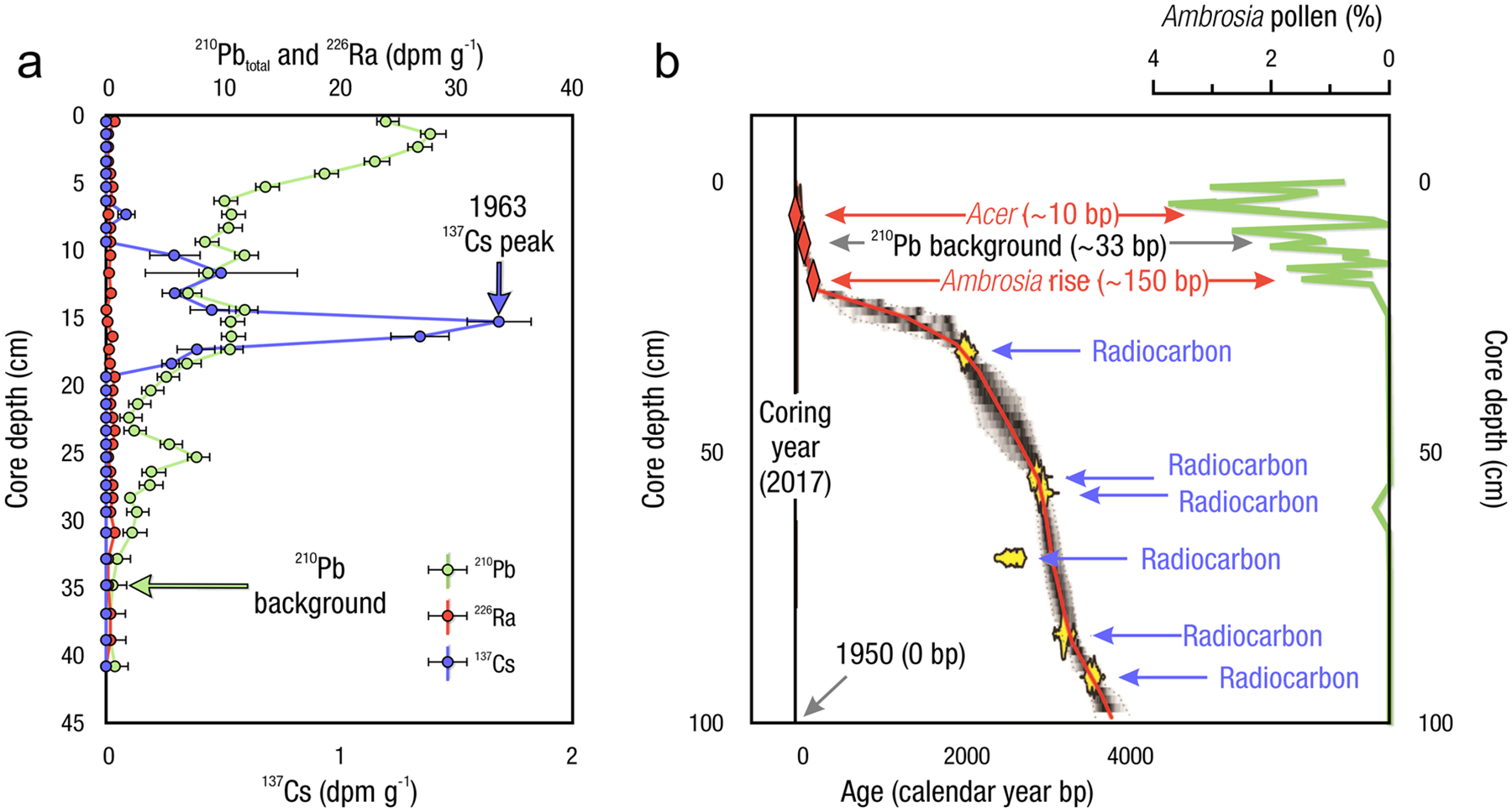
(**a**) Coastal wetland soil core depth profile of lead-210 (^210^Pb) excess (green), cesium-137 (^137^Cs) (blue), and radium-226 (^226^Ra) (red) from a sediment core collected in Sage Lot Pond, Waquoit Bay National Estuarine Research Reserve, Massachusetts, USA (Gonneea et al. 2019). ^137^Cs peak occurs in 1963 at ~ 15 cm depth, while ^210^Pb_excess_ reaches background levels (i.e., ~ 0.1 dpm g^−1^) by 35 cm where the ^210^Pb line meets the ^226^Ra; note very low ^226^Ra activity, which is common in organic soils; (**b**) age-depth model from a peat core collected in Great Dismal Swamp National Wildlife Refuge, Virginia and North Carolina, USA, integrating data from radiocarbon dates, ^210^Pb, and pollen biostratigraphy. Years on x-axis are in calendar years before present (bp), with 1950 as year 0. The vertical black line indicates the coring year (2017, − 67 bp). The green line represents % *Ambrosia* pollen. The red and yellow polygons are violin plots that show the probability-density functions for discrete age estimates. Red violin plots are based on pollen biostratigraphy showing a sharp increase in *Ambrosia* at 150 ± 20 years bp following colonial land clearance and an increase in *Acer* at − 10 ± 10 years bp following expansion of maple gum forests after canal construction, and a ^210^Pb estimate of 33 ± 20 years bp. The ^210^Pb age determination for this example was made by selecting the depth at which ^210^Pb reached background levels and assigning it an age of 100 ± 20 years before coring in 2017. Yellow violin plots are based on radiocarbon age estimates. The red line represents the best fit line. The gray shading indicates 2 standard deviations uncertainty associated with the age-depth model. The age-depth model was made using the Bayesian age modeling program Bacon (Blaauw and Christen 2011), inserting the ^210^Pb and pollen ages as calendar years and the radiocarbon dates in radiocarbon years, calibrated to calendar ages using the Bacon modeling program

*Stratigraphic dating tools:* Stratigraphic markers identify specific horizons in the soil profile (referred to as chronohorizons) and rely on knowledge of timing of land cover and vegetation changes, climate events, introduction of pollutants, or release of volcanic tephra into the sedimentary system. Pollen biostratigraphy is particularly useful in wetland sediments because of favorable preservation conditions, and pollen marker horizons can be dated using historical knowledge of anthropogenic modification of landscapes. For example, in eastern North America, increased abundance of ragweed (*Ambrosia*) in sediment cores is used as a marker of European settlement (e.g., agricultural horizon; Fig. 19b) (Brush 1984; Willard et al. 2003; Williams et al. 2004; Oswald et al. 2007). Introduction of non-native species, such as *Casuarina* in south Florida, USA (Langeland 1990; Wingard et al. 2007), and other human-related changes to vegetation (e.g., forest clear cutting) provide similar biostratigraphic markers (Chmura et al. 2001). Sediment layers in wetlands can be affected by erosion, deposition, and other processes that can alter the dating results. For example, pollen in systems such as coastal wetlands may be reworked (i.e., mixed with older materials) through tidal forces (Neulieb et al. 2013), which may produce an artificially old date. Therefore, knowledge of local geology, hydrology, and climatic events help interpret dating results and estimate the age of the sediment layer with greater certainty.

Deposition of pollutants from industry can provide additional chronohorizons if the timing of industrial pollution in a region is known. One such marker is the amount of total Pb in sediments (not to be confused with ^210^Pb), which began increasing after advances of the Industrial Revolution in the late nineteenth century, followed by further increases in concentrations with the introduction of leaded gasoline in 1921 (Fig. 19b) (Siver and Wizniak 2001). The elimination of Pb from gasoline in the mid-1970s reduced sediment concentrations greatly, and characteristic patterns of Pb concentration provide useful stratigraphic horizons for much of the twentieth century.

Tephra, or volcanic ash, from individual eruptions have unique chemical and mineralogical signatures. Geochemical analyses of tephra provide independent time horizons for areas with volcanic fallout. As tephra layers from specific volcanic events are identified, characterized, and dated, tephras with the same geochemistry and minerology can then provide an additional chronohorizon (Lowe 2011).

*Where:* The location criteria to collect cores for radiometric or stratigraphic dating are generally the same representativeness criteria as for soil C content (Section “Soil Collection”). However, there are some differences that should be considered. For ^137^Cs, peaks are not always distinct, particularly in wetlands that experience variation in redox conditions related to wet-dry cycles (Drexler et al. 2018). ^210^Pb deposition is greater downwind of continental landmasses, so eastern coasts in the northern hemisphere have better ^210^Pb data than western coasts (and vice versa for southern hemisphere) (Zhang et al. 2021a).

*When:* Unlike radioisotopes with short half-lives (such as Beryllium-7 [^7^Be] with a half-life of 53 days), the half-lives of ^137^Cs, ^210^Pb, and ^14^C are long enough to preclude the need for radiometric dating immediately after sample collection. Postponement of analysis for as long as a few years likely will not affect the results if properly stored to prevent secondary biological growth. Macrofossil, pollen, geochemical, and tephra sampling often occurs after core description, which can occur either immediately after collection or later if the core has been stored.

*Who:* In general, radiometric and stratigraphic dating methods require specialized equipment with highly trained personnel; thus, samples are often sent to laboratories dedicated to each method. Analysis of ^210^Pb and ^137^Cs requires laboratories with gamma or alpha counters and technicians trained in their use. Gamma analysis does not require any special chemical treatment, whereas alpha analysis involves chemical leaching with hydrochloric and nitric acids, requiring a laboratory and staff capable of handling such digestions. Radiocarbon dating samples are typically submitted to external laboratories that maintain the specialized equipment (such as AMS), standards, and protocols required for the analyses. Individual investigators select material to submit for radiocarbon analysis, often necessitating personnel trained in macrofossil identification. Preparation of pollen for either biostratigraphy or AMS dating (Mensing and Southon 1999; Neulieb et al. 2013; Zimmerman et al. 2019) requires a laboratory with capabilities for sediment digestion with hydrofluoric and other acids (Traverse 2007) and personnel trained in extracting pollen from sediments. Isolated tephra samples are typically sent to an equipped tephrachronology laboratory for analysis and interpretation.

*How:* The methods to collect and transport soils are described in Section “Soil Collection”.

*Core description:* After collection of the core, its lithology (physical characteristics) is described, including color, texture, and composition (e.g., peat, organic-rich silt, etc.), particularly noting depths at which transitions take place. A ‘good’ core for radiometric dating is one that does not have any signs of disturbance as seen visually, such as obvious inversions (e.g., where older sediment is mixed above younger), truncations (where erosion removes part of the sediment column), and inclusions (wood chunks) within the core profile. Discontinuities (gaps in deposition) may add to uncertainties when dating. Anomalous roots, stones, etc. may indicate a disturbed setting which may not be reliably dated or interpreted. Acceptable cores are then subsampled for organic C content and dry bulk density (Section “Soil Analysis - Bulk Density, Loss-on-Ignition, Elemental Analysis”), and other targeted analyses (Kroetsch et al. 2011).

*^210^Pb and ^137^Cs:* For short-lived radioisotopes, samples are typically measured to a depth where ^210^Pb activity reaches supported background levels or until the full ^137^Cs peak has been captured (Lu and Matsumoto 2005; MacKenzie et al. 2011). For ^210^Pb and ^137^Cs, samples are typically measured from the upper 10 to 50 cm of wetland cores to represent a ~ 50 to 100-year time interval (Fig. 18a). Sampling for ^210^Pb and ^137^Cs analyses typically uses continuous increments of 1 to 2 cm for the upper part of the core; thicker sampling increments lead to greater uncertainty in age-depth models calculated from radioisotopes (Lu and Matsumoto 2005; MacKenzie et al. 2011). While the sediment mineral fraction contains the ^137^Cs and ^210^Pb, if gamma analysis is used, it is possible to measure bulk sediment without the need to separate the sample based on the type of material, making post-processing relatively easy. If using bulk sediment, ensuring a homogeneous medium is important and can be challenging. Some gamma detector geometries (i.e., shape) and efficiencies may preclude analysis of bulk sediment, for example if sample volume is too high or efficiency is too low for reliable detection, requiring large roots and other vegetation to be removed (e.g., from sieving). Removing plant material may reduce detector count time, but is itself an intensive processing step. For gamma counting, the sample should be referenced to a standard in the same geometry and sediment configuration.

The amount of sample material needed for laboratory analysis depends on the type of counter used. Gamma counters are non-destructive but typically require larger sample sizes (1–5 g) and longer analysis times (24–48 h), depending on sample size and level of activity. With larger samples (20–100 g) and with high-efficiency detectors, analysis times can be as short as 4 to 8 h. The sample preparation for gamma counters involves air, oven, or freeze drying, weighing, grinding, homogenizing, and sieving (Section “Soil Analysis - Bulk Density, Loss-on-Ignition, Elemental Analysis”). Coarse and fine fragments of the soil sample are weighed separately. Homogenized and sieved samples are then sealed in vials and placed inside the well of the gamma spectrometer. When performing gamma analysis on sediment samples, it is important to account for self-adsorption, where decay events are not measured by the detector because they attenuate in adjacent sediment particles. This is particularly important at the 46.5 keV peak, where total ^210^Pb is measured. Self-attenuation is highly dependent on sample composition and geometry, but generally increases with mass (e.g., Cutshall et al. 1983). Alpha counting is a destructive technique, but the lower detection limits of the counters require smaller sample sizes (0.2–0.5 g). Detection limits are dependent on individual methods and counting times, with typical limits for gamma counters of 0.1 to 0.5 disintegrations per minute per gram (dpm g^−1^) and ~ 0.001 dpm g^−1^ for alpha counters (Zaborska et al. 2007). The analyses provide data on activity of short-lived radioisotopes for each sample, and these data are used to calculate an age model (Section “Radiometric and Stratigraphic Dating – Age-depth Model Construction”).

If non-destructive gamma counting is used, the sample is placed in a container and inserted into a gamma counter, where gamma emission spectroscopy is used to measure ^210^Pb, ^226^Ra, and ^137^Cs, typically at 46.5, 352, and 663 keV energies respectively. If alpha counting is used, samples are first leached with hydrochloric and nitric acid before being plated and placed in an alpha counter and detected via 210-polonium (^210^Po). The leaching process requires 3 to 5 h of heating at 180 °C followed by overnight incubation at 80 °C. The total preparation time can range up to 24 h. One advantage of using alpha over gamma spectroscopy is that multiple soil samples can be analyzed at the same time and is therefore timesaving compared to gamma analysis. Alpha analysis provides data on ^210^Pb by measuring ^210^Po, as ^210^Pb and ^210^Po are believed to be in secular equilibrium (i.e., production of radioisotope is equal to its decay rate). The change between the peak of the ^210^Po and the known peak of the isotope tracer ^209^Po will determine the ^210^Pb activity of a sediment sample. Most of the sample preparation for alpha analysis should be done inside a fume hood and follow safety protocols (e.g., awareness of the spill kit locations, wearing laboratory coats, long pants, full shoes, and safety glasses) because it involves working with acid. More detailed information on dating methods can be found in Sanchez-Cabeza et al. (2000), Turetsky et al. (2004), Walker (2005), Suckow (2009), MacKenzie et al. (2011), Baskaran (2012), and Sanchez-Cabeza and Ruiz-Fernández (2012).

*Radiocarbon:* Radiocarbon does not require establishing a depth-activity curve like ^210^Pb and ^137^Cs, and therefore does not require continuous vertical sampling. Because of the long half-life of ^14^C, there are effectively no depth limitations in wetland sediment cores, aside from the potential influence of ‘bomb’ C and ^14^CO_2_ in uppermost samples. Unlike ^137^Cs and ^210^Pb, the material used for radiocarbon dating may influence the resulting ages (see below). Individual samples are submitted for analysis based on their relative distribution in the core; these often focus on lithology (texture, color, composition), physical properties, or other proxy transitions that may represent key changes to the environment that could affect the way CARs are interpreted.

Sample selection for radiocarbon dating requires an understanding of the C sources of the picked material or substrate, and how that material ended up in a core. Terrestrial or wetland emergent plant macrofossils, such as seeds and leaves, are excellent candidates for radiocarbon dating because the source of C in the plants is CO_2_ from the atmosphere and therefore will produce dates corresponding to the time when the plant was living. Submerged plants, on the other hand, can take up dissolved inorganic C from the water column in which they live, whose original C source may be highly depleted in ^14^C due to biological processes (e.g., respiration), geological context (e.g., carbonate dissolution), or from physical mixing (e.g., with ocean waters), resulting in an older apparent age than when those plants were growing (referred to as the ‘reservoir effect’) (Colmer et al. 2011; Jull and Burr 2015). For the same reason, radiocarbon of animals that consume aquatic plants or insects (i.e., ducks) can result in the incorporation of reservoir C, thereby compromising the radiocarbon age. Similarly, calcareous microfossils such as mollusks may incorporate old C from underlying carbonate strata and be subject to reservoir effects (Pigati et al. 2010; Alves et al. 2018). Plant roots are also generally avoided because roots often penetrate deeper strata than where C fixation is taking place, meaning they will likely produce a younger age than the layer of sediment or peat in which they were found. Radiocarbon dates also may be obtained on pollen isolated from sediments of freshwater wetlands (Brown et al. 1989; Mensing and Southon 1999).

The use of bulk sediment samples is generally not recommended for radiocarbon analysis because it may be affected by the inclusion of either younger or older C than the age of deposition of the stratigraphic horizon. Moreover, bulk samples may have radiocarbon-dead material with C older than the radiocarbon detection limit (Walker et al. 2007). However, in many wetlands, macrofossils of upland species are often not available for radiocarbon dating, in which case bulk sediment, pollen, or shells may be used; it is possible to determine the reservoir age and/or apply a carbonate correction, but results should be interpreted with caution (Pigati et al. 2010). If bulk sediment is used for radiocarbon dating (1–10 g needed), the soil samples typically are collected from the basal (bottom) increment and at regularly spaced intervals throughout the core.

Plant macrofossils are typically isolated by first sieving the sediment through a 250 μm sieve to remove the fine particles. The remaining coarse fraction is examined under a dissecting microscope, and identifiable seeds and leaves are separated from the rest of the sample using forceps. Macrofossils are identified using modern reference collections that include physical reference material, online databases, and botanical guides (e.g., Montgomery 1977; Lévesque et al. 1988). Samples typically are rinsed with deionized water and dried before submission to a radiocarbon laboratory.

Preparation of pollen samples for radiocarbon dating differs from the methods outlined below for using pollen as a stratigraphic marker (see Brown et al. 1989). Sample processing involves demineralization using hydrochloric and hydrofluoric acids, removal of humic acids using potassium hydroxide, and sieving to isolate the target size fraction (usually 10–150 μm). Several techniques have been developed recently to isolate pollen from other organic residue and target specific size classes (such as pine and other conifers), including flow cytometry (Zimmerman et al. 2019) and an on-chip sorter (Kasai et al. 2021).

After samples are collected for radiocarbon dating, material is sent to a laboratory specializing in radiocarbon dating. Given the potential for uncertainty around age estimates, multiple replicates are recommended. Pretreatment protocols vary with the type of material being dated and any radiocarbon laboratory-specific requirements.

*Stratigraphic markers:* For stratigraphic markers such as pollen, pollution or other geochemical excursions, and tephra, a general core description, as described above, is needed. Also, for pollen or pollution, historical information that can tie land-use change to a specific historical period is required. For tephra, geochemical techniques are used to link specific tephras to a given eruption, but the age still often needs to be determined by other radiometric or stratigraphic techniques.

*Pollen:* Pollen sampling for biostratigraphy in wetland cores typically starts with coarse sampling from a core (e.g., every 5–10 cm) to identify the approximate depth of a known horizon or other stratigraphic marker, followed by finer resolution increments to refine the results (Fig. 19b). Pollen processing techniques concentrate palynomorphs (organic-walled microfossils) from sediments through a series of chemical and physical steps that aim to eliminate or minimize non-pollen elements. Although the specific techniques employed vary with the type of sediment being processed, they typically involve demineralization through acid digestion with hydrofluoric acid, followed by removal of cellulose by acetolysis with sulfuric acid and acetic anhydride, oxidation with nitric acid, and removal of humic acids using potassium hydroxide (Doher 1980; Traverse 2007). Pollen residues are mounted on microscope slides in mounting media such as glycerin jelly or silicon oil, and assemblages are identified and quantified using a compound microscope with 400X to 1000X magnification.

*Pollution:* The concentrations or isotopic ratios of trace metals originating from human activities in wetland sediment cores can be tied to industrial practices and used to inform the chronology of sediment deposition. The local context is ideally considered prior to analyses to assess sources and pathways of trace metals into a given wetland and the extent to which metal concentrations in the paleo-record may be affected by proximity to a point source, long-range transport, and watershed-specific processes (Dunnington et al. 2020). Using this information, select trace metals or a collection of trace metals are measured and used to deduce peak output and deposition in relation to the rise and decline of an industrial practice such as coal combustion, wastewater discharge, or the use of leaded gasoline in motorized vehicles (Givelet et al. 2003; Cheyne et al. 2018; Peteet et al. 2018, 2020). Metals commonly used include As, Cd, Cu, Fe, mercury [Hg], Mn, Pb, titanium [Ti], and Zn, and source attribution often requires measurement of isotopic composition in addition to total concentrations (Cheyne et al. 2018). Measurements of the concentrations of other elements may be plotted against trace metal concentrations to normalize for non-anthropogenic-related variation that could affect metal concentrations and trends within the core (e.g., Ti as a proxy for anthropogenic dust and terrestrial sediment inputs; Fe and Mn as proxies for redox conditions; Givelet et al. 2003; Peteet et al. 2018). Common instruments used to measure metals in sediment samples include atomic absorption spectrometry, inductively coupled plasma mass spectrometry (ICP-MS), and x-ray diffraction (XRD) (Section “Soil Analysis - Bulk Density, Loss-on-Ignition, Elemental Analysis”).

*Tephra:* If present, tephras are noted during core description and sampled for tephrachronology. Tephras can also be located by noting brief excursions (i.e., deviations from expectation) in LOI or magnetic susceptibility, though not all tephras are magnetic. A smear slide can also help determine if an excursion is a tephra by examining the grains under a cross-polarizing microscope and noting the presence of glass shards. If a tephrachronology laboratory identifies the tephra from a specific eruption, this date is assigned based on historical records of the eruption.

*Key Covariates and Ancillary Measurements:* Many of the key covariates that are needed for radiometric or stratigraphic dating methods are the same as those required for soil C, specifically dry bulk density and C content, while other variables are important for interpretations (e.g., pH, and redox potential) (Section “Soil Analysis - Bulk Density, Loss-on-Ignition, Elemental Analysis”).

*Delta 13C (δ^13^C):* Measurements of δ^13^C are usually obtained at the same time that a sample is analyzed for radiocarbon when using AMS, which helps improve the accuracy of the radiocarbon age. The δ^13^C of a sample is used to correct for errors introduced from metabolic and respiratory pathway differences between the modern standard material and the sample material. If a sample is too small to obtain both δ^13^C and ^14^C, a δ^13^C of − 25‰ is assumed (Aitken 1990). The δ^13^C is measured by the radiocarbon laboratory, which makes the correction to a radiocarbon date before returning the results. A range of stable isotopes, such as ^15^N, can be measured in sediments and peat and can provide additional insights into changes in nutrient cycling and hydrological regimes that can affect C cycling with time (Section “Soil Analysis - Bulk Density, Loss-on-Ignition, Elemental Analysis”).

*Other biotic assemblages:* In addition to finding biostratigraphic horizons for pollen, it is also possible to identify changes in other biotic communities over time, such as plant macrofossils, invertebrates, diatoms, and dinoflagellate cysts and foraminifera (Pendea and Chmura 2012; Hu et al. 2023), which can help interpret changes in C accumulation rates. Assessing stable isotopes of various biotic assemblages can provide additional information. For example, an analysis of invertebrate stable isotopes (δ^2^H in chironomid head capsules, and δ^13^C in *Daphnia* ephippia) from sediment cores in the Prairie Pothole Region of North America indicated increased use of methanotrophic bacteria as a food source during a wet period, which was likely related to a concurrent increase in CH_4_ production in the prairie wetlands (Hu et al. 2023). Specialized training in identifying and interpreting changes in biotic assemblages throughout a core is required.

#### Radiometric and Stratigraphic Dating – Age-Depth Model Construction

*What:* Age-depth modeling allows calculation of vertical SAR, which is then used to calculate long-term CAR, as well as changes in rates that may have occurred through a wetland’s history due to environmental or anthropogenic perturbations to the system (e.g., sea-level rise) (Reimer and Reimer 2007; Bronk Ramsey 2009; Jull and Burr 2015; Reimer et al. 2020). Age-depth modeling of wetland soils first entails building separate age-depth models for each dating technique (^137^Cs, ^210^Pb, ^14^C, stratigraphy), and then combine the information for an overall, whole-core age-depth model that incorporates information from each method-specific model (Figs. 18, 19) (Mauquoy et al. 2004; Reimer and Reimer 2007; Blaauw and Christen 2011; Jull and Burr 2015; Reimer et al. 2020; Stuiver et al. 2021).

*Where* and *When:* After ages are determined using radiometric dating technique, age-depth models are calculated using statistical programs on standard computers.

*Who:* Age-depth modeling commonly requires knowledge of one or more programing languages (e.g., R, Python). While there are numerous programs or packages to assist investigators with constructing age-depth models, training and experience is still required to evaluate potential biases from various dating and modeling techniques.

How:

*^137^Cesium age-depth models:* Age-depth models using ^137^Cs require knowledge of the depth of the ^137^Cs onset and peaks. In some cases, the 1963 peak (with higher activity) may be easier to identify than the first occurrence of ^137^Cs in 1954 (with lower activity). Due to its chemical properties, ^137^Cs (and ^210^Pb) can mobilize in wetland sediments and can move up or down the sediment profile away from the original onset and peak locations, which should be considered before incorporating these data into age models (Drexler et al. 2018). In assessing the degree of this mixing on the use of 1954 onset or 1963 peak, the ‘sharpness’ of the increase in values can indicate the degree of downward mixing, as can ancillary data such as color or particle size density. A distinct 1963 peak verified by errors bars can provide some certainty that the peak is accurate (e.g., Thorne et al. 2018). More data points (i.e., finer sample increments and more of them) will provide greater certainty in the construction of ^137^Cs soil profiles. Ideally 20 (or at least 10) dates are used from each of three replicate cores.

*^210^Pb age-depth models:* ^210^Pb based age models typically rely on one of two approaches to assign dates to individual intervals: 1) accretion rates are derived from changes in unsupported ^210^Pb activity as a function of depth or accumulated mass (i.e., Constant Initial Concentration [CIC] or Constant Flux Constant Sedimentation rate [CF:CS] models, respectively); or 2) accretion rates are derived from the entire ^210^Pb inventory (Constant Rate of Supply [CRS] and Plum models) (Goldberg 1963; Aquino-López et al. 2018; Arias-Ortiz et al. 2018; Iurian et al. 2021).

The CIC model assumes constant initial ^210^Pb concentrations in the top layer of sediment, leaving radioactive decay as the only process controlling the down-core activity of ^210^Pb, while the similar CF:CS model assumes constant sedimentation and ^210^Pb delivery to the surface layer. In practice, the CIC and CF:CS models derive accretion rates from the slope of log normalized activity versus depth (vertical accretion in mm yr^−1^) or accumulated mass (mass accumulation rates [MAR]) in g m^−2^ yr^−1^) (Turetsky et al. 2004; Walker 2005; Suckow 2009). These models yield one accretion rate per interval where a slope is fitted. If multiple intervals are fitted throughout a core, multiple sedimentation rates may be calculated, however temporal resolution is low since each interval requires multiple points to fit a slope and assumes constant accretion over each interval (Appleby and Oldfield 1992). Sediment interval ages are calculated from the sample depth and accretion rate. The CIC and CF:CS models are well suited for ^210^Pb profiles that display exponential decay curves, but may not appropriate in locations where sedimentation may change through time as may occur, for example, due to ecosystem response to environmental perturbations that are not constant in time (e.g., alternating periods of drought and deluge).

The CRS model is a variant on the advection-decay equation (Goldberg 1963; Appleby and Oldfield 1978; Sanchez-Cabeza et al. 2000; Sanchez-Cabeza and Ruiz-Fernández 2012) and also assumes that ^210^Pb supply to the sediment surface is constant through time, but allows for changing sedimentation rates, in addition to decay rates, to control the down-core activity of ^210^Pb. With the analytical implementation of the CRS model, it is crucial to measure the entire ^210^Pb inventory to achieve accurate age models; missing sections of the core or not counting to the point where excess ^210^Pb is below detection limits, can result in poor age models with high uncertainty. There is an implementation of the CRS model (called Plum) set within a Bayesian statistical framework that can determine dates and accretion rates in cores with less sampling resolution (Aquino-López et al. 2018). Plum is available as a package *rplum* in the programming language R (Blaauw et al. 2022a). The CRS and Plum models are appropriate for cores where ^210^Pb profiles do not display simple exponential decay curves because sedimentation rates may have changed. Unlike the CRS model, the Plum approach can model gaps in the ^210^Pb profile and associated uncertainty. The certainty of ^210^Pb age-depth models is increased in the presence of multiple data points of CAR over a longer period of time; therefore, a ^210^Pb age-depth model for a natural wetland with C accumulation of 100 + years will be more robust than that of a ^210^Pb age-depth model for a recently restored wetland (Creed et al. 2022).

*Radiocarbon age-depth models:* Radiocarbon dates of samples are provided to investigators as ‘radiocarbon years before present’, where present day is considered 1950 AD. The results will also include a measurement error on the radiocarbon age. Radiocarbon years are then calibrated to calendar (also referred to as ‘secular’) years to account for variability in the past in the atmospheric concentration of ^14^C (Reimer et al. 2020; Stuiver et al. 2021) prior to producing an age-depth model. Because ^14^C/^12^C ratios are influenced by environmental and extraterrestrial factors, calibration curves, based on well-dated sequences such as tree rings that relate calendar to radiocarbon years are used to convert measured radiocarbon dates to calendar years (Suess 1970; Heaton et al. 2020; Reimer et al. 2020). These calibrations differ for northern versus southern hemispheres and marine versus terrestrial settings, so care must be used in selecting the appropriate calibration curve and researchers should use up-to-date calibration software. Likewise, some depositional settings require application of a reservoir correction to account for age biases caused by underlying carbonates and marine systems. Several programs are used to calibrate individual radiocarbon dates, including *Calib* (Stuiver et al. 2021) and *OxCal* (Bronk Ramsey 2009), and some calibration programs allow for an input of a mixture of radiocarbon and calendar ages (Blaauw and Christen 2011; Aquino-López et al. 2020). Radiocarbon age-depth models are generally not extrapolated outside of the upper and lower radiocarbon dates unless other types of age constraints are available.

*Stratigraphic age models:* Stratigraphically based dates require knowledge of the timing of the various events. For example, the timing of introduction of non-native species or land management changes in specific areas will constrain interpretation of pollen- or pollutant-based chronologies. Use of tephra dates requires calibration of tephras to specific dated eruptions. Typically, stratigraphically based dates do not form the sole basis of an age model but are integrated with other evidence to provide a whole-core age-depth model.

*Whole-core age-depth model:* Whole-core age-depth models integrate complementary data from both discrete dates (i.e., from ^137^Cs, radiocarbon dates, or stratigraphic markers) and continuous data (i.e., from ^210^Pb) to produce a whole-core age-depth model (Fig. 19b). Classical age-depth modeling involves specifying either a linear or other (e.g., smoothed splines) function between the dated tie points (Blaauw 2010). Bayesian age-depth modeling (e.g., Bacon model, Blaauw and Christen 2005, 2011) has now superseded the classical, frequentist methods in terms of preferred approach, and can be implemented using software such as the *rbacon* or *oxcAAR* packages for R (Hinz et al. 2021; Blaauw et al. 2022b). In this approach, prior information including an assumed accumulation rate is specified to constrain the probability distributions for calibrated ages and the interpolated ages between the radiocarbon tie points. Note that Plum builds on the Bacon model framework to incorporate ^210^Pb data, ^137^Cs, and ^14^C ages, as well as calendar ages from stratigraphic markers, to create a single age model from a range of sources (Aquino-López et al. 2018).

*Appropriateness of data for age-depth reconstructions:* In some cases, the above analyses yield data that are inconsistent or hard to interpret, and thus do not meet the age-depth model assumptions. For examples, a missing ^137^Cs peak, a ^210^Pb profile that is either unchanging or increases with depth (rather than the anticipated exponential decay), or old ^14^C ages stratigraphically above younger ages, can indicate a mixed sediment column or disruption in soil accumulation. Such inconsistent data can result from a core that is not suitable for constructing a robust age model or reconstructing past rates of sediment and C accumulation. Age reversals in radiocarbon dates can occur (Fig. 19), but Bayesian age-depth modeling that incorporates all dates in a profile, can produce an age model that includes the 95% confidence intervals for an age model, based on the probabilities of radiocarbon to calendar calibration, as well as the information from the dates above and below each radiocarbon control point (Fig. 19b).

### Greenhouse Gas Fluxes

#### Definitions and Units

*Definitions:* Ecosystem greenhouse gas (GHG) flux generally refers to emissions to or uptake from the atmosphere, which are governed by GHG production, consumption, and transport processes. GHG fluxes through aquatic transport of dissolved gases are discussed in Section “Lateral Flux”. Often the most dominant ecosystem C flux is the exchange of CO_2_, which is primarily controlled by ecosystem scale gross primary productivity (GPP) and ecosystem respiration (ER, also often abbreviated by ‘RECO’). GPP represents total C fixation by plants through photosynthesis, with autotrophic respiration (R_A_) of plants returning roughly half of this fixed CO_2_ back to the atmosphere (Chapin et al. 2002). Dead plant material is also largely decomposed to CO_2_ through a cascade of organisms, from macrofauna to microbes, and processes, from physical breakage to fermentation, which collectively support heterotrophic respiration (R_H_) (Bridgham 2014). Collectively, R_A_ and R_H_ are the two components of ER which represent atmospheric CO_2_ emissions. The difference between GPP and ER is considered net ecosystem productivity (NEP, a unit of productivity) or net ecosystem exchange (NEE, a unit of flux [NEE = − NEP]); Section “Overview of Wetland Carbon Pools and Fluxes” details wetland C balance. Because ecosystem GHG fluxes are operationally defined as atmospheric exchanges, processes occurring within soils or within the water column, such as CH_4_ oxidation to CO_2_ (Raghoebarsing et al. 2005), are not captured by atmospheric measures of CO_2_ and CH_4_ fluxes. Similarly, lateral C flux (e.g., tidal transport) can complicate interpretation of GPP and ER in some wetlands (Yan et al. 2008). N_2_O flux, despite not being a C-based gas, is often converted to CO_2_-equivalents (CO_2-eq_) and included as part of wetland GHG studies due to its high warming potential compared to CO_2_ and CH_4_ (Neubauer and Megonigal 2015; Forster et al. 2021).

ER is relatively slow in the O_2_ limited conditions of wetland soils (Reddy and DeLaune 2008), which results in an imbalance between GPP and ER and can lead to long-term C accumulation in wetland soils. Low O_2_ conditions also result in the production and emission of CH_4_, which may not always contribute much to wetland C budgets, but is important for wetland radiative balance (Section “Overview of Wetland Carbon Pools and Fluxes”). A potentially large portion of CH_4_ does not reach the atmosphere, and instead is oxidized to CO_2_ by methanotrophic bacteria aerobically or anaerobically (Bridgham et al. 2013). Parts of wetlands that experience wetting and drying tend to favor N_2_O production (Reddy and DeLaune 2008; Tangen and Bansal 2022) because both oxic and anoxic metabolic steps are required for ammonification, nitrification, and denitrification. There is considerable uncertainty regarding the role of wetlands on global N_2_O budgets (Tian et al. 2020).

Transport of GHGs to and from the atmosphere can occur through combinations of advection, diffusion, and ebullition (gas bubbles out of the sediments). If plants are present, then GHG exchange occurs between plants and the atmosphere through photosynthesis and respiration, but also through plant-mediated transport of GHG (e.g., CH_4_) through their stems or stomata (Joabsson et al. 1999; Laanbroek 2010; Milberg et al. 2017; Bansal et al. 2020).

GHG fluxes from wetlands are spatially heterogeneous across multiple scales ranging from meters to landscapes. Within-wetland spatial heterogeneity has been associated with factors such as vegetation, soils, water depth, and microtopography (Ueyama et al. 2023). GHG flux can vary over hourly, daily, multiday, seasonal, and interannual time scales (Koebsch et al. 2015; Bansal et al. 2018; Knox et al. 2021). Temporal variability has been associated with factors such as solar radiation, temperature, precipitation, atmospheric pressure, wind speed, plant activity, and water-level changes (Knox et al. 2021).

*Units:* Fluxes are typically expressed as a quantity per area per time (e.g., μmol CO_2_ m^−2^ s^−1^, g CO_2_ m^−2^ d^−1^). While the units for the area and time terms will logically vary with the spatial and temporal scales of individual studies, the units for the quantity term often depend on the focus of the study. For example, CH_4_ fluxes might be reported using units of grams C (g CH_4_-C) as part of an ecosystem C budget; or as grams CH_4_ if the study is focused on exchanges of CH_4_ with the atmosphere; or as grams CO_2-eq_ to address questions related to climate change. Studies focused on instantaneous fluxes often use molar units in seconds (e.g., μmol CO_2_ s^−1^ or nmol CH_4_ s^−1^). The choice of units is somewhat arbitrary since it is mathematically straightforward to convert between units to reach an intercomparable metric such as g C m^−2^ day^−1^. Importantly, any normalization of fluxes of mass to CO_2-eq_ units requires selecting among multiple different GHG metrics (e.g., GWP, SGWP) of radiative balance and choosing a time scale (e.g., 20-year, 100-year) that is appropriate for the questions being asked (Section “Overview of Wetland Carbon Pools and Fluxes”).

It is worth noting that different studies can assign positive or negative signs to the same flux, depending on the frame of reference. Thus, a process such as CO_2_ fixation via photosynthesis can be considered a positive or negative flux, depending on whether the flux is expressed relative to the wetland (positive, since photosynthesis acts as a C source to the wetland) or the atmosphere (negative, because photosynthesis removes CO_2_ from the atmosphere). One way around this potential source of confusion is to report all fluxes as positive numbers and then use descriptive words such as ‘efflux out of the soil’ or ‘emission to the atmosphere’ so readers unambiguously know the direction of gas transport. In analyses that need both positive and negative fluxes (e.g., C budgets), studies should clearly define the associated processes and direction of flux (i.e., into or out of the wetland or atmosphere).

**Rationale:** GHG emissions and uptake determine wetland radiative balance and short- and long-term effects on climate (Section “Overview of Wetland Carbon Pools and Fluxes”), which determine their usefulness as nature-based climate solutions. The processes that control GHG fluxes are highly dependent on environmental conditions, and therefore are sensitive to weather and climate, hydrology, and vegetation phenology and community composition, as well as from management actions such as wetland drainage and restoration. Multiple studies have demonstrated increases in wetland CH_4_ emissions from future warming (Bansal et al. 2023; Bao et al. 2023; Zhang et al. 2023), which will need to be considered in climate mitigation planning. Even so, the large stores of C in wetland soils represent a lifetime radiative cooling effect from wetlands, which could be negated through wetland loss, primarily for agriculture (Fluet-Chouinard et al. 2023).

#### Chamber Measurements

**Fig. 20:**
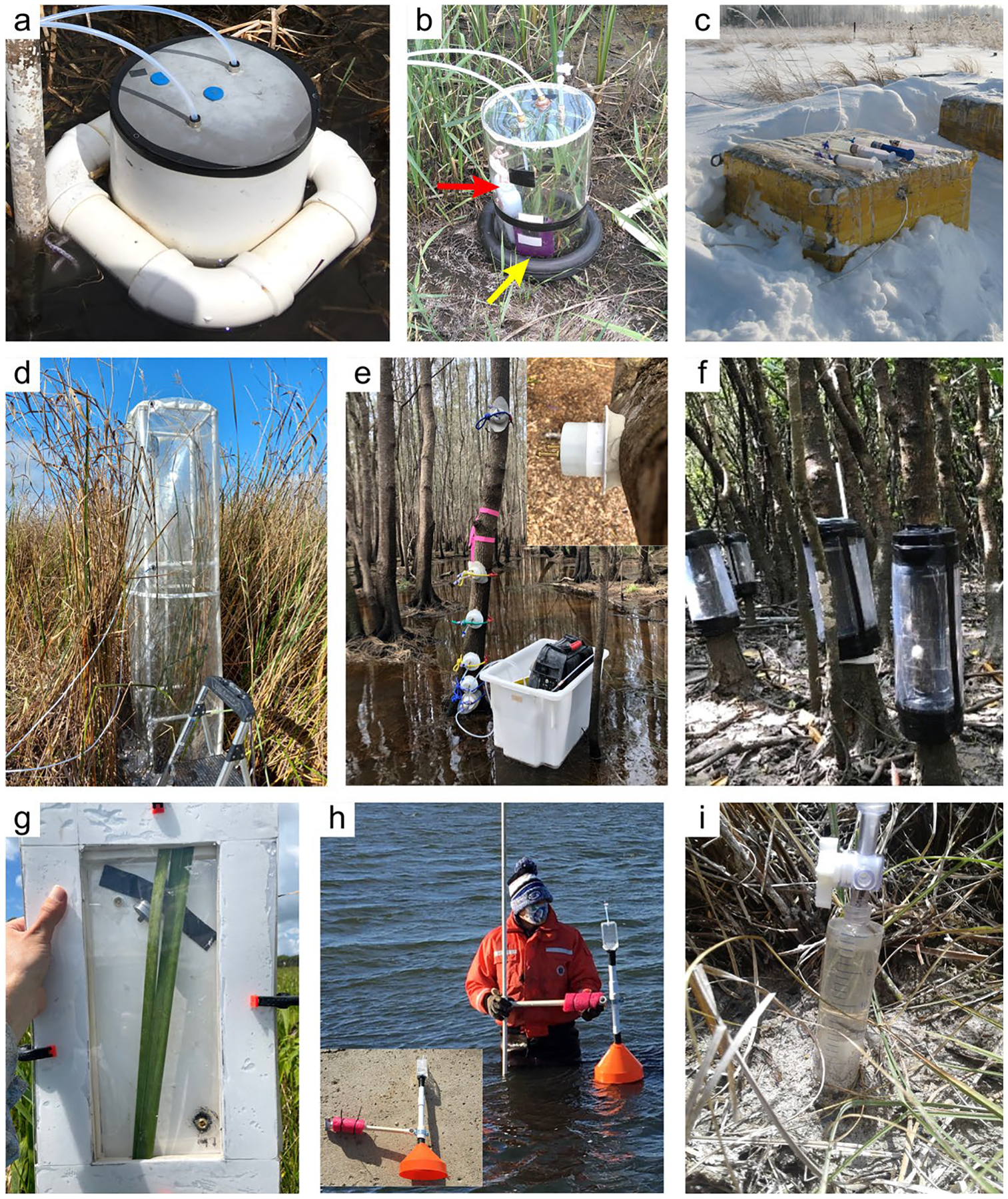
(**a**) A floating gas flux chamber made of polyvinyl chloride (PVC) connected with inlet and outlet tubes to a high frequency gas analyzer; (**b**) clear, static chamber over vegetation at the edge of an experimental wetland (at Northern Prairie Wildlife Research Center, North Dakota, USA): red arrow pointing to ice pack to keep chamber cool and yellow arrow pointing to fan to mix air; (**c**) non-growing season chamber measurement in permafrost regions of China; (**d**) whole-plant chamber over *Phragmites* for emergent macrophyte and soil fluxes; (**e**) gas flux measurements of tree stems using the Small Nimble In Situ Fine-Scale Flux (SNIFF) method with cavity ring-down spectroscopy (Picarro, GasScouter) gas analyzer from six stem heights within subtropical *Casuarina* sp. lowland forest; (**f**) measurements of methane transport and carbon dioxide respiration from the stems of mangrove *Kandelia*; (**g**) leaf chamber equipped with a digital thermometer over *Typha*; (**h**) deploying inverted cone ebullition trap (2.5-cm diameter PVC) with plastic funnel (20-cm diameter) attached to air-tight collection bottle on top with valve; (**i**) submerged peatland ebullition trap using a syringe at Fletcher Creek Ecological Preserve, Ontario, Canada. Images with permission from Olivia Johnson (a, b, h), Xiaoxin Sun (c), Scott Jones (d), Luke Jeffery (e), Jia-fang Huang (f), and Maria Strack (i)

*What:* Measurement of GHG flux using static chambers (also referred to as ‘non-steady-state chambers’ or ‘enclosures’) is the most commonly employed approach in GHG flux studies (Fig. 20) (Hutchinson and Livingston 1993; Livingston and Hutchinson 1995; Healy et al. 1996; Levy et al. 2011; Pihlatie et al. 2013; Collier et al. 2014). By calculating the change in gas concentration within chambers over minutes to hours, these measurements quantify the net GHG vertical flux out from or into the underlying soil or water column enclosed by the chamber. Gas concentrations can be discretely collected for laboratory analysis or measured continuously using a high-frequency gas analyzer. The manual static chamber approach (as opposed to automated chambers) is relatively inexpensive, can be used at multiple nearby locations simultaneously, allows for experimental manipulations, and does not require a power supply, making it logistically feasible compared to other approaches.

The rate of GHG flux will depend on a suite of biophysical mechanisms. The small footprint of chambers (cm^2^ to m^2^) and short deployment times provides an opportunity to directly link fluxes to specific environmental conditions and gas transport pathways. However, due to high variability in GHG fluxes in both space and time, and the localized spatial and temporal scope of inference from chamber measurements, extrapolating to larger areas and longer timeframes should be considered with caution (Vargas et al. 2011). Chamber-based flux measurements are often temporally interpolated to calculate cumulative flux (i.e., the total amount of GHG flux over time), which generates additional uncertainty due to missing information between consecutive flux measurements. High spatial and temporal replication can help characterize functional relations between fluxes and mechanistic covariates such as soil moisture, redox potential, vegetation, and temperature (Webster et al. 2008a, b; Bridgham et al. 2013; Bridgham and Ye 2013; Bansal et al. 2016, 2023; Yue et al. 2022).

*Where:* Static chamber-based GHG fluxes are measured at the soil or water surface. Some study designs involve clipping or cutting vegetation within the chambers, while others leave vegetation intact or place chambers adjacent to plants. Differences among these methods capture different components of GHG flux (R_H_, R_A_, ER, NEE; see *Partitioning flux into GPP, R_A_, R_H_*). Paired chambers with and without plants can provide information on plant-mediated CH_4_ flux (Bansal et al. 2020; Hill and Vargas 2022).

Placing chambers along transects across vegetation zones and hydrological conditions is a common practice (e.g., Miller 2011; Creed et al. 2013; Finocchiaro et al. 2014; Comer-Warner et al. 2022). Many studies have demonstrated differences in flux among catchment landscape positions and vegetation zones (Phillips and Beeri 2008; Dunmola et al. 2010; Badiou et al. 2011; Creed et al. 2013; Desrosiers et al. 2022), as well as between wet, dry, and transitory hydrological states (Sun et al. 2012; Tangen and Bansal 2019). In addition to these factors, landscape-level GHG flux heterogeneity has been associated with wetland type, climate, land use, and geochemistry (Turetsky et al. 2014; Morse et al. 2015). Therefore, placement of chambers will depend on the specific research question, study system scale, and geographic extent.

*When:* GHG fluxes change at diurnal, synoptic (multi-day), seasonal, and annual time scales (Knox et al. 2021). Mid-day is the most common time of day for manual sampling due to logistical challenges of measuring fluxes at night. However, mid-day sampling can bias estimates of average flux rates due to diurnal variation in temperature, solar radiation, soil and litter moisture, and microbial and plant activity (Kuehn and Suberkropp 1998; Kuehn et al. 2004; Cueva et al. 2017; Yang et al. 2017; Bansal et al. 2018; Sieczko et al. 2020). Nighttime sampling, or 24-h sampling campaigns, help to understand if mid-day samples over- or under-estimate average daily flux rates; a correction factor can be applied to limit potential bias (Cueva et al. 2017).

Seasonal variation is one of the largest sources of temporal variation (Knox et al. 2021), with flux rates generally increasing during the warmest months of the growing season. Non-growing season fluxes during ice-cover periods are also important because studies have shown CO_2_ and CH_4_ fluxes in winter months can account for 10 to 20% of annual fluxes (Sun et al. 2012; Delwiche et al. 2021), although the winter contribution can also be minimal in some wetland systems (Hanson et al. 2016). Flux rates also can differ, both in magnitude and direction (emission versus uptake), between inundated, dry, and transitional states (Kim et al. 2012; Tangen and Bansal 2019) or tidal cycles (Capooci and Vargas 2022a). Thus, sampling events distributed periodically (from 3 times per week to once a month, depending on logistics) and during transient events throughout the growing season improve characterization of GHG fluxes. GHG fluxes often occur in pulses within hours following rain events (Enanga et al. 2016) and are influenced by tidal cycles and micrometeorological conditions (Capooci and Vargas 2022b). Thus, episodic sampling is advised to capture pulse events. Co-located eddy covariance towers (Section “Eddy Covariance”) can guide sampling designs and times, especially in cases where less-observable gradients of hydrologic GHG fluxes affect variability in time (e.g., Knox et al. 2019) and space (e.g., Windham-Myers et al. 2018). Optimization statistical sampling protocols (e.g., temporal univariate Latin Hypercube sampling) can inform how to best measure gases of interest (Vargas and Le 2023).

*Who:* Collection of gas samples and measurement of covariates (e.g., temperature, barometric pressure, humidity, etc.) are relatively straightforward, but still require a moderate level of training or technical expertise. Laboratory analyses of the gas samples require more advanced technical capability and training using specialized instruments such as gas chromatographs (see below). Proficiency with programming may also be needed for calculating flux rates from the static chamber samples.

*How:* The chamber approach involves deploying static chambers at the soil or water surface, often using chamber bases (also called ‘collars’) to seal the chamber to the soil, or using floatation devices to float the chamber on the water surface (Fig. 20). Bases are used in soils or when water levels are relatively shallow. Bases are typically inserted 2 to 10 cm below the surface. It is best to wait at least a day after inserting bases prior to chamber measurement to minimize effects of soil disturbance on GHG flux (Tangen et al. 2015). Bases can also create microhabitats that differ from the surrounding area (e.g., standing water or altered vegetation inside bases) and it is recommended to move them occasionally. A small hole can be drilled in the side of the collar to allow water to drain and can be plugged prior to GHG flux measurements.

Static chambers have been constructed with variety of materials and a range of sizes and shapes. Chamber material can be transparent or opaque. Internal temperatures of transparent chambers can increase substantially over the sampling interval, which can affect biochemical and physical transport processes, and also affect flux calculations. Footprint sizes can also vary greatly, with enclosures ranging from small chambers (e.g., ~ 0.03 m^2^; Finocchiaro et al. 2014) to mesocosm-scale (e.g., 8.5 m^2^; Bridgham et al. 1995) and large whole-ecosystem enclosures (e.g., 115 m^2^; Hanson et al. 2017). Other considerations include: adding fans to circulate air within larger chambers to ensure gases are well mixed; using small-diameter vents to maintain pressure equilibrium between the chamber and the ambient air outside, particularly under windy conditions (see Parkin and Venterea 2010 for details on vent size and shapes; Zhu et al. 2014; Zhu et al. 2015); and creating a seal between the chamber and base to avoid gas leakage. Seals can be created using rubber or compressed foam seal strips (Rolston 1986) or placing the chamber into a groove or trough with standing water around the base (Winton and Richardson 2016).

Once a static chamber has been placed over a base or float and sealed, gases accumulate in the headspace and samples of headspace gas are collected repeatedly over a specified amount of time. Special care should be taken to avoid artificially inducing bubble release (ebullition) from soils when moving around the sample location and when deploying the chamber. Ideally, structures such as boardwalks are constructed to avoid disturbances from trampling in mucky conditions. Gas concentrations can be collected discretely or continuously from static GHG chambers. For manual collection, a headspace gas sample is collected by syringe either through a valve or by inserting a needle through a rubber septum in the chamber top. The gas sample is then injected into an exetainer or vial for transport to a laboratory (Magen et al. 2014) for analysis on a gas chromatograph. If a high-frequency gas analyzer is available, it can be attached directly to the chamber via inlet and outlet tubing to provide real time, continuous changing gas concentrations. Whether discrete or continuous sampling occurs, the length of chamber deployment depends on environmental conditions and flux rate stability. Deployments can vary from a few minutes to > 1 h, with interval sampling as needed to capture a change in gas concentration over time to calculate flux rates. It is ideal to aim for as short a deployment time as possible to reduce chamber artifacts (Healy et al. 1996) and increase replication.

Calculations of GHG flux rates typically require data describing chamber dimensions (headspace volume, surface area), atmospheric pressure and air temperature inside the chamber, and the length of time between sampling intervals. Measurements are corrected for each time point using the ideal gas law to calculate moles of gas:

(13) n=PV/RT

where P is partial pressure of the GHG (concentration reading from analyzer [ppm] × pressure [atm] in chamber); V is the volume of chamber headspace (L); R is the ideal gas constant (0.0821 L atm mol^−1^ K^−1^); and T is the temperature in Kelvin inside the chamber. Fluxes are calculated as change in concentration over time over area:

(14) $$\text{mol }{\text{time}}^{-1}{\text{area}}^{-1}=\text{n}/\left(\text{sampling interval}\times \text{surface area}\right)$$

Gas concentrations that increase linearly over time typically represent diffusive flux. Accumulation of gases inside the chamber during flux measurements can reduce the diffusion gradient enough to artificially slow flux rates (static chamber artifact), and therefore non-linear equations are used to back-calculate the actual flux (Hutchinson and Mosier 1981). Large jumps in concentrations over short timeframes likely indicate ebullition events. A major limitation of using static chambers with relatively few manual point measurements is that it is often difficult to discriminate diffusive flux, chamber artifacts, and ebullition processes from random error. High-frequency gas analyzers and specialized software packages (e.g., *HMR* R package, Pedersen 2022) can be used to partition artifacts and ebullitive fluxes (e.g., Capooci et al. 2019; Villa et al. 2021; Capooci and Vargas 2022b).

GHG flux data processing includes considering criteria for fluxes that do not follow linear/nonlinear trends. Typically, R^2^, p-values, or Root Mean Square Errors for the slope of the increase in gas concentration over time in the chamber are used to accept or reject flux measurements either of a single GHG or multiple GHGs (Petrakis et al. 2017; Rudberg et al. 2021). If gas concentrations or calculated flux rates are very low, assigning no values to the flux measurement (i.e., not applicable, N/A) instead of a value of zero (i.e., not statistically different from zero) would bias the flux estimates upwards.

*CH_4_ flux through plants and trees:* Vegetation in wetlands can affect CH_4_ emissions (Bodmer et al. 2021). To supply required O_2_ for root metabolism, wetland plant species use either unidirectional diffusion or pressurized-ventilation through aerenchyma tissues (Armstrong et al. 1992; Björn et al. 2022). Both O_2_ transport pathways allow for GHG transfer from the sediment to the atmosphere (often referred to as plant-mediated flux). There are a number of methods to quantify plant-mediated flux using chambers placed over plants or directly on leaves (Fig. 20b, d, g) (Yavitt and Knapp 1998; Bansal et al. 2020; Villa et al. 2020). If chambers are placed over plants, then short incubation times are used because increased humidity in the chamber can affect stomatal activity or inhibit the pressurized-ventilation system. Studies on plant-effects on CH_4_ flux should ideally concurrently measure porewater CH_4_ concentrations (Section “Dissolved Greenhouse Gases, Dissolved Inorganic Carbon”) and ebullition (below) to fully assess how plants influence CH_4_ emissions (Bansal et al. 2020).

Open flow-through chambers are designed similarly to closed static chambers, except with the addition of inflow and outflow ports with blowing air (i.e., with a fan). Gas samples are collected from inflow and outflow ports for flux calculations (see Stefanik and Mitsch 2014). Flow-through chambers have proven useful in some situations, such as over large plants (e.g., Stefanik and Mitsch 2014) because they can remain in place and used over longer timeframes. However, they require controlled and measured airflow for flux calculations and to constantly flush the chambers of accumulating gases (Valente et al. 1995).

Tree stems have also been identified as relevant sources of CO_2_ and CH_4_ fluxes (Pangala et al. 2017). Although CO_2_ emissions from tree stems (i.e., stem respiration) is a well-known process (Edwards and Hanson 1996), CH_4_ fluxes from tree stems represent a novel physiological mechanism (Covey and Megonigal 2019; Vargas and Barba 2019) that is relevant for local-to-global CH_4_ budgets (Barba et al. 2019a; Zhang et al. 2022). Most tree stem flux studies have been performed using manual chambers similar to those used for soils, but usually with a smaller diameter to fit a tree stem (Fig. 20e, f) (Warner et al. 2017; Jeffrey et al. 2020), or modified to cover a larger vertical area within a stem (e.g., rectangular form; Pitz et al. 2018), or using semi-rigid chambers that wrap around the tree stem (Siegenthaler et al. 2016). High-frequency gas analyzers coupled with automated chambers to measure stem CO_2_ and CH_4_ (Pitz and Megonigal 2017; Barba et al. 2019b) provide information of diel and synoptic patterns across the growing season. It is also important to measure CH_4_ fluxes in multiple locations on the tree stem because CH_4_ flux is generally higher from lower portions on the main trunk (Pangala et al. 2017).

*CH_4_ ebullition:* Recent research has demonstrated that ebullition can be a major pathway for CH_4_ to the atmosphere from the sediments in a variety of water body types, including lakes (Bastviken et al. 2004; DelSontro et al. 2018), reservoirs (Deemer et al. 2016), rivers (McGinnis et al. 2016), and wetlands (Stamp et al. 2013; Jeffrey et al. 2019; Stanley et al. 2019). While chambers combined with high resolution CH_4_ sensors can be used to distinguish ebullition from diffusive emissions (e.g., Goodrich et al. 2011), submerged bubble traps can directly quantify ebullitive emissions (Flury et al. 2010; Maeck et al. 2014; Linkhorst et al. 2020). An ebullition trap consists of a fully submerged inverted funnel with a detachable collection container at the apex and some anchoring feature, such as weights, ropes, buoys, or a frame (Fig. 20h, i). Traps can be customized for wetland conditions or logistical considerations (i.e., collapsibility for easier transport). Frequently, subsurface (buried) traps with syringe collectors are used to estimate ebullition in peatland environments (Fig. 20i) (Strack et al. 2005). The volume and CH_4_ concentration of captured gas (converted to moles using the ideal gas law) over the time of deployment relative to the surface area covered by the trap provides an area-based ebullitive CH_4_ flux rate. Automated bubble traps have also been built that use pressure sensors (Maeck et al. 2014) or optical detectors (Delwiche et al. 2015) to quantify and log GHG ebullitive flux. While manual traps are less expensive, automation saves labor time and provides higher temporally resolved data. All ebullition traps are limited in spatial resolution by the number of traps constructed and deployed, although methods of strategic statistical sampling can be employed to adequately resolve spatial heterogeneity within a system (Beaulieu et al. 2016).

Other approaches used to estimate CH_4_ ebullitive fluxes include hydroacoustics, robotically controlled devices, and process-based models. The hydroacoustics approach uses an echosounder to record the acoustic backscattering of bubbles in the water column, which can be scaled to bubble volume if the echosounder has been calibrated, and to CH_4_ ebullitive flux if the CH_4_ bubble concentration has been measured (Ostrovsky et al. 2008; DelSontro et al. 2015). This approach, however, is best used in waters deeper than 2 m as there is a near-field zone of inaccurate data associated with each echosounder depending on the shape and beam angles of the transducer (Simmonds and MacLennan 2006), and overlap with macrophytes in shallow waters tends to complicate distinguishing bubbles from plants. Another approach to estimate CH_4_ ebullition is using an optical CH_4_ detector placed just above the water surface and propelled by a robotically controlled watercraft (Grinham et al. 2011). If diffusive emissions are below the detection limit of the sensor, then this system would quantify only ebullitive emissions. Systems like these, however, are best employed in water bodies without disruptions by emergent macrophytes.

Process-based models combined with gas pressure thresholds are commonly used to estimate CH_4_ ebullition from wetlands. Water table elevation, barometric pressure and temperature, along with CH_4_ production and oxidation rates, are major factors influencing ebullitive CH_4_ flux in wetland environments (Fechner-Levy and Hemond 1996; Walter et al. 1996). The gas pressure threshold at which ebullition occurs has typically been calculated as when the total pressure exerted by four gases (CH_4_, CO_2_, N_2_, and O_2_) exceeds ambient pressure (Tang et al. 2010; Raivonen et al. 2017). Recent model comparisons have shown though that using bubble volumes instead of pressure as a threshold produces estimates more comparable to observations (Peltola et al. 2018). Models have the advantage of being able to use high resolution input data to output high resolution ebullitive results, but, like all models, they need to be paired with field data to validate their usefulness.

*Gas chromatographs:* Gas chromatography is a well-established and robust method for analyzing GHG concentrations over a wide concentration range from less than atmospheric concentrations up to several percent by volume (e.g., Sitaula et al. 1992; van der Laan et al. 2009). Gas chromatograph (GC) systems include an injector for sample introduction, one or more columns for separating analytes, and detector(s) for measuring analytes. The temperature of the columns is precisely ramped or kept constant with an oven. A carrier gas (e.g., He, hydrogen gas [H_2_], N_2_, Ar) is required to move the sample through the column(s). Design, setup, and method development (including appropriate calibration and quality control schemes) is time-consuming. It is important to work with the supplier and a specialized technician to make sure the equipment is set up correctly and meets the needs of the investigation.

Gas samples can be injected into a GC manually or with an autosampler. Manual injections are time intensive, require user training, and are subject to user inconsistency. Injection precision can be improved markedly by including a sample loop in the injection system and/or use of an autosampler. Though the up-front cost is high, autosamplers can save considerable technician time over the lifetime of the GC.

Column selection and configuration depends on the analytes of interest, resolution, and analysis time. There are two main types of columns, capillary and packed. Capillary columns are typically long (10 m or more), small diameter (internal diameter < 1 mm) fused silica tubes with a thin film of stationary phase coated on the inside. Packed columns are shorter and larger in diameter (internal diameter 2–4 mm). They are typically made of stainless steel or Teflon and packed with fine particles that serve as the stationary phase. It is important to choose a stationary phase that is appropriate for GHG separation. Multiple columns are often configured with automated valves to optimize analyte separation, direct analytes to the appropriate detectors, and avoid exposing columns or detectors to gases that can damage them (Mosier and Mack 1980; Sitaula et al. 1992; van der Laan et al. 2009). For example, constant exposure of the electron capture detector (ECD) to hydrogen sulfides (H_2_S) from saline wetland gas samples can compromise the cell and result in a radioactive leak. Three detector types are commonly used for GHG applications: thermal conductivity detector (TCD) for CO_2_, flame ionization detector (FID) for CH_4_, and ECD for N_2_O (Table 8). CO_2_ can also be quantified using an FID on a GC system equipped with a methanizer, a device that converts CO_2_ into CH_4_. The FID + methanizer is more sensitive than the TCD detector, allowing higher accuracy measurements of lower CO_2_ concentrations. This, combined with the smaller number of detectors required, results in GCs with two-detectors (ECD + FID) and a methanizer being more economical than three-detector GCs (ECD + FID + TCD) for most GHG applications. However, TCDs can detect a wide range of compounds and are non-destructive, making three-detector GCs a more flexible option. ECDs contain a radioactive source (typically nickel-63 [^63^Ni]) and must be licensed and periodically tested for radiation leaks.

Carrier gas selection is informed by several considerations including cost, safety, availability, and separation and detection efficiency. Traditionally, He gas has been used, but is increasingly scarce and expensive. H_2_ has many of the same characteristics as He, and is abundant, but can be a laboratory safety concern. N_2_ and Ar gases are also abundant but do not always work well with a TCD due to their thermal conductivities being similar to many gases of interest. Both N_2_ and H_2_ can be generated on site with the appropriate equipment.

*High-frequency gas analyzers and automated chambers:* High-frequency gas analyzers are often compatible with static chambers. These analyzers provide continuous changes in gas concentrations at sub-minute timeframes in situ for multiple gases, which increases accuracy, decreases post-processing time, and may allow researchers to increase spatial replicates. The shortened sampling time (e.g., < 10 min) helps limit chamber artifacts during deployment (e.g., warmed air, increased humidity and pressure, elevated gas concentrations altering flux rates) (e.g., Lin et al. 2021). Furthermore, high-frequency gas analyzers can be coupled with automated chambers, thereby providing continuous flux measurements over many hours to days (Petrakis et al. 2017; Diefenderfer et al. 2018; Capooci et al. 2019; Capooci and Vargas 2022a, b). Field-portable high frequency analyzers can also be used to measure headspace gas through direct injection into the device (Capooci and Vargas 2022b).

Using both automated chambers and high-frequency analyzers is relatively expensive (although see Duc et al. 2013, 2020; Bastviken et al. 2020 for low-cost options) and is limited to a reduced number of automated chambers (e.g., < 20) and to a restricted deployment distance. Long-term deployment of automated systems may be limited by battery life, the sensitivity of the equipment to environmental conditions (e.g., high atmospheric moisture content), and potential damage by snow, ice, floods, animals, or accumulation of salts in the equipment (in coastal systems). Overall, the application of these systems can provide high-quality information regarding the temporal patterns of GHG fluxes from wetlands at short (hourly) time scales.

Key Covariates and Ancillary Measurements:

*Air temperature:* Air temperature is necessary to correct GHG concentration measurements for the ideal gas law. Ideally, air temperature is measured continuously inside chambers, which should not be in direct sunlight to avoid heating above air temperature. However, if chambers are deployed over relatively short time periods and are opaque or insulated to avoid heating, then ambient air temperature may suffice as a proxy.

*Chamber collection and condition information:* Chamber volume, calculated from the footprint area and height from the top of the chamber to the soil or water surface, is necessary for calculating flux rates. Measures of height can vary due to microtopographic variation around the chamber, so multiple measurements are initially needed for an accurate, average height. In some systems or conditions, small changes in height can occur between sampling events, such as for chambers situated in shallow water. Therefore, chamber height may need to be measured more frequently. Careful notes on timing of gas sampling are needed for flux calculations as well. Ambient conditions (e.g., weather) and vegetation (e.g., vegetation cover, species or functional group, open water) should also be noted.

*Environmental conditions:* Several atmospheric, aquatic, and soil variables are useful in interpreting GHG fluxes, including but not limited to: air, water, and soil temperature, PAR, or other measures of incoming radiation, wind speed, salinity, water depth, DO, volumetric water content (soil moisture), redox potential, and pH. These and other common covariates are measured with various probes and sensors (Sections “Carbon in Wetland Soils”, “Carbon in Wetland Waters”). Calculation of water-filled pore space, another common covariate for GHG flux, requires additional soils data, including organic C or organic matter content (%), dry bulk density, and particle density (volumetric mass of solid soil).

*Partitioning flux into GPP, R_A_, R_H_:* Gases collected using clear chambers placed over plants provide information on NEE, which is the net effect of GPP minus R_A_ and R_H_. The clear chamber can then be darkened using shade cloths or with a second opaque chamber to measure ER (R_A_ + R_H_). GPP is calculated as ER – NEE (Hill and Vargas 2022). Care must be taken to allow adequate time for respiration rates to stabilize following darkening, and that a sufficiently dark shade cloth (e.g., black out roller blinds) is used to inhibit all photosynthesis. In addition, the Kok effect (i.e., light-inhibition of respiration) may introduce systematic bias, although this effect is rarely considered in wetlands (see Heskel et al. (2013) and Yin et al. (2020) for reviews of Kok effect). To partition ER into R_A_ and R_H_, plants can be clipped to remove R_A_. If aboveground plants are clipped to isolate R_H_, belowground roots from plants adjacent to the chamber may still contribute to measured CO_2_ flux. Trenching around a chamber footprint using a shovel or saw can remove/limit root respiration, but will initially cause disturbance.

*Leaf-level gas flux:* Photosynthesis and respiration can be measured directly from plant leaves using specialized portable infrared CO_2_ gas analyzers, which are dynamic chambers capable of maintaining or manipulating light, temperature, and CO_2_ concentrations for continuous non-invasive flux assessments of GHGs and water vapor (e.g., LI-6800 or LI-6400XT, LI-COR Biosciences). Some modules include chlorophyll fluorescence measurements to assess quantum efficiency of photosynthesis (e.g., light use).

*CH_4_ production in trees:* There is uncertainty as to whether CH_4_ within tree stems is produced in the soil and then transported through the tree stems (Barba et al. 2019a; Covey and Megonigal 2019) or if CH_4_ is produced inside the tree stems without any (or little) contribution from CH_4_ produced in soils (Barba et al. 2021; Smits et al. 2022). Automated measurements of CH_4_ (from soils and tree stems; Barba et al. 2019b) with ^222^Rn as a natural tracer (Megonigal et al. 2020) can be used to test whether CH_4_ produced in the soil is transported through tree stems. Incubation (Section “Laboratory Incubations”) of wood cores collected from active tree stems can be used to assess in situ CH_4_ production (Pangala et al. 2017; Barba et al. 2021; Smits et al. 2022) and oxidation inside tree stems (Zeikus and Ward 1974; Yip et al. 2019).

*CH_4_ production and CH_4_ oxidation using incubations:* Soil from chamber-flux locations may be collected to measure CH_4_ oxidation and production potential (as well as the production and consumption of numerous other gases such as CO_2_) in the laboratory by incubating fresh soil slurries or intact cores in jars, test tubes, or serum bottles (for details see Bridgham and Ye (2013), Inglett et al. (2013), and Section “Laboratory Incubations”). For CH_4_ production, incubations are conducted under anaerobic conditions by flushing containers with N_2_ or He, and then periodically collecting gas samples using a syringe through septa and analyzing samples using a GC. It is often assumed that CH_4_ production is linear over the time period of measurement (usually days) with only a final concentration sample collected, but rates can change over time (Ye et al. 2016), and it is recommended that multiple samples are collected, especially for longer incubations. For CH_4_ oxidation, incubations are conducted under aerobic conditions with periodic sample collection (usually hours). Different groups of methanotrophs (high-affinity and low-affinity) oxidize CH_4_ depending on the atmospheric concentration with unique rates, so it is important to carefully consider the initial CH_4_ concentrations (Meyer et al. 2020). The first-order kinetic rate constant is often determined under controlled-laboratory conditions and multiplied by field-based soil CH_4_ measurements to estimate oxidation rates (e.g., Meyer et al. 2020).

*Anaerobic oxidation of CH_4_:* Mechanistic studies may want to include anaerobic oxidation of CH_4_ (often referred to as ‘AOM’), the process whereby CH_4_ is converted to CO_2_ using alternate electron acceptors such as SO_4_^2−^, NO_3_^−^, metals, or soil organic matter (Gao et al. 2022), as opposed to aerobic CH_4_ oxidation which uses O_2_ as an electron acceptor. AOM has long been known to be an important process in marine coastal wetlands with high availability of SO_4_^2−^ (Knittel and Boetius 2009; Segarra et al. 2015). AOM can also influence freshwater wetlands (Martinez-Cruz et al. 2018; Gao et al. 2022). Thus, even under anaerobic conditions, observed net CH_4_ production is the balance between methanogenesis and methanotrophy. AOM is typically quantified using incubation studies by measuring labeled ^14^CH_4_ or ^13^CH_4_ and the corresponding production of labeled CO_2_ (Gupta et al. 2013; Segarra et al. 2015). It requires the use of specialized equipment to measure the labeled gases such as a gas radioactivity detector, GC, isotope ratio mass spectrometer, or cavity ring-down spectrometer. Like other incubation studies, it is important to preserve anoxic conditions of sediment samples and use ambient concentration of CH_4_ to estimate in situ AOM rates.

*CH_4_ production pathways through stable isotopes:* The relative fraction of ^13^CO_2_ and ^13^CH_4_ can provide information on anaerobic pathways of CH_4_ production in chamber-based measurements of wetland GHG. Acetate fermentation leads to enriched ^13^C and depleted deuterium (^2^H) compared to CH_4_ formed by the CO_2_ reduction pathway (Chanton et al. 2006). Measurements of δ ^13^CH_4_ can provide information on the degree of oxidation among treatments, with enriched ^13^C indicating greater CH_4_ oxidation (Bodmer et al. 2021). Gas samples for isotopic analysis can be sent to specialized laboratories.

*Boundary layer models to estimate diffusive flux:* If dissolved GHG concentrations in surface water are measured (Section “Dissolved Greenhouse Gases, Dissolved Inorganic Carbon”) concurrently with chamber fluxes (e.g., using the headspace equilibration method or automated measurements of *p*CO_2_ and *p*CH_4_), diffusive flux rates from the water can be distinguished using the boundary layer method (Liss and Slater 1974; Cole et al. 2010; Bansal and Tangen 2019). Gas flux across the water-atmosphere interface depends on two main factors: the concentration gradient between the water and the air, and the gas transfer velocity for a given gas at a given temperature. Values for gas transfer velocities can be derived from environmental variables including fetch length, wind speed, atmospheric pressure, temperature, salinity, wave action, and water velocity. However, gas transfer velocity values and boundary layer models are not consistent across studies and have primarily been parameterized in marine and lake systems (Wanninkhof 2014; Erkkilä et al. 2018). Consequently, modeled diffusive flux does not always correspond well with measured diffusive flux (Tian et al. 2021).

*24-h chamber deployments:* Traditional chamber measurements are based on short-term deployments (< 60 min) and multiple measurements during this period by manual sampling or high-frequency gas analyzers. These are good practices for vegetated surfaces as plants can respond quickly to changed conditions in the chambers, and for open water when focusing on gases with high solubility and at levels relatively near atmospheric equilibrium (typically < 20-fold supersaturation) making equilibration to chamber headspace occur relatively fast, such for CO_2_ or N_2_O. However, for open water CH_4_ flux measurements a large fraction of the aquatic CH_4_ flux occurs via ebullition, representing episodic, spatially scattered, very high fluxes. To capture such fluxes in representative ways it is beneficial to cover as much space and time as possible with sample collection. In addition, CH_4_ is commonly supersaturated 100- to 1000-fold in surface water, leading to equilibration taking a long time. It can take many days to reach equilibrium between the chamber headspace and water concentrations. For example, in a comparison between linear and non-linear CH_4_ flux calculations from 24-h flux chamber deployments, the difference was less than 10% (Bastviken et al. 2010). Furthermore, aquatic fluxes can exhibit diel cycles resulting in a bias if fluxes are measured during daytime only (Sieczko et al. 2020).

In the above context, and for floating flux chambers on open water where the water under the chamber is replaced continuously, chamber deployments spanning 24 h with measurements of chamber headspace gas content only at the start and the end of the deployment provide an opportunity to maximize space–time coverage of chamber measurements and ensure diel representativeness for open water CH_4_ flux measurements. The benefits of this strategy include less worktime spent on each chamber. The traditional approach with multiple manual measurements during the deployment time limits replication to a relatively few chambers at the same time. In contrast, the 24-h-multi-chamber approach allows deployments of many chambers simultaneously, thereby covering much more space and time of the system. The drawbacks include less information about what occurs in each individual chamber over time during the deployments and loss of capacity to directly detect bubbling events in each chamber. However, the multiple simultaneous fluxes generated by the 24-h-multi-chamber approach can be used to statistically separate ebullition (high fluxes and large heterogeneity between chambers) from diffusive flux (low fluxes with low heterogeneity between chambers) as described in detail in Bastviken et al. (2004). This statistical separation is not as precise as ebullition detection using funnel traps and, in general, the 24-h-multi-chamber approach yields less precise information from each individual flux chamber. However, it remains a favorable alternative when it can be assumed that the spatial heterogeneity and temporal variability in the whole studied system is of greater importance for overall flux assessments than the temporal flux variability in each chamber over the traditional short time periods. In other words, the choice between traditional short-term measurements and the 24-h-multi-chamber approach is a trade-off between prioritizing the precision in each measurement versus covering and assessing spatiotemporal variability in the whole study environment.

*Low cost GHG sensors for flux chamber measurements:* The recent development of inexpensive sensors suitable for use inside flux chambers (Bastviken et al. 2015, 2020) can make chamber measurements less dependent on expensive instruments and generates opportunities to overcome the above trade-off between short- or long-term deployments. Such sensors provide multiple measurements over time, thereby enabling detailed temporal data acquisition for each 24-h-multi-chamber. These inexpensive sensors can also be applied in conjunction with automated chambers. This also enables applying the 24-h-multi-chamber strategy for gases equilibrating faster with chamber headspaces than CH_4_ (e.g., CO_2_). In such cases, a 24-h chamber deployment yielding CH_4_ flux during the whole deployment period, will yield CO_2_ flux from sensor data close to deployment start only (as CO_2_ equilibrates fast). Later in the deployment when CO_2_ has equilibrated with the chamber headspace, the CO_2_ sensor data will give information allowing calculation of the surface-water concentrations using Henry’s Law. The use of low-cost gas sensors could thereby become important for monitoring programs targeting aquatic GHG fluxes in multiple ways.

*Hydrological inversion (stratification/mixing) of water column:* In some wetlands with deeper ponded water (> 0.5 m) that is not constantly flowing (such as in tidal systems), GHGs produced in wetland sediment can accumulate at the sediment–water interface during the day when the water column is stratified. Then, at night, when surface temperatures cool, the water column mixes or inverts, bringing GHG-rich waters to the surface for emissions to the atmosphere (Poindexter et al. 2016). Stratification and mixing patterns can be assessed using temperature sensors deployed at multiple depths throughout the water column (Holgerson et al. 2022), and ideally paired with dissolved GHG measurements using in situ CO_2_ or CH_4_ sensors, microelectrodes, or the manual headspace equilibration methods (Section “Dissolved Greenhouse Gases, Dissolved Inorganic Carbon”).

#### Eddy Covariance

**Fig. 21:**
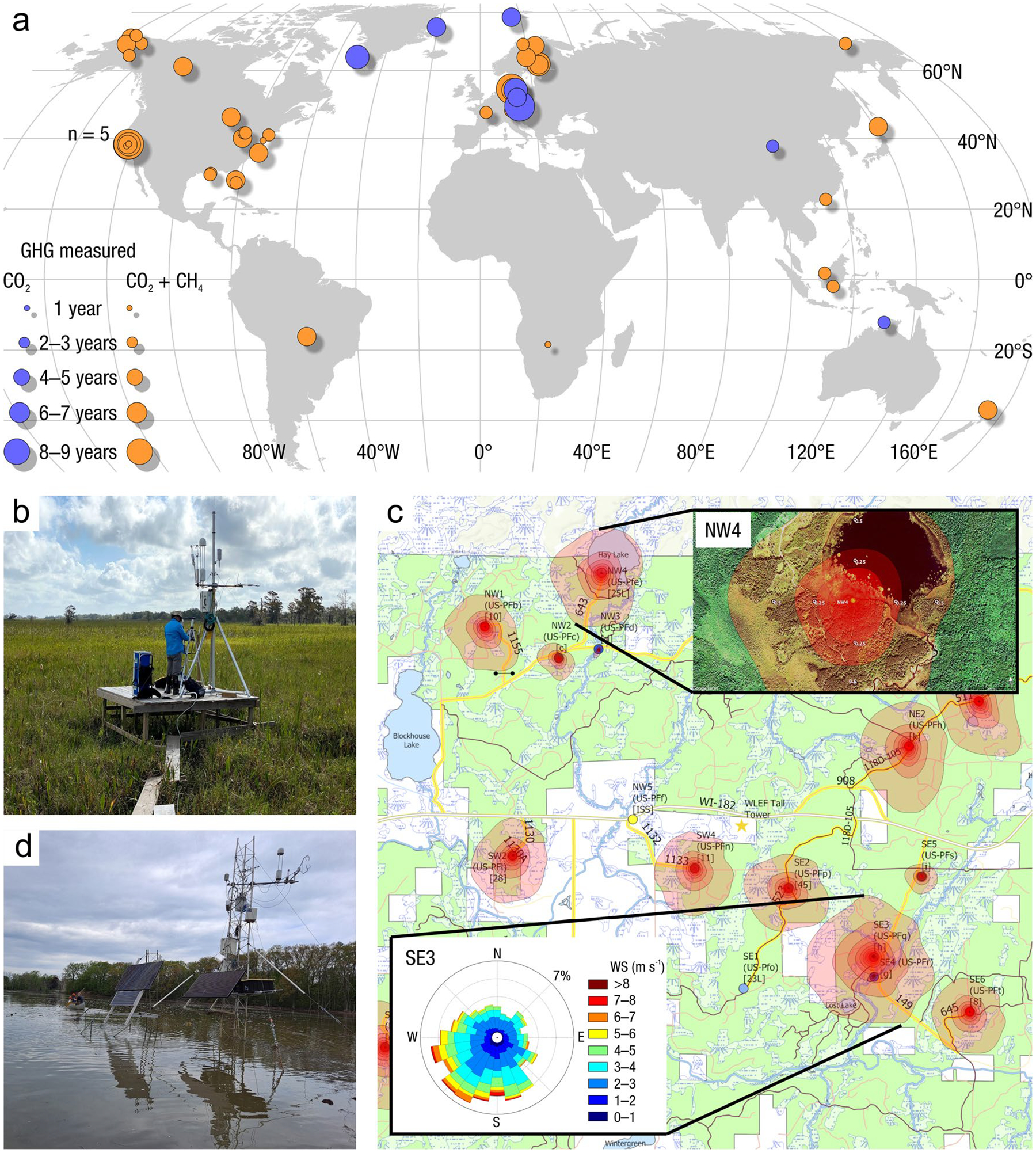
(**a**) Location of the 51 wetland tower sites from the FLUXNET-2015 and FLUXNET-CH_4_ databases that report eddy covariance (EC) measurements of carbon dioxide (CO_2_) and methane (CH_4_) fluxes (https://fluxnet.org/). The size of the dots represents the number of years of measurements, blue dots represent sites with CO_2_ fluxes only, and orange dot sites have both CO_2_ and CH_4_ fluxes; (**b**) an EC tower in the AmeriFlux network located in a freshwater marsh site in the Salvador Wildlife Management Area, Louisiana, USA (US-LA2); (**c**) site map of EC tower sites from the Chequamegon Heterogenous Ecosystem Energy-Balance Study Enabled by High-density Extensive Array of Detectors 2019 (CHEESEHEAD19; Butterworth et al. 2021) study. Red polygons represent June to October, 2019 EC tower footprint climatology, with red shading depicting relative contribution of spatial locations to total footprint; (c, top right inset) aerial image of lakeshore tower NW4 showing 90% footprint climatology in shading, with distance from tower noted in km, showing maximum fetch of 500 to 600 m from the tower, (c, bottom left inset) wind rose plot from site SE3 showing frequency of wind speed (WS) as a function of wind direction; (**d**) an EC tower of the Ameri-Flux network located in a flooded marsh site Old Woman Creek National Estuarine Research Reserve off of Lake Erie, Ohio, USA (US-OWC). Images with permission from Eric Ward (b); CHEESE-HEAD19 (http://cheesehead19.org) (c); base map of panel c produced by J. Mineau, University of Wisconsin with U.S. Forest Service base map; aerial image of site NW4 from Google Earth Imagery; footprint and wind rose plots by B. Butterworth, National Oceanic and Atmospheric Administration CIRES; and Gil Bohrer (d)

*What:* While many methods to measure wetland C pools and fluxes occur at the plot-scale, other methods are highly effective at capturing information at larger spatial scales (Baldocchi et al. 1988). The eddy covariance (EC) method is one of the few ways to directly quantify the net vertical exchange of GHGs, water, and energy, near-continuously at the whole ecosystem scale. EC instruments are mounted on ‘towers’ (e.g., a platform with scaffolding and other support structures) to measure high-frequency fluctuations of vertical wind velocity and the gas concentration of interest (e.g., CO_2_, CH_4_). Vertical fluxes are then calculated by averaging the covariance of fluctuations of vertical velocity and concentration of the gas of interest over a half-hour to hour period. One benefit of EC is since covariance is calculated on fluctuations, absolute accuracy of concentration and velocities is less important over having high precision and short time sampling intervals. EC measurements play an important and complementary role towards understanding wetland biogeochemistry for many reasons. This approach enables us to study wetland gas fluxes on time scales ranging from hours to days, seasons, and years. Furthermore, measurements capture a broad swath of land, integrating a wide area into a site-level measurement (Morin 2019). The horizontal dimension of the EC sampling area, referred to as the ‘flux footprint’, can extend tens to hundreds of meters upwind of the sensor and is continuously changing with prevailing conditions (Fig. 21; Rey-Sanchez et al. 2022). It is likely that some observations may be attributed to areas outside the wetland, which requires attribution and filtering of those data (see below).

EC measurements are well-suited for capturing the complex processes, emergent properties, and spatial heterogeneity inherent in wetland ecosystems. For example, the simultaneous measurements of CO_2_, water vapor, and CH_4_ help determine how recent photosynthate may prime soil microbial processes, how xylem transport may facilitate CH_4_ losses through vegetation, or the role that water stratification plays in the convective overturning or suppression of gas transfer (Poindexter et al. 2016; Sturtevant et al. 2016; Villa et al. 2019b; Ueyama et al. 2023).

EC towers not only provide GHG flux information at the site level, but also contribute to regional and global networks of GHG fluxes (Fig. 21a). Most regional flux networks (e.g., AmeriFlux, European Fluxes Database, AsiaFlux) facilitate an easy submission of flux data, allowing the data to be run through quality control, assigned a digital object identifier, and made publicly available in a standard format. Sharing data in this way has been a hallmark of the flux community and has allowed for significant global syntheses beyond what can be learned from a single site (e.g., Chang et al. 2021; Delwiche et al. 2021; Knox et al. 2021; Ueyama et al. 2023).

*Where:* There are many considerations when deciding where to locate an EC tower. Generally, towers should be placed in relatively flat, homogenous settings with uniform surrounding vegetation (Fig. 21b). Ideally, towers are placed close to the center of the wetland so that fluxes are measured from all wind directions and edge effects are avoided from nearby landscapes (e.g., adjacent grasslands) not of interest for wetland measurement. Relaxing this assumption requires further data screening or footprint analysis, and many small wetlands (< 10 ha) may not be suitable for EC. In some cases (such as prevailing winds coming from one predominant wind direction), towers are placed at the downwind edge of the wetland.

EC towers require a stable platform (e.g., dock or board-walk). A frequent problem in wetlands is finding good enough ‘footing’ for a tower platform. Towers are often placed on uplands adjacent to wetlands and the data are filtered for wind direction. Towers may also be placed on a clay lens that provides natural stability or using wooden boards or other construction materials to provide a solid foundation that can be anchored by guylines. An adequate power source is also important if using a closed-path system (see below *Hardware*).

The decision to establish an EC tower can be based, in part, on the location of other EC towers. There are many sites worldwide using the EC method to measure wetland (and upland) CO_2_ and CH_4_ fluxes (Knox et al. 2019; Delwiche et al. 2021). *Circa* 2023, there are 51 wetland sites included in the FLUXNET-2015 and FLUXNET-CH_4_ global databases (Fig. 21a), with standardized and gap-filled data available at: https://fluxnet.org/, with most towers in freshwater wetlands. Although the sites in the FLUXNET database span all continents except Antarctica, the majority are concentrated in North America and Europe, with a growing number of sites in Asia. Sites in the FLUXNET database cover a broad range of climates and a large fraction of wetland ecosystems (Knox et al. 2019; Delwiche et al. 2021), although tropical wetlands and southern latitude sites are notably underrepresented (Fig. 21a). An important objective of FLUXNET and regional flux networks is to increase site network representativeness by installing new towers in under-sampled regions (Villarreal and Vargas 2021). Therefore, increasing the number of EC tower sites in the tropics is particularly important since more than half of global CH_4_ emissions are estimated to come from this region (Dean et al. 2018; Saunois et al. 2020a). Additionally, compared to northern latitude wetlands, the biogeochemistry of southern latitude wetlands remains much less understood (Pangala et al. 2017).

*When:* One of the greatest advantages of the EC technique is its high temporal resolution of flux measurements. Raw data are recorded at 10 to 20 Hz (i.e., 10 to 20 times per second) and fluxes are averaged on a 30- to 60-min time step. Despite the continuous nature of EC data, in reality, year-round data are challenging to collect due to a number of logistical challenges. The maintenance of an EC tower is time- and labor-intensive, with frequent site visits recommended (e.g., ideally twice or more per month) in order to clean instruments and download data. Towers often need to be disassembled during winter months in areas with adverse weather conditions. Similarly, tower damage is a serious risk during extreme storm, hurricane, and fire events that can lead to data gaps. Also, instrument failure or power supply issues can lead to gaps in the data record. Even so, EC towers are typically deployed for multiple years at a single site. Once these data gaps are filled (see *Gap-filling* below), seasonal and annual budgets can be calculated by integrating GHG fluxes over time.

*Who:* EC measurements require considerable expertise in micrometeorology or biometeorology. Training is available and offered through universities as well as private companies (e.g., LI-COR Biosciences), and ready-to-use software packages (e.g., EddyPro, Tovi, REddyProc) for raw data processing, quality assessment, and flux data post-processing are available. Textbooks and other texts focused on biometeorology and EC theory are also recommended (Aubinet et al. 2012; Foken et al. 2012; Hicks and Baldocchi 2020; Burba 2022). With adequate training (~ 1-year intensive training), EC expertise is attainable even for wetland scientists without a micrometeorology background.

How:

*Hardware:* At the minimum, EC towers require: 1) a platform (Fig. 21b) (i.e., a tower or tripod); 2) a fast-response infrared (or related) gas analyzer to measure the GHG of interest (CO_2_, CH_4_, water); 3) a sonic anemometer to measure high-frequency 3D wind velocities; 4) standard meteorological sensors for temperature, humidity, pressure, and shortwave radiation; 5) data logging systems; 6) power supply to continuously log, store, and transmit data. Tower height is usually at least several meters above the height of vegetation to sufficiently resolve turbulence, preferably around 150% of canopy height (see Munger et al. (2012) and Burba (2022) for more detailed calculation and overview). The taller the tower, the larger the sampling footprint, so there is a tradeoff in constraining the sampling footprint to the wetland versus maintaining sufficiently turbulent flow to meet assumptions for EC. Towers come in a wide variety of sizes and forms, but ideally they are small enough in diameter to not significantly distort water or air flow, while sturdy enough to minimize vibration and keep the instrument position extremely steady. Sonic anemometers are typically mounted on 1-m booms pointed into the predominant wind direction. Most towers require several solar panels depending on local conditions and power draw of the gas analyzer, pumps, and communication systems. It is possible to transmit raw, high-frequency (10–20 times per second) data by cellular networks if 4G or better coverage is available. Otherwise, to collect high-frequency data, data cards will need to be swapped at some regular interval (usually biweekly).

All flux towers are susceptible to lightning strikes and power surges, even more so in exposed wetlands. Lightning damage prevention is one of the most time-consuming and labor-intensive parts of EC tower installation. The most basic protection is to add lightning rods or a dissipater to the top of each flux tower. Additional protection is typically provided with metal grounding rods added at guyline anchors, preferably in periodically dry environments since wetland water provides a channel for electricity flow. Some lightning protection kits include all necessary materials for flux tower protection.

*Gas analyzers:* Gas analyzers can either be enclosed (closed-path) or exposed (open-path). Both options have their specific advantages and disadvantages; closed-path analyzers are not as prone to disturbances in the measurement path (e.g., through water or dust), and many offer a wide variety of trace gas species besides CO_2_ and CH_4_ (e.g., carbon monoxide [CO], N_2_O). Closed-path sensors are usually installed in a small building located near the tower base. Closed-path systems have higher power needs to continuously draw air from the tower top through tubing using pumps. Therefore, their operation often is generally not possible at remote sites without line power or permanently running generators. Open-path gas analyzers for the measurement of CO_2_ and CH_4_ in the atmosphere are mounted close to the sonic anemometer on the tower itself and therefore do not need additional pumps. The calculated fluxes of gases like CO_2_ and CH_4_ need to be corrected for density fluctuations in temperature and humidity (Chamberlain et al. 2017). Over wetlands, these corrections, which are physically based, are relatively small when evaporation is high and sensible heat flux is low. While open-path analyzers have lower power requirements, they also are more sensitive to density corrections compared to close path systems, and need regular calibration and cleaning depending on pollen, birds, dust, and moisture (Massman and Lee 2002). Data quality from open-path sensors can therefore be a problem that requires detection and removal of data based on sensor quality control flags and sensor maintenance. A leaf wetness sensor is another low-cost option to detect periods of extensive condensation or riming. Coastal wetlands may face additional challenges in the form of salt spray, tides, and storm surge.

Fortunately, a number of manufacturers have standardized much of the instrumentation and infrastructure for EC measurements so that most components can be purchased from a single vendor (e.g., LI-COR Biosciences, Los Gatos Inc., Picarro Inc., Aerodyne Research Inc.), including data logging programs. Similarly, EC flux processing software is free and widely available (e.g., EddyPro from LI-COR Biosciences).

*Assumptions of the EC footprint:* There are many assumptions which need to be addressed in real-world application of the EC method in a wetland. Foremost, there is the assumption of a horizontally homogenous, flat surface area of interest. This assumption is needed to establish an internal boundary layer that contains a constant flux layer. Most wetlands can meet the horizontal criterion since they are relatively flat. On the other hand, the mosaic of water, vegetation, mudflats, tussocks, and hollows can form a very heterogeneous source-sink environment. This condition bends the assumption of a homogenous source or sink environment. A recent study showed only half of existing wetland EC towers were representative of their surrounding landscape (Chu et al. 2021). The long-term pattern of the footprint (i.e., footprint climatology) may not reflect the vegetation distribution of the entire wetland. Flux is not just a function of the fraction of water, vegetation, and soil in the footprint, but also the spatial distribution of those patches (Hatala Matthes et al. 2014). Hence, interpretation of fluxes from wetlands generally requires analysis with flux footprint models and ideally high-resolution remote sensing over the footprint.

Some wetlands are relatively small in size and may violate assumptions about homogeneity of landscape required for EC towers. Also, for larger wetlands, spatial heterogeneity in sources and sinks may be important to consider for flux interpretation, particularly if the length scale of heterogeneity is equal to or larger than the field of view of the EC sensors. Seasonal and diel shifts in wind direction may change the footprint of towers away from the wetland site of interest.

Wind speed and variance, friction velocity, and sensible heat flux are required for running most flux footprint filter models (Schmid 2002) to identify, for each time period, what area of the ground contributed to the flux (Fig. 21c). Common calculated metrics include percentile footprint or upwind fetch, for example, 50%, 80%, or 99% footprint, representing the percentage of the area of ground that contributes to the flux (Fig. 21c). The data are rejected (i.e., filtered) if the footprint lies mostly outside of the wetland. Rey-Sanchez et al. (2022) presented a technique for mapping spatial heterogeneity and detecting CH_4_ hot spots within an EC tower footprint. A footprint climatology across a season or year can be estimated by superposition of half-hourly footprint areas over a time period, which can estimate the ‘airshed’ of the flux measurement. More advanced techniques to better understand and attribute EC flux measurements, including using chamber-based flux measurements in the EC footprint (e.g., Forbrich et al. 2011; Morin et al. 2017; Rey-Sanchez et al. 2018; Hill and Vargas 2022), using multiple, nested towers (e.g., Helbig et al. 2017), or advanced statistical approaches (e.g., Xu et al. 2017; Griebel et al. 2020), may be necessary for more complex landscapes (e.g., polygonal tundra, lake-wetland complexes, coastal wetlands) or small-scale wetlands.

*Meteorology:* Fortunately for gas flux measurements using EC towers, many of the key atmospheric covariates (e.g., air temperature and humidity) needed for calculating flux are already measured. A sonic anemometer measures wind speed and direction, which is used to determine friction velocity and sensible heat flux. Measurements of water vapor are used to derive latent heat flux (also known as evapotranspiration). It is often recommended to measure temperature and humidity separately with independent sensors to fill in data gaps due to quality control issues during precipitation or low turbulence. In addition, a measure of light is important, either from PAR sensors that measure PPFD in μmol m^−2^ s^−1^ or incoming broadband all-sky solar radiation in watts m^−2^. While PPFD and all-sky solar radiation are highly correlated, there are subtle differences that make it worthwhile to measure both. Barometric pressure is also recommended, both for air density corrections and for detection of pressure pumping events, where rapid drops in atmosphere pressure, such as during weather fronts, lead to significant CO_2_ or CH_4_ emissions (Knox et al. 2021).

*Data filtering:* With the basic set of measurements, quality control of EC data for systematic error can be performed following standard protocols for filtering data during periods of low turbulence using friction velocity, non-stationary conditions, and poor sensor clarity, as implemented in most common EC software packages (Mauder et al. 2013). Not filtering for these conditions can lead to large systematic bias in flux estimates (> 50%), especially at night or winter, due to the challenges of EC flux measurements during low turbulence. Random errors from measurement noise and turbulence can be large at short time scales, from 10% up to 40% (Hollinger and Richardson 2005; Salesky and Chamecki 2012; Post et al. 2015), but average out to small values (a few %) over days to seasons. Propagating measurement error, sampling error from turbulence, and gap-filling uncertainty is useful for providing uncertainty ranges for annual C and GHG fluxes (Pastorello et al. 2020).

Most EC flux measurements also suffer from lack of ‘energy balance closure’; that is, the sum of turbulent heat fluxes (sensible and latent heat) does not balance available net radiation (sum of net solar and long wave radiation) and surface heating (Mauder et al. 2020). Measurements of net radiation, either directly or by measuring all four components (incoming and outgoing solar and longwave radiation), along with measurement from at least one soil heat flux plate, can be used to estimate the energy balance and possibly identify periods of poor closure for filtering. Soil heat flux can be challenging to measure in wetlands, and might be substituted by a vertical profile of soil and water temperature measurements (Drexler et al. 2004).

*Storage flux and other air flow considerations:* For taller towers (> 10 m), there may be cases where ‘storage flux’, the net accumulation of gases below the sensor height, is an important term for estimating net GHG flux (Xu et al. 2019a), especially at hourly timescales. Within-site air flows, typical around rough surfaces and temperature transitions (such as water-to-land) can create lateral air flows under the tower and bias EC measurements; correction methods for these air flows are limited (Higgins et al. 2013; Kenny et al. 2017). To assess storage flux requires vertical profiles of the scalar of interest (e.g., CO_2_ concentration, air temperature, or water vapor content) along a tower (typically ~ 1 per 10 m, usually clustered near the surface). Measurements of storage flux is recommended for wetlands with tall vegetation (e.g., forested wetlands), closed canopies, or non-flat topography, especially if half-hourly or diel cycle analysis is a research objective.

*Gap-filling:* Gaps in the data record are effectively unavoidable. Gaps are usually associated with low turbulence nighttime conditions, winter months, or instrument failure, leading to rejection of data. The frequency of gaps is higher for some times of day (i.e., more at night), seasons (i.e., more when cold), and meteorological conditions (i.e., more when raining). These periods are associated with different flux rates than the average, which then makes the average of observed (no gaps) data biased towards daytime, summer, and clear weather. Gap-filling is therefore necessary to obtain an unbiased estimate for daily, seasonal, and annual fluxes (Moffat et al. 2007).

The large amount of flux and covariate data from EC measurements allows for gap-filling, the process of imputing missing flux and meteorological data (Moffat et al. 2007). Most gap-filling methods rely on flux covariates of temperature, PPFD, or solar radiation, and sometimes humidity or vapor pressure deficit (e.g., see Wutzler et al. 2018; Pastorello et al. 2020). For sites where the water table is below the soil surface for some part of the year, a high-resolution micro-topography map together with measurements of soil temperature and soil moisture enable estimates of decomposition activity that may be useful for gap-filling and partitioning. Methods for reliably filling gaps, particularly short gaps occurring at night, are well established, especially for CO_2_ and water (Moffat et al. 2007).

Gap-filling CH_4_ fluxes for wetlands may require additional covariate information such as water table depth and more elaborate approaches due to non-linear relationships between covariates and CH_4_ flux (Kim et al. 2020; Irvin et al. 2021). The random forest machine learning algorithm had greater accuracy and less bias for gap-filling CH_4_ fluxes compared to marginal distribution sampling, artificial neural network, and support vector machines (Kim et al. 2020; Irvin et al. 2021). The more commonly used marginal distribution sampling approach for gap-filling CO_2_ fluxes can be adapted to use different input variables for CH_4_ gap-filling; however, performance is poorer than the best machine learning models (Irvin et al. 2021). There is greater uncertainty with gap-filling lagged effects of CH_4_ flux, such as a short-term emission spikes after an extreme precipitation event or during ebullition. Estimates of uncertainty from both random error and gap-filling need to be propagated and reported alongside annual budgets.

*Partitioning fluxes:* A unique feature of net CO_2_ fluxes measured by EC is that they are amenable to partitioning into component fluxes of GPP and ER for all hours of the day using a variety of approaches (Desai et al. 2008), though there is inconsistency among studies regarding which standardized algorithm to apply (Pastorello et al. 2020). Many of the partitioning algorithms are calibrated and designed for non-wetland sites, and may not be appropriate for wetland sites. Other emerging techniques rely on carbonyl sulfide measurements, solar-induced fluorescence, stable C isotope partitioning, and high-frequency decomposition, but require additional ancillary measurements such as estimates of water use efficiency or archived high-frequency observations (e.g., Scanlon and Kustas 2010; Fassbinder et al. 2012; Sulman et al. 2016; Li et al. 2018b; Spielmann et al. 2019). For example, stable isotope partitioning uses flux measurements of ^13^CO_2_ to calculate isoflux (i.e., the product of NEE and the δ^13^C of NEE; Bowling et al. 2001; Wehr and Saleska 2015; Oikawa et al. 2017), which can be used to mathematically solve for GPP and ER. However, isotopic modeling requires assumptions regarding C fractionation, as well as specialized sensors (e.g., quantum cascade laser spectrometers). Partitioning latent heat flux measurements into evaporation and transpiration is also important for understanding processes driving GHG fluxes (Stoy et al. 2019). If woody vegetation is present, sap flux measurements of plant water use can be used to partition evapotranspiration.

*Key Covariates and Ancillary Measurements:* Ancillary measurements are important for interpreting, gap-filling, and modeling EC GHG flux measurements. Also, measures of additional variables within the footprint can help discern the underlying mechanisms controlling net flux rates. Many of these ancillary measurements, such as environmental conditions, are described in more detail in Section “Chamber Measurements”.

*Soil in the EC footprint:* Periodic surveys for soil/peat depth, C content, and possibly other elements (e.g., organic N, Fe, S, Ca, Mg) may provide important information on long-term C accumulation, total C pools, peat compaction rates, and correction of water table depth for compaction. By combining soil core data with EC data, the C lost through lateral hydrological flow can be estimated (Forbrich et al. 2018; Bogard et al. 2020a). Measurements of soil moisture, redox potential, and salinity also help interpret GHG fluxes. Details of measurements of C constituents and covariates in soils are described in detail in Sections “Carbon in Wetland Soils” and “Carbon in Wetland Waters”, and “Chamber Measurements”.

*Water in the EC footprint:* Measurements of water depth and volume, temperature, DO and dissolved GHGs, and chemistry are key ancillary measurements in EC footprints (Section “Carbon in Wetland Waters”), as are lateral hydrological fluxes (Section “Lateral Flux”). Combining high frequency in situ water chemistry analyzers with EC flux data is a robust technique to understand mechanisms of flux. For coastal or tidally influenced sites or inland saline wetlands, measurements of water salinity and DO are especially important for studies on CH_4_ flux. Periodic samples of DOC, POC, and DIC within the wetland and its inflows and outflows may be useful at some wetland sites to estimate lateral fluxes (Webb et al. 2019; Bogard et al. 2020a; Arias-Ortiz et al. 2021), which can account for 10 to 80% of NEE across a range of wetland types (Webb et al. 2019; Bogard et al. 2020a).

*Vegetation in the EC footprint:* The vegetation community in a wetland is a primary factor influencing GHG fluxes. Over an EC footprint, vegetation can range from being sparse with only floating and submerged vegetation, to dense monocultures of emergent macrophytes, or forests with dense tree canopies and mixes of understory species and functional types. Therefore, a comprehensive survey of vegetation composition (and ideally biomass, NPP, and phenology) across the footprint is extremely useful in discerning mechanisms driving the magnitude and temporal patterns of GHG fluxes (Sections “Carbon in Wetland Vegetation” and “Net Primary Productivity”). Spatial data on vegetation also provides the information needed to interpret variation due to changes in flux footprints.

It is possible using digital cameras to capture seasonal growth of vegetation, where images are analyzed based on the fraction of green pixels relative to red and blue (Richardson et al. 2018). The greenness index extracted from digital images can be helpful for understanding the phenology of the system (Vázquez-Lule and Vargas 2021) and for providing a proxy for photosynthesis in GPP models (Knox et al. 2017); it also is useful for including in machine learning gap-filling algorithms. In addition, airborne imagery, such as National Agriculture Imagery Program (NAIP, Fig. 21c), or satellite-derived greenness indices such as the Normalized Difference Vegetation Index (NDVI) from the Moderate Resolution Imaging Spectroradiometer (MODIS), Landsat, or Sentinel can be used to assess phenology, biomass, and LAI in the EC footprint (e.g., Ju and Bohrer 2022), albeit in some cases satellite resolution is larger than the footprint (Section “Remote Sensing”; Hill et al. 2021).

*Chamber measurements in the EC footprint:* GHG flux chamber measurements for CO_2_ or CH_4_ are also useful for evaluating EC fluxes and partitioning components of the C budget (Fig. 20). Auto-chambers with high frequency analyzers that continuously measure C fluxes at one location may provide a strong constraint on decomposition rates, though power needs and placement on aqueous surfaces may complicate their use in wetlands. Surveys with soil collars and floating chambers across key vegetation types, within the flux tower footprint, and in all directions from the EC flux tower are highly useful for diagnosing homogeneity of the tower measurement and scaling up to wetland scale C fluxes. Accurate logs of the location of the chambers relative to tower are highly recommended. Similarly, bubble traps for CH_4_ ebullition may be worthwhile at some sites. Additional measurements of CO_2_ and light response curves can also be useful for EC flux interpretation.

*Albedo:* Aside from the effects of wetland GHG fluxes on climate, wetlands can also affect climate through their effect on albedo, the proportion of radiation or light that is reflected without being absorbed by a surface. The albedo of wetlands influences local surface temperatures and can reduce heat extremes. Low albedos over wetlands indicate that solar energy is absorbed by wetlands, which will mean there is more energy available to evaporate water that can result in localized cooling (i.e., transforming solar radiation to latent heat). Despite the potential role of wetland albedo, it is not yet directly integrated into IPCC reports (Sumner et al. 2011; Hesslerová et al. 2019; Wu et al. 2021). There are efforts to normalize the effects of albedo in CO_2-eq_ (Bright et al. 2015; Bright and Lund 2021). Albedo can be measured with upward and downward looking shortwave radiation sensors, common in many EC installations (Helbig et al. 2020).

*Aquatic eddy covariance:* Inspired by EC work at the terrestrial atmosphere-surface interface, the EC technique has been successfully modified to measure in situ DO fluxes underwater between benthic surfaces and the water above (Berg et al. 2003, 2022; McGinnis et al. 2008; Glud et al. 2010; Long et al. 2013; Attard et al. 2014; McCann-Grosvenor et al. 2014; Chipman et al. 2016). The technique allows benthic DO fluxes to be measured under naturally varying field conditions – a crucially important feature because most drivers of benthic exchange (e.g., current flow, wave action, light) are excluded or altered in traditional flux methods (Berg et al. 2003). Aquatic EC measurements of true in situ DO fluxes have, for example, provided new information on metabolism and C sequestration from seagrasses (Rheuban et al. 2014; Long et al. 2015a; Berg et al. 2019; Berger et al. 2020), coral reefs (Long et al. 2013), canopy-forming macro-alga beds (Attard et al. 2019; Volaric et al. 2019), and oyster and mussel reefs (Volaric et al. 2018; Attard et al. 2020).

Historically, concentrations of DO have been measured with Clark-type microelectrodes (Revsbech 1989) with a response time (*t*_90%_) of 0.2 to 0.3 s (Berg et al. 2003; Kuwae et al. 2006; McGinnis et al. 2008). Because microelectrodes are highly fragile, more robust optical fiber sensors (Chipman et al. 2012; Long et al. 2015b; Huettel et al. 2020) or miniature planar O_2_ optodes (Berg et al. 2016) are often used. A standard instrument for measuring the 3D velocity field above the benthic surface is an acoustic Doppler velocimeter that records the velocity at a speed up to 64 Hz. Under typical field conditions, one flux estimate can be derived from 15 min of data measured 5 to 30 cm above the benthic surface (Lorrai et al. 2010; Berg et al. 2017), and it typically represents a sediment surface area between 10 to 100 m^2^ (Berg et al. 2007). Due to unique challenges encountered underwater, such as biofouling of sensors and sensor breakage (Chipman et al. 2012, 2016; Huettel et al. 2020), continuous aquatic EC measurements are usually limited to a few days, thus compiling several individual deployments is needed for longer temporal coverage (Berger et al. 2020). Most steps in aquatic EC data processing have been adopted from standard protocols used in the atmospheric boundary layer method (Berg et al. 2009, 2022; Lorrai et al. 2010; Reimers et al. 2012).

Measurements of atmosphere-water gas exchange are a new application of aquatic EC (Berg et al. 2017; Berg and Pace 2017; Long and Nicholson 2018). By using the same sensors as for the benthic environment but positioning them upside down immediately below the atmosphere-water interface, fluxes of O_2_ can be measured. Moreover, by combining the atmosphere-water gas flux with the measured bulk gas concentration in the surface water, the standard gas exchange coefficient, *k*_600_, can be derived and translated to a coefficient for any gas of interest (Berg and Pace 2017) including CO_2_, CH_4_, and N_2_O. Atmosphere-water fluxes determined this way generally have greater accuracy than those estimated from empirical and theoretical relationships (Berg and Pace 2017; Berg et al. 2020). The atmosphere-water gas flux rates and *k_600_* coefficient can be measured from a moving platform in lakes, reservoirs, and ponds with near-stagnant water (Berg et al. 2020).

*Diel oxygen method and stable isotopes to measure aquatic ecosystem metabolism:* The term ‘aquatic ecosystem metabolism’ refers to cycling of organic materials in the water column (i.e., GPP, ER, NEP), which is affected by light, temperature, and water depth (Jankowski et al. 2021). For locations where EC is cost prohibitive or other considerations such as footprint or topography violate EC assumptions, the diel O_2_ method is a relatively low-cost method that uses high frequency daily fluctuation in DO concentration to estimate aquatic GPP, ER, and NEP (Odum 1956b; Staehr et al. 2010; Hall et al. 2016; Jankowski et al. 2021). Several methods can be used to calculate GPP, ER, and NEP; some examples are: the light–dark shift technique for GPP (Revsbech and Jørgensen 1983); rate of nighttime O_2_ change, and water-atmosphere O_2_ exchange for ER; simple subtraction of the two in terms of daily rates results in NEP (Swaney et al. 1999); and more complex Bayesian statistical models (Holtgrieve et al. 2010). Although the diel O_2_ method is considered robust and suitable for systems with deeper waters (e.g., lakes; Holtgrieve et al. 2016), the need for multiple sensors to capture spatial variation, inability to measure anaerobic respiration, inability to incorporate gas exchange of emergent vegetation, and highly variable diel O_2_ patterns are disadvantages of the technique, particularly in shallow wetlands (Staehr et al. 2010).

The concentration of CO_2_ measured by partial pressure (*p*CO_2_) in wetlands can also provide estimates of NEP, or information on contributions of microbial respiration in groundwater and soil to whole catchment CO_2_ budgets (Jones and Mulholland 1998). *p*CO_2_ can also be affected by geochemical inputs (e.g., carbonate weathering; Stets et al. 2009; Marcé et al. 2015). Combined DO and *p*CO_2_ data provide a more complete picture for investigating net changes in biological activity and abiotic processes (Bortolotti et al. 2016; Vachon et al. 2020). The photosynthetic and respiratory quotients (mol O_2_ to mol C) are important parameters for estimating NEP, which can vary widely in pelagic systems (Williams et al. 1979; Berggren et al. 2012). Constraining these terms at the entire ecosystem scale is challenging, and often a fixed value is assumed.

Measurements of the isotopic composition (delta oxygen-18 [δ^18^O]) of DO and water can be used to quantify GPP, respiration, and NEP (Bocaniov et al. 2012; Bogard et al. 2017). While the technique has limitations related to assumptions of steady state conditions in mass balance calculations, it yields accurate estimates under most conditions (Bogard et al. 2017, 2020b). A new application for upscaling aquatic metabolic rates involves pairing isotopically derived estimates of GPP with independent observations of remotely sensed water color in shallow surface waters (Bogard et al. 2019; Kuhn et al. 2020).

### Litter and Organic Matter Decomposition

#### Definitions and Units

**Fig. 22:**
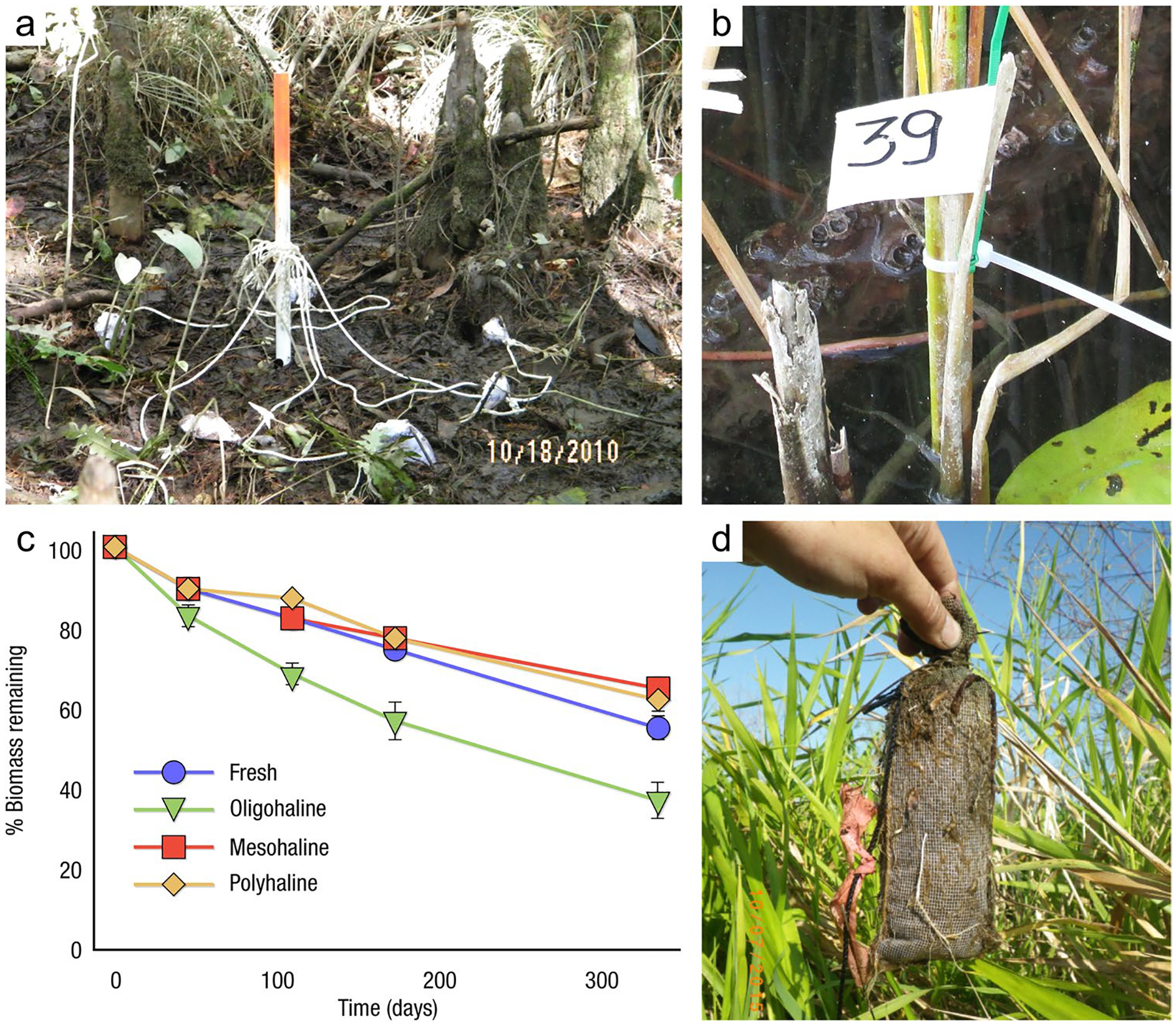
(**a**) Litter bags placed on the sediment surface and tied to a center pole to keep samples in place during periods of high water and to help locate bags during recovery of samples in a tidal freshwater forested wetland in South Carolina, USA; (**b**) physically tagged standing litter in Lake Dagow, Germany; (**c**) graph of biomass remaining in litter bags over time in fresh, oligohaline, mesohaline, and polyhaline marshes in Louisiana, USA (Stagg et al. 2018); (**d**) litter bag retrieved from a freshwater herbaceous marsh in Louisiana, USA. Images with permission from Camille Stagg (a, c, d) and Manuela Abelho (b)

*Definitions:* Decomposition is the physical breakdown and biochemical transformation of organic matter found in wetland vegetation, water, and soil C pools. The rate and extent of organic matter decomposition depends on interactions with the physical, chemical, and biological conditions in the environment, as well as the molecular complexity of the organic matter (Schmidt et al. 2011; Lehmann and Kleber 2015; Stagg et al. 2017a). For many wetland plants, significant quantities of standing-dead litter can accumulate aboveground before reaching the sediment surface (Fig. 22) (e.g., Christian et al. 1990; Asaeda et al. 2002). Thus, the natural sequence of plant litter decomposition begins in the aerial standing-dead position or floating in the water column, then at the sediment surface, and finally centimeters to meters belowground in the soil. In general, wetlands accumulate disproportionally large quantities of C in soil due to prevalent anoxic conditions that reduce decomposition rates (Mcleod et al. 2011).

Decomposition processes in wetlands involve a complex array of detrital consumers (e.g., invertebrates, archaea, bacteria, fungi) and environmental conditions (e.g., hydrology, O_2_ and nutrient availability, temperature, pH). Plant litter is first shredded by invertebrates and other animals (e.g., fish; Simon et al. 2019) and then decomposed further by extracellular enzymes produced by microbes, resulting in the breakdown of chemical constituents such as lignin and cellulose, and the formation of CO_2_ and CH_4_, microbial biomass, and small molecular weight breakdown products such as dissolved and particulate organic matter (Gessner et al. 2010). The rate at which these transformations take place in wetlands is strongly influenced by the types, quantities, and activities of the detrital consumers present, their response to prevailing environmental conditions, the chemistry of the plant litter resources they are metabolizing, as well as the myriad potential interactions that may occur within and between different decomposer groups within the detrital food web. Plant litter from various allochthonous and autochthonous sources may be quite diverse and vary in chemical quality, physical characteristics, and the time to become available to detrital consumers (e.g., Middleton and McKee 2001).

*Units:* Decomposition of organic matter, typically plant material, is measured and expressed as the quantity of plant mass remaining compared to the initial mass over a specified time period (Fig. 22c) (e.g., % dry mass remaining or % ash-free dry mass remaining per unit time). The rate of plant mass loss is typically determined using an exponential decay model (M_t_ = M_o_ × e^−kt^), which can also be expressed as a linear regression model after natural log transformation (ln (M_t_ / M_o_) = − kt + b), where M_t_ is the mass remaining at time t (days), M_o_ or b is the calculated initial mass remaining at time 0 (which are ideally close to 100%), and k is the decay rate coefficient (i.e., rate of loss or slope) (Bärlocher 2020). In some cases, often where accumulated mass loss is high, the exponential decay model may not be the most appropriate to represent the change in mass over time. In these cases, double (or more) exponential or asymptotic models can be considered (Berg and Laskowski 2005). Multiple decay models are sometimes needed if decomposition rates change over time (Wider and Lang 1982). Once determined, decay rates can be compared between plant species or wetland habitats. Decomposition can also be measured using standardized substrates, such as cotton strips instead of native plant material (Maltby 1988; Tiegs et al. 2019). When using cotton strips, decomposition is expressed as the percent loss in tensile strength over a specified time period (days to a few weeks), which is measured as the force, in Newtons, required to tear experimental strips following field incubation minus the mean force required to tear reference strips that were inserted in the field and immediately removed (Latter et al. 1988; Verhoeven and Arts 1992; Slocum et al. 2009). Decomposition of SOC is typically expressed as the amount of C respired as CO_2_ and CH_4_ per mass of soil or mass of initial C (e.g., mg C respired per g soil; % soil C respired). Decomposition can be quantified using laboratory incubations or in the field and expressed as a rate (as with litter bags) or total C loss through respiration.

**Rationale:** The production and decomposition of organic matter is a fundamental, biogeochemical process in all ecosystems (Moore et al. 2004; Berg and Laskowski 2005; Hagen et al. 2012), and as such, considerable research has examined decomposition processes within a variety of wetland habitats (e.g., Webster and Benfield 1986; Yarwood 2018; Stoler and Relyea 2020; Xia et al. 2021). Understanding the fate of wetland organic material is crucial for modeling of detrital pathways that are central to C cycling and ecosystem energy flow. Changing rates of wetland decomposition from warming temperatures, altered hydrological regimes, and sea level rise will undoubtedly affect how wetlands contribute to the global climate.

#### Mass Loss of Litter

*What:* Various in situ methods are used to quantify and understand the environmental drivers of decomposition. Field decomposition experiments can use native plant litter or standardized substrates such as cotton strips or tea bags (e.g., Mueller et al. 2018). Native plant litter substrates provide more realistic rates of decomposition, while standardized substrates are more comparable among wetland studies. However, decomposition outcomes of standard substrates are not necessarily equal to that of native plant litter or organic matter. Decomposition rates determined using either native plant litter or standardized substrate can be correlated to climate and other environmental factors (Fig. 22c) (e.g., trophic status, edaphic conditions). When litter decomposition experiments are paired with GHG measurements (Section “Greenhouse Gas Fluxes”) and lateral flux (Section “Lateral Flux”) measurements, the data can help partition the quantities of C that: 1) accumulate in the soil profile; 2) are quickly returned to the atmosphere; and 3) are laterally moved to an adjacent land cover type.

*Where:* In situ experiments using litter bags or related approaches can occur on the soil surface, subsurface, or in the aerial standing environment (Fig. 22a, b). Because litter decomposition is subject to topographical and microclimatic variation, it is ideal that litter is collected, deposited, and examined in a way that mimics the natural decomposition processes. Replicate locations of litter deposits are needed within a site for site-level characterization.

*When:* Decomposition experiments using native plant litter or standardized substrates are often conducted during the growing season. Litter bags are typically incubated for weeks to months, but experiments can also last for one or more years. Standardized substrates such as cotton strips, composed of rapidly degradable cellulose, are generally incubated for days to weeks. The length of time is dependent on the substrate and the environmental conditions. If multiple time points are desired, then replicate samples within a site are retrieved sequentially for destructive analysis.

*Who:* Protocols for assembling and deploying litter bags for mass loss experiments are relatively straightforward for technicians following training. The decisions regarding litter bag material and mesh size, substrate (litter) material, and deployment location require subject-matter expertise and local knowledge of the study area. One of the appealing features of decomposition experiments using litter bags is that they are accessible to non-experts. In fact, many citizen-science projects (e.g., TeaComposition H_2_O, Keuskamp et al. 2013) have included litter bag deployments. The interpretation of data, modeling decomposition rate, and ancillary measurements can be more involved and require statistical expertise.

How:

*Standing litter tagging:* For plants such as emergent macrophytes or floating-leaf macrophytes, if plant material is prematurely collected in a living green state or following early senescence and enclosed in litter bags, it is unlikely to represent naturally occurring decomposition processes and thus bears a risk of introducing artifacts. An approach to examine decomposition of standing dead or floating litter is to follow mass loss of shoots that have been physically tagged in a non-destructive natural position (Klok and van der Velde 2017; Kuehn and Gessner 2020). Physically tagged shoots (Fig. 22b) can be periodically collected and the mass loss of specific plant organs (leaf blades, leaf sheaths, stems) determined based on declines in either area-specific mass (g cm^−2^) (e.g., Gessner 2001) or other morphometric measures used to estimate dry mass of the tagged plant parts (Kuehn et al. 1999).

*Litter bags:* As stated above, the spatial and temporal context in which plant litter decomposes is a crucial consideration when selecting methods to examine plant litter decomposition. Methods chosen ideally do not (or minimally) fundamentally alter the natural sequence or the environmental conditions of decomposition (Bärlocher 1997). Most studies use known amounts of pre-dried, native plant litter that is preferably air-dried with correction to oven dried weight (since air drying generally does not remove all the moisture while oven drying may volatize N). Dried litter is enclosed in litter bags of varying mesh size (e.g., 1–10 mm, Fig. 22d), which allows for size-selective exclusion of macroinvertebrate detrital consumers (Bärlocher 2020). The litter bag technique has been criticized because studies often only measure microbial decomposition and can limit shredding by large detritivores (e.g., crabs), or introduce other experimental artifacts that deviate from the natural decomposition process (Boulton and Boon 1991; Bärlocher 1997).

For each sampling location, multiple litter bags are typically deployed at study initiation, and individual litter bags are subsequently retrieved at pre-determined time intervals over the study period (Fig. 22a). Sample intervals are typically geometric; for example, in a one-year study where four litter bags are installed at initiation, individual litter bags can be retrieved at four intervals (e.g., 1, 3, 6, and 12 months after installation) to follow a model of exponential decay (Hackney and De La Cruz 1980). Other sampling intervals can be used depending on field site location, substrate quality, and the research questions. Each litter bag should have a unique label, which is often a plastic or non-reactive metal disc placed into, or externally attached to, the bags. This ensures that individual variations in initial mass can be correctly accounted for following retrieval. During deployment, the litter bag samples can either be placed on the surface or inserted into the ground, often at multiple depths (Middleton 2020b). Aboveground litter should be placed on the soil surface in an area where that litter/tissue type is likely to be produced and deposited in that system. It is recommended to pin aboveground bags to the soil surface using PVC pins or similar (e.g., straightened plastic-coated paper clips). Belowground bags should be in good contact with surrounding soil. A trowel or sharpshooter shovel can be used to make a vertical slit in the ground and insert the litter bag belowground. Following insertion, the hole is gently pressed together to insure good soil contact. Samples can be tethered to immovable objects (e.g., flag, prop-root, or stake) with string and marked with flagging if necessary. During litter bag retrieval, utmost care should be taken to ensure that decomposing material does not fragment and fall out of bags during collection. Adhering material should be carefully pulled off, but the bags should never be rinsed in water or otherwise agitated. Litter bag samples can be refrigerated for up to 1 week prior to drying, but should otherwise be frozen until analysis.

*Standardized substrates:* While many studies use native plant litter material in litter bags, the use of a standardized C substrate in field incubation studies provides information on decomposition trends that can be compared among studies, but does not provide natural decay rates. For example, decay rates using a standardized substrate measured across a salinity gradient can be used to assess the relative change in decomposition that may occur with sea-level rise, which may differ considerably from decomposition rates of native vegetation communities that also changes along salinity gradients. Thus, standardized substrates are useful for isolating the effects of environmental factors on decay rates from confounding influences of litter or substrate quality and source material (Stagg et al. 2018).

Several types of standardized substrates are commonly used in wetland incubation studies, including cotton strips (Maltby 1988; Penton and Newman 2007), artist canvas (Slocum et al. 2009; Tiegs et al. 2013), wooden popsicle sticks (Baker et al. 2001a, b), or tea bags (Keuskamp et al. 2013). Selection of the standard material will depend on the ecosystem and research questions. For example, wooden disks (Romero et al. 2005) and popsicle sticks (Baker et al. 2001a, b) have been used in forested wetland studies to simulate decomposition of woody roots and debris. In herbaceous wetlands, cotton strips with a high cellulose content have been widely used as a proxy for evaluating decomposition of macrophyte tissue (Mendelssohn et al. 1999). Provisioning of a given substrate from a sole source, such as Shirley fabric cotton, has been promoted to reduce variability among studies (Maltby 1988). However, Shirley Burial cloth, which had well documented composition and properties, and was specifically designed for soil decomposition studies (Harrison et al. 1988), is no longer produced. The recommended replacement, artist canvas, is an appropriate substitute, but should also be assessed for disparity in chemical composition, particularly across different production runs (Slocum et al. 2009).

Some studies have supported cotton strips within vertical frames, thus enabling the measurement of decomposition profiles across the soil–water interface (e.g., Newman et al. 2001). It is also possible to compare rates of decomposition between plant litter and cloth to identify any differences due to length of time of exposure or inherent differences in decomposition among natural and synthetic substrates for a particular ecosystem type (Middleton 2020b).

Tea bags are also used as standardized substrate in global decomposition surveys. Tea bags are advantageous for global surveys because they can be sourced, and the materials are standardized. One of the first publications establishing the Tea Bag Index evaluated two types of tea that differed in plant material structure (Keuskamp et al. 2013). The difference in the tea types allows users to deploy both types of bags and to later calculate decomposition rate and a stabilization factor (quantified as the difference between measured and predicted mass loss).

*Key Covariates and Ancillary Measurements:* Standard measurements of abiotic factors are important for understanding decay rates including flood regime (depth, duration, and frequency), soil moisture, porosity and mineral composition, soil and water redox potential, salinity, and temperature (Sections “Carbon in Wetland Soils” and “Carbon in Wetland Waters”), and biotic factors including vegetation biomass and community composition, plant litter quality (lignin, cellulose, nutrients) (Sections “Carbon in Wetland Vegetation” and “Net Primary Productivity”), and microbial community composition (Section “Wetland Microbiome”). Litter or substrate quality is commonly analyzed for relative lignin, cellulose, C:N, N:P, and other nutrient contents of specific interest to the study.

*Lignin and cellulose:* Lignin and cellulose content can be measured using sequential digestions such as the acid detergent fiber analysis (produces lignin and cellulose residue) followed by the acid detergent lignin analysis (produces lignin residue) (Van Soest and Wine 1968; Gessner 2020b). Lignin phenols, indicative of plant sources, can be quantified directly using cupric oxide oxidation and coupled to compound-specific stable C isotope analyses (Wysocki et al. 2008). Some of the chemicals used for lignin analysis are hazardous, requiring a fume hood.

#### Laboratory Incubations

**Fig. 23:**
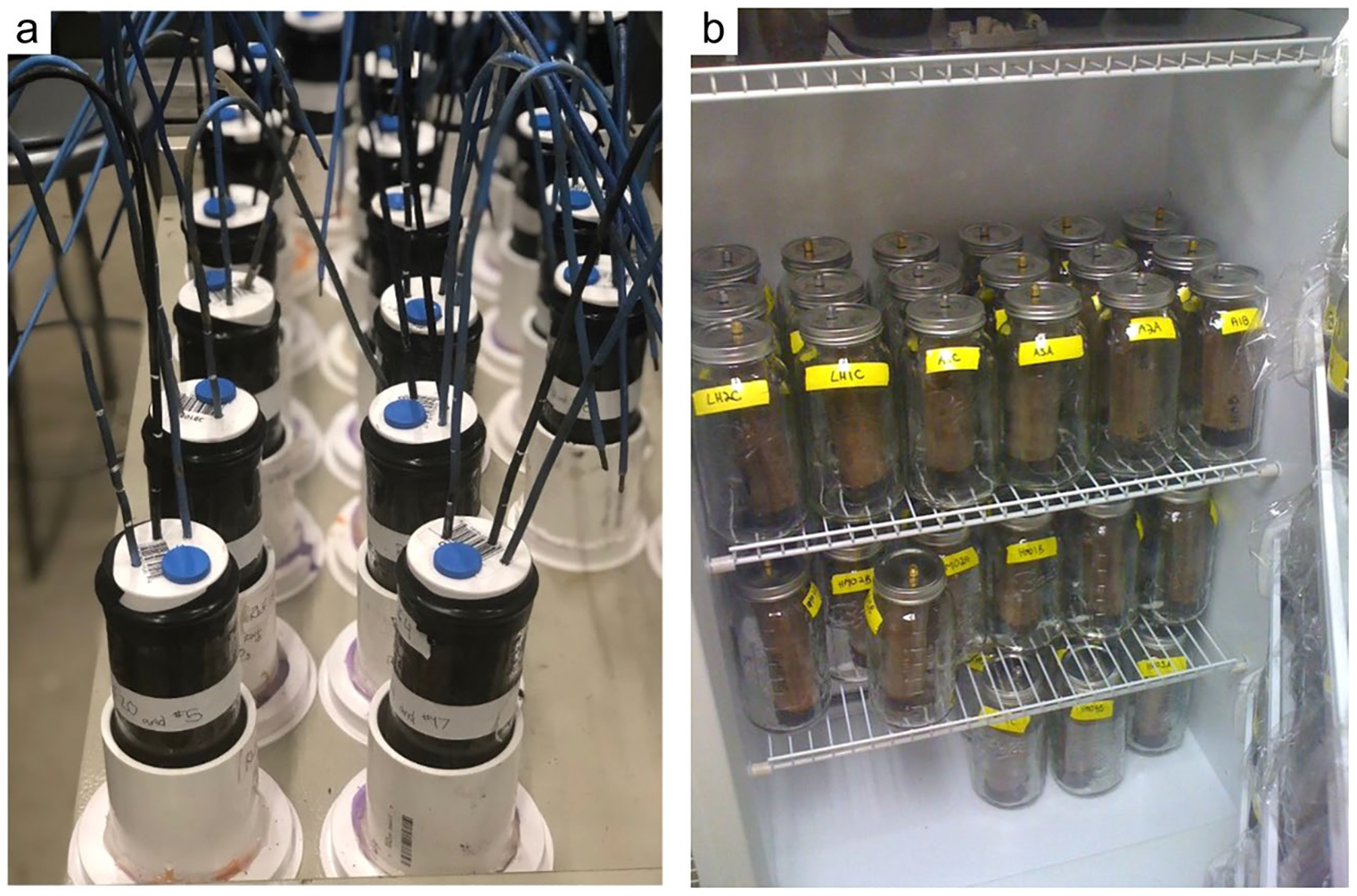
(**a**) Intact soil cores (4-cm diameter, 30-cm deep) collected from the Cowlitz River in Oregon, USA. Soils were incubated in glass containers and capped to maintain anaerobic conditions. Lids were fitted with blue rubber septa for gas collection and three redox probes at different depths to capture vertical spatial gradients; (**b**) sections of cores from a boreal riparian forest incubated within glass (mason) jar incubation vessels. Incubations were capped during the measurement period and kept dark and at constant temperature in an incubator. Gases are sampled with a syringe through a septum Luer Lock fitting in the lid. Images with permission from Stephanie Yarwood (a) and Mark Waldrop (b)

*What:* Laboratory incubations of plant litter or soils assess the influence of biotic and environmental factors on organic matter decomposition potentials under controlled conditions (Schädel et al. 2020). Incubations consist of a specific mass (or volume) of litter or soil contained within a closed- or flow-through container from which headspace gas or liquid discharge can be collected and analyzed for GHG concentrations (Fig. 23) or dissolved C constituents. Sometimes incubations can incorporate exogenous substrates (e.g., ^13^C-labeled glucose) to examine turnover of specific compounds or the priming effect (Guenet et al. 2018). Importantly, rates of organic matter decomposition quantified by incubations can differ from those measured in the field (e.g., Rinkes et al. 2013). Therefore, incubations are best suited to examining relative *potential* rates of decomposition among treatments and how these potential rates may change with environmental perturbations. For example, incubations can provide information on wetland responses to increasing N deposition (Bulseco et al. 2019), which may otherwise be difficult to measure in the field due to a lack of appropriate field sites or logistical issues. Despite the known discrepancies between in situ (field) and potential (incubation) decomposition rates, many variables in process-based models are parameterized using incubation studies. The Soil Incubation Database (SIDb) is a publicly available collection of incubation studies on C and CH_4_ fluxes (Schädel et al. 2020).

*Where:* See Section “Soil Collection” for details on collecting soil samples. Laboratory incubations may use soils collected from multiple cores that can be analyzed separately or combined and homogenized. Incubations that homogenize multiple field samples provide improved spatial representation of a site average, but lose information about within-site variation and the process of homogenization may also lead to artifacts (e.g., disturbed natural soil structure) (Fig. 23b). Intact cores maintain the structure of the soil, but, if an incubation experiment involves additions of exogenous substrates, it can be difficult to uniformly disperse the substrate into soil due to variable constraints on diffusion or physical transport. If soils are collected in saturated conditions, care should be taken to maintain anoxic conditions to limit rapid oxidation of elements such as Fe. Incubations can be conducted in test tubes, flasks, mason jars, or larger containers. The choice of container will depend on several factors including: the volume of sample for incubation, the planned measurements, permeability of the container and closure/seals, and interaction of the sample with container material. Incubation vessels should be free of metals, salts, and organic materials by washing glassware with phosphate-free soap, acid rinsing, and then baking (glassware) at high temperatures (e.g., 500 °C to remove organics). Autoclaving alone sterilizes vessels but may not remove all organics.

*When:* Soils for laboratory incubations can be collected year-round (conditions permitting) or more frequently to examine seasonal dynamics. Some studies target specific time points in the growing season such as during peak plant biomass. Following sample collection, the incubations themselves are established and monitored for anywhere between a few days up to a few years. The length of incubation is determined by the ability to maintain incubation conditions without the build-up of waste products or depletion of substrates, which can lead to experimental artifacts.

*Who:* Incubations generally require more highly trained personnel to set up and conduct studies, particularly if multiple constituents or end products are being measured. Laboratories are often equipped with specialized instruments to measure end products such as produced/consumed GHGs, soil and dissolved C constituents, and soil and water chemistry.

*How:* To assess decomposition rates (e.g., mg C released g^−1^ soil [or g^−1^ C] hr^−1^), incubation containers are sealed and held at set temperature(s) in the dark, during which periodic collection of GHGs can be used (including the initial, pre-treatment condition) to calculate GHG production (in moles) over time. For accurate quantification of GHG production under saturated conditions, dissolved GHGs are ideally measured as well (Section “Carbon in Wetland Waters”).

Incubations to quantify anaerobic decomposition should be handled appropriately to ensure an atmosphere with little to no O_2_ (e.g., 0–5 ppm), which can be challenging due to high atmospheric concentrations. Best practices include conducting and sampling incubations within O_2_-free chambers (i.e., glove bags) or air-tight containers while monitoring O_2_ concentrations (if feasible) in sampled headspace (Fig. 23a). Flushing incubation chambers with ultrahigh purity N_2_ or zero air (i.e., gas devoid of hydrocarbons and CO_2_) is often conducted as well to establish anoxic conditions. Any additions made to the incubation (e.g., water) should contain little to no O_2_. In contrast, incubations to quantify aerobic decomposition potential require sufficient atmospheric O_2_ and can be maintained by providing a large headspace volume in sealed containers, by periodically adding zero (CO_2_-free) air, or by sealing incubation containers for specific time increments (e.g., a few hours or days) instead of sealing for entirety of the incubation.

*Key Covariates and Ancillary Measurements:* A multitude of abiotic and biotic factors that influence anerobic or aerobic organic matter decomposition are ideally monitored during incubations, including: water content, temperature, O_2_ and nutrient concentrations, terminal electron acceptors, redox potential, microbial community size or composition, light, salinity, bulk density, and SOC content (Sections “Carbon in Wetland Soils”, “Carbon in Wetland Waters”, and “Greenhouse Gas Fluxes”). At a minimum, water content and temperature are most important to measure, followed by SOC content. See Section “Chamber Measurements” for descriptions of CH_4_ production and oxidation incubations.

*Chemical composition of plant litter and soil organic matter:* The molecular structure (or chemical quality) of plant litter and SOM can affect heterotrophic microbial activity and thus provides a mechanistic context to understand variability in decomposition rates. Common compounds of interest in wetlands include phenolics, tannins, and ligno-cellulose (complex of lignin and cellulose). Stoichiometric ratios (e.g., CHN:P; lignin:N) are broadly correlated with rates of litter decomposition (Melillo et al. 1982; Berg and McClaugherty 2020) and are important metrics to measure. The chemical composition of litter and SOM can be assessed with a variety of methods–each with its own advantages, limitations, context, and ease of application. There are traditional methods (e.g., gas chromatography [Section “Chamber Measurements”], liquid chromatography, spectroscopic, thermal methods) and newer methods (e.g., FTICRMS, near-edge X-ray absorption fine structure; Lehmann and Solomon 2010; Heister et al. 2012; Tfaily et al. 2015) that are beyond the scope of this review; see method reviews by Kögel-Knabner (2000), Sparks et al. (1996), and Cook and Bianchi (2013) for more detailed information. Generally, these methods rely on broad chemical characterization (e.g., elemental stoichiometry), direct quantification of specific compound classes (e.g., extraction and quantification of a biomarker like lignin), or assessment of organic matter functional groups (e.g., alkyl-C with NMR spectroscopy) or thermal stability (e.g., TGA). Selection of the appropriate method for organic matter characterization will therefore depend on whether a holistic (spectroscopy, elemental stoichiometry) or targeted (biomarkers) approach is desired, or whether specialized (NMR spectroscopy) or conventional (wet chemistry) equipment is available.

*Stable isotope additions:* The addition of exogenous organic substrates that are enriched in heavier stable isotopes such as ^13^C or ^15^N can be added to an incubation to trace its turnover and fate (e.g., as a GHG, remaining in soil, transformation into specific biopolymers, incorporation into microbial biomass). This experimental design is typically used to understand mechanisms and rates of microbial decomposition of specific organic substrates (Creamer et al. 2015). This approach requires quantification of the stable isotope ratios of C (or other elements such as N, P, or S) in the target organic matter pools or compounds (Sections “Carbon in Wetland Soils”, “Carbon in Wetland Waters”, and “Greenhouse Gas Fluxes”).

*Photodegradation:* In addition to biodegradation of plant litter through microbial processes, a portion of C in litter is lost as CO, CO_2_, or CH_4_ through photodegradation (also referred to as ‘photomineralizatoin’ or ‘photomethanization’ for CH_4_) from sunlight. The relative contribution of photodegradation versus biodegradation on C emissions or the composition difference of litter-derived DOM can be accessed by conducting laboratory-based incubations under lighted and darkened conditions (Song et al. 2020a), by sterilizing litter by autoclaving to eliminate microbial activity, or by using multiple light sources with varying spectra such as ultraviolet and visible light (Song and Jiang 2020).

### Wetland Microbiome

#### Definitions and Units

*Definitions:* Microbial communities (e.g., mesofauna, phytoplankton, fungi, bacteria, archaea, viruses) mediate a number of C pools and fluxes, including the decomposition and transformation of complex C compounds found in plant litter and SOM into smaller molecular weight compounds that can accumulate in wetland soils or leave wetlands via gas flux of CO_2_ and CH_4_ or laterally as DOC or POC. Changes in the abundance and activity of these microbial communities can result in direct (and often outsized) changes to fundamental biogeochemical processes. For example, changes in fungal to bacterial ratios (Talbot et al. 2015; Soares and Rousk 2019), mesofauna abundance (Waldrop et al. 2012), or the abundance of methanogens and methanotrophs (Vizza et al. 2017) can all simultaneously and interactively influence decomposition rates and CH_4_ flux. Here we present a very brief overview on methods to measure microbial biomass, productivity, metabolic activity, and community composition, which can provide mechanistic information on variability in C pools and fluxes in space and time.

*Units:* Microbial biomass associated with plant litter or soil is typically measured and reported as the quantity of biomass per gram of litter or soil. Likewise, rates of microbial production are measures as μg or mg of biomass produced per gram of litter or soil per unit time such as hour or day. Enzyme activities are typically reported as μmol of C decomposed or transformed per gram of litter or soil (or per gram of C) per hour. In some studies, rates of decomposition, CO_2_ flux, or enzyme activity are normalized to the amount of microbial biomass in order to better understand C use efficiency and to make normalized comparisons across experimental treatments or studies.

**Rationale:** Collectively, measurements of bacterial, archaeal, and fungal communities are increasingly applied within a variety of aquatic habitats and found to be useful in assessing the role and importance of these microbes in wetland C cycling and in quantifying the microbial food base that is available to wetland detritivores (Buesing and Gessner 2006; Gulis et al. 2006, 2019; Gessner et al. 2007; Kuehn et al. 2014; Kuehn 2016). Rates of microbial production and transformation of C are often used to parameterize process-based models (e.g., E^3^SM [https://e3sm.org/)]), which are then used to predict C dynamics at larger spatial scales.

Where and When:

*Sample preservation:* Microbial biomass and community composition can be measured from litter, soil, and water. Because a freeze/thaw cycle can rupture (or lyse) microbial cells, assays for microbial growth (e.g., stable isotope probing) are ideally done on freshly collected material. However, logistical considerations often require preservation of samples (Zizka et al. 2022). Preservation of environmental samples for molecular analysis lacks a consistent methodology across laboratories, yet it can be critically important for scientific studies (Rubin et al. 2013). The method of preservation for samples to be used for molecular analysis depends on the comparisons being made, the analysis to be done, the type of sample collected, and the amount of time the sample is to be preserved (Zizka et al. 2022). Ideally, samples are frozen as soon as possible after collection. Storage in − 80 °C freezers or − 190 °C liquid N_2_ storage tanks can preserve samples for many years (Pasternak et al. 2019). However, there may be situations when samples can be maintained in a − 20 °C freezer for DNA analysis for up to a decade (Zizka et al. 2022), or even refrigerated at 4 °C or at room temperature for up to two weeks, but this has only been demonstrated for aerobic surface soils (Lauber et al. 2010). RNA is less stable than DNA, therefore samples are ideally flash frozen (e.g., liquid N_2_) in the field at the time of sampling. If comparisons are made among broad regions, storage conditions may not be as critical to beta diversity, although alpha diversity can be affected (Lauber et al. 2010). Freeze drying is also a useful technique to maintain cell, enzyme, and DNA integrity of the sample (Clasen et al. 2020). However, for long-term storage, freeze dried soils should be kept frozen (Pasternak et al. 2019). Thawing and refreezing of samples should generally be avoided because it can cause significant decomposition (spoilage) (Clasen et al. 2020). Preservation solutions can also be added to soils such as denatured alcohol, but these could result in changes in beta diversity (Clasen et al. 2020; Zizka et al. 2022). Overall, both short-term and long-term storage of soil samples is best at − 80 °C (Pasternak et al. 2019), although it is just as critical to maintain consistent sampling, extraction, and processing steps.

*Who:* Some assays, such as microbial biomass, are relatively non-technical and straightforward to conduct. Other assays of composition and activity incorporating microbial DNA can range from medium complexity (qPCR or 16S RNA) to high complexity (metagenomics or stable isotope probing) analysis. Nucleic acid approaches are numerous, but all of them require specialized equipment. Fortunately, many commercial and university laboratories offer DNA extraction, amplification, and sequencing services, making these methods increasingly available, albeit sometimes costly.

*How:* There are many methods to measure microbial pools and activity that are beyond the scope of this review. Here we provide a basic overview of the various options of methods and references for additional information.

#### Total Microbial Biomass and Activity

Chloroform fumigation extraction has been used for many decades to assess total microbial biomass in wetland and other soil types (Vance et al. 1987). Three subsamples are required: one sample to measure soil moisture content, one sample fumigated with chloroform vapors (to kill and lyse cells) in a desiccator for 24 to 48 h, and a third as an unfumigated control. Both the chloroform fumigated and an unfumigated sample are extracted with 0.5 M potassium sulfate (K_2_SO_4_) or other salt. The resulting filtrate is analyzed for total C, and microbial biomass is determined as the difference between unfumigated and fumigated soils. A correction factor is often (but not always) applied to account for the incomplete efficiency of the method (Joergensen 1996). Measurements of total biomass are often paired with some measure of microbial activity. For example, substrate-induced respiration (SIR) can be used to determine the potential microbial activity for a given soil. For SIR, substrates such as glucose, plant residues, or fermentation products are added to a soil slurry to stimulate microbial activity. The soil is then incubated at a constant temperature and the headspace gas is periodically measured, with the assumption that the rate of gas emissions following substrate addition is proportional to microbial activity. For wetland soils, both CO_2_ and CH_4_ are measured in the headspace gas (Wright and Reddy 2007). SIR can be combined with selective inhibitors on bacteria and fungi. For example, antibiotics that target protein synthesis in fungi or bacteria can be added along with C substrates, resulting in a fungal to bacterial activity ratio (Bailey et al. 2003).

#### Bacterial and Archaeal Biomass, Growth, Production

Bacteria and archaea play an important role in organic matter decomposition and CH_4_ production and consumption in wetlands (Yarwood 2018). Hence, several techniques have been developed to estimate their abundance, biomass, and production associated with decaying organic matter in wetlands and other aquatic ecosystems. A commonly used approach to assess abundance and biomass is direct count microscopy (Buesing and Gessner 2002), where cells associated with decomposing litter or sediments are detached from the substrate and the resulting cell suspension is filtered, stained with a fluorescent dye (e.g., SYBR Green II), and counted using an epifluorescence microscope (Noble and Fuhrman 1998; Buesing and Gessner 2020, Gessner 2020b). Cells are then assigned to size classes or photographed and analyzed with an image analysis system to calculate biovolumes and biomass (Buesing and Gessner 2020). Alternatively, microbial abundance and biomass can be estimated via flow cytometry (Wang et al. 2010; Frossard et al. 2016), using commercially available kits.

Rates of bacterial production can be obtained by measuring the incorporation of radiolabeled precursor molecules into bacterial macromolecules, such as protein or DNA (Buesing et al. 2020). The incorporation of [^3^H]thymidine (Findlay et al. 1984) and [^3^H]leucine (Buesing and Gessner 2003; Gillies et al. 2006) are the two most commonly used methods. The incorporation and synthesis rate of macromolecules (e.g., [^3^H]leucine incorporation into bacterial protein) is considered to be directly proportional to the production (P_bacteria_) of bacterial biomass when using empirical or theoretical derived conversion factors (Buesing and Marxsen 2005). If estimates of bacterial biomass (B_bacteria_) are known, then growth rates (μ_bacteria_) can be determined by calculating the P_bacteria_ to B_bacteria_ ratio and converting these ratios to a growth rate using the following formula (μ_bacteria_ = ln[1 + P_bacteria_/B_bacteria_]) (Buesing et al. 2020). Polymerase Chain Reactions (PCR) based techniques can also be used to quantify bacterial groups (see below).

#### Fungal Biomass, Growth, and Production

Fungi are also a key decomposer community in wetlands (Gessner et al. 2007; Kuehn 2016). Historically, determining fungal biomass in decaying organic matter, such as plant litter, has been challenging because fungal hyphae grow within decomposing plant tissues and are not easily separated from plant litter using optical or mechanical methods (Gessner and Newell 2002). However, evidence has emerged on the usefulness of ^14^C-acetate incorporation into fungal ergosterol in the quantification of fungal biomass and fungal growth rates within decaying organic matter (Gessner and Newell 2002; Gessner 2020a; Suberkropp et al. 2020). Fungal growth rates (μ_fungi_) are directly proportional to acetate incorporation rates and can be calculated using either empirical or theoretical conversion factors (Gessner and Newell 2002). Growth rates can then be multiplied by fungal biomass (B_fungi_) to obtain rates of fungal production (P_fungi_): μ_fungi_ × B_fungi_ = P_fungi_.

#### Microbial Community Phospholipid Fatty Acid Analysis

**Fig. 24:**
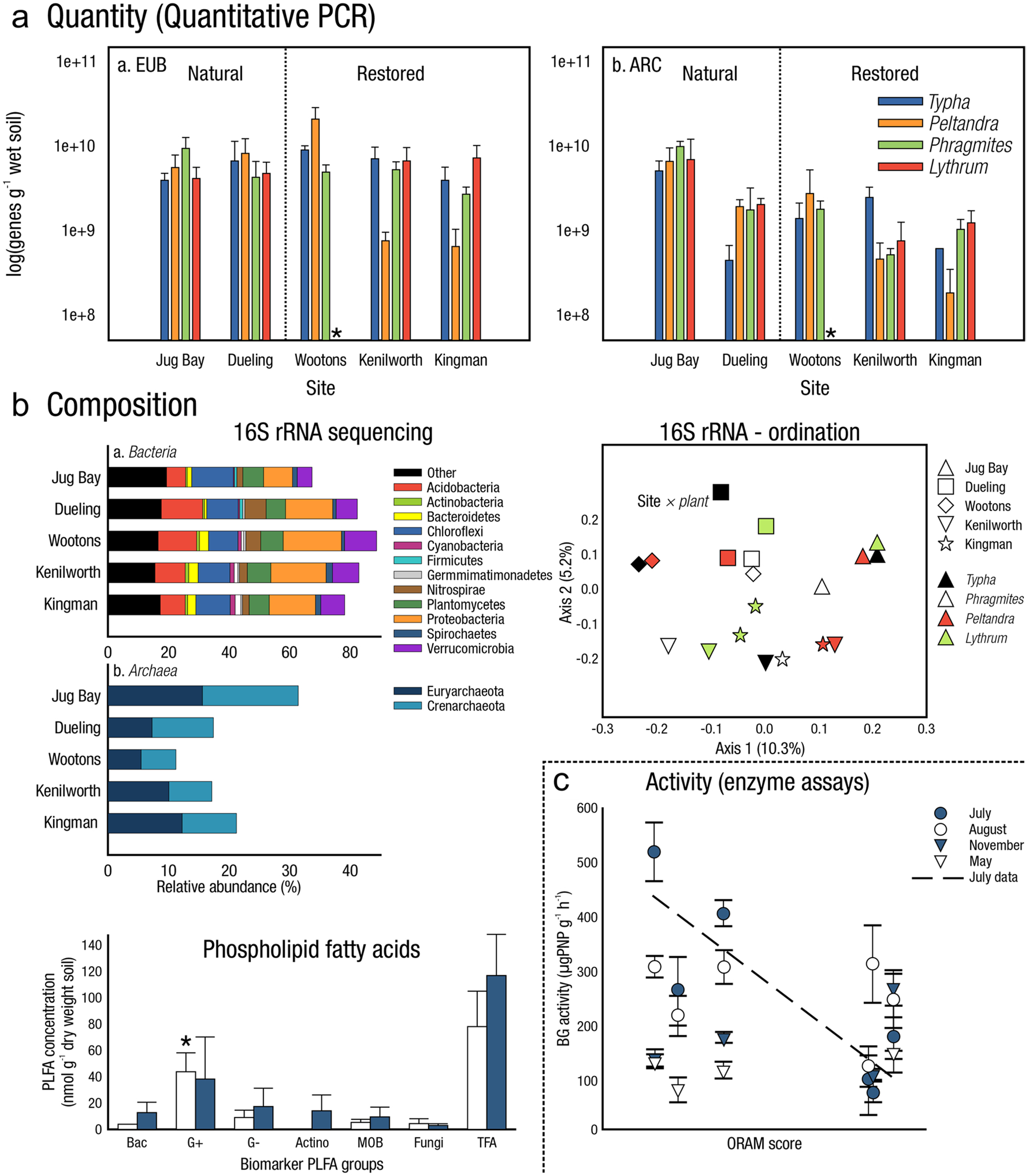
Examples of data to assess the (**a**) quantity, (**b**) composition, and (**c**) activity of the wetland microbial community. (**a**) Quantification of bacterial (EUB, left) and archaeal (ARC, right) 16S rRNA gene copies in natural and restored wetlands using quantitative polymerase chain reaction (PCR) (modified from Prasse et al. 2015); (**b**) microbial community composition measured using 16S rRNA gene sequencing (Prasse et al. 2015) (top left), a principal component analysis ordination based on 16S rRNA bacterial and archaeal amplicon sequencing (% variance explained) (modified from Prasse et al. 2015) (top right), and concentrations of phospholipid fatty acid (PFLA) biomarkers (Bac = total bacteria, G + = Gram positive bacteria, G- = Gram negative bacteria, Actino = actinobacteria, MOB = methane oxidizing bacteria, TFA = total fatty acids) (modified from Chowdhury and Dick 2012) (bottom left); (**c**) activities of Beta-glucosidase (BG, an enzyme for breaking down complex polymers such as cellulose) and Ohio Rapid Assessment Method (ORAM) scores in a forested wetland (modified from Rokosch et al. 2009)

A number of methods assess the composition of microbial communities, including phospholipid fatty acid (PLFA) extraction and analysis (Fig. 24). Phospholipid fatty acids are found in all cell membranes and can be extracted from soil slurries using chloroform, methanol, and PO_4_^3−^ buffers (White and Ringelberg 1998). The PLFA method requires separation of phospholipids using solid phase extraction columns and a series of drying and concentrating steps to achieve a purified sample, which can then be quantified by gas chromatography following esterification. A modification of the procedure allows 96 samples to be extracted simultaneously (Buyer and Sasser 2012). The resulting fatty acid profile can be further categorized into biomarkers characteristic of fungi, total bacteria, Gram positive bacteria, and Gram negative bacteria. One limitation of the PLFA approach is that the standard method does not capture archaeal lipids because they are ether linked. Analysis of archaeal lipids is possible using a alternate methods (Gattinger et al. 2003), but requires greater analytical work. PLFA data can be analyzed using multivariate statistics to compare community composition or summed to assess microbial biomass. Often, peaks for total fungi and total bacteria are used to determine a fungal to bacterial ratio. The PLFA method can be combined with stable isotope analysis to determine which groups of microorganisms are consuming added substrates. For example, Balasooriya et al. (2013) added ^13^C-labeled rhizodeposits into wetland soil and was able to identify the specific microbial groups that use the rhizodeposits to support growth and activity.

#### Molecular Microbial Community Analysis

A number of methods can characterize microbial communities based on nucleic acids (DNA/RNA) and proteins. The most common method is the extraction of DNA from soil or litter samples and amplification of target genes using PCR (also called ‘amplicon sequencing’). The choice of target gene (phylogenetic or functional) determines which groups are characterized. For example, 16S ribosomal ribonucleic acid (16S rRNA) genes are used to characterize the taxonomic composition of bacteria and archaea (Fig. 24b) (Prasse et al. 2013); the intergenic spacer region of fungal rRNA can be used to assess fungal composition (Gonzalez Mateu et al. 2020). In some studies, specific microbial processes, such as methanogenesis or CH_4_ oxidation, can be targeted by amplifying a known functional gene (e.g., the mcrA gene that codes for CH_4_ coenzyme A or the pmoA gene for particulate CH_4_ monooxygenase), although the presence of genes does not necessarily indicate function (expression) as many genes can be dormant. Regardless of the gene targeted, resulting data are analyzed with bioinformatic pipelines such as Qiime2 (qiime2.org) and characterized using public databases (e.g., Green Genes, UNITE) that match sequence data to known microbial taxa or functional genes. Multivariate statistics are often applied to compare microbial community composition. Advances in sequencing technology have made high throughput sequencing inexpensive and more readily accessible on common platforms (e.g., Illumina).

‘Shotgun metagenomic sequencing’ is another sequencing technique. Shotgun sequencing requires extracting and sequencing all DNA and/or RNA from soil (as opposed to targeted genes with PCR; Abraham et al. 2020). Data from DNA shotgun sequencing can be used to characterize the composition and functional potential of the entire microbial community. Data from RNA shotgun sequencing provides an even more detailed analyses of microbial function, as the messenger RNA reflects expression of specific functional genes or metabolic processes (i.e., metatranscriptomics; Hultman et al. 2015). RNA seq, as it is sometimes referred, can indicate active metabolic processes at the time of sampling (Angle et al. 2017).

The extracted DNA or RNA can also be used to quantify microbial groups using a quantitative PCR (qPCR) approach that quantifies the total abundance of genes rather than relative abundance (Fig. 24a). Like standard PCR, a gene of interest is selected and targeted for amplification. During the amplification process, fluorescent dyes are used to track the number of DNA copies via optic readings. Back calculations of both standards and samples can then determine the original number of that gene in the soil (Prasse et al. 2013; Angle et al. 2017). qPCR can be used to compare biomass of microbial groups and to help standardize sequencing data. Many molecular techniques such as stable isotope probing, Q-stable isotope probing, microarrays, CHIP-stable isotope probing, and others, can provide important information regarding C cycling processes, but are beyond the scope of this review (Emerson et al. 2017). It should also be noted that viral activity targeting methanogens or other microbial taxa can shape microbial communities and turnover rates, although most viral populations in wetlands are unclassified at any taxonomic level (Dalcin Martins et al. 2018).

#### Soil and Litter Enzyme Activities

Extracellular hydrolytic and oxidative enzymes are involved in the breakdown of macromolecular compounds in plant litter and soils. Enzymes exist in cells, on cell membranes, on cell remnants, in biofilms, on soil particles, and in solution. Enzyme activity is strongly affected by the microhabitat (temperature, diffusion, pH, water films), which can affect substrate availability and enzymatic efficiency (Fig. 24c). Thus, enzyme activity measured in the laboratory is considered a *potential* process, reflective of the total quantity of enzyme under optimum conditions. C degrading enzymes include those that breakdown cellulose, chitin, and lignin, while other nutrient releasing enzymes release P and N from organically bound compounds. After enzymatic breakdown of complex compounds, release of smaller molecular weight C-, N-, and P-containing compounds can be incorporated into microbial cells (Burns et al. 2013). Most extracellular enzymes are produced by microorganisms such as bacteria and fungi, although some enzymes can also be released by plant roots within the soil. Rates of enzyme activity have been used to understand, estimate, and model potential rates of decomposition of different C compounds and nutrient releasing microbial activities in litter and soils (Schimel and Schaeffer 2012; Waldrop et al. 2012). Ratios of C degrading enzymes to nutrient releasing enzymes can be used as an assay of relative nutrient limitation by the soil microbial community (Sinsabaugh et al. 2008; German et al. 2011). Microbial community composition and the decomposition processes are functionally linked when combined with metagenomic and functional genomic techniques, which detect and quantify the organisms and functional genes responsible for producing important enzymes (Arnosti et al. 2014).

Assays of enzyme activity are relatively straightforward, inexpensive, and quick (i.e., minutes to hours) to conduct. Minimum requirements include a colorimeter, substrates, and basic laboratory materials such as pipettors and buffers. Assays are typically performed on freshly sampled litter or soils (within several days of collection); enzymes degrade rapidly as the litter or soil is disturbed and microbes alter their activity. Freeze drying is a useful way of preserving soil or litter prior to assays, as it maintains enzyme integrity and makes it easier to weigh small quantities of dry mass. Assays can be conducted either colorimetrically (using p-nitrophenol or L-DOPA substrates, Sinsabaugh et al. 2008), or fluorometrically using methyl umbelliferyl linked substrates (German et al. 2011). Methyl umbelliferyl linked substrate assays have lower detection limits and are easier to control for soil interference (such as quenching). To avoid interference from soil in colorimetric assays, final colorimetric measurements can be done on supernatant material. For methyl umbelliferyl linked substrate assays, soil particles may remain in solution, but a separate quenching control should be conducted for each sample (German et al. 2011). Both colorimetric and fluorometric assays can be run as high throughput assays on 96 well plates (Burns et al. 2013; Jackson et al. 2013). Care should be taken to make sure that soils are well homogenized because assays are done on very small aliquots of materials. Many analytical replicates (often 8, or one column on a microplate) per sample are recommended.

### Lateral Flux

#### Definitions and Units

*Definitions:* C can enter or leave a wetland as a particulate or solute if there is physical movement of water between a wetland and its surrounding water-shed, referred to as ‘lateral flux’. Lateral flux of C particles can also occur via aeolian transport, or anthropogenically through manual removal of C pools, such as tree or peat harvesting or sediment dredging (Roulet 2000; McKee et al. 2012; Kolka et al. 2022). Here we primarily focus on lateral fluxes of C associated with water movement (i.e., hydrological fluxes). When quantifying lateral C fluxes, the two main components to measure are: 1) hydrological fluxes; and 2) concentrations of C constituents in water. Methods to collect and measure dissolved GHGs, DIC, DOC, and POC, as well as covariates such as temperature, depth, pH, salinity, etc., in discrete water samples were described in Section “Carbon in Wetland Waters”.

The main types of lateral hydrological flux are surface-water flow and groundwater flow, both into and out from wetlands. Overland flow of water moving downhill from surrounding uplands (i.e., runoff) can also be an important hydrological flux to wetlands. Lateral transport of wetland C can also occur within wetlands, such as during periods of sediment mobilization and recirculation following storm events. Precipitation and evapotranspiration are important vertical hydrological fluxes in wetlands that influence lateral fluxes. In tidal wetlands, quantifying lateral C fluxes is complicated by bidirectional tidal exchange of water between the wetland and estuary or coastal ocean (Wang et al. 2016). Subsurface flow of water to a wetland that occurs above the water table, sometimes called ‘interflow’, also can occur, although its contribution to lateral C flux usually is relatively small. Disturbances such as major storms can trigger ‘event flows’ that laterally transport orders of magnitude more C than all other hydrological fluxes combined (Dalzell et al. 2005; Eimers et al. 2008). Event flows are often overlooked because most studies use periodic grab sample collections, rather than event-based sampling or extended time-series data, potentially biasing results (Chu et al. 2018; Bogard et al. 2020a). Thus, even where lateral fluxes are measured, they have high uncertainty. The challenges with accounting for multiple, interacting hydrological fluxes and dynamically changing C constituents and their concentrations in water makes lateral C fluxes one of the least understood and most poorly quantified components of wetland C budgets (Fig. 2a).

Even though we separate lateral fluxes into three distinct vectors (surface, ground, overland flows) and their associated methods, distinctions between the sources of water tend to blur in wetlands. For instance, in tidal wetlands, it is logical to assume that surface-water flows in response to tides are both the source and vector of lateral C flux. However, the C carried by those tidal flows may be sourced from the wetland, upland, and/or estuary. The C from the upland might enter the wetland and tidal channel from overland runoff or from groundwater discharge. Groundwater can mix with wetland porewater and surface water, and therefore the groundwater will carry C from both upland and wetland sources (Bogard et al. 2020a). Thus, investigations of lateral flux need to be extremely clear regarding methodologies, scale of inference, and assumptions regarding sources, sinks, and fate of C to and from wetlands. In some studies, it may be more realistic to estimate lateral flux through a mass balance approach, in which lateral flux is estimated as the remainder of vertical C inputs minus vertical exports plus C burial (Forbrich et al. 2018; Krauss et al. 2018b), as demonstrated in Bogard et al. (2020a).

The fate of laterally exported C is another consideration regarding wetlands as C sources or sinks, particularly in coastal marine systems that exchange water with the ocean (Chu et al. 2018; Santos et al. 2021). Specifically, exported dissolved CO_2_ could evade relatively quickly to the atmosphere (e.g., degassing; Serikova et al. 2018; Turner et al. 2023), while net alkalinity export of DIC (mostly as HCO_3_^−^), DOC or POC may be stored in ocean waters or sediment for extended periods of time (Van Dam et al. 2021). Long-term storage of exported inorganic or organic C to receiving ocean or other waters remains an unresolved question regarding the role of wetlands in the global C cycle.

*Units:* The units used to describe concentrations of C constituents in discrete water samples are described in Section “Carbon in Wetland Waters”. Hydrological fluxes are characterized by lateral flow rates of water that are typically measured in units of volume per unit time (e.g., per hour, per 5 or 15 min). Fluxes of C to or from wetlands are determined by multiplying C concentrations, in terms of mass per volume (e.g., g C L^−1^), by lateral flows (e.g., L hr^−1^) to obtain C fluxes in mass per unit time (e.g., g C hr^−1^). C flux rates can be normalized to an area-based flux rate (e.g., g C hr^−1^ m^−2^) and upscale in space and time (e.g., kg C yr^−1^ ha^−1^). Groundwater flow requires measurements of hydraulic gradient (absolute difference in hydraulic head divided by distance [e.g., m m^−1^]) and hydraulic conductivity (distance that water flows per unit time [e.g., m yr^−1^]).

**Rationale:** Collectively, lateral fluxes of POC, DOC, DIC, and dissolved GHGs can be some of the largest components of a wetland’s net ecosystem C balance (NECB) in both inland and especially tidal wetlands (Herrmann et al. 2015; Wang et al. 2016; Li et al. 2018a; Najjar et al. 2018; Webb et al. 2019; Bogard et al. 2020a; Cabral et al. 2021). If there is net export or import of C by lateral fluxes, attempts to close the C balance without quantifying lateral fluxes may result in inaccurate estimates of NECB in both size and sign. For example, in a peatland in Scotland, the average NEE was measured to be 278 kg C ha^−1^ yr^−1^ absorbed from the atmosphere, indicating net C uptake; however, 304 kg C ha^−1^ yr^−1^ was laterally exported over the same period, implying the peatland experienced a net C loss during the study (Billett et al. 2004). Similarly, in another example, a single rain event in an Australian savanna accounted for nearly 40% of annual lateral C export and shifted the landscape from a net C sink to a source for several days (Duvert et al. 2020).

The dynamic, and often ephemeral, nature of lateral C fluxes challenges measurements and modeling approaches (Zhou et al. 2023). Increasing the number of observational studies on lateral flux is crucial for the discipline to help refine and constrain regional, national, and global C budgets (Hayes et al. 2018). Moreover, in countries such as the United States, wetland protection is based on wetland connectivity to larger, more permanent water bodies or rivers (Wade et al. 2022), which has particular relevance to depressional wetlands that are often labeled as geographically isolated wetlands that are not permanently connected to other water bodies and therefore not always protected (Mushet et al. 2015a; Rains et al. 2016). A recent United States Supreme Court decision (circa May 2023, ‘*Sackett v. Environmental Protection Agency*’) restricted protection of wetlands to only those that maintain continuous surface connection to larger, navigable Waters of the United States (WOTUS), thereby losing protection of millions of acres of wetlands that have seasonal or belowground hydrology (Jaffe 2023).

#### Surface-Water Inputs and Exports from Rivers, Streams, Tides

*What:* For many wetlands, dissolved and particulate C in surface water (i.e., water above the sediment surface) enters and leaves in channelized flows of water via streams, rivers, or point effluent sources. Accurate estimates of surface-water flow are critical to constraining lateral C flux rates (Wang et al. 2016; Campeau et al. 2017; Bogard et al. 2020a). For well-established stream channels, quantifying hydrological exchange can be relatively straightforward (e.g., Zhu et al. 2019a). However, in many wetland settings, surface-water flow occurs, at least in part, via poorly established, diffuse channels, through which water can flow at variable rates and sometimes intermittently (Shaw et al. 2012).

Estimating hydrological fluxes in tidal systems is particularly challenging because channelized surface water can flow in either direction (e.g., with both headwater flooding and backflooding), which induces sheet flow in multiple directions (Wang et al. 2016). Therefore, water can enter or drain from the wetland through alternate pathways independent of a main channel (i.e., smaller channels and overland exchanges) (Bergamaschi et al. 2012a). In tidal systems, relative elevation is a key driver of water exchange and can therefore be used to model water (and C) movement and the extent of the contributing catchment area to surface flows (Bergamaschi et al. 2012b; Wang et al. 2016; Bogard et al. 2020a).

*Where:* Surface-water flow into a wetland commonly occurs via discrete channels from the upland terrain. In some cases, the input of wastewater effluent can represent the dominant surface-water input pathway and the main component of the water budget (Zhu et al. 2019a; Zhou et al. 2023). However, when flowing surface water enters the relatively flat terrain of wetlands, the flows become broader and poorly defined (Bergamaschi et al. 2012a; Shaw et al. 2012), and can be further concealed by dense emergent vegetation. For flow-through wetlands, the location of minimal elevation (also called spill point) is the location where surface water flows away from the wetland if wetland stage (water level) is higher than the elevation of the wetland spill point (Shaw et al. 2012). Understanding flow and cycling of C from the source of water to the spill point is important to help identify sampling points along the water flow paths.

In the case of coastal or estuarine wetlands with broad sheet-flow, the location of inflow and outflow often cannot be identified as a singular point but instead is viewed as a plane across which water is flowing (and thus where velocities and concentrations are measured). During an individual tidal cycle, the relative importance of broad, non-channelized flows to total discharge may be greatest during the highest tides (i.e., spring tides during a full or new moon; Bergamaschi et al. 2012a). While such exchanges lead to imbalances in water budgets constructed from monitoring of tidal exchanges within larger channels, these imbalances are often relatively small (< 10% of the net volume of exchange) and can be accounted for in C budgets (e.g., Bergamaschi et al. 2012a; Bogard et al. 2020a).

*When:* The flow regime of surface waters should be considered when developing a sampling timeline, to ensure that variations in surface flows are captured. Surface-water flows can be continuous, intermittent, or ephemeral, depending on the location and stability of the source of flow entering the wetland. In the case of surface-water flow out from the wetland, the timing and duration depends on the stage of the wetland relative to the spill-point elevation. Event-based, ephemeral flows, such as following storms, can laterally transport large amounts of wetland C (Duvert et al. 2020). Over longer (decadal) timescales, annual rates of lateral C export can change dramatically with shifting climatic conditions, such that export during wet conditions can be an order of magnitude greater than during extended drought (Zhou et al. 2023).

In tidal systems, the timing of high and low tides must be accounted for in study design and the timing of sampling. Tidal exchanges vary as a function of the lunar cycle, such that on a monthly cycle, the tidal range (total change in surface-water elevation between the high and low tide) is greatest during spring tides and reduced during neap tides (e.g., Bergamaschi et al. 2012a). At annual timescales, surface-water elevation and therefore tidal flooding extent also depends on other factors including discharge rates from nearby rivers that can increase water levels during high flows, and anthropogenic flow management (Bergamaschi et al. 2012a; Bogard et al. 2020a). Given the complex and dynamic controls of tidal wetland hydrology, sampling is often undertaken at 15-min to hourly intervals to ensure the lateral hydrological and C fluxes are adequately characterized, often requiring in situ sensors (Section “In situ Sensors and Analyzers”; Maher et al. 2013a; Chu et al. 2015; Wang et al. 2016; Taillardat et al. 2018; Bogard et al. 2020a).

*Who:* For well-established channels, measurements of streamflow are relatively straightforward for trained researchers (Rantz 1982). However, if flow rates are slow and channels obscure (as occurs frequently in wetlands), greater expertise and background knowledge of local wetland and watershed hydrology are needed to establish sampling locations and frequency to quantify surface-water flow (Variano et al. 2009).

**Fig. 25:**
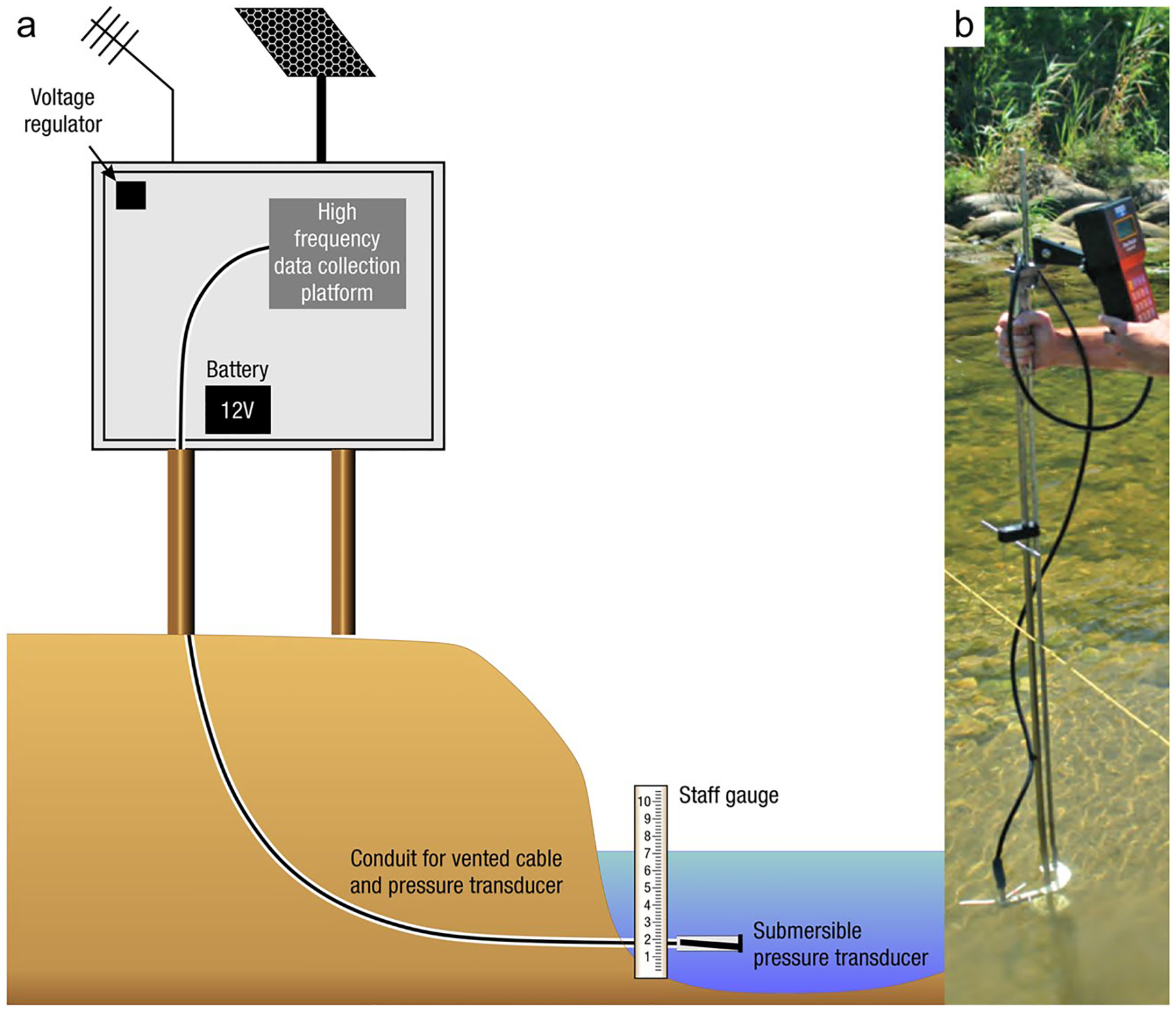
(**a**) Conceptual diagram of an automated measurement of stage (depth) using a staff gauge for discrete measurements and an automated pressure transducer attached to a high frequency data logging platform with battery power, solar panels, voltage regulator, and communications hardware; (**b**) field technician taking a discrete measurement of stream discharge using a handheld flowmeter. Paired discrete measurements of stage and discharge are used to develop site-specific relationships that can estimate streamflow discharge using continuous measurements of stream stage. Images from Sauer and Turnipseed (2010) (a) and Turnipseed and Sauer (2010) (b)

*How:* A gaging station is commonly used to relate measurements of stream stage to total flow (discharge) in the stream channel in terms of volume of water time^−1^ (e.g., L hr^−1^) (Fig. 25; Rantz 1982). Once a sufficient number of paired measurements of stream stage and discharge are made over a substantial range of streamflows, a stage-discharge relation can be established. Stream stage can more easily be measured continuously than measurements of discharge. With a stage-discharge relation determined, near-continuous record of streamflow discharge is possible using a pressure transducer. Flow also can be measured by routing water through an artificial structure, such as a flume or weir, that has a known relation between stage and discharge.

Several components are needed to collect, store, and transmit stream stage data. A submersible pressure transducer (Section “Water Sample Collection - Surface Water, Porewater, Groundwater”) that measures water depth based on the pressure of water above the sensor is placed adjacent to a manual staff gauge that indicates the stream stage. The pressure transducer output commonly is programmed so it matches the staff gauge. For longer deployments, it is ideal to have a data-collection platform with a power source such as a battery, a solar panel to charge the battery, a voltage regulator to prevent over-charging the battery, as well as a communications system for transmitting the data (Fig. 25a).

A manually read staff gage ideally is mounted to an object that does not move. Unless a bridge pier or equivalent is nearby, this can be a challenging objective. Streams and rivers are highly dynamic settings and staff gages need to measure surface-water stage over the full range, from a dry streambed to flood stage. Mounting a staff gage to a pipe driven deeply into the streambed is a common approach, but even deeply set pipes can move due to streambed scour, encounters with floating logs, or from moving ice flows. Therefore, annual surveys to reference marks of known elevation are commonly accomplished to document movement of staff gages.

Measurements of streamflow discharge can be made manually with handheld flowmeters (Fig. 25b) or with acoustic doppler profilers that can be handheld, permanently mounted, or deployed using crewed or autonomous aquatic vehicles. Another approach to quantify discharge is through conservative tracer dilution methods, which can be done through steady-state injections or slug injections (e.g., of dye or salts) to quantify water flow (Kilpatrick and Cobb 1985). For wetlands with poorly established stream channels, or where surface-water flow is exceptionally slow, increased spatial replication is required to establish the cross-sectional area through which streamflow occurs and determine the area-averaged direction and velocity of flow.

Concentrations of POC, DOC, DIC, *p*CO_2_ and *p*CH_4_, as well as environmental covariates like water temperature, can be measured through discrete grab sampling or with in situ sensors such as sondes (Section “Carbon in Wetland Waters”). Floating nets can be installed in streams to capture macro-detritus (POC > 2 mm) (Gao et al. 2018). The temporal and spatial variability in both flow and C concentration determines the frequency and locations of measurements required. Once spatially and temporally explicit measurements of water volumes and water fluxes are quantified, they can be combined with information on wetland surface areas and multiplied by C content to estimate lateral C fluxes in surface waters. When advective paths cannot be quantified, a modeling alternative to flow-based accounting is gradient-based monitoring of *p*CO_2_ concentrations (Ho et al. 2014, 2017).

*Key Covariates and Ancillary Measurements:* Many of the key covariates and ancillary measurements that characterize water chemistry and quality, such as turbidity, salinity, and temperature, are described in Section “Carbon in Wetland Waters”. For understanding of the drivers of water flow, it is recommended to measure antecedent precipitation, tidal cycle, topography, and water-table depth.

#### Groundwater Inputs and Exports

**Fig. 26:**
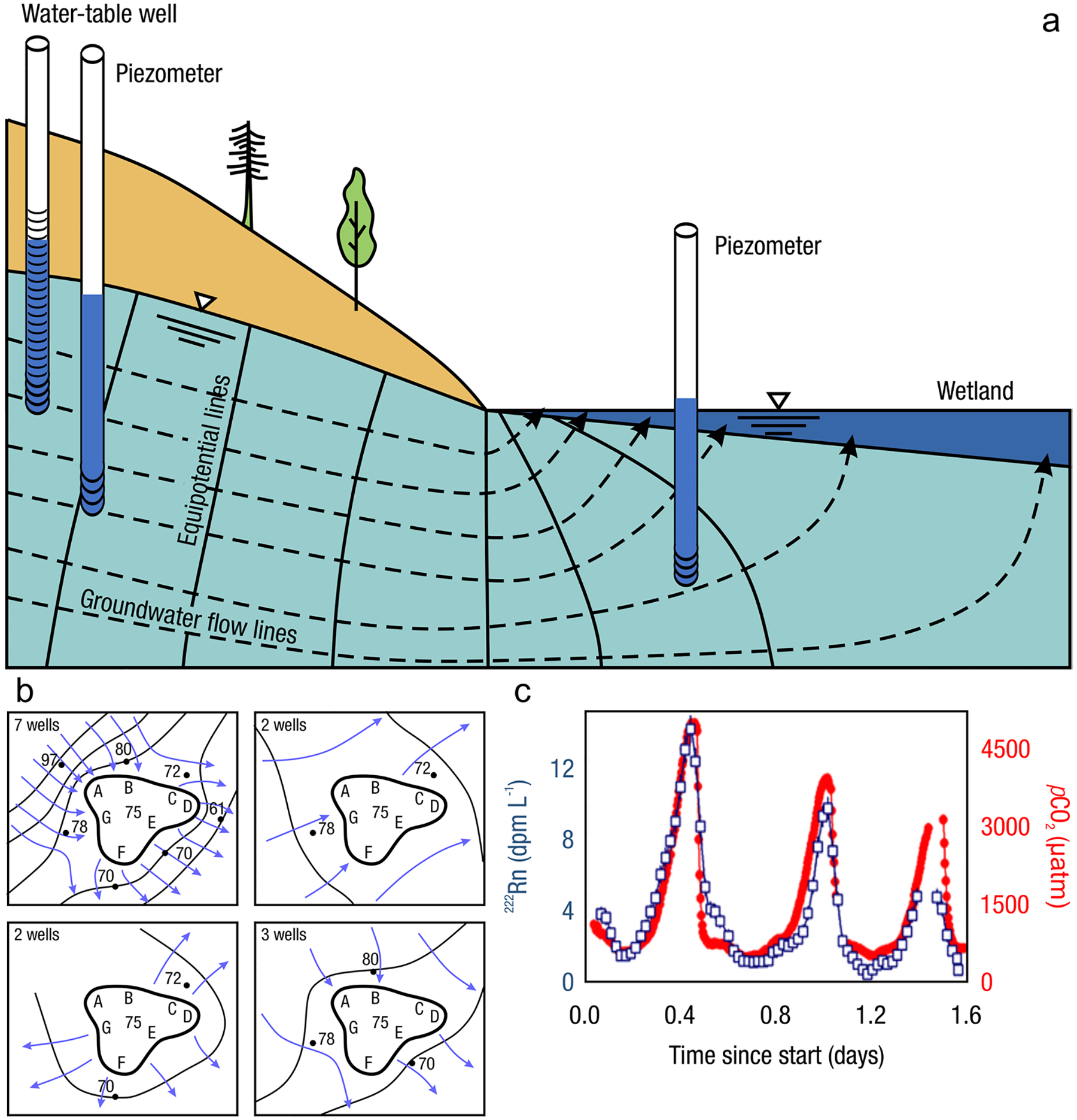
(**a**) Conceptual diagram of groundwater flow characterized using measurements of hydraulic head at specific locations relative to the wetland surface water using a water-table well and piezometers (Rosenberry and Hayashi 2013). The water-table well (left) shows higher hydraulic head of the groundwater relative to wetland stage on a horizontal axis, indicating the potential for groundwater to discharge laterally to the adjacent wetland. The piezometer (immediately to the right of the water-table well) has lower hydraulic head than the water table, indicating a downward component of movement of groundwater through the flow domain. The piezometer located within the wetland (right) shows hydraulic head that is higher relative to wetland stage, indicating groundwater has the potential to flow upward, into the wetland. Equipotential lines are lines of equal hydraulic head. Dashed lines indicate flow paths and direction of groundwater flow; (**b**) lines of equal hydraulic head in soils surrounding wetlands (thin black lines) and perpendicular groundwater flow lines (blue lines with arrow) based on a network of 7, 2, or 3 water-table monitoring wells surrounding a hypothetical wetland. Numbers indicate hydraulic head (m) associated with each water-table well and wetland stage (75 m). Water flows from higher to lower head. Letters indicate the wetland shoreline segment associated with each of the seven monitoring wells (Rosenberry and Hayashi 2013); (**c**) an example of coupling Radon-222 (.^222^Rn) (a natural groundwater tracer, squares with blue line) and the partial pressure of carbon dioxide (*p*CO_2_, dots with red line) demonstrating how sub-daily fluctuations in groundwater seepage contribute to aquatic C dynamics. Image from Rosenberry and Hayashi (2013) (a, b) and Santos et al. (2012) (c)

*What:* Water that flows below the water table is technically defined as groundwater flow (Fig. 26), although most wetland studies refer to shallow groundwater as porewater. To understand the direction of groundwater flow, measurements of hydraulic head (i.e., the elevation of groundwater measured in a water-table well or piezometer) and wetland stage (i.e., the elevation of the surface water level) are needed (Section “Water Sample Collection - Surface Water, Porewater, Groundwater”). Whether groundwater flow is into or out from a wetland is determined by the relative heights between the hydraulic head in soils adjacent to a wetland compared to the stage of the wetland. If hydraulic head is higher than the wetland stage, groundwater will flow toward the wetland; and the opposite, if wetland stage is higher than adjacent hydraulic head, water will flow away from the wetland.

Hydraulic head and gradient is commonly measured in surficial aquifers using water-table wells and expressed along a horizontal axis. Hydraulic gradient also can be determined along a vertical axis with multiple piezometers installed at different depths (Fig. 26a). Other methods also are available for quantifying groundwater exchange, depending on the scale of the setting (Rosenberry and LaBaugh 2008), including using radioisotopes tracers.

*Where:* The locations of groundwater flow into or out from a wetland can occur across the entire basin of the wetland. If soil adjacent to and beneath the wetlands are homogeneous, then the greatest rates of exchange are typically focused at the break in slope that occurs at the shoreline, where a sloping water table intersects a flat ponded water surface (Fig. 26a). In a tidal wetland, this point of maximal exchange tracks the oscillating surface water elevation, moving both horizontally and vertically in response to rising and falling tides. The rate of groundwater flow into a wetland will decrease exponentially with distance from shore. However, if soils are heterogeneous, the greatest flow will occur where soils are most transmissive (e.g., sand lenses). Many wetlands are highly productive and generate substantial litter that accumulates as decomposing organic matter on the soil surface. These organic accumulations likely transmit water (and C constituents) differently than areas with less accumulation, thereby adding heterogeneity in the locations of groundwater flow. Macropore structures, such as animal burrows and root-decay channels, can lead to preferential groundwater flow paths (i.e., ‘pipes’), increasing hydraulic conductivity and exchange between groundwater and surface water (e.g., Guimond et al. 2020).

*When:* Flow of groundwater and exchange with surface water occurs relatively slowly (e.g., weeks to years) compared to most other hydrological fluxes. Seasonal variability in groundwater flow is affected by precipitation patterns, such as large amounts or prolonged periods of rain, which often result in a subsequent period of greater groundwater discharge into wetlands. Similarly, groundwater flow is relatively high during spring snowmelt in parts of the world that receive snow. In tidal wetlands (such as mangroves and saltmarshes), tidal oscillations (Maher et al. 2013a), seasonal changes in relative sea level (Wilson et al. 2015b), and storm surges (Wilson et al. 2011) can all result in substantial flow and exchange of groundwater with surface water.

*Who:* There are a number of traditional techniques to quantify groundwater flow and exchange rates (Rosenberry and Hayashi 2013) that require basic training and some experience to conduct efficiently. Other techniques that employ natural tracers (e.g., radon, radium, and ions) or model-based approaches (Burnett et al. 2006) require additional expertise depending upon the approach used, from simple models with few input parameters to complete isotope mass-balance models (Krabbenhoft et al. 1994; Rosenberry and Hayashi 2013).

*How:* Determination of hydraulic gradient and hydraulic conductivity is most often accomplished with a network of monitoring water-table wells situated within tens to hundreds of meters of the wetland margin (Fig. 26a; see Section “Carbon in Wetland Waters” for details on water-well and piezometer hardware installation and use). The local hydraulic gradient is calculated as the difference between hydraulic head in a water-table well and surface-water stage, divided by the distance from the well to the nearest ponded shoreline of the wetland (m m^−1^). If the wetland has no ponded water such that a water table is beneath the soil surface, the hydraulic gradient is determined as the difference between two measurement points of hydraulic head, one adjacent to and one within the wetland. The rate of groundwater flow is calculated as the product of the hydraulic gradient and the ability of the soils to transmit water, termed hydraulic conductivity (m day^−1^). Hydraulic conductivity can be determined using single-well pumping tests (commonly called slug tests).

A network of monitoring wells provides the ability to develop a water-table contour map, from which groundwater flowlines can be drawn that, collectively, can distinguish wetland shoreline reaches where groundwater is discharging into a wetland or where wetland water is flowing out from the wetlands (Fig. 26b). Although more monitoring wells provide more precise information on the direction of groundwater flow, a minimum of three wells can usually provide a reasonable estimate of wetland-scale groundwater exchange (Rosenberry and Hayashi 2013).

A network of piezometers, which have a shorter screened interval (centimeters to decimeters) than water-table wells, can be installed to measure both horizontal and vertical hydraulic gradients. Because each individual piezometer indicates the hydraulic head at a specific point (location and depth), installing clusters of piezometers with screened interval at multiple depths can be used to determine hydraulic gradient on the vertical axis. The vertical hydraulic gradient is the difference in hydraulic head between adjacent piezometers, divided by the difference in screen depths. This can be particularly useful in locations with multiple subsurface sediment layers with differing hydraulic conductivities.

For localized groundwater exchange, a seepage meter (Rosenberry et al. 2020) can directly quantify the flow across the sediment–water interface of the area covered by the equipment, typically about 0.25 m^2^. Seepage meters must be deployed with care to reduce measurement errors, particularly in areas with flowing surface water (Rosenberry 2008).

*Radioisotope tracers:* If a conservative constituent (e.g., a chemical or other compound that is not altered by chemical or biological processes) is quantified in the groundwater from each monitoring water-table well and also the wetland water, a combined water and chemical budget can provide the net exchange of groundwater with surface water, as well as distinguish the groundwater flowing into and away from wetlands.

A suite of conservative, radioisotope tracers can be used to estimate groundwater flow and surface-water exchange rates (Fig. 26c). For example, ^222^Rn is a noble gas produced through the uranium (U) decay series via alpha decay of ^226^Ra (Section “Radiometric and Stratigraphic Dating - Laboratory Techniques”, Fig. 18). Most soils contain trace amounts of U, therefore ^222^Rn occurs in most groundwater and porewater. Since ^222^Rn is a gas with a relatively short half-life (~ 3.8 days), it is lost rapidly from surface waters due to both atmospheric exchange (evasion) and radioactive decay (Burnett and Dulaiova 2003). Therefore, a mass balance approach can be employed to determine groundwater discharge rates through the measurement of groundwater and surface-water ^222^Rn activity: assuming steady state conditions, groundwater discharge must deliver enough ^222^Rn to balance the combined ^222^Rn loss due to evasion and decay. C exchange can then be calculated by multiplying groundwater C concentrations (e.g., DOC, *p*CO_2_) by the volumetric groundwater flux. For example, Santos et al. (2012) coupled automated ^222^Rn and *p*CO_2_ sensors to show that submarine groundwater discharge was a source of CO_2_ to surface water (Fig. 26c). ^222^Rn has the advantage of being found in most subsurface water, regardless of salinity, and is particularly useful as a conservative tracer of dissolved gas transport in GHG studies.

When conducting Rn measurement, it is important to minimize gas exchange between the water sample and air. Water samples can be collected by lowering one end of a clear tube into the water source and the other end into the bottom of the sample container. The sample container is filled gently from the bottom using a peristaltic pump such that no bubbles are generated in the container; clear tubing is used to monitor for gas bubbles. The container is filled to overflowing and allowed to overflow ideally for an entire container volume and capped tightly with no headspace. It is important to begin analysis as soon as practicable after sample collection because of the short half-life of ^222^Rn (~ 3.8 days).

Radioisotopes of radium (Ra), radium-223 (^223^Ra), radium-224 (^224^Ra), radium-226 (^226^Ra), and radium-228 (^228^Ra), are another useful tracer of groundwater discharge. Like ^222^Rn, they are naturally occurring, derived from radioactive decay of their parent Th isotopes (also part of the U decay series). Therefore, these radioisotopes have a sedimentary source and tend to be enriched in groundwater and porewater relative to surface water. Unlike ^222^Rn, which is a dissolved gas, these Ra isotopes all exist as cations (Ra^2+^). As such, cation exchange with aquifer solids is an important (often dominant) control on dissolved Ra activity. Therefore, Ra activities tend to be low in fresh (no/low salinity) ground-water because most Ra cations remain adsorbed to the aquifer solid phase. As a result, Ra is more frequently used as a groundwater tracer in coastal brackish and saline aquifers where competition with other abundant marine cations (Na^+^, K^+^, Ca^2+^) drives Ra^2+^ into solution, yielding relatively high and more easily quantifiable Ra activities. These four Ra isotopes have a wide range in half-life, with ^226^Ra and ^228^Ra having half-lives of 1,600 and 5.8 years, respectively, while ^223^Ra and ^224^Ra having half-lives of 11.4 and 3.6 days, respectively. The four Ra isotopes have been used as tracers of water movement and mixing in wide range of applications (review by Garcia-Orellana et al. 2021). Generally, ^228^Ra and ^226^Ra are used as tracers of processes active over longer temporal and spatial scales (e.g., long groundwater flow paths), while ^224^Ra and ^223^Ra are more useful over smaller scales. In addition, due to differences in the amount of time it takes for each isotope to come into secular equilibrium (production equal to decay) with its parent Th isotope, isotope ratios (e.g., ^228^Ra:^226^Ra) can be used to estimate groundwater residence times. Furthermore, aquifers typically have unique Ra isotope ratios, which can be used to ‘fingerprint’ the dominant sources of groundwater from a heterogeneous mix of shallow, tidal wetland aquifers (Schutte et al. 2020). As with ^222^Rn, groundwater discharge can be estimated by building a mass balance of one or more Ra isotopes that includes all source and sink terms and volumetric groundwater flux (e.g., Porubsky et al. 2014).

When collecting water for Ra measurement, the water volume must be appropriate to the expected Ra activity. Fresh water may require tens of liters, while only 2 to 4 L may suffice for saline groundwater. For the shorter-lived isotopes ^223^Ra and ^224^Ra, analysis must be completed as soon as possible after collection. It is convenient to collect Ra samples into Cubitainers because they are collapsible, come in a variety of sizes, and have threaded openings to attach tubing easily. Water samples are gravity-drained at a rate not to exceed 1 L min^−1^ through 15 to 20 g of manganese-coasted acrylic fibers that are held in a clear, rigid plastic tube and connected to the Cubitainer with flexible tubing. The manganese-coated fibers trap Ra that is dissolved in the water sample, concentrating it for laboratory analysis using a gamma ray spectrometer (Moore 1984). It is important to minimize sediment on the manganese-coated fibers. For sediment-laden water samples, the Cubitainer can be set up carefully such that most of the sediment settles to the bottom of the Cubitainer. A small wad of acrylic fiber that is not coated in manganese can be placed between the sample and the manganese fiber as a pre-filter. The full and empty Cubitainer masses can be measured in the field using a hanging scale to calculate the volume of water filtered.

*Key Covariates* and *Ancillary Measurements:* Key covariates and ancillary measurements that are relevant to lateral C flux in water are described in the water sampling approach of Section “Carbon in Wetland Waters”, including water temperature, electrical conductivity, pH, salinity, turbidity, DO, and Chl-*a*.

#### Overland Inputs from Upland Runoff

*What:* Flow of water and associated C into a wetland over the land surface that does not enter the wetland via channelized flows is termed ‘diffuse overland flow’, also referred to as ‘runoff’. This term often is small and not always measured, or it is determined as the residual of a complete wetland water budget (i.e., after accounting for all other hydrological fluxes). However, during prolonged periods of rain, or during spring snowmelt, overland flow can be substantial and deliver a large amount of C as POC, DOC, and DIC to a wetland, especially if large quantities of decaying plant materials (e.g., litter) are present at the location that water is flowing into a wetland. In other cases, overland flow may bring large amounts of sediment with low C content, lower soil C density in wetlands. For depressional wetlands, overland flow out of a wetland can occur over a broad spill area, which can temporarily connect geographically isolated wetlands with adjacent wetlands and streams (Vanderhoof et al. 2017).

*Where:* Overland flow can occur along all or a large portion of a wetland perimeter whenever the land surface is sufficiently saturated with water and has a slope toward a wetland steep enough to convey water. In more hummocky landscapes, such as those found in the Prairie Pothole Region of North America, much of surface (and shallow subsurface) flow will concentrate within relatively narrow, convergent areas of the land (valleys) surrounding the wetland. These highly focused flow paths may account for the majority of the inflowing water into a wetland. Overland flow is typically greater in soils with low permeability. In cooler climates, snowmelt over frozen ground is a primary source of inflow. Efforts should be taken to identify both diffuse and focused runoff paths and situate flow traps (described below) for both types of flow.

*When:* Overland flow is nearly always ephemeral with flow occurring typically following large or prolonged periods of rainfall or during snowmelt.

*Who:* Setting up flow traps is relatively straightforward and requires limited training. Like other components of a wetland water budget, knowledge of hydrography is required to determine where and when to deploy traps.

**Fig. 27:**
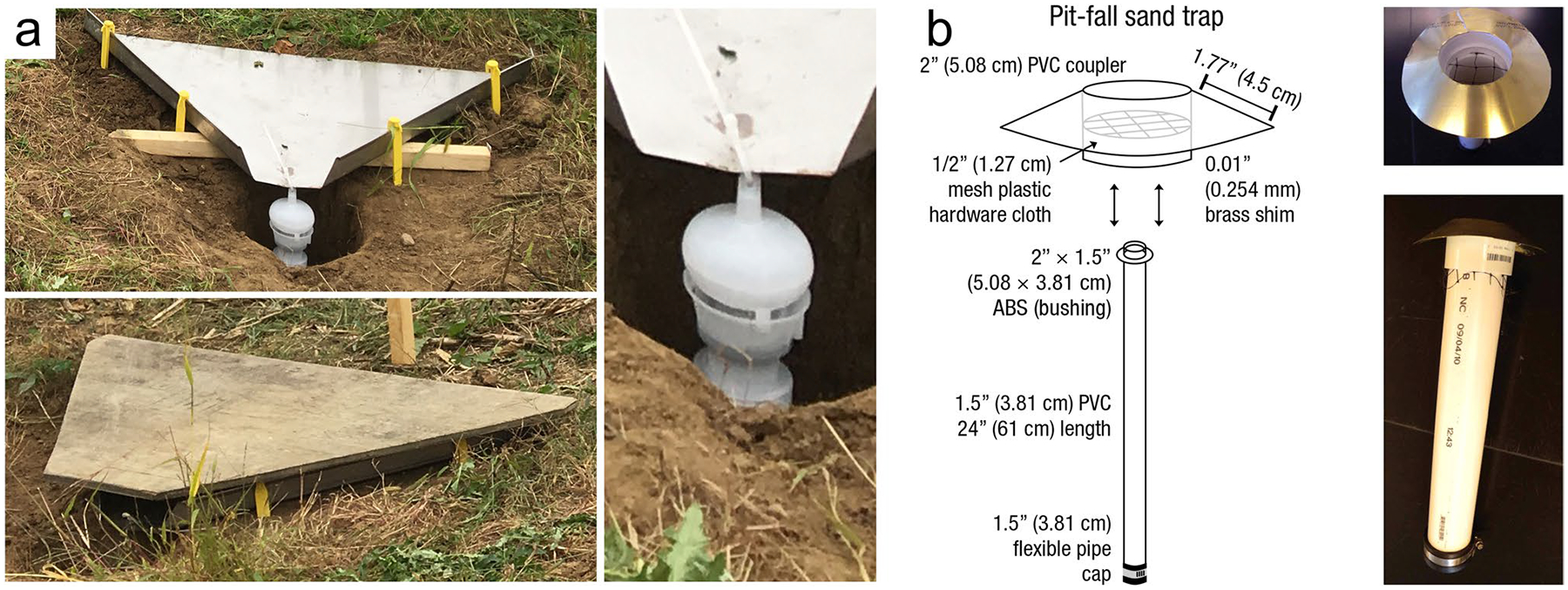
(**a**) Images of flow traps (runoff trays) that direct overland flow to a single location for collection and analysis; with protective cover off (top left) and on (bottom left), with sample bottle (right, I-Chem Storm Water Sampler from Forestry Suppliers) with cover. The plastic cover over the bottle slows the rate of water collection, thereby providing a better representation of water from an entire runoff event, not just the start of the event. A coarse filter below the dome is used to prevent clogging by coarse debris. A drainage system (e.g., trench) is excavated downslope from the collection hole to avoid overtopping of the collection bottle (Page et al. 2020); (**b**) diagram and picture of a pit-fall sand trap used to estimate contribution of aeolian sand to sedimentation in a backbarrier marsh (Rodriguez et al. 2013). Images used with permission from Bryan Page (a) and Antonio Rodriguez (b) [ABS, Acrylonitrile–Butadiene–Styrene; PVC, polyvinyl chloride]

*How:* Accurate quantification of overland flow can be challenging and commonly is accomplished over a small area and extrapolated over a larger area where it is determined to occur. One common method is to construct a flow trap that consists of a barrier that intercepts and then routes overland flow to a single location. Flow is then calculated based on the amount of water collected per event or time, and water samples are also collected to determine concentrations of various C constituents (Fig. 27a) (Section “Carbon in Wetland Waters”). Adding protective covers over the traps and collection vessels can reduce clogging from extraneous coarse debris (Page et al. 2020). In some instances, large and rapid overland flow events may cause collection vessels to fill rapidly before the end of the event. A dome cover placed over the collection vessel can slow the rate of fill to extend the period of collection to help capture the entire flow event.

*Aeolian transport of wetland C (inputs from soil erosion and sedimentation):* There are relatively few studies on the effects of wind erosion on wetland sediment (e.g., Adib et al. 2018; Gao et al. 2019). In sub-humid, semi-arid and arid environments, such as the Northern Great Plains of North America, and the Prairie Pothole Region in particular, wind erosion may be a dominant geomorphic process, presumed to be the major form of soil erosion and sedimentation, and therefore a significant form of lateral transport of organic and inorganic C into wetlands from the surrounding landscape. Sediment and soils can be transported laterally into and out of wetlands through aeolian transport, which can lead to vertical accretion or loss of sediment (de Groot et al. 2011; Rodriguez et al. 2013). Ephemeral wetlands, such as playas, can be important sources of windblown sediment (Rivas et al. 2019). Anthropogenic alterations to the landscape, such as for forest or agricultural management, can dramatically increase the production and transport of sediment by wind (and water) by leaving soils bare and disturbed, even if only for short periods (Forman et al. 2008; Owens et al. 2016; Sapkota and White 2019). The creep (i.e., dragged particles too heavy for wind to lift) and saltation (i.e., particles moved by wind but too large to become suspended in the air) fractions of wind-eroded sediment from the wetland catchment largely get trapped in the riparian vegetation surrounding the wetland, with only the suspended sediment fraction making it into the wetland. Evidence of such wind-eroded sedimentation is observed as an asymmetric pattern around the wetland, reflecting the prevailing wind direction.

Erosion triggered by tillage causes the progressive downslope movement of soil and creates a ‘tillage step’, which can be observed in the outer riparian area surrounding wetland. In agricultural catchments, this form of erosion can dominate the movement of soil into the wetland environment. Within depressional wetland catchments, erosion of upland soils can be quite high, moving soil and sediment (and associated C, N, and P) into wetland basins, particularly along wetland edges and riparian zones (Habibiandehkordi et al. 2019; Zarrinabadi et al. 2023).

Principal factors affecting aeolian transport and capture in wetlands include wind speed and direction (particularly during storm events), wetland and catchment morphology, particle size and composition (e.g., organic or mineral), surface moisture and surface conditions (e.g., snow-covered), and vegetation cover and structure (Kuzovkina and Quigley 2005; Adib et al. 2018; Gao et al. 2019; Rivas et al. 2019). To assess aeolian transport, a series of pit-fall sand traps (Fig. 27b) can be distributed within wetlands. The distribution of such traps is critical in assessing aeolian transport versus other sedimentation transport processes (e.g., overland flow). Aeolian sediment is collected, weighed periodically, and can be analyzed for C, nutrients, contaminants, seeds, and propagules (Rivas et al. 2019). A high-resolution digital elevation model using RTK GPS or LiDAR, along with anemometers to measure wind speed and direction, can aid in the interpretation of results by enabling the identification and characterization aeolian sedimentary features, and distinguishing them from features created by water erosion and tillage erosion (Sankey et al. 2010). Atmospheric P is deposited as dust that is composed of P-bearing mineral and organic materials (e.g., soil, soot, pollen), and therefore, can be used as a proxy for C inputs. The amounts and patterns of soil erosion and sedimentation within a wetland catchment can also be determined using an array of ^137^Cs inventory measurements, non-eroded ^137^Cs reference sites, and a ^137^Cs mass balance approach (Zhuang et al. 2015). These data, coupled with ^137^Cs and ^210^Pb profile distributions and inventories within the riparian and open-water areas, and measurements of the enrichment of clay, organic C, and P in the materials accumulating can be used to quantify wind-eroded sediments and distinguish them from water- and tillage-eroded sediments (Zarrinabadi et al. 2023).

*Anthropogenic removal*: Aside from lateral transport of C through hydrologic pathways, C can also enter and/or leave a wetland through anthropogenic removal. Anthropogenic removal, such as tree or peat harvest, can have implications for wetland C budgets and post-harvest effects on C fluxes (Roulet 2000; McKee et al. 2012; Kolka et al. 2022).

*Key Covariates and Ancillary Measurements:* The key covariates and ancillary measurements for overland lateral flux are the same as those described in Section “Carbon in Wetland Waters”, including weather conditions and precipitation. Automated sensors (of C constituents, covariates, or flow rate) that can be deployed in situ and collect, store, and transmit data may be needed to accurately characterize overland flows.

## Upscaling in Space and Time: Wetland Carbon Modeling and Remote Sensing

### Definitions and Units

**Fig. 28:**
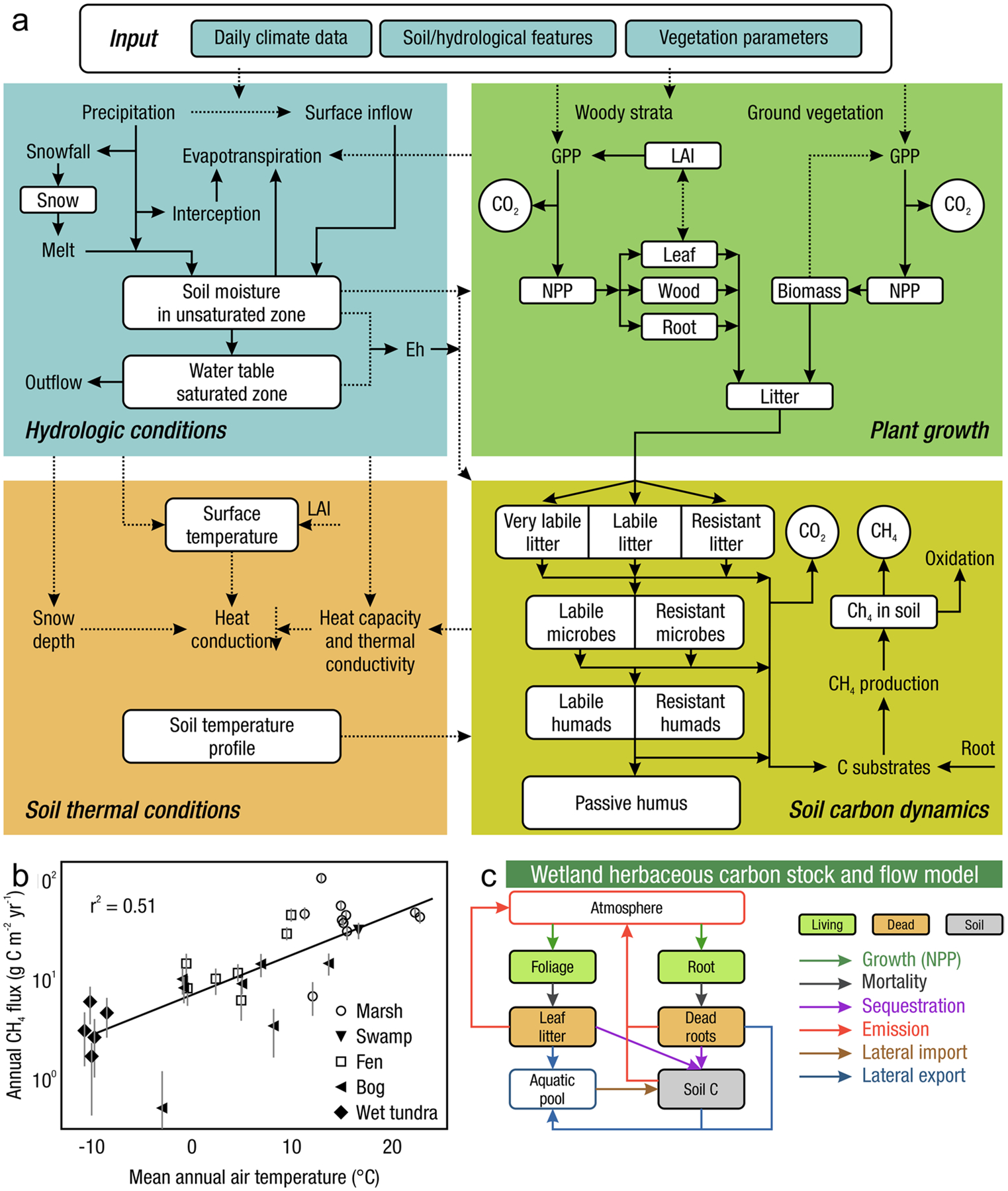
Example of (**a**) a conceptual process-based model Wetland-DNDC (Zhang et al. 2002; Lloyd et al. 2013); (**b**) eddy covariance annual methane (CH_4_) emissions from multiple wetland types (symbols) as a function of mean annual temperature (black line) (Delwiche et al. 2021); (**c**) a carbon stock and flow model of an herbaceous wetland. [C, carbon; CO_2_, carbon dioxide; Eh, redox potential; GPP, gross primary productivity; LAI, leaf area index; NPP, net primary productivity]

*Definitions:* While the preceding sections describe measurements of individual C pools and fluxes within the wetland C cycle, many study objectives require models to estimate multiple C pools and fluxes over large spatial and temporal scales. Ecosystem C modeling combines data from various sources (e.g., ground-based measurements, remotely sensed measurements, laboratory-derived rates) to develop local- to global-scale estimates of C pools and fluxes (Fig. 28). The prevalence of anaerobic biogeochemical processes driven by hydrological conditions is a principal feature of wetland C models that distinguishes them from upland C models. In addition, the potential exchanges of C between wetlands and adjacent aquatic systems also differentiate wetland C models. Process-based ecosystem C models are often used to forecast changes in wetland C pools or fluxes in response to environmental drivers such as climate change, climate variability, management actions, or disturbances. Data-driven ecosystem C models (also referred to as ‘statistical’, ‘empirical’, or ‘observation-based’ models) often have relatively high predictive power, but also have very high data requirements and may be difficult to interpret.

**Fig. 29:**
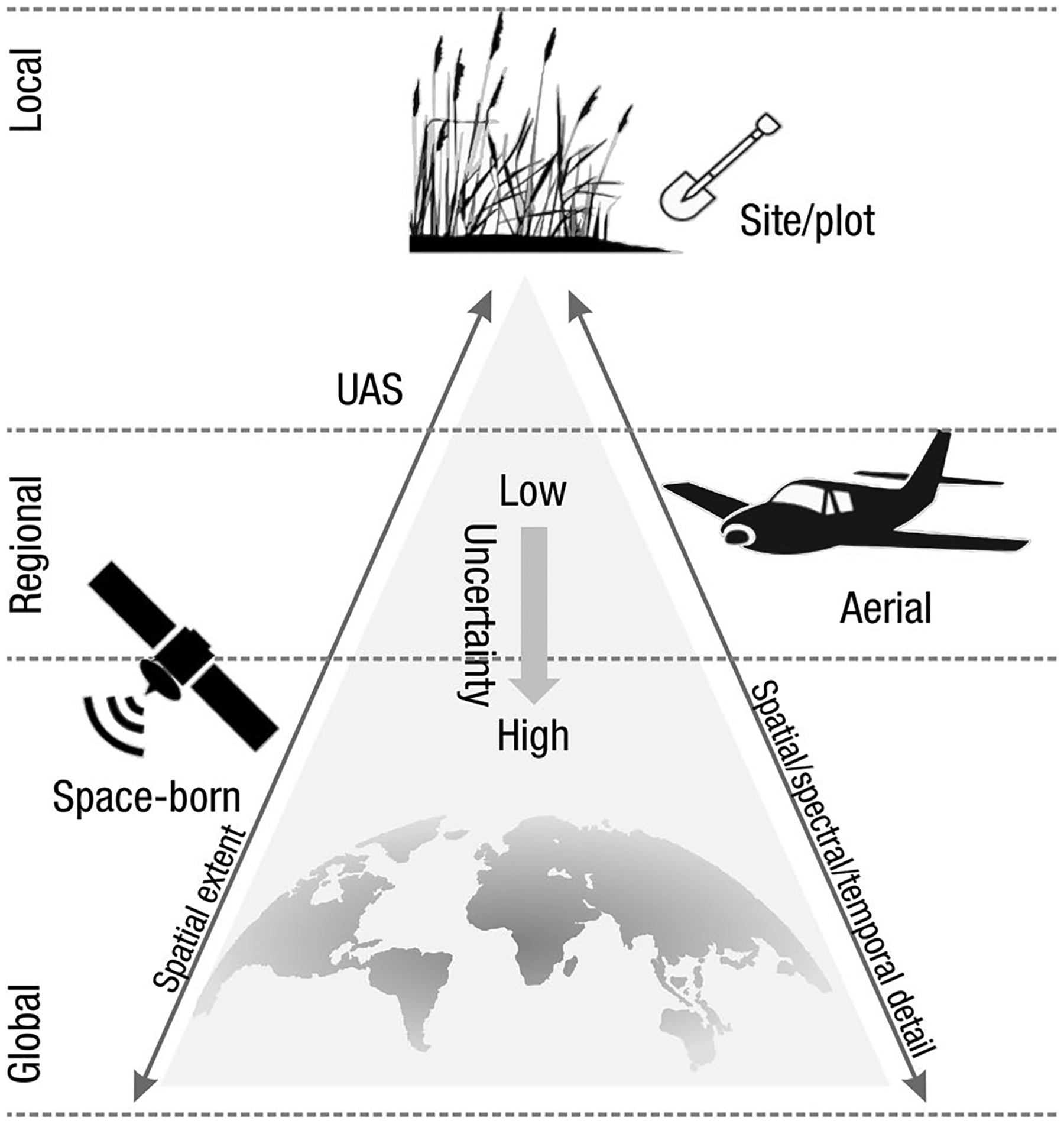
Carbon monitoring systems and platforms in relation to uncertainty and remote sensing resolution domains. Site/plot scale data (top) have lower uncertainty, but also have the lowest spatial scale of inference. Space-born satellites can provide global-scale data, but also have the highest uncertainty (modified from Campbell et al. 2022). [UAS, Uncrewed Aircraft Systems]

Remote sensing is a broad term for data retrieval methods that use sensors physically separated from the wetland ecosystem of interest, typically obtained from satellites, aircraft, uncrewed aircraft systems (UAS, drones), tower-mounted cameras (e.g., PhenoCam), or other airborne platforms (Fig. 29). Remote sensing models use optical data (e.g., reflected solar radiation) to map and classify wetlands around the world, as well as to characterize wetland hydrological regimes, biological processes (e.g., phenology), and physical features (e.g., topography). Application of remote sensing methods in wetlands poses unique challenges due to the high spatial heterogeneity and temporal variability of wetland features driven by hydrological fluctuations (Klemas 2013; Byrd et al. 2014; Mishra et al. 2015; Tiner et al. 2015). The physical interactions between water and electromagnetic radiation present additional difficulties for characterization of wetlands compared to uplands. Remotely sensed data can be acquired through several open-source datasets (e.g., Landsat satellite archive; Li et al. 2021) or through commercial sources. In general, remote sensing models of wetland C pools and fluxes are relatively under-developed compared to models for upland systems (e.g., forests); within the wetland community, most modeling efforts have focused on coastal wetlands and then peatlands, but other wetland types (e.g., mineral soil wetlands) are far less studied (Campbell et al. 2022).

Combining ecosystem modeling with remote sensing can be an extremely useful tool for estimating wetland C pools and fluxes over space and time. However, the required field and laboratory data needed for parameterization, calibration, and validation of models are often sparse or non-existent for many wetland types and locations, leading to high uncertainty in wetland C estimates. Models can help by identifying the largest sources of uncertainty in the wetland C pools or fluxes being simulated, which can then guide new field and laboratory investigations (Clark et al. 2011; Dietze et al. 2013). The topics of ecosystem modeling and remote sensing are vast and beyond the scope of this review. Here we present an overview to help field and laboratory researchers understand how the utility of their data can be improve and used in models of wetland C pools and fluxes.

**Rationale:** Landscape-scale field sampling campaigns to assess wetland C dynamics rarely have enough data to characterize averages and distributions of the C pool sizes or flux rates through repeated, ground-based measurements alone. Accordingly, data-driven or process-based models are required for upscaled assessments of wetland C pools and fluxes based on observations from individual or multiple studies. The largest scale of such assessments is global, such as the IPCC Assessment Reports. Large advances have been made in incorporating ecosystem C cycle processes into the earth system models (ESMs) from the first IPCC assessment report (AR1) in 1990 to the sixth report (AR6) in 2021 (IPCC 2021). However, large-scale ESMs have difficulty in accurately representing C cycling in wetlands due to their biogeochemical complexities and challenges imposed on remote sensing by the presence of surface water (Bailey et al. 2003; Poulter et al. 2022). Even in the AR6, climate feedbacks from wetland CH_4_ and CO_2_ fluxes are not included in most climate models. Continued improvement of large-scale wetland C models with new field and laboratory observations will help reduce uncertainty in estimates of global wetland GHG budgets. Efforts to develop off-the-shelf wetland C pool and flux estimates are underway (e.g., NASA Carbon Monitoring System) to facilitate monitoring, reporting, and verification (MRV) of wetland C budgets. Improved estimates of wetland C pools and fluxes will provide essential guidance to policy makers and land managers about how to best regulate and manage wetlands as nature-based climate solutions to meet national and international GHG emissions and C sequestration objectives.

### Wetland Carbon Modeling

*What:* Generally, models have parameters, or predictors, which have coefficients that need to be calibrated, and once calibrated, the model results need to be validated using a variety of methods. There are two main modeling approaches, process-based and data-driven, which are used for modeling wetland C dynamics. Data-driven models generally do not use a priori parameterizations and are therefore more dependent and constrained by the amount and representativeness of data used to build the model. For this reason, observation-based models also tend to display more erroneous/biased behavior outside of data training conditions. Process-based models use knowledge of processes and functional relationships among variables based on well-established causal mechanisms (e.g., photosynthetic responses to light, temperature, and CO_2_ concentrations) into relevant inputs and functional forms that are embedded in models. It should be noted that, at some level, all process-based models are empirical in nature, with parameters calibrated with results from experimental or observational studies. Newer modeling approaches incorporate aspects of both process-based and data-driven models (Clark and Gelfand 2006; Mohankumar and Hefley 2022). Some modeling approaches, such as C stock and flow models (also known as ‘gain–loss’ methods), represent wetland C as a series of inter-related C pools and fluxes, and one or more external pools of C (such as the atmosphere or ocean). These kinds of models represent an intermediate solution between heavily parameterized, computationally intensive process-based models and complex data-driven models that are challenging to interpret (Sleeter et al. 2022).

*Where and When:* The spatial and temporal scopes of inference of models are generally constrained by the range of input data, especially for data-driven approaches. Process-based models are designed to be generalizable, and thus can extrapolate beyond the range of data, although uncertainty increases with greater extrapolation.

*Who:* Simple data-driven models, such as regressions, can be developed by individuals with basic statistical backgrounds. More complex, multi-parameter modeling often requires individuals with theoretical training as well as experience using one or more programing languages (e.g., R, JavaScript, Fortran, C, Python, MATLAB). Process-based modeling also requires subject matter expertise to parameterize models and define functional relationships among variables.

*How:* Modeling, whether data-driven or process-based, involves combining data to derive numerical expressions that produce predictions (Luo et al. 2011).

*Process-based models:* Process-based models aim to simulate the underlying mechanisms that influence wetland C pools and fluxes (Fig. 28a). These models have specific domains of space and time, which may be targeted to an unknown C pool or flux, or attempt to address the whole C cycle among other biogeochemical cycles (water, N). For example, a process-based model of CH_4_ fluxes may include aspects of photosynthetic C inputs, decomposition of C substrates, and microbial processes (Zhang et al. 2002; Grant et al. 2015; Schädel et al. 2020). Because process-based models make predictions based on a mechanistic understanding of ecosystem processes, this class of model is crucial to forming testable hypotheses and making predictions of ecosystem response to previously unobserved conditions, such as those associated with rising atmospheric CO_2_ concentrations, relative sea-level rise, and extreme events. However, caution is still advised when making predictions that are beyond the range of observations as some model assumptions may not hold true under novel conditions.

Process-based models of the wetland C cycle typically require a host of parameters for initialization. Use of field and laboratory results from within the modeling domain (i.e., the target wetland ecosystem) is preferable for model parameterization, calibration, and validation, although those data and associated metadata are often lacking in the literature. If empirical data needed for model parameterization are not available, then model default parameters initially rely on information from other ecosystems (e.g., uplands) (Morris et al. 2016). Subsequently, model calibration is achieved by adjusting parameters to best fit observed responses in wetlands. It is important that the scale of data used in calibration matches the scale at which the process is represented in the model (e.g., monthly temperature response of ecosystem GPP should not be calibrated with instantaneous temperature response of leaf-level photosynthesis). When a single wetland C pool or flux is unmeasured, some studies attempt to fully account for and model all other wetland C pools and fluxes and use the residual as an estimate of the missing wetland C pool or flux (Krauss et al. 2018b).

Many process-based models provide a framework for considering anaerobic soil biogeochemical processes (Grant et al. 2015). The Denitrification Decomposition model (DNDC) developed by Li et al. (1992) explicitly incorporates soil redox reactions, providing the basis for the development of Wetland DNDC, an ecosystem scale wetland C model (Zhang et al. 2002; Fig. 28a). Since anoxic soil conditions are usually driven by hydrological conditions, a significant challenge in modeling wetland biogeochemistry is simulating the hydrological regime. Simplified representations of wetland hydrology are typically used for point-scale assessments, but this approach is not feasible when simulating watersheds or larger areas. The coupling of watershed-scale hydrological and biogeochemical models occurs (e.g., RHESSys, Tague and Band 2004), but is often not practical. For this reason, separate models for hydrology and biogeochemistry can be coupled. For example, Dai et al. (2012) used a coupled modeling framework employing MIKE SHE (DHI 2017) to simulate watershed hydrology and Forest DNDC to simulate C dynamics in uplands and wetlands within the watershed. Complex models also incorporate microbial processes (Chang et al. 2020), which generally rely on laboratory-based studies for process rates (e.g., The Soil Incubation Database [SIDb]; Schädel et al. 2020).

Process-based models for simulating C dynamics in wetlands are often developed for specific wetland systems (Melton et al. 2013; Xu et al. 2016). Organic soil wetlands or peatlands have distinct physical and chemical properties compared to mineral soil wetlands, which influences the C dynamics. As an example, focusing on the northern peatlands, St-Hilaire et al. (2010) developed the McGill Wetland Model to provide a tool for predicting C sequestration and turnover in northern peatlands. The C dynamics in coastal marine wetlands, mediated by marine biogeochemistry and tidal hydrology, are quite different from C dynamics in terrestrial wetlands. Accordingly, Dai et al. (2018) developed the Mangrove Carbon Assessment Tool to simulate the C dynamics in coastal marine forests.

*Data-driven models:* Data-driven models are built using statistical relationships between response variables and predictors (Fig. 28b). These empirical approaches can range from simple regressions with variables selected by investigators, to more complex regressions (e.g., generalized additive modeling) or machine learning algorithms (e.g., artificial neural networks, random forest, gradient boosted decision trees) with automated variable selection procedures that incorporate a large number of potential predictors (e.g., Warner et al. 2019; Bansal et al. 2023; Ueyama et al. 2023). Models that are developed using linear correlations or simple regressions are easy to interpret, but often have lower predictive accuracy and precision than machine learning algorithms. Models developed through machine learning and deep learning algorithms generally have higher performance because they can reproduce non-linear relationships and interactions without the need for underlying statistical assumptions or *a priori* knowledge of processes. However, the relationship between environmental drivers and wetland C responses may be difficult to interpret. Data-driven models may be subject to overfitting input data, meaning the model performance is overestimated, and the model becomes less applicable outside the spatial and temporal range of data used to build the model. Consequently, proper validation and test strategies are needed to avoid overfitting (Roberts et al. 2017; Meyer et al. 2019). Test and validation strategies also help report bias in data-model agreement.

New methods are being developed (e.g., conditional importance rankings, partial dependency plots, and Shapley Additive exPlanations values) to improve the interpretability of machine learning models. Physics-informed machine learning approaches combine data-driven and process-based modeling approaches; examples include using machine learning to optimize process-model parameters or combining Bayesian statistics and machine learning to improve spatial predictions (Mohankumar and Hefley 2022). Some data assimilation approaches allow for data collected via different methods and scales to be synthesized in a common, hierarchical framework (Collier et al. 2018). For example, Wilkinson et al. (2018) used a Bayesian hierarchical framework to estimate C accumulation rates in wetlands from disparate field and laboratory studies by accounting for uncertainty in data provided in individual studies (e.g., means and standard errors).

Data representativeness, especially at larger regional to global scales, remains a potential weakness for data-driven models, whereby model selection is influenced by geographic availability of input data (Jung et al. 2020). Methods to assess uncertainty for machine learning are also not well developed and inconsistent across studies; ensemble-based uncertainties are likely to be too narrow (too confident) and scaling approaches have been recommended in Irvin et al. (2021). Artificial intelligence (AI) models are often able to assess uncertainties and errors if sufficient data exist (Grunwald 2022).

*Carbon stocks (pools) and flows as a model:* Process-based models of wetland C rely on a conceptual model of C stocks and flows (Fig. 28c) as parts of an ecosystem C cycle (or ‘budget’). Note, the term ‘stock’ is typically used to describe individual C pools in these models. The conceptualization of ecosystems as stocks and flows of energy or elements is nearly as old as the discipline of ecology itself (Taylor 1988), starting with G.E. Hutchinson’s representation of the global biogeochemical C cycle as a “Circular Causal System” (Hutchinson 1948) and H.T. Odum’s representation of ecosystems as electrical circuits (Odum 1956a). C stock and flow models are not only of conceptual importance, but also serve as a simple empirical model with considerable power in modeling C dynamics at the landscape scale. While it is theoretically possible to track C in wetland ecosystems through repeated measurements of stocks alone, termed a ‘stock-change’ method, it is rarely practical to maintain such a large monitoring program for long periods of time. Therefore, often both stocks and flows are estimated or modeled based on literature values.

C stock and flow models may be initially parameterized against more complex process models and/or empirically measured C pools and fluxes. Such intermediate complexity models are often embedded in hierarchical frameworks to increase computational efficiency. One example of this approach is the use of stock and flow models embedded within state-and-transition simulation models of land-use and land-cover (LULC) change (Daniel et al. 2018; Sleeter et al. 2019). Stock and flow parameters for C pools and fluxes for each LULC class can be modeled from field data or drawn from a distribution of such values. Future C dynamics under different LULC scenarios can then be characterized in a Monte Carlo framework that combines uncertainties of C stocks and flows with those regarding LULC transitions. In this way, measurements of C pools and fluxes can be combined with remotely sensed LULC in a conceptually simple, yet highly dynamic, modeling platform for evaluating management decisions on ecosystem C at the landscape scale (Sleeter et al. 2019).

### Remote Sensing

*What:* Remotely sensed data can be used to identify and analyze different types of Earth surface features, including wetlands. Various sources of remotely sensed data provide optical and physical data to measure wetland characteristics. Remotely sensed data can broadly be acquired by passive or active sensors (Fig. 29). Both types of sensors can be either mounted on hardware (e.g., platforms, poles, or towers), or attached to UASs, airplanes, or satellites. Passive sensors measure electromagnetic energy from the sun that is reflected off objects on the ground (referred to as ‘spectral reflectance’). Many satellites, such as Landsat-4, −5, −7, −8, −9, Sentinel-2, and Wordview-1, −2, −3, −4, use passive sensors that generally do not penetrate through clouds, vegetation, or tree canopies, and the data are affected by shadows from vegetation and topography. In contrast, active sensors (e.g., LiDAR, synthetic aperture radar [SAR]) measure changes in electromagnetic energy that originate from the sensor. Active sensors can generally penetrate clouds and vegetation, though the degree of penetration varies with wavelength and the density of vegetation. Selection of the appropriate sensor dataset to achieve study objectives requires an understanding of accuracy, and spatial, temporal, and spectral resolution of various sensors.

Sensors are typically optimized for a particular application, and therefore capture different parts of the electromagnetic spectrum, referred to as bands. For the purposes of wetland mapping and C flux modelling, the forthcoming (*circa* 2023) NASA-ISRO Synthetic Aperture Radar (NISAR) satellite will operate in both S-band (suitable for the detection of changes in Earth’s surface structure and roughness) and L-band (sensitive to changes in the dielectric properties). This increased capability will result in more precise measurements of various Earth surface attributes such as topography, vegetation, and soil moisture.

Remote sensing modeling approaches are typically either pixel-based or object-based. For pixel-based approaches, statistical models can be derived where spectral band data serve as the predictors and the ground-truth data serve as the response variable, which is called ‘supervised classification’. If models perform well, they can be used to predict pixel responses in areas without ground-truth information, providing landscape-scale spatially explicit predictions. The complex, dynamic, and patchy matrix of wetland waters, soils, and vegetation cause areas to fluctuate between open water, mudflats, floating vegetation, and sparse to closed canopy, resulting in mixed land-cover pixels that change over short time periods (Rivero et al. 2007, 2009).

Spectral band data are highly correlated with each other. Thus, for parametric statistical approaches such as linear regression, spectral band data are often simplified into spectral indices, such as the Normalized Difference Vegetation Index (NDVI), where multiple bands known to have particular physical relationships with ground-truth data are simplified into a single number. Indices are also useful because ratios among bands often predict wetland characteristics better than the bands themselves. Alternatively, machine learning (e.g., random forest) and multivariate latent variable approaches (e.g., partial least squares regression) are less sensitive to correlated predictors and can use all spectral information without the need for spectral indices (Smith et al. 2002; Mevik and Wehrens 2007).

An alternative to (or in combination with) pixel-based approaches is object-based image analysis (OBIA). OBIA is especially useful when working with datasets with high spatial resolution (i.e., small pixels) but low spectral resolution (i.e., few bands) as it allows for the use of additional object characteristics beyond spectral information, such as object features (e.g., wetland shape and size). For OBIA, spectrally similar pixels are grouped into objects as polygons through a process called segmentation (Dronova et al. 2011; Halabisky et al. 2011; Hossain and Chen 2019). Once an image is segmented, the user can classify objects of interest using spectral, spatial, and temporal characteristics of the object and its relationship to other objects through a set of user defined rules or statistical methods. Spatial and hierarchical relationships between objects can be applied to identify and classify multiple segmented objects as wetlands by grouping and relating polygons to each other (Blaschke 2010; Georganos et al. 2018).

Objects, once segmented and classified, can also be used to summarize spatial and temporal pixel-based analysis for individual wetlands and used to explore the variability of model results across wetland types (Halabisky et al. 2016). Data summarized at the wetland scale can be helpful in relaying results to policymakers and practitioners who may be more familiar with viewing wetlands as delineated objects similar to polygon-based wetland inventories (e.g., U.S. Fish and Wildlife Service’s National Wetlands Inventory).

**Fig. 30:**
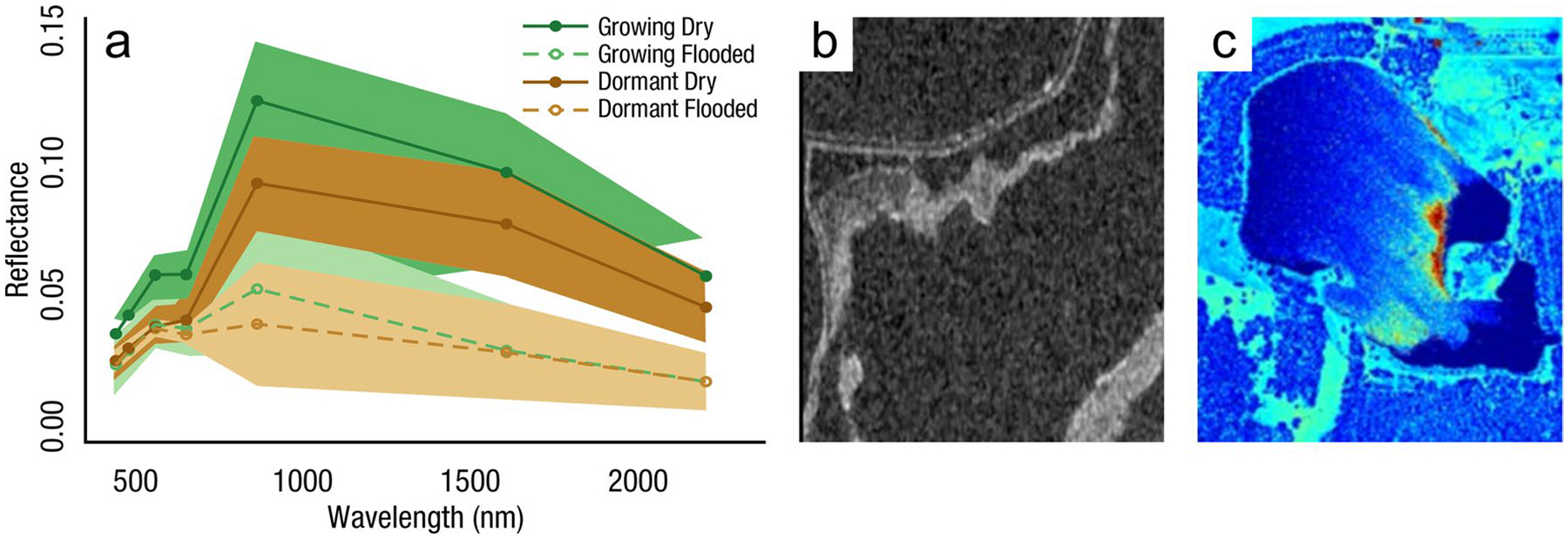
(**a**) Spectral reflectance during growing (green) and dormant (brown) seasons under flooded (dashed lines) and dry (solid lines) conditions (Narron et al. 2022). Growing season reflectance is overall higher than during the dormant season, but flooding attenuates light, lowering reflectance regardless of season, especially at longer wavelengths; (**b**) Sentinel-1 Ground Range Detected (GRD) C-Band Synthetic Aperture Radar (SAR) backscatter (VH polarization). Water areas show as dark regions, indicating high absorption and attenuation of the SAR signal, whereas lighter features indicate uplands (modified from Twele et al. 2016); (**c**) Light Detection and Ranging (LiDAR) intensity over a pond and surrounding upland. Blue to red indicates lower to higher intensity of LiDAR returns to the sensor, respectively. The pond mainly has very low intensity returns (very dark blue) and absorbs the LiDAR signal, but occasionally has very high intensity returns (red color) due to specular reflection off the water surface (modified from Acharjee et al. 2016). Image with permission from Venkat Devarajan (c)

*Where and When:* The spatial and temporal scale of inference for any sensor depends on its spatial, spectral, and temporal resolution, spatial extent, and timespan of operation (Campbell et al. 2022). For example, the Landsat satellite archive, a global remote sensing dataset often used for estimating wetland C pools and fluxes, is especially helpful for time series analysis as Landsat 4 and newer have high quality images dating back to the mid-1980s with a 30-m spatial resolution and an 8 to 16-day return interval. Other commonly used global remote sensing datasets include Sentinel-2 MultiSpectral Instrument data, which provide multispectral optical imagery at 10- to 60-m spatial resolutions, depending on wavelength, at 5-day intervals since 2015. Similarly, Sentinel-1 provides a SAR ground range detected product, which represents active radar data and can detect changes under cloud cover and tree canopy at day or night with a spatial resolution that varies from 10- to 40-m at a 6-day interval (Fig. 30b). Landsat, Sentinel-1, and Sentinel-2 datasets are all freely available (as of 2023); commercial satellite data may provide higher quality data, but may also be costly.

Coarser spatial resolution satellite products, such as Moderate Resolution Imaging Spectroradiometer (MODIS, pixel sizes range from 250–1,000 m) and Visible and Infrared Scanner (VIRS, pixel sizes starting at 0.75-km) imagery (freely available global data; launched in 2002 and 2011, respectively) are also useful for wetlands with high temporal variation because they provide multispectral, daily data and several derived land surface products such as surface temperature and GPP (O’Connell et al. 2017; NASA 2023). However, MODIS and VIRS may not be appropriate for smaller wetlands (e.g., < 500 m^2^) that are less than one (or a few) pixel(s) in size because these result in mixed-pixels and potentially low signal to noise detection. Vegetation-based NASA composite products, such as NDVI, GPP, and NPP, are calibrated for terrestrial systems, and therefore likely to have high error rates when used for wetlands. Specifically, vegetation-based products typically do not account for the reduction in spectral reflectance caused by water backgrounds or changes in ecosystem productivity caused by temporary to permanent flooding (Cho et al. 2008; O’Connell et al. 2017; Tao et al. 2018; Hawman et al. 2021; Narron et al. 2022). Other data sources, such as aerial imagery from the National Agriculture Imagery Program (NAIP) with 3 or 4 bands have much higher spatial resolution (often 1 m), but lower temporal resolution, as data-collection flights are typically performed only once a year at most. Accordingly, NAIP-derived surface water inundation products do not capture intra-annual variability in wetland inundation extent. Many other optical satellite missions exist, such as WorldView, Hyperion, IKONOS, SPOT, and GeoEye, each of which have trade-offs in terms of spatial, spectral, and temporal resolution, as well as cost and spatiotemporal scope. Kim et al. (2014) compared both multiresolution and spectral effects of three remote sensing sources (SPOT [10 m], Landsat [30 m], and MODIS [250 m]) using machine learning models (random forest) of total N and total P in a wetland in south Florida, USA. Results showed similar errors and model performances among sources, but the finer resolution images from SPOT characterized the variability of N and P with higher precision than coarser scaled Landsat and MODIS; similar results can be expected for soil C modeling.

*Who:* Modeling wetland C pools and fluxes using remotely sensed data is an interdisciplinary process that often requires knowledge of geographic information system (GIS), computer programming, physics, statistics, and geospatial analyses. In addition, remotely sensed data have very high data volume (gigabytes, terabytes, petabytes), and often require experience with, and access to, high performance computing. It is particularly useful if remote sensing experts also have subject matter expertise on the wetland C pool or flux of interest, and with the ground-truth data they are modeling. Despite the high level of training needed in this field, advancements in cloud computing, increased availability of satellite time series and UAS data, and the usage of cloud-based remote sensing platforms (e.g., Google Earth Engine, Gorelick et al. 2017) have increased the accessibility for non-specialists to manipulate and analyze thousands of satellite images instantly.

**Fig. 31:**
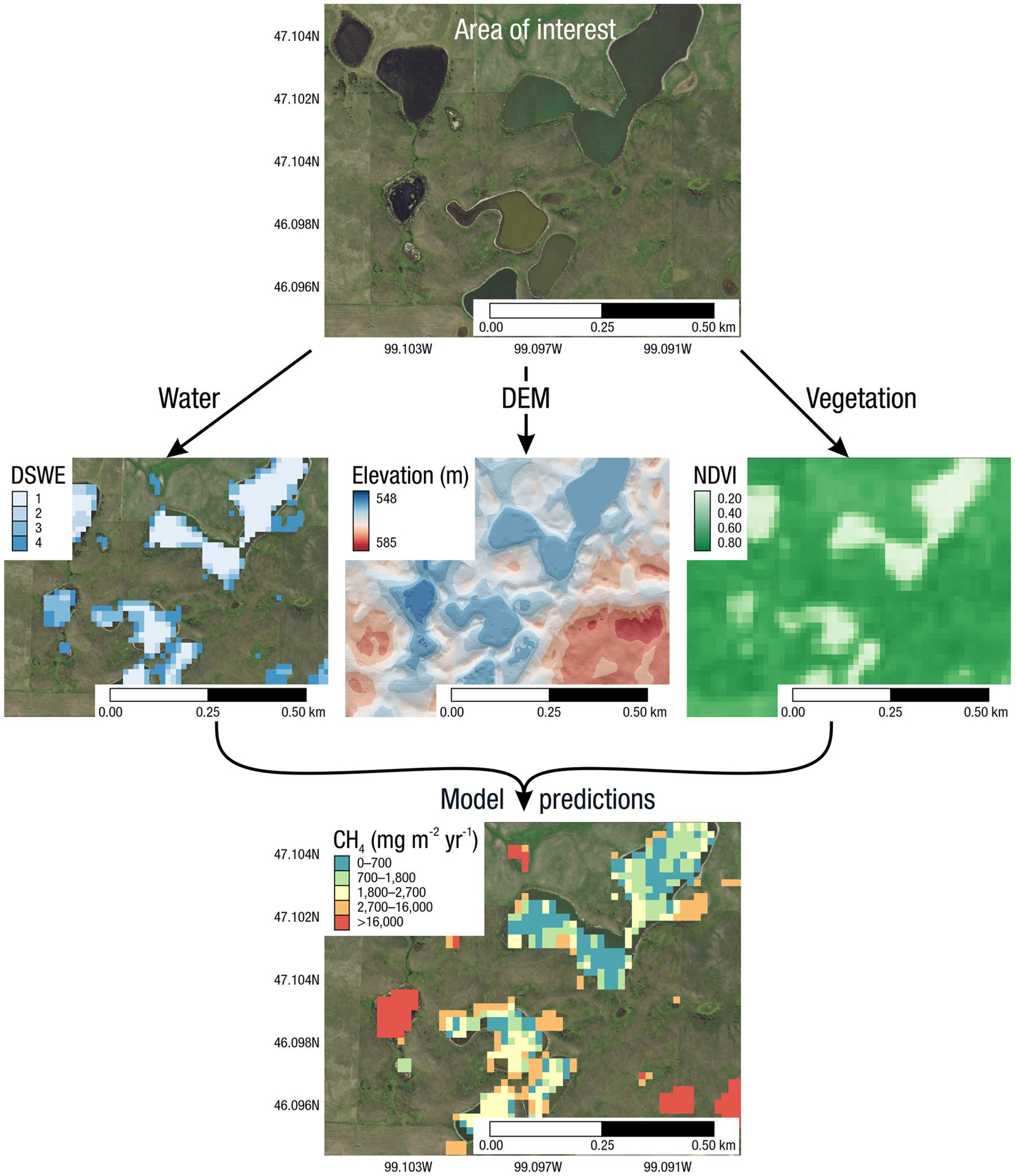
Example of a workflow to generate spatially explicit predictions (bottom) of wetland methane (CH_4_) fluxes using imagery (middle) over a defined area of interest (top). In this example, the area of interest is the Cottonwood Lake Study Area in North Dakota (USA), part of the Prairie Pothole Region of North America; (middle, left) Dynamic Surface Water Extent (DSWE) classification using Landsat imagery (Jones 2019); (middle, center) Digital Elevation Model (DEM) using Light Detection and Ranging (LiDAR); (middle right) Normalized Difference Vegetation Index (NDVI) using Landsat imagery; (bottom) growing season CH_4_ emissions based on Bansal et al. (2023). Areal images from National Agriculture Imagery Program (NAIP) Digital Ortho Photo Image (https://www.fisheries.noaa.gov/inport/item/49508)

*How:* Remote sensing can be used to describe a variety of wetland characteristics related to surface water extent and dynamics, vegetation, and soils, which are then used to model wetland C pools and fluxes (Fig. 31). Remote sensing C models are typically statistical, data-driven models that are trained against ground-based data; the ground-based data will ideally have GPS coordinates (down to 4 significant digits at least) and other metadata, such as weather conditions and time of day. The models are then used to estimate wetland C pools or fluxes at unsampled locations. It is best not to extrapolate models outside the field data range to avoid excessive uncertainties, and to use appropriate training, testing, and validation procedures when model building.

*Wetland hydrology:* Wetland surface-water dynamics, specifically the duration and extent of flooding, are important drivers of variability in aboveground and belowground wetland C pools and fluxes (Knox et al. 2021). The routine acquisition frequency and long-term archive of satellite imagery provides the ability to map surface-water dynamics through time (Halabisky et al. 2016; Zhang et al. 2021b). There are several existing surface-water datasets derived from the Landsat archive such as European Commission’s Joint Research Centre Global Water Dataset (JRC, Pekel et al. 2016), Dynamic Surface Water Extent (DSWE, Jones 2019), Harmonized Landsat and Setinel-2 surface reflectance dataset (HLS, Claverie et al. 2018), and Wetland Area and Dynamics for Methane Modeling (WAD2M, Zhang et al. 2021b). These datasets provide information of flood frequency and trends over time summarized at the pixel scale. The products rely on the principle that water rapidly attenuates electromagnetic radiation with increasing water depth and turbidity (Hossain et al. 2015b), attenuating longer wavelengths more rapidly than shorter wavelengths (Cho et al. 2008; Acharjee et al. 2016; Twele et al. 2016; Fig. 30a), with all wavelengths eventually approaching zero. Existing datasets do not always capture wetland surface water in situations where light is not completely attenuated, such as for small wetlands that are highly vegetated with emergent, floating, and submerged aquatic vegetation, shallow water or rapidly fluctuating tidal water, and turbid or chlorophyll-rich water.

In general, remotely sensed information at finer spatial resolution provides more spatial detail, but requires more storage space and processing time that may be beyond what is needed to meet study objectives and high-performance computational capacity. For example, a multi-scale machine learning model at increasing pixel resolutions in a subtropical wetland demonstrated that even coarser pixel resolutions can produce well performing wetland C models, indicating that a finer spatial resolution is not always needed to meet study objectives (Kim and Grunwald 2016). However, for many research questions, a pixel resolution of 30 m or more is too coarse for monitoring surface-water dynamics. In these cases, sub-pixel methods, sometimes trained by pixel aggregation (DeVries et al. 2017) or spectral mixture analysis (Halabisky et al. 2016), when combined with OBIA (Hondula et al. 2021), can provide reliable estimates of surface-water extent. These surface-water estimates can be used to reconstruct surface water time series that can be integrated with other datasets (e.g., in situ data, climate data) for understanding landscape scale wetland hydrological dynamics and for climate change modeling (Kissel et al. 2020).

*Wetland extent using LiDAR and DEMs:* Topographic data are widely used to identify depressions in the landscape where water tables are likely to be at or above the surface and, accordingly, where wetlands are likely to form. Automated GIS digital terrain analysis methods have been developed to map depressions from topographic data surfaces, referred to as digital elevation models (DEMs). DEMs can be created by interpolation of spatially dense spot height data (point clouds) obtained from airborne LiDAR surveys. LiDAR is an active remote sensor that maps features in three dimensions by emitting pulses of electromagnetic light are reflected back to the sensor to estimate relative elevation (Fig. 30c). These airborne systems are used to generate DEM grids with fine horizontal (e.g., 0.5 m) and vertical accuracies (e.g., < 10 cm), which permit mapping of very small wetland features. Models applied to LiDAR data can further distinguish between terrain elevation surfaces (derived from the ‘last return’ of LiDAR pulse) and canopy surface models (derived from the ‘first return’ of LiDAR pulse).

The high density of LiDAR pulses from airborne surveys are particularly useful to detect obscured wetlands underneath vegetation canopies (i.e., ‘cryptic’ wetlands; Creed et al. 2003; Lang et al. 2013). Several studies (Creed et al. 2003; Creed and Beall 2009) have used the Bayesian probabilistic distribution of elevation errors in DEMs to generate surfaces of depression probability (p_dep_) to classify pixels in wetland depressions (Planchon and Darboux 2002; Lindsay et al. 2004; Lindsay and Creed 2006; Webster et al. 2011). Serran and Creed (2016) further conducted pairwise clustering processes (i.e., creating homogeneous image objects by merging regions with similar color, smoothness, and compactness) in depressions to improve representation of wetland boundaries and capture the smallest wetlands. LiDAR data have also been used to estimate extent of saturated soils (or wet areas) by extracting topographic wetness indices (Beven and Kirkby 1979), elevation-above-stream (Rennó et al. 2008), and depth-to-water (Murphy et al. 2008). These indices can be used to map wet areas within wetlands (Lidberg et al. 2020), to find vegetated wetlands (e.g., Lang et al. 2013), to identify areas with hydrological connectivity (e.g., Creed and Beall 2009), and to map drainage features to identify restorable wetlands (Waz and Creed 2017). DEMs can also be interpolated from published topographic maps with a grid resolution (pixel spacing) that is constrained by the scale of the topographic maps. For example, Creed et al. (2008) used maps with 1:10,000 and 1:20,000 contour intervals to generate 10-m DEMs.

*Wetland vegetation:* The most common remote sensing derived wetland C pool is aboveground plant biomass, which typically is assessed from spectral indices such as NDVI. NDVI is calculated as a normalized ratio of the difference between NIR and red wavelength bands (Rouse et al. 1974):

(15) NDVI=(NIR−red)/(NIR+red).

Leaf chlorophyll absorbs red visible light while leaf structures reflect NIR. NIR reflection is related to leaf area and leaf water content, while red absorption increases with leaf chlorophyll content and is related to photosynthetic capacity. Plants with greater chlorophyll content and greater leaf area often have higher NDVI values and greater plant aboveground biomass. NDVI values over vegetation differ from other common land cover, such as soil (absorbs less red and reflects less NIR) or water (absorbs both red and NIR). Other spectral indices are also useful in wetlands, such as the Normalized Difference Water Index (NDWI), of which several derivatives exist (Ji et al. 2009; DeVries et al. 2017), or the Soil-Adjusted Vegetation Index (SAVI), which is often a high performing index in coastal wetlands (Byrd et al. 2018). Whichever index is used, models need to be trained against species- or community-specific ground-truth data to account for physical differences in leaf chemical composition and the light scattering properties of canopy structures. Imagery from automated multi-spectral cameras to capture time-lapse pictures of vegetation and the surrounding area can be used in conjunction with satellite-derived vegetation indices to model phenology (Vázquez-Lule and Vargas 2021).

It is important to understand how physical features, such as the presence and depth of water, interact with vegetation indices. For example, shallow water will attenuate the longer NIR wavelength more than visible light, which reduces NDVI, even when vegetation canopies are similar (Fig. 30a). Thus, NDVI cannot be directly compared across water depths, and modeling wetland vegetation requires information on water depth and extent as part of the vegetation estimation protocol (Byrd et al. 2014; O’Connell et al. 2017, 2021). For example, O’Connell et al. (2021) first estimate pixel flooding dynamics to account for water’s spectral reflectance and plant physiological variation across flooded and dry pixels, and then used machine learning methods to estimate aboveground biophysical proxies and belowground biomass. Remote sensing of aboveground vegetation may also need to account for spatial variation in thatch (built up dead grasses) as a model covariate if it covers emergent vegetation (Byrd et al. 2018).

Remotely sensed data are often used in biophysical, plant-centric models of GPP and NPP in terrestrial systems, which have been adapted to wetland habitats. An example is the production efficiency model, which assumes a linear relationship between vegetation productivity (GPP or NPP), the fraction of photosynthetically active radiation (PAR) intercepted, and a constant biome-specific light use efficiency (LUE) parameter (Monteith 1972; Monteith et al. 1977; Kumar and Monteith 1981; Ruimy et al. 1994; Stuart-Haëntjens et al. 2015). These biophysical models are used to estimate local to global scale plant production across many types of biomes (Turner et al. 2006; Zhang et al. 2009). However, biome-specific LUE values are typically not available for wetlands (Running et al. 2004; Running and Zhao 2015; Tao et al. 2018) and likely differ from terrestrial systems due to differences in hydrology and vegetation (Hawman et al. 2021). LUE and production efficiency can be estimated from EC flux towers (Yan et al. 2008; Barr et al. 2013; Tao et al. 2018; Feagin et al. 2020), though more work is needed to derive LUE across the range of wetland conditions (Hawman et al. 2021).

Similar to production efficiency models, chlorophyll production models are another method to estimate GPP from remotely sensed chlorophyll (Gitelson et al. 2003, 2006). Chlorophyll is a good indicator of GPP because it fluctuates with phenology, stress, and the photosynthetic capacity of vegetation. Gitelson et al. (2003) and Gitelson et al. (2006) estimated GPP through consistent (not species specific) relationships between chlorophyll and GPP. Indices of Chl-*a* in standing water can also be determined using satellite imagery (Seegers et al. 2021), which is important for detecting harmful algal blooms.

Another approach for estimating aboveground biomass is through the use of allometric relationships with canopy height and structure (Simard et al. 2008). For example, forest canopy height can be estimated with sensor such as IceSAT, SRTM, GLAS, or TanDEM-X, and then used to model tree biomass through the use of generalized or site-specific allometric relationships (e.g., Lagomasino et al. 2016). LiDAR provides a high-resolution approach for developing allometric equations for tree and emergent biomass (Cook et al. 2009; Babcock et al. 2018; Owers et al. 2018; Stovall et al. 2018). LiDAR can also be used to link fine-scale features within a wetland, such as surface micro-topography, to structural attributes of the vegetation (e.g., leaf area index, Luo et al. 2015). Stovall et al. (2019) used LiDAR to characterize the relief and distribution of hummocks and hollows in a forested wetland, demonstrating how considering small-scale variation in surface elevation can control C dynamics.

*Wetland soil properties:* The use of remote sensing applications to assess the distribution and properties of soils (in particular C content) is challenging, but advances in sensor technology have enhanced the capability and resolution for discerning soil properties (see synthesis by Wulf et al. 2015). The spectral signature of soil color and certain soil properties can be measured from passive, multispectral sensors, especially when the sensor measures portions of the electromagnetic spectrum outside of the visible light (e.g., NIR) (Mulder et al. 2011). However, passive sensors cannot measure soil below the soil surface, hence their application typically involves a statistical relationship between the surface optical and physical data and the soil attribute. Similarly, passive sensors cannot assess soil surfaces covered by vegetation, thereby further constraining applications. Active sensors, such as ground penetrating radar and SAR can non-invasively penetrate through vegetation and surface soils to assess subsurface soil properties and basin morphology (Comas et al. 2004; McClellan et al. 2017). For example, ground penetrating radar has been used in conjunction with traditional core sampling to estimate organic soil C pools of subtropical and tropical peatlands (Comas et al. 2015; McClellan et al. 2017) and in boreal peatlands (Comas et al. 2004).

*Atmospheric inverse modeling:* Atmospheric inverse models provide a ‘top-down’ perspective on large-scale surface-atmosphere exchange patterns. They are valuable for evaluating ‘bottom-up’, data-driven, or process-based upscaling models from a top-down perspective, and therefore contribute to reducing uncertainties associated with extrapolation and constraining net wetland C budgets over longer timescales (Saunois et al. 2020a, b; Munassar et al. 2022). As opposed to bottom-up analysis, many details within the system (e.g., environmental drivers, spatial heterogeneity) are generally treated as a ‘black box’. Therefore, inversion results are not suitable for constraining fluxes at specific wetland sites, and can only provide limited insights into processes and controls that determine C flux variability. Even so, the capability to produce data-driven, large-scale flux estimates make atmospheric inversions a powerful tool for long-term monitoring of GHG emissions (Rödenbeck et al. 2022) at national to continental scales (Villalobos et al. 2021; Chen et al. 2022), and for providing an independent reference to evaluate scaled up chamber or EC flux products or gridded process-based model simulations (Deng et al. 2022).

Atmospheric inverse modeling (e.g., Enting 2002; Yu et al. 2021) constrains regional (~ 400 km^2^, e.g., Gerbig et al. 2003) to global scale (e.g., Gurney et al. 2002) surface-atmosphere gas exchange processes based on observations of atmospheric trace gas mixing ratios (e.g., from towers, aircraft, or satellites). The technique uses atmospheric transport modeling (Lin et al. 2003) to link atmospheric observations of GHGs to their respective sources on the ground. Subsequently, statistical approaches such as Bayesian optimization (e.g., Tarantola 1987; Enting 2002) or Kalman filters (e.g., Peters et al. 2007) are used to identify the spatial and temporal flux field that agrees best with the atmospheric observations. Operation of atmospheric transport models, assigning and weighing uncertainties for different assimilated datasets, and the operation of atmospheric mixing ratio observations all need to be carefully calibrated against international standards for this application, all require expert knowledge that cannot be easily acquired.

Atmospheric inverse modeling requires a spatially dense network of highly calibrated atmospheric mixing ratio measurements (Shiga et al. 2013). To reliably constrain surface flux fields one either has to choose a very low spatial and temporal resolution or assimilate additional data to constrain models (Michalak et al. 2004), since available data are relatively sparse in most regions of the globe. A common approach is to provide prior flux fields from data-driven upscaled GHG flux data or process-based models as a ‘best guess’ starting point. These initial fluxes will then be ‘nudged’ towards a version that agrees best with the atmospheric mixing ratio observations by considering the various uncertainty sources such as transport and mixing errors, measurement errors in fluxes and mixing ratios, and aggregation errors (Gerbig et al. 2003; Andrews et al. 2014).

To specifically target surface GHG fluxes from wetlands (Miller et al. 2016), a key requirement of atmospheric inverse modeling is high quality maps of wetland area and type, ideally including seasonal and interannual variability in wetland extent or ponded area (Sheng et al. 2018; Zhang et al. 2021c). In addition, wetland-specific prior GHG flux rates are required. Since wetlands are often highly structured and integrated into heterogeneous landscapes that usually contain multiple other land cover types, constraining wetland-specific GHG fluxes through atmospheric inversions will be most accurate when operating the model at fine spatial resolution (Tan et al. 2016), which usually requires a regionally distributed data collection plan. However, while smaller modeling domains allow the use of a finer grid resolution, they also require a dataset of boundary conditions to resolve the effect of GHG mixing ratio variability within air masses entering the domain, adding an additional source of uncertainty.

## Conclusion

This review article on methods to measure wetland C pools and fluxes, while extensive, mainly provides general, practical guidance for investigators to consider when planning field campaigns, prior to taking measurements. We advocate that investigators read the source literature, much of which (but not all) is cited in this article, for any wetland C pool or flux that is under consideration for measurement. It is always ideal to make major decisions, such as how the data will be used, prior to conducting data collection. For example, data intended for use in a ‘community-contributed’ dataset must adhere to specific metadata requirements. During the process of mining the literature, we strongly suggest taking note of article-specific definitions of various C pools and fluxes, as many terms and acronyms are used synonymously. Also take note of C pool or flux values (with units for later conversion to a common metric) from relevant articles to become familiar with the expected range of values, which will help determine if measured values ‘make sense’ and are relatively high or low in comparison to other systems; large, ‘structured’ datasets can facilitate comparisons between observed and expected values. However, some wetland C pools and fluxes, such as CH_4_ emission via plant or ebullitive pathways, have limited published values for comparisons, potentially indicating a scientific research gap. In this review we provide examples of many scientific research gaps, many of which exist due to methodological challenges that require time, personnel, and funding. Yet, in many cases, creativity and ‘thinking outside the box’ can overcome methodological challenges. We urge that novel solutions, both those provided here and those you may develop, be transferred to the larger scientific community for others to use, and to avoid ‘reinventing the wheel’.

We covered common and cutting-edge methodological approaches, but there are additional details for each approach and many alternative approaches that were not included. Moreover, the discipline of wetland C cycling is continually evolving new methods to assess wetland C. Therefore, we encourage investigators learn from previous work, while also keeping an open mind for new methods to better measure wetland C pools and fluxes. The better we understand the mechanistic processes driving rates of C uptake and loss from wetlands, the better we can model C dynamics and manage wetlands to optimize climate benefits (i.e., radiative cooling) while maintaining or increasing co-benefit ecosystem services from wetlands such as wildlife habitat, nutrient retention, and flood mitigation.

## Data Availability

No previously unpublished data were used in this article. Figure 1 map was generated using publicly available data from Zhang et al. (2021c; https://doi.org/10.5194/essd-13-2001-2021). Figure 31 was generated using publicly available data from Bansal et al. (2023; https://doi.org/10.5066/P9PKI29C). All other data figures are cited from previously published articles with permissions.

## Supplementary Material

Supplement1

## Figures and Tables

**Table 1:** List of common acronyms and definitions used to abbreviate various methodologies, carbon pools, and carbon fluxes in this review and the wetland scientific literature. Note that some acronyms, like SAR, are used to abbreviate more than one phrase in the literature, therefore it important to check for source-specific definitions

Acronym	Definition	Acronym	Definition	Acronym	Definition
aCAR	apparent rate of carbon accumulation	FDOM	fluorescent dissolved organic matter	PCR	polymerase chain reactions
AMS	accelerator mass spectrometry	GHG	greenhouse gas	PLFA	phospholipid fatty acid
aNPP	aboveground net primary productivity	GPP	gross primary productivity	PIC	particulate inorganic carbon
AOM	anaerobic oxidation of methane	GWP	global warming potential	POC	particulate organic carbon
bNPP	belowground net primary productivity	k_600_	standard gas exchange coefficient	POM	particulate organic matter
CAR	carbon accumulation rate	LAI	leaf area index	PPFD	photosynthetic photon flux density
CDOM	colored dissolved organic matter	LiDAR	light detection and ranging	R_A_	autotrophic respiration
Chl-*a*	chlorophyll-*a*	LOI	loss-on-ignition	redox	reduction–oxidation
CHN	carbon-hydrogen–nitrogen	LUE	light use efficiency	R_H_	heterotrophic respiration
CO_2(aq)_	aqueous or dissolved carbon dioxide	LULC	land use and land cover	RTK GPS	Real-time kinematic global positioning system
CO_2-eq_	carbon dioxide-equivalent unit	MAR	mass accumulation rate	SAR	sediment accretion rate
dbh	diameter at breast height	MIMS	membrane inlet mass spectrometry	SET	surface elevation table
DEM	digital elevation model	NDVI	Normalized Difference Vegetation Index	SGWP	sustained global warming potential
DIC	dissolved inorganic carbon	NECB	net ecosystem carbon balance	SOC	soil organic carbon
DO	dissolved oxygen	NEE	net ecosystem exchange	SOM	soil organic matter
DOC	dissolved organic carbon	NEP	net ecosystem production	SUVA_254_	specific ultra-violet absorbance at 254 nm
DOM	dissolved organic matter	NMR	nuclear magnetic resonance	TBCA	total belowground carbon allocation
EC	eddy covariance	NPP	net primary productivity	TOC	total organic carbon
E_h_	electrical potential	OBIA	object-based image analysis	TSS	total suspended solids
ER	ecosystem respiration, often abbreviated as ‘RECO’				
PAR	photosynthetically active radiation				

**Table 2:** Here we provide a list of syntheses, meta-analyses, and compilations of wetland carbon pools and fluxes as resources for researchers to use as a priori information and for post hoc comparisons to other studies. [C, carbon; CH_4_, methane; CO_2_, carbon dioxide; GHG, greenhouse gas; GPP, gross primary production; N, nitrogen; NEE, net ecosystem exchange; NEP, net ecosystem production; NPP, net primary productivity; N_2_O, nitrous oxide; SOC, soil organic carbon]

Pools and/or flux	Additional information	Wetland type	Location	Reference
Aboveground and below-ground biomass, NPP Smalley method review		All herbaceous	United States	Stagg et al. (2017b)
Aboveground and below-ground biomass, soil C pool	Wetland area	Mangrove, Salt Marsh	Global	Alongi (2020)
Aboveground and below-ground lignin content		All herbaceous	United States	Stagg et al. (2018)
Blue C pools and GHG fluxes		Coastal	Global	O’Connor et al. (2020)
C accumulation		Freshwater marshes	North America	Loder and Finkelstein (2020)
C pools and fluxes	CH_4_, C accumulation	Natural and constructed freshwater wetlands	Global	Kayranli et al. (2010)
C pools and fluxes; NEE and CH_4_ fluxes	Wetland area	Terrestrial wetlands	North America	Kolka et al. (2018)
C pools, accumulation rates, NEE and CH_4_, GPP, NEP, lateral export	CH_4_-salinity relationships, wetland area	Tidal wetlands and estuaries	North America	Windham-Myers et al. (2018), Feagin et al. (2020)
C sequestration		Tidal, saline wetlands	Global	Chmura et al. (2003)
C sequestration	Climate, nutrients	All	Global	Cheng et al. (2020)
C uptake and emission	Wetland area	All	China	Xiao et al. (2019)
CH_4_ emissions		Tropical freshwater wetlands	Costa Rica	Nahlik and Mitsch (2011)
CH_4_ emissions		Created and natural freshwater wetlands	Ohio, United States	Nahlik and Mitsch (2010)
CH_4_ flux		All	Subtropical, temperate, and northern high latitude regions	Turetsky et al. (2014)
CH_4_ flux	Water table depth	Tropical peat swamps	Southeast Asia	Hergoualc’h and Verchot (2012)
CH_4_ flux		All	Global	Saunois et al. (2020a)
Eddy covariance CH_4_ flux	Non-wetland CH_4_ flux	All	Global	Knox et al. (2019) Delwiche et al. (2021)
Eddy covariance CO_2_ flux	Water table depth	Bogs and fens	North America	Sulman et al. (2010)
Eddy covariance CO_2_ flux		Inland and coastal wetlands	Global	Lu et al. (2017)
GHG emissions		Coastal and riparian wetlands, and peatlands	Global	Tan et al. (2020)
GHG emissions		Reservoirs	Global	Deemer et al. (2016),
Long-term soil C pools and accumulation rates	N pool and accumulation, dry bulk density	Peatlands	North of 45°N	Loisel et al. (2014)
Modern organic C accumulation rates		Coastal and aquatic inland ecosystems	Global	Wilkinson et al. (2018)
Organic C pool	Wetland area, CH_4_	All	Global	Mitra et al. (2005)
SOC		Restored wetlands	Global	Xu et al. (2019b)
SOC		Tropics and permafrost	Global	Köchy et al. (2015)
SOC pools and rates in temperate and tropical wetlands		Freshwater, temperate and tropical wetlands	United States, Costa Rica	Bernal and Mitsch (2008)
SOC pools and rates in temperate wetlands	Dry bulk density	Freshwater, temperate wetlands	Ohio, United States	Bernal and Mitsch (2012)
Soil C accumulation rates		Mangroves	Global	Breithaupt et al. (2012), Breithaupt and Steinmuller (2022)
Soil C accumulation rates		Coastal marshes	Global	Ouyang and Lee (2014)
Soil C pools	Soil N	Restored and created wetland	United States	Yu et al. (2017)
Soil C pools		Freshwater inland, tidal estuarine	Conterminous United States	Nahlik and Fennessy (2016)
Soil C pools		Freshwater inland and tidal wetlands	United States	Uhran et al. (2021), Wardrup et al. (2021)
Soil C pools and fluxes, plant biomass, NPP, CH_4_ fluxes	Wetland area	All	North America	Bridgham et al. (2006)
Soil C pools, aboveground C pools, ecosystem C exchange, soil C burial, lateral flux estimation	Bonafide blue carbon wetland type, high C stock and flux potential	Tidal freshwater forested wetland and oligohaline marsh	United States	Krauss et al. (2018b)

**Table 3:** Definitions of terms commonly used when describing carbon (C) in wetland soils. Note: Some terms are often used synonymously, such as ‘soil’ and ‘sediment’ or ‘accretion’ and ‘accumulation’, therefore it is important to check how terms are specifically defined (see Sections “Carbon in Wetland Soils” and “Carbon Accumulation in Wetland Soil” for additional discussion on terminology)

Term	Definition	Units	Reference
Allochthonous C	C that is produced in one location, transported, then deposited in another, reflecting ex situ production	g C	Howard et al. (2014), Van de Broek et al. (2018), Windham-Myers et al. (2019)
Autochthonous C	C that is produced then deposited in the same location, reflecting in situ production	g C	Howard et al. (2014), Van de Broek et al. (2018), Windham-Myers et al. (2019)
Blue carbon	C accumulated in the sediment and biomass of vegetated coastal ecosystems influenced by sea level and tidal dynamics such as mangroves, freshwater tidal forests, tidal marshes, and seagrass meadows	g C	Nellemann et al. (2009), Howard et al. (2014), Krauss et al. (2018b), Windham-Myers et al. (2019)
C accumulation rate (CAR); apparent rate of C accumulation (aCAR)	Rate of C build up over a specified area and timeframe. CAR is not a direct measurement of C deposition only because it includes both C inputs and losses through decomposition. Therefore, CAR is sometimes referred to as the apparent rate of C accumulation (aCAR)	g C m^−2^ yr^−1^	Clymo (1984), Tolonen and Turunen (1996), Charman (2002), Yu (2011), Packalen and Finkelstein (2014)
C burial	Generic term that can have more than one definition, referring to CAR over decadal or longer time scales	g C m^−2^ yr^−1^	
C content (or concentration)	Proportion of sediment dry mass that is comprised of C. Usually expressed as either a percent or per unit mass of soil. Inorganic versus organic C fractions are sometimes separated	%; g C kg^−1^ soil	Mitsch and Gosselink (2015)
C density	Proportion of dry bulk density consisting of C; calculated as product of dry bulk density and C content and reported on a volumetric basis	g C cm^−3^	Howard et al. (2014)
C pool	Snapshot quantity of C in soils, vegetation, or water within a given area and time	g C m^−2^, Mg C ha^−1^	Ciais et al. (2014), Howard et al. (2014), Windham-Myers et al. (2019)
C reservoir	Subsystems that store C and support C transformations (e.g., soil, water, vegetation, atmosphere)		Ciais et al. (2014), Howard et al. (2014), Windham-Myers et al. (2019)
C sequestration	Generic term that can have more than one definition, referring to the removal and accumulation of C on recent or longer timescales in wetland soils and vegetation. This C may be removed from the atmosphere as carbon dioxide via photosynthesis or transferred from another C reservoir. Organic versus inorganic C may be specified	g C m^−2^ yr^−1^	Kayranli et al. (2010), Mitsch et al. (2013), Van de Broek et al. (2018)
C stock	This review: the sum of soil, water, and vegetation C pools. Note: the term ‘stock’ and ‘pool’ used synonymously in the literature	g C m^−2^; Mg Cha^−1^	Windham-Myers et al. (2019)
C storage	Generic term that can have more than one definition, referring to the longterm soil C pool		
Cumulative dry mass, Mass-depth	Mass of dry soil per unit ground area. For this measure, depth varies such that each sample contains the same dry mass per unit ground area	g cm^−2^	Gifford and Roderick (2003), Wendt and Hauser (2013)
Dry bulk density	Soil density measured as dried soil sample divided by its original (in situ) volume	g cm^−3^	Howard et al. (2014)
Equivalent Soil Mass	Mass of soil in a standard or reference surface layer	Mg ha^−1^	Ellert and Bettany (1995), Wendt and Hauser (2013), Fowler et al. (2023)
Long-term rate of C accumulation (LORCA)	Soil C accumulation rates over longer timeframes (e.g., > 100 years)	g C m^−2^ yr^−1^	Tolonen and Turunen (1996), (Rydin and Jeglum 2006), Young et al. (2019), Loder and Finkelstein (2020), (Loder and Finkelstein 2020)
Mass accumulation rate (MAR)	Accumulated mass of dry soil per unit ground area over a certain time interval	g cm^−2^ yr^−1^	Appleby (2002), Abril (2003)
Peat	Deposits that are predominantly composed of partially decomposed in situ plant material and have high water and organic C contents. Often defined as > 65% organic matter		Charman (2002), Hiraishi et al. (2014), Mitsch and Gosselink (2015)
Recent/short-term rate of C accumulation (RERCA)	Soil C accumulation rates over the last 50 to 100 years	g C m^−2^ yr^−1^	Tolonen and Turunen (1996), Rydin and Jeglum (2006), Young et al. (2019), Loder and Finkelstein (2020)
Sediment accretion rate (SAR, also referred to as ‘sedimentation rate’)	Net total sediment deposition (inorganic and organic) in wetland soils that can be measured as vertical change per unit time	mm yr^−1^	Thomas and Ridd (2004), Drexler (2011)
Soil inorganic C	C of mineral origin including precipitates (i.e., calcretes) and biogenic carbonate (i.e., shells) and dissolved CO_2_	%, g	Howard et al. (2014), Windham-Myers et al. (2019)
Soil organic C (SOC)	C of biotic origin (i.e., plant, animal, microbial material)	%, g	Reddy and DeLaune (2008)
Soil organic matter (SOM)	Un- or partially decayed organic constituents (e.g., plant, animal, and microbial material) in the soil	%, g	Howard et al. (2014)
Teal C	C accumulated in the sediments and in vegetation biomass of inland freshwater wetlands		Nahlik and Fennessy (2016)
Total soil C pool	Total mass of soil organic and inorganic C in defined areas; calculated as the product of the C pool (mass per unit area) and total areal extent	Pg, Tg	Yu et al. (2010), Howard et al. (2014), Packalen et al. (2014)
Vertical accretion rate	Net rate of vertical (elevation) change (including both gains and losses) in sediment thickness	mm yr^−1^	Eagle et al. (2022)

**Table 4:** Examples and descriptions of commonly used coring devices, arranged from the simplest (handheld) to the most complex (machine powered) designs. While this list captures the range of types of coring devices used in wetland soils, it is not exhaustive

Coring device	Description	Soil type	Pros	Cons	References
Open barrel corer	There are many types of open barrel corers, both commercially available and homemade, as this is a generic term for a cylinder that is open at both the top and bottom The bottom edge of the cylinder is typically sharpened Cylinder may be solid or split (vertically) and may include a liner or core catcher Larger diameter cores can be equipped with plastic sleaves to allow visual inspection and freezing/slicing. Larger diameter core can also lower compaction	Ideal for non-fibrous sediment including in organic-rich to dense silt- and clay- or sandy sediments	Inexpensive, simple, and rapid method of recovering cores Several cores can be collected on the same day and kept intact until processing	Soil compaction may occur, causing overestimates of bulk density Dry bulk density corrections are needed	Reinhardt et al. (2000), Caldwell et al. (2005), Besonen (2012), Giannopoulos et al. (2019), Fourqurean et al. (2012)
Gouge auger	Steel cylinder auger with approximately 1/3 of the barrel open such that the barrel is ‘C’ shaped in cross section Includes a sharpened cutting edge and a T-handle Small or large diameter in shallow versus deeper depths, respectively It is inserted without twisting so that one face of the core remains intact, minimizing compaction Different models are commercially available in various diameters and lengths 1-m extension rods are available, allowing sampling up to 3 m	Soft and saturated soils. Highly organic soils to any soil with limited amounts of clay and with few large woody debris pieces	Portable, relatively light-weight, and easiest to travel with among the deeper coring devices Minimizes compaction Inexpensive, simple, and rapid method of recovering cores	Extension rods available, but intact soil collection difficult when deeper than 1 m Not appropriate for fluidic soils	Fourqurean et al. (2015), Fernández-Ugalde et al. (2020)
Modified Hoffer probe	Serrated steel coring bit with a T-handle Includes 1-m extension rods allowing sampling up to 5 m	Used mainly for coring frozen peat and stone-free, frozen, fine-textured soils	Portable, reliable, light weight	May be difficult to sample to great depths, requiring two people for depths greater than 4 m Shattering and compaction can occur	Brown (1968), Zoltai (1978)
Macauley peat corer (also known as Russian peat corer)	Semi-cylinder with rotating blade	Peat, organic sediment, and fluid mineral soils	Portable Extension rods allow collection of long sequences (≥ 5–10 m) Minimal compaction	Not suitable for stiff, mineral-rich sediments	Jowsey (1966), Franzén and Ljung (2009)
Box-style corers (i.e., Wardenaar)	Rectangular ‘box’ with sharp edges that can be oscillated to penetrate peat	Fibrous peat	Large, uncompressed surface peat monoliths	Portable, but relatively heavy Only collects top 50 to 100 cm May require a separate bulk density core/subsample	Wardenaar (1987)
Piston corer (also referred to as triple corer)	Stainless-steel or polyvinyl chloride (PVC) barrel with a moveable piston that can be locked into place, providing the suction to keep the core in the barrel as it is extracted Could include a core catcher and coring platform for open water coring	Ideal for non-fibrous sediment (marshes, emergent wetlands with standing water)	Extension rods can be added allowing extraction of long cores (5–10 or more m) Cores are extruded on site into rigid PVC tubing facilitating transport	Requires team of people and flat surface for extruding Can be portable, but difficult to carry/transport to remote sites	Livingstone (1955), Wright (1967), Kemp et al. (1971), Fisher et al. (1992), Sansone et al. (1994)
Hargis corer	Hand-operated coring device composed of an acrylic plastic cylinder, a removable cutting head, a piston with a T-handle, and rubber caps. The piston is positioned at the soil surface and is pulled upward as the coring device is pushed downward	Ideal for fibrous soils (e.g., root mats) with high porosity, high organic matter content	Can be operated by one person Minimal compaction Cuts through plant tissues The cutting head can be removed and used on another cylinder	Only collects top 50 cm Not suitable for stiff sediments	Hargis and Twilley (1994)
The core-freezer (also referred to as the frozen finger)	In situ frozen coring using a brass or copper tube surrounded by a jacket with crushed solid carbon dioxide and n-butyl alcohol, ethyl alcohol, or ethanol	Very soft sediment where the upper flocculated sediment needs to be captured intact	Allows for in situ, intact coring in very soft sediments and those covered by even deep water Preservation of deoxyribonucleic acid (DNA) and ribonucleic acid (RNA)	Slight distortion of the top centimeter of sediment due to freezing with conical concavity Heavy Not suitable for difficult to penetrate soils	Shapiro (1958)
Cryogenic coring device	Involves in situ freezing of a sediment sample, followed by removal of the sediment sample in its frozen state	Highly porous soils, or containing gases that can disrupt the soils during coring and recovery	Eliminates compaction, dewatering, and loss of flocculent material at the water–sediment interface Preservation of DNA and RNA	Needs liquid nitrogen or dry ice to be transported Samples must be processed rapidly and kept frozen	Knaus and Cahoon (1990)
Percussion corer	Set up with ladder or tripod and sledge-hammer to insert core tube which is fit with a core catcher and serrated or sharpened lower edge. Core tube can be extracted using tripod-mounted winch	Ideal for stiff, difficult to penetrate sediments	Facilitates access to sediments that are difficult to core using other means	Risk of compaction with the percussion action Limits on overall depth possible set by core tube length and ladder or tripod height	Gilbert and Glew (1985), Reasoner (1993)
Vibra-corer	Trailer- or track-mounted gasoline powered, vibrating corer, or smaller, battery powered device	Organic soils; in harder soils, high-powered gasoline models are needed	High-powered gasoline machines facilitate access to sediments that are difficult to core using other means	Risk of compaction Road access generally required Gasoline or other power supply required Battery powered models cannot penetrate hard soils	Finkelstein and Prins (1981)
Snow, Ice, and Permafrost Research Establishment corer (SIPRE)	Gasoline-powered drill with diamond-tipped drill bit	frozen sediment or peats	Allows for coring of frozen soils Can core several meters deep	Does not work on unfrozen soft sediment or peat Heavy, gasoline powered	Hughes and Terasmae (1963), Rand and Mellor (1985)

**Table 5:** Advantages and disadvantages of methods and associated equipment for collecting surface water, porewater and/or groundwater

Equipment	Advantages	Disadvantages
Bottle	Rapid collection of surface water	Restricted to ‘arms reach’ or extension pole length
Suction tube	Collect surface water at specific depths in the water column	Suction artifacts
Van Dorn sampler	Collection of all water carbon (C) constituents at specific depths No advective pressure	Can be relatively costly
Peepers	Limited potential for artifacts Collect samples from different depths at the same time	Require days to weeks for equilibration Some membranes are subject to biofouling or rupture Ideally deoxygenate chamber and materials to prevent oxidation effects Placement and retrieval challenges Clogging in clay-rich soils
Piezometer and water-table wells	Relatively larger sample volume Can obtain deeper samples Can obtain samples from multiple depths	May cause advection, water movement, and pressure change Greater installation effort and equipment costs
Tension lysimeters and sippers	Rapid collection Collect samples from specific depths	May cause advection, water movement, and pressure change Low sample volume Clogging in clay-rich soils
Squeezing and centrifugation	Extracts water from micropores and macropores	Specialized equipment required Potential overestimation of C in water

**Table 6:** Definitions and units of terms commonly used when describing carbon in wetland vegetation

Term	Definition	Common units	Reference
Net biomass production	The dry weight (g) of the organic matter (including flowers, fruits, leaves, twigs, branches, stems, roots) produced over a given time interval	g m^−2^ yr^−1^	Brinson et al. (1981), Cronk and Fennessy (2001)
Peak biomass production	The maximum net biomass produced (dry weight), typically reported for a growing season	g m^−2^ yr^−1^	Wiegert and Evans (1964), Cronk and Fennessy (2001)
Ash-free dry weight	The weight of plant material after combustion at 550 °C to determine the organic C content of plant tissues as the difference between dried plant matter and ash-free dry weight	g	Weil et al. (2019)
Primary productivity	Biosynthesis of organic biomass over time	g C m^−2^ yr^−1^	Brinson et al. (1981), Cronk and Fennessy (2001)
Primary production	Organic biomass produced over a given area	g C m^−2^	Brinson et al. (1981), Cronk and Fennessy (2001)
Gross primary productivity (GPP)	The sum of photosynthetic C gain *without* inclusion of losses due to autotrophic respiration, mortality, and predation	g C m^−2^ yr^−1^	Brinson et al. (1981), Cronk and Fennessy (2001)
Net primary productivity (NPP)	The change in the pool of plant organic matter over time. NPP is equivalent to GPP minus all losses of C, including to autotrophic respiration and biomass loss. Often specified as aboveground or belowground	g m^−2^ yr^−1^	Brinson et al. (1981), Cronk and Fennessy (2001), Khan et al. (2009)
Turnover rate	The biomass lost over a growing season (leaf loss, herbivory, etc.), expressed as an annual biomass turnover rate. Can be used to correct measures of peak biomass	g m^−2^ yr^−1^	Margalef (1963), Dickerman et al. (1986), Morris and Haskin (1990), From et al. (2021)
Longevity	The amount of time it takes for new biomass growth to replace standing biomass. Equivalent to 1 divided by the turnover rate	yr	Cormier et al. (2015), From et al. (2021)
Leaf area index (LAI)	Surface area of leaves or whole plants, normalized for ground area	Leaf area per unit ground area; m^2^ m^−2^	Williams et al. (2017)

**Table 7:** Comparison of methods for estimating soil carbon (C) accumulation. Asterisks (*) denote sediment accretion rate (SAR) methods, to which a C content conversion factor is applied to determine C accumulation rate (CAR)

Method	Description	Temporal scale	Pros	Cons	References
Sediment traps, filters, plates*	Various methods that passively collect surface-deposited materials over a specified area and time frame	Days/Event – years	Inexpensive; high precision; potential for high temporal resolution	Likely to overestimate material retention; may alter local hydrodynamics; bias against materials larger than trap aperture (e.g., coarse litter); sensitive to disturbance; sensitive to collection vessel design; C content of deposited material needs to be estimated or measured	Reed (1989), Nolte et al. (2013), Kelleway et al. (2017)
Marker horizons*	Change in sediment C pool above a placed marker horizon (e.g., feldspar)	Months – years	Inexpensive; can be paired with surface elevation tables, or used in isolation	May overestimate long-term CAR; Excludes subsurface processes; only measures net accumulation and not net erosion; C content of deposited material needs to be estimated or measured	Noe et al. (2016)
Reference benchmarks: dendrogeo-morphology*	Change in sediment C pool compared to a natural benchmark of woody vegetation of known or measured age	Years – decades	Inexpensive; can capture both positive (sedimentation) and negative (erosion) fluxes; can capture both vertical and lateral fluxes	Annual growth ring measurement requires specialized training; C content of deposited or eroded material needs to be estimated or measured	Noe et al. (2022)
Reference benchmarks: sediment pins or repeat surveying*	A network of shallow pins or rods are placed vertically into a wetland surface, and the change in substrate height (relative to pin) monitored over time, or the surface elevation measured over time	Days/Event – years	Inexpensive; can capture both positive (sedimentation) and negative (erosion) fluxes	Intensive monitoring including the placement of stable benchmarks; sensitive to disturbance; C content of deposited or eroded material needs to be estimated or measured	Saynor et al. (1994)
Surface elevation tables (SET)*	Permanent subsurface elevation benchmarks are monitored, providing high precision data on wetland surface change. Often paired with marker horizons	Years – decades	High precision measurements; captures accumulation and subsidence; global network	Intensive and expensive setup and monitoring, limiting replication; sensitive to disturbance; difficult to know C content of portion of subsurface that is changing	Webb et al. (2013), Lovelock et al. (2014)
Repeated measurement of C pool	The difference in soil C pool is compared in cores collected at two different points in time	Years – decades	Can integrate surface and subsurface processes; integrates intra- and inter-annual variability	Requires a long time frame of measurement, or availability of historical soil data	Lamont et al. (2020)
Space-for-time chronosequences	Differences in C pools are measured across a series of sampling locations that represent ages of ecosystem development (e.g., time since creation/restoration or primary succession following disturbance)	Years – millennia	Useful for long-term research questions; can integrate surface and sub-surface processes	Can be difficult to find appropriate study locations minimally influenced by confounding variables; sampling intensive	Pickett (1989), Osland et al. (2012), Kelleway et al. (2016), Marchand (2017), Krauss et al. (2018b)
Radiometric dating of isotopes	Calculation of an age-depth model of substrate evolution derived from the measurement of isotopes with known decay profiles	Decades – millennia	Provides long-term information	Expensive; computationally challenging	Jones et al. (2017)
Chronographic, stratigraphic approaches	Use specific, identifiable horizons in the soil profile with known ages associated with landcover change (i.e., chronohorizons)	Decades – millennia	Can link changes in environmental conditions (e.g., land use/land cover change) to distinct horizons	Requires detailed information of land-use history	Fenstermacher et al. (2016)

**Table 8:** Common detectors used for greenhouse gas (GHG) analyses by gas chromatography

Detector	GHG analyte	Required gases	Detection mechanism
Thermal Conductivity Detector (TCD)	CO_2_; can measure multiple gases	Carrier as reference gas	Measures difference between thermal conductivity of carrier gas plus analyte and that of pure carrier gas using a Wheatstone bridge
Flame Ionization Detector (FID)	CH_4_	Hydrogen and air (for combustion) and carrier as makeup gas	Ions are generated during analyte combustion in H_2_ flame, collected on an electrode and measured (Poole 2015)
Electron Capture Detector (ECD)	N_2_O	Nitrogen or argon/CH_4_ as makeup gas	Electrons from radioactive source collide with makeup gas creating free ions that generate a standing current. Electronegative analyte molecules capture electrons, reducing the standing current (Poole 2015)

*CO_2_* carbon dioxide, C*H_4_* methane, *N_2_O* nitrous oxide
