# Supplementary material for: Practical Guide to Measuring Wetland Carbon Pools and Fluxes
Source: Wetlands (Wilmington). Author manuscript; Available in PMC 2024 Nov 28. (PMC10684704; doi:10.1007/s13157-023-01722-2)
Supplement: Supplement1 [file NIHMS1948591-supplement-Supplement1.pdf]

Supplementary Information for Practical Guide to Measuring Wetland Carbon Pools and Fluxes

Sheel Bansal[1*], Irena F. Creed[2], Brian A. Tangen[1], Scott D. Bridgham[3], Ankur R. Desai[4], Ken W. Krauss[5], Scott C. Neubauer[6], Gregory B. Noe[7], Donald O. Rosenberry[8], Carl Trettin[9], Kimberly P. Wickland[10], Scott T. Allen[11], Ariane Arias-Ortiz[12], Anna R. Armitage[13], Dennis Baldocchi[14], Kakoli Banerjee[15], David Bastviken[16], Peter Berg[17], Matthew Bogard[18], Alex T. Chow[19], William H. Conner[20], Christopher Craft[21], Courtney Creamer[22], Tonya DelSontro[23], Jamie A. Duberstein[20], Meagan Eagle[24], M. Siobhan Fennessy[25], Sarah A. Finkelstein[26], Mathias Göckede[27], Sabine Grunwald[28], Meghan Halabisky[29], Ellen Herbert[30], Mohammad M.R. Jahangir[31], Olivia F. Johnson[1,32], Miriam C. Jones[7], Jeffrey J. Kelleway[33], Sara Knox[34], Kevin D. Kroeger[24], Kevin A. Kuehn[35], David Lobb[36], Amanda L. Loder[37], Shizhou Ma[38], Damien T. Maher[39], Gavin McNicol[40], Jacob Meier[1], Beth A. Middleton[5], Christopher Mills[41], Purbasha Mistry[38], Abhijit Mitra[42], Courtney Mobilian[21], Amanda M. Nahlik[43], Sue Newman[44], Jessica L. O'Connell[45], Patty Oikawa[46], Max Post van der Burg[1], Charles A. Schutte[47], Changchun Song[48], Camille L. Stagg[5], Jessica Turner[49], Rodrigo Vargas[50], Mark P. Waldrop[22], Marcus B. Wallin[51], Zhaohui Aleck Wang[52], Eric J. Ward[5], Debra A. Willard[7], Stephanie Yarwood[53], Xiaoyan Zhu[54]

*Corresponding author: Sheel Bansal, sbansal@usgs.gov

Affiliations

[1] U.S. Geological Survey, Northern Prairie Wildlife Research Center, Jamestown, ND, USA
[2] Department of Physical and Environmental Sciences, University of Toronto Scarborough, Toronto, ON, Canada
[3] Institute of Ecology and Evolution, University of Oregon, Eugene, OR, USA
[4] Department of Atmospheric and Oceanic Sciences, University of Wisconsin-Madison, Madison, WI, USA
[5] U.S. Geological Survey, Wetland and Aquatic Research Center, Lafayette, LA, USA
[6] Virginia Commonwealth University, Department of Biology, Richmond, VA, USA
[7] U.S. Geological Survey, Florence Bascom Geoscience Center, Reston, VA, USA
[8] U.S. Geological Survey, Water Mission Area, Lakewood, CO, USA
[9] U.S. Forest Service, Pacific Southwest Research Station, Davis, CA, USA
[10] U.S. Geological Survey, Geosciences and Environmental Change Science Center, Denver, CO, USA
[11] Department of Natural Resources and Environmental Science, University of Nevada, Reno, Reno, NV, USA
[12] Ecosystem Science Division, Department of Environmental Science, Policy and Management, University of California, Berkeley, CA, USA.
[13] Department of Marine Biology, Texas A&M University at Galveston, Galveston, TX, USA
[14] Department of Environmental Science, Policy and Management, University of California, Berkeley, CA, USA
[15] Department of Biodiversity and Conservation of Natural Resources, Central University of Odisha, Koraput, Odisha, India
[16] Department of Thematic Studies – Environmental change, Linköping University, Linköping, Sweden
[17] Department of Environmental Sciences, University of Virginia, Charlottesville, VA, USA

[18] Department of Biological Sciences, University of Lethbridge, Lethbridge, AB, Canada
[19] Earth and Environmental Sciences Programme, The Chinese University of Hong Kong, Shatin, Hong Kong SAR, China
[20] Baruch Institute of Coastal Ecology and Forest Science, Clemson University, Georgetown, SC, USA
[21] O'Neill School of Public and Environmental Affairs, Indiana University, Bloomington, IN, USA
[22] U.S. Geological Survey, Geology, Minerals, Energy and Geophysics Science Center, Menlo Park, CA, USA
[23] Department of Earth and Environmental Sciences, University of Waterloo, Waterloo, ON, Canada
[24] U.S. Geological Survey, Woods Hole Coastal & Marine Science Center, Woods Hole, MA, USA
[25] Departments of Biology and Environmental Studies, Kenyon College, Gambier, OH, USA
[26] Department of Earth Sciences, University of Toronto, Toronto, ON, Canada
[27] Max Planck Institute for Biogeochemistry, Department for Biogeochemical Signals, Jena, Germany
[28] Soil, Water and Ecosystem Sciences Department, University of Florida, Gainesville, FL, USA
[29] School of Environmental and Forest Sciences, University of Washington, Seattle, WA, USA
[30] Ducks Unlimited, Memphis, TN, USA
[31] Department of Soil Science, Bangladesh Agricultural University, Mymensingh, Bangladesh
[32] Department of Biological Sciences, Kent State University, Kent, OH, USA
[33] School of Earth, Atmospheric and Life Sciences and Environmental Futures Research Centre, University of Wollongong, NSW, Australia
[34] Department of Geography, McGill University, Montreal, Canada[35] School of Biological, Environmental, and Earth Sciences, University of Southern Mississippi, Hattiesburg, MS, USA
[36] Department of Soil Science, University of Manitoba, Winnipeg, MB, Canada
[37] Department of Geography, University of Toronto, Toronto, ON, Canada
[38] School of Environment and Sustainability, University of Saskatchewan, Saskatoon, SK, Canada; Shizhou Ma orcid (https://orcid.org/0000-0001-8407-6632), Purbasha Mistry orchid (https://orcid.org/0000-0001-9044-1149)
[39] Faculty of Science and Engineering, Southern Cross University, Lismore NSW, Australia
[40] Department of Earth and Environmental Sciences, University of Illinois at Chicago, Chicago, IL, USA
[41] U.S. Geological Survey, Geology, Geophysics, and Geochemistry Science Center, Denver, CO, USA
[42] Department of Marine Science, University of Calcutta, Kolkata, West Bengal, India
[43] U.S. Environmental Protection Agency, Office of Research and Development, Center for Public Health and Environmental Assessments, Pacific Ecological Systems Division, Corvallis, OR, USA
[44] South Florida Water Management District, Everglades Systems Assessment Section, West Palm Beach, FL, USA
[45] Department of Marine Science, University of Texas at Austin Marine Science Institute, Port Aransas, TX, USA

[46] Department of Earth and Environmental Sciences, California State University, East Bay, Hayward, CA, USA
[47] Department of Environmental Science, Rowan University, Glassboro, NJ, USA
[48] Key Laboratory of Wetland Ecology and Environment, Northeast Institute of Geography and Agroecology, Chinese Academy of Sciences, Changchun, China
[49] Freshwater and Marine Science, University of Wisconsin-Madison, Madison, WI, USA
[50] Department of Plant and Soil Sciences, University of Delaware, Newark, DE, USA
[51] Department of Aquatic Sciences and Assessment, Swedish University of Agricultural Sciences, Uppsala, Sweden
[52] Department of Marine Chemistry and Geochemistry, Woods Hole Oceanographic Institution, Woods Hole, MA, USA
[53] Environmental Science and Technology, University of Maryland, College Park, MD, USA
[54] Key Laboratory of Songliao Aquatic Environment, Ministry of Education, Jilin Jianzhu University, Changchun, China

Membrane inlet mass spectrometry (MIMS)

Membrane inlet mass spectrometry (also termed as 'membrane introduction mass spectrometry' or 'membrane interface mass spectrometry') (MIMS) was first used in 1963 (Hoch and Kok 1963) to separate volatile organic compounds from water or air by a thin membrane and has been employed in on-line and real-time analyses in industrial processes (e.g., fermentation, water chlorination) and environmental monitoring (e.g., urban air plumes, municipal tap water) (Ketola et al. 2002). Based on our knowledge, the first application of MIMS to wetland samples for determination of carbon dioxide (CO_2) and methane (CH_4) concentrations was conducted by Lloyd et al. (1986). Since then, MIMS has been used in the study of greenhouse gases (GHGs) in marine sediments (Bell et al. 2012), peat cores (Benstead and Lloyd 1996; Beckmann et al. 2004), wetland soils (Askaer et al. 2010; Elberling et al. 2011), terrestrial ecosystems, and grassland systems (Sheppard and Lloyd 2002).

This approach typically uses a semi-permeable polymer to enrich certain analytes from gaseous or liquid samples. As solutions tangentially cross the membrane, analytes are partitioned across the membrane while the bulk of the matrix is rejected. Analytes pass through the membrane at rates that depend on their solution concentration, their solubility in the membrane, and their diffusivity in the membrane. Analyte concentration is at maximum on the high-pressure side (sample side) of the membrane and falls to a minimal value on the vacuum side. These separated analytes are then directly transferred as mixtures (often using a helium carrier gas acceptor phase) to a mass spectrometer for their subsequent resolution and measurements.

The MIMS device consists of a vacuum inlet fitted with a permeable silicone tube. The inlet allows gas to pass into the vacuum system, where it is routed through a cold trap (typically dry ice) and into a quadrapole mass spectrometer. Water from samples or a standard is pumped through the membrane using a peristaltic pump. Partial pressure data are acquired on the data acquisition system in multiple ion-monitoring mode and can be processed using standard spreadsheet software.

Typically, a long stainless steel gas inlet capillary probe (1.56 mm outside diameter, 0.5 mm inside diameter) with a 50 µm diameter orifice near the tip, is used to insert into the soil core (Sheppard and Lloyd 2002). The advantage of MIMS is that it can be used to quantify a number of gas species, continuously and simultaneously, and it can record spatial and temporal variations in subsurface gas concentrations as low as 1 µM (Lloyd and Scott 1985; Lloyd and James 1987).

Based on the mass-to-charge ratio (m/Z) of characteristic positive ions of gases, a variety of gases can be monitored (e.g., m/z = 15: CH_4, m/z = 32: oxygen (O_2), m/z= 44: CO_2). This technique enables the direct measurement of multiple gas species throughout soil cores with minimal disturbance. The MIMS device is also a field portable instrument (Etzkorn et al. 2009). Perhaps the only disadvantage is the high operating cost for purchasing and maintaining the instrument. Although the instrument is considered portable, the gas chromatography–mass spectrometry power requirement access to remote areas is still a difficult task.

Macroalgae: Nutritional values and chemical analysis

Marine macroalgae (seaweeds, kelp) are an economically valued, renewable resource of food, biofuel, and biofertilizer. Food consumption of brown, red, and green macroalgae can be largely attributed to its nutrition properties, which also make it sought-after for fodder, fertilizer, cosmetics, and medicines (Robledo and Freile Pelegrín 1997; Dawes 1998; McHugh 2003; Banerjee et al. 2020). In terms of human consumption and nutrition, macroalgae are excellent sources of proteins, lipids, carbohydrates, minerals, vitamins, antioxidants, and phytochemicals, and thus provide numerous health benefits (Table S1; Parekh and Chauhan 1982; Kumari et al. 2010; Holdt and Kraan 2011; van Ginneken et al. 2011; Banerjee et al. 2020; Ganesan et al. 2020; Lozano Muñoz and Díaz 2022). Globally, it is estimated that around 8 million tons of macroalgae are harvested annually to support its many uses (McHugh 2003). The exploitation of marine algae for nutritional purposes is primarily based on its biochemical constituents (Parekh and Chauhan 1982). Macroalgae show great variation in nutrient content based on species, level of maturity, geographical distribution, and environmental conditions like seawater temperature, salinity, light, and nutrients (Dawes 1998).

Protein, carbohydrate, lipid, and astaxanthin chemical analyses

Protein analysis following the method originally from Lowry et al. (1951). About 0.1 g of powered macroalgae is extracted with trisodium phosphate (Na_3PO_4) (buffer pH = 0.7) and centrifuged. An aliquot of sample extract is added to a reagent of sodium carbonate (Na_2CO_3) and another reagent of copper(II) sulfate ($CuSO_4$). Then, Folin-Ciocalteu phenol reagent (2:1) is added and left undisturbed for 30 minutes for color development. The intensity of the color is measured at 660 nm. For quantifying the protein content of the sample, a standard curve is prepared with a known concentration of bovine serum albumin as standard. The value is expressed in percentage. For additional information regarding protein measurement using the Folin phenol reagent (Lowry et al. 1951) see reviews by Peterson (1979) and Singleton et al. (1999), an application by Ledoux and Lamy (1986), and an assessment of the Folin-Ciocalteu reagent by Everette et al. (2010).

Carbohydrate content can be estimated by using the procedure of Sadasivam and Manickam (2007). Dried macroalgae powder (0.1 g) is extracted with 80% methanol and centrifuged. This extraction is repeated twice, and the pooled supernatant is evaporated until the methanol is removed. The sample extract is then combined with anthrone reagent and the absorbance is measured at 630 nm using a spectrophotometer. The value is expressed as mg g^{-1} (dry weight) or percentage using glucose as standard.

The lipid contents of dried macroalgal samples can be determined by continuous extraction in a lipid extractor (Soxhlet Apparatus, Folch et al. 1957) for 3 hours using petroleum ether as a solvent. Astaxanthin content can be estimated using the procedure of (Banerjee et al.

2009). Dried powdered seaweed is extracted with dimethyl sulfoxide and centrifuged until the extract is colorless. Absorbance is measured at 471 to 477 nm.

Macroalgal biomass often varies seasonally and can be affected by several abiotic and biotic factors such as salinity, temperature, pH, and nutrient concentrations (Banerjee et al. 2009). Thus, it is important to collect key covariates and ancillary variables when sampling macroalgae (Fig. S1).

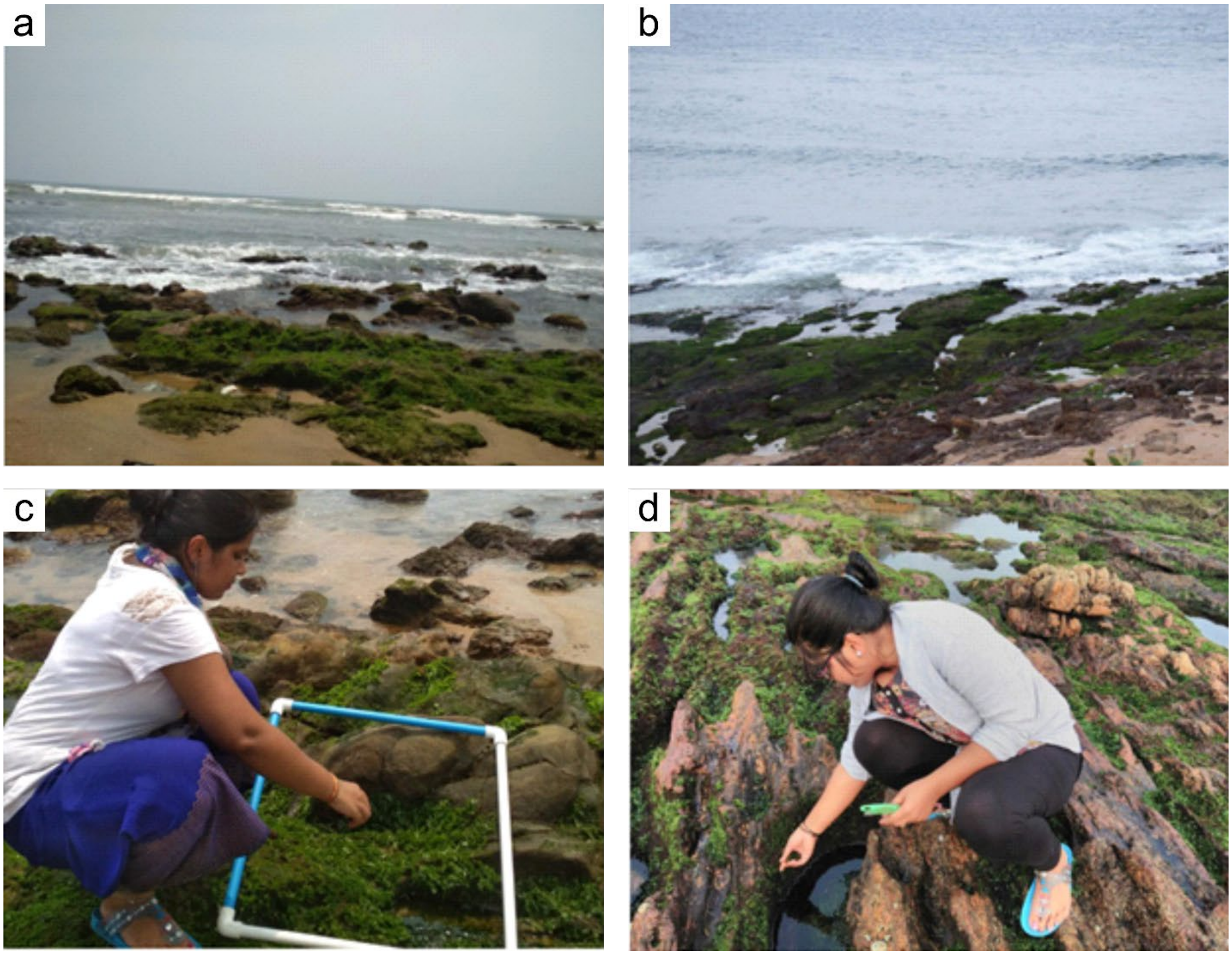

Fig. S1 Macroalgal (seaweed) growth on rocky surfaces along the coast of India. Student Prajna Paramita Mohapatra (Banerjee lab) collecting macroalgae (seaweeds) by hand scraping biomass from within a sample quadrat from Vishakhapatnam coast of Andhra Pradesh in western Bay of Bengal, India. Images with permission from Banerjee and Mohapatra

Table S1. Health benefits of macroalgae, see Lozano Muñoz and Díaz (2022) and citations within. Photo credit: Kakoli Banerjee

**Constituent**	**Percentages**	
Nutrients	Protein: 12%<br>Calories: 2%<br>Carbohydrate: 2%<br>Dietary Fiber: 1%	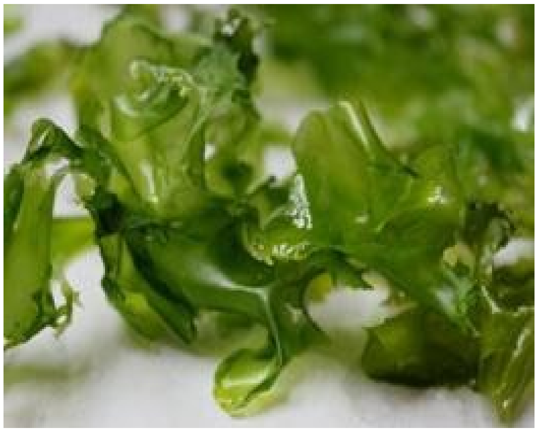
Vitamins	Vitamin A: 104%<br>Vitamin C: 65%<br>Folate: 37%<br>Riboflavin: 10%	
Minerals	Manganese: 49%<br>Copper: 13%<br>Iron: 10%<br>Potassium: 10%	
**Health Benefits**		
Useful in maintaining healthy digestion		Helps to prevent colon cancer and leukemia
Protects skin against harmful effects of ultraviolet B radiation and slows down aging process		
Effective in exerting anti-diabetic effects		Helps to detoxify and cleanse body
Reduces risk of mental deliberation and goiter hypothyroidism		
Helps to strengthen eyes and hairs		Benefits in improving heart and dental health
Prevents threat of stroke and coagulation		Helps to protect against influenza B virus
Caution: Excess intake may raise levels of thyroid-stimulation hormone. Avoid usage during pregnancy and lactation.		
% Daily value per 100 g of seaweed (laver) provides 65% of daily requirement of vitamin C.		

Disclaimer: Any use of trade, firm, or product names is for descriptive purposes only and does not imply endorsement by the U.S. Government.

References

Askaer L, Elberling B, Glud RN, Kühl M, Lauritsen FR, Joensen HP (2010) Soil heterogeneity effects on O_2 distribution and CH_4 emissions from wetlands: In situ and mesocosm studies with planar O_2 optodes and membrane inlet mass spectrometry. Soil Biology and Biochemistry 42:2254–2265. https://doi.org/10.1016/j.soilbio.2010.08.026

Banerjee K, Ghosh R, Homechaudhuri S, Mitra A (2009) Seasonal variation in the biochemical composition of red seaweed (*Catenella repens*) from Gangetic delta, northeast coast of India. Journal of Earth System Science 118:497–505. https://doi.org/10.1007/s12040-009-0045-2

Banerjee K, Turuk AS, Paul R (2020) Seaweed: The blue crop for food security as mitigation measure to climate change. Plant Archives 20:2045–2054. http://plantarchives.org/20-1/2045-2054%20(5892).pdf

Beckmann M, Sheppard SK, Lloyd D (2004) Mass spectrometric monitoring of gas dynamics in peat monoliths: effects of temperature and diurnal cycles on emissions. Atmospheric Environment 38:6907–6913. https://doi.org/10.1016/j.atmosenv.2004.08.004

Bell RJ, Savidge WB, Toler SK, Byrne RH, Short RT (2012) In situ determination of porewater gases by underwater flow-through membrane inlet mass spectrometry. Limnology and Oceanography: Methods 10:117–128. https://doi.org/10.4319/lom.2012.10.117

Benstead J, Lloyd D (1996) Spatial and temporal variations of dissolved gases (CH_4, CO_2, and O_2) in peat cores. Microbial Ecology 31:57–66. https://doi.org/10.1007/BF00175075

Dawes CJ (1998) Marine botany, 2nd edn. John Wiley & Sons, Inc., New York

Elberling B, Askaer L, Jørgensen CJ et al (2011) Linking soil O_2, CO_2, and CH_4 concentrations in a wetland soil: Implications for CO_2 and CH_4 fluxes. Environmental Science & Technology 45:3393–3399. https://doi.org/10.1021/es103540k

Etzkorn JM, Davey NG, Thompson AJ et al (2009) The use of MIMS-MS-MS in field locations as an on-line quantitative environmental monitoring technique for trace contaminants in air and water. Journal of Chromatographic Science 47:57–66. https://doi.org/10.1093/chromsci/47.1.57

Everette JD, Bryant QM, Green AM, Abbey YA, Wangila GW, Walker RB (2010) Thorough study of reactivity of various compound classes toward the Folin−Ciocalteu reagent. Journal of Agricultural and Food Chemistry 58:8139–8144. https://doi.org/10.1021/jf1005935

Folch J, Lees M, Stanley GHS (1957) A simple method for the isolation and purification of total lipides from animal tissues. Journal of biological chemistry 226:497–509. https://doi.org/10.1016/S0021-9258(18)64849-5

Ganesan AR, Subramani K, Shanmugam M et al (2020) A comparison of nutritional value of underexploited edible seaweeds with recommended dietary allowances. Journal of King Saud University - Science 32:1206–1211. https://doi.org/10.1016/j.jksus.2019.11.009

Hoch G, Kok B (1963) A mass spectrometer inlet system for sampling gases dissolved in liquid phases. Archives of Biochemistry and Biophysics 101:160–170. https://doi.org/10.1016/0003-9861(63)90546-0

Holdt SL, Kraan S (2011) Bioactive compounds in seaweed: Functional food applications and legislation. Journal of Applied Phycology 23:543–597. https://doi.org/10.1007/s10811-010-9632-5

Ketola RA, Kotiaho T, Cisper ME, Allen TM (2002) Environmental applications of membrane introduction mass spectrometry. Journal of Mass Spectrometry 37:457–476. https://doi.org/10.1002/jms.327

Kumari P, Kumar M, Gupta V, Reddy CRK, Jha B (2010) Tropical marine macroalgae as potential sources of nutritionally important PUFAs. Food Chemistry 120:749–757. https://doi.org/10.1016/j.foodchem.2009.11.006

Ledoux M, Lamy F (1986) Determination of proteins and sulfobetaine with the folin-phenol reagent. Analytical Biochemistry 157:28–31. https://doi.org/10.1016/0003-2697(86)90191-0

Lloyd D, Davies KJP, Boddy L (1986) Mass spectrometry as en ecological tool for in situ measurement of dissolved gases in sediment systems. FEMS Microbiology Ecology 2:11–17. https://doi.org/10.1111/j.1574-6968.1986.tb01933.x

Lloyd D, James CJ (1987) The Pasteur effect in yeasts: Mass spectrometric monitoring of oxygen uptake, and carbon dioxide and ethanol production. FEMS Microbiology Letters 42:27–31. https://doi.org/10.1111/j.1574-6968.1987.tb02294.x

Lloyd D, Scott RI (1985) Mass spectrometric monitoring of dissolved gases. In: Poole RK and Dow CS (eds) Microbial gas metabolism: Mechanistic, metabolic, and biotechnological aspects Academic Press, New York, pp 304

Lowry OH, Rosebrough NJ, Farr AL, Randall RJ (1951) Protein measurement with the Folin phenol reagent. Journal of biological chemistry 193:265–275

Lozano Muñoz I, Díaz NF (2022) Minerals in edible seaweed: Health benefits and food safety issues. Critical Reviews in Food Science and Nutrition 62:1592–1607. https://doi.org/10.1080/10408398.2020.1844637

McHugh DJ (2003) A guide to the seaweed industry. FAO Fisheries Technical Paper 441. http://www.fao.org/3/y4765e/y4765e00.htm

Parekh RG, Chauhan VD (1982) Seasonal variations in the chemical constituents of the marine alga *Ulva* sp. and seawater. Seaweed Research and Utilization 5:1–4

Peterson GL (1979) Review of the folin phenol protein quantitation method of lowry, rosebrough, farr and randall. Analytical Biochemistry 100:201–220. https://doi.org/10.1016/0003-2697(79)90222-7

Robledo D, Freile Pelegrín Y (1997) Chemical and mineral composition of six potentially edible seaweed species of Yucatán. Botanica Marina 40:301–306. https://doi.org/10.1515/botm.1997.40.1-6.301

Sadasivam S, Manickam A (2007) Biochemical methods, 3rd edn. New Age International Pvt. Ltd, New Delhi

Sheppard SK, Lloyd D (2002) Effects of soil amendment on gas depth profiles in soil monoliths using direct mass spectrometric measurement. Bioresource Technology 84:39–47. https://doi.org/10.1016/S0960-8524(02)00012-3

Singleton VL, Orthofer R, Lamuela-Raventós RM (1999) [14] Analysis of total phenols and other oxidation substrates and antioxidants by means of folin-ciocalteu reagent. In: Packer L (ed) Methods in Enzymology—Volume 299: Oxidants and Antioxidants Part A. Academic Press, pp 152–178. https://doi.org/10.1016/S0076-6879(99)99017-1

van Ginneken VJT, Helsper JPFG, de Visser W, van Keulen H, Brandenburg WA (2011) Polyunsaturated fatty acids in various macroalgal species from north Atlantic and tropical seas. Lipids in Health and Disease 10:104. https://doi.org/10.1186/1476-511X-10-104
